# Mechanochemical nitration of arenes and alcohols using a bench-stable organic nitrating reagent†

**DOI:** 10.1039/d5gc02232k

**Published:** 2025-05-28

**Authors:** Vasiliki Valsamidou, Subrata Patra, Besa Kadriu, Michel Gaspard Metzger, Ludovic Gremaud, Dmitry Katayev

**Affiliations:** a Department of Chemistry, Biochemistry and Pharmaceutical Sciences, University of Bern Freiestrasse 3 3012 Bern Switzerland dmitry.katayev@unibe.ch; b Haute école d'ingénierie et d'architecture de Fribourg Boulevard de Pérolles 80 1700 Fribourg Switzerland

## Abstract

The installation of a nitro group, essential for synthesizing valuable nitrated compounds, is traditionally associated with harsh reaction conditions, hazardous reagents, and significant environmental concerns. Recent advancements in sustainable nitration methodologies have led to the development of environmentally benign, mild, and non-acidic nitrating reagents, which are often derived from an organic scaffold and can be recycled after the completion of the process. In this study, we demonstrate the practical application of saccharin-derived reagents in mechanochemical electrophilic nitration, utilizing vibratory ball milling under LAG (Liquid-Assisted Grinding) conditions to efficiently functionalize a wide array of alcohols and arenes. This method decreases solvent usage while preserving high selectivity and reactivity, enhancing green chemistry metrics, and fostering greater sustainability in nitration protocols.

## Abstract 2

Rapid, efficient, and sustainable protocols for the electrophilic nitration of various alcohols and aromatic compounds under mechanochemical conditions have been introduced.

## Graphical abstract

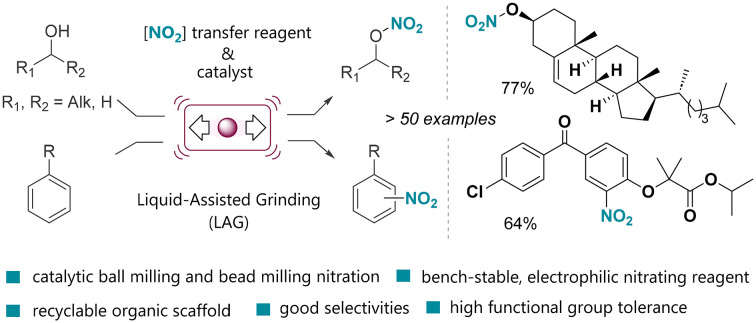

**Green foundation**

1. This work advances green chemistry by developing a milder, more sustainable electrophilic nitration protocol using mechanochemistry. Unlike traditional methods that rely on hazardous reagents and harsh conditions, this approach employs a benign organic nitrating agent under solvent-minimized ball milling conditions. The energy-efficient and straightforward nature of mechanochemistry significantly enhances green metrics compared to conventional solvent-based processes.

2. The key innovation lies in using a saccharin-based, bench-stable nitrating reagent for the efficient nitration of alcohols and arenes under mechanochemical conditions. This method provides superior green metrics compared to similar electrophilic nitration approaches and is scalable to the gram scale through bead milling, demonstrating practical applicability and flexibility with various milling techniques.

3. A current limitation is the need for higher mechanical energy to achieve good yields with electron-deficient substrates. Proof-of-concept bead milling suggests that advanced setups could improve energy transfer and reaction efficiency. Switching to inert, corrosion-resistant materials like zirconia may reduce metal contamination, enhance reactivity through denser media, and extend equipment lifespan, supporting greener processing.

## Introduction

The nitro group is critical in organic chemistry and serves as a key functionality in numerous pharmaceuticals,^1^ dyes,^2^ and energetic materials.^3^ Its unique ability to modulate the physicochemical properties of molecules, coupled with its versatility as a precursor for diverse compounds, such as amines, hydroxylamines, aldehydes, carboxylic acids, isocyanates, and heterocycles^4^—establishes it as a pivotal part of a chemist's toolbox in academia and industry. Aromatic nitro compounds have historically been important, especially in developing dyes and explosives. In the late 1950s, the antibiotic chloramphenicol was discovered,^1*c*^ representing the first natural product identified to contain a nitro group. This breakthrough extensively highlighted the potential of nitrated aromatic compounds within the pharmaceutical industry. In addition to the vast array of biologically active nitroarene compounds currently available, nitrate esters have emerged as a subject of interest among synthetic chemists. These esters are highly versatile functional groups and intermediates in synthetic chemistry,^5^ with applications in propellants and pharmaceuticals, particularly as vasodilators.^6^

With today's environmental challenges in mind, organic chemists actively develop and refine synthetic methodologies to enhance their sustainability. The ecological impact of chemical processes is often assessed using quantitative metrics such as atom economy,^7^ as well as a semi-quantitative process metrics tool EcoScale,^8*b*^ which has since evolved into part of the broader framework of green chemistry principles.^8^ By prioritizing greener alternatives and adhering to these principles, chemists are paving the way for a future where chemical innovation supports both environmental health and industrial progress. Introducing a nitro group in this context presents considerable challenges, as conventional nitration approaches frequently conflict with sustainable development goals (SDG's).^8^ The traditional approach to nitro group installation, particularly electrophilic aromatic nitration *via* the “mixed acid” method (HNO_3_/H_2_SO_4_), remains prevalent.^9^ While effective, this method poses significant environmental and safety challenges. The use of toxic and explosive nitrating mixtures, along with the generation of mineral acid waste, creates critical drawbacks. Furthermore, the harsh reaction conditions significantly reduce functional group tolerance, often leading to undesirable by-products, over-nitration, and excessive waste generation. Alternative electrophilic nitrating agents, such as acyl nitrates, nitryl halides, and nitronium salts,^9*a*,10^ have been developed to address these issues. However, their broader application is hindered by several limitations, including high sensitivity to moisture and air and susceptibility to thermal decomposition, which pose significant handling and stability concerns.

**Scheme 1:**
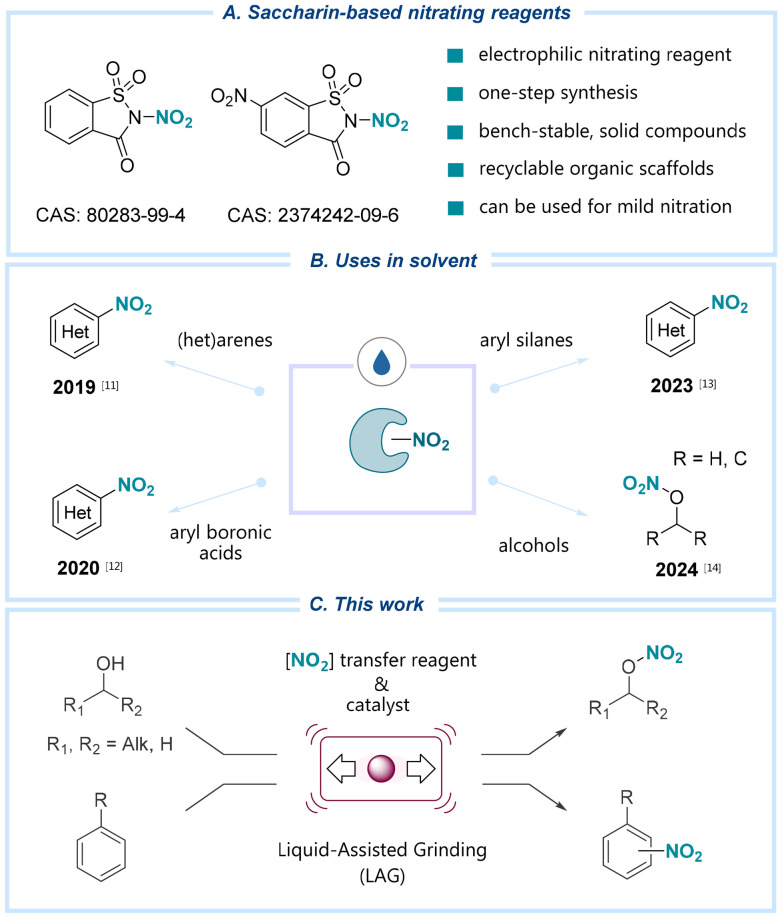
(A) Nitrating reagents based on saccharin scaffold. (B) Overview of applications of these reagents under solvent-based conditions. (C) This work: nitration of alcohols and arenes using dinitrosaccharin under mechanochemical and LAG conditions, catalyzed by a Lewis acid.

In response to these challenges, our research group introduced mild and recyclable nitrating reagents based on succinimide and saccharin scaffolds (Scheme 1A).^11^ These reagents act as precise, controllable sources of nitryl radicals and nitronium ions and have demonstrated efficacy in the radical-mediated nitration of alkenes,^12^ the electrophilic nitration of arenes, the *ipso*-substitution of aryl and heteroarylboronic acids,^13^ organosilanes,^14^ and, more recently, the functionalization of alcohols (Scheme 1B).^15^ To further enhance the sustainability of our protocols, we recently sought to reduce the environmental footprint by integrating mechanochemistry into our synthetic approaches.^16^ This impact- and friction-driven technique, which uses minimal to no solvents, has been widely recognized for its resource efficiency and alignment with green chemistry principles.^17^ In addition, mechanochemical techniques offer advantages over traditional solution-based methods, such as shorter reaction times, improved or divergent selectivity, and simplicity of operation.^18^

Building on the principle of sustainable synthesis and encouraged by the excellent reactivity of N-nitroheterocyclic reagents in solution,^15^ we aimed to translate their reactivity to mechanochemical conditions (Scheme 1C). Although mechanochemical nitration protocols have been previously reported, they typically require a large excess of inorganic reagents, resulting in significant waste generation.^19^ Herein, we present our findings demonstrating that using vibratory ball milling, the saccharin-based reagent NN facilitates efficient electrophilic nitration of a broad range of alcohols and aromatic compounds under liquid-assisted grinding (LAG) conditions. This mechanochemical technique provides distinct advantages, including reduced solvent consumption, shorter reaction times, a simplified operational procedure, and excellent functional group (FG) tolerance compared to conventional solution-phase methods. Furthermore, we successfully demonstrated the scalability of this methodology by employing bead mill technology for larger-scale synthesis.

## Results and discussion

We began our investigation by studying the nitration of 4-nitrophenethyl alcohol, as its corresponding O-nitrated adduct exhibits enhanced stability. The optimization was conducted in a 10 mL stainless steel jar using three stainless steel balls (∅ = 12 mm) in the Retsch Mixer Mill MM 500 Vario. Following the initial screening of conditions, we were pleased to observe that subjecting alcohol 2-(4-nitrophenyl)ethan-1-ol to the nitrating reagent NN under Lewis acid catalysis resulted in the nitrated compound 4 (Table 1). Alternative organic nitrating reagents previously developed in our group did not provide any nitrated product (Table 1, entry 2). A range of Lewis acids was also evaluated (entries 3 and 4, see ESI for details, page S4†), with Sc(OTf)_3_ identified as the most suitable for catalyzing alcohol nitration, yielding the nitrate ester with a 90% yield (Table 1, entry 1). Without Lewis acid, the reaction proceeded but resulted in a significantly reduced yield (entry 5). Similarly, the omission of hexafluoroisopropanol (HFIP)—an additive known to facilitate nitro-group transfer *via* coordination^11*a*^—considerably reduced the product yield (entry 6). Other solvents, such as MeOH, assisted in lubricating the solids within the reaction, leading to lower yields (entry 7). To evaluate the impact of energy on reactivity, a comparative experiment was conducted in which the reaction mixture was manually ground using a mortar and pestle for 10 minutes, yielding 32% (entry 8). This suggests that effective mixing, combined with appropriately tuned mechanical force and controlled energy input, is essential for achieving the desired reactivity. The reaction was performed using a PTFE milling jar and balls to exclude potential catalytic effects of metal particles introduced through the mechanical wear of stainless-steel milling components. The reaction proceeded as expected, albeit with a lower yield, likely due to differences in material density and, consequently, the level of energy transfer (Table 1, entry 9). This hypothesis was further confirmed during scale-up activities with the Dyno®-mill using exclusively ZrO_2_/Y_2_O_3_ beads and a milling chamber.

**Table 1:** Reaction development and optimization

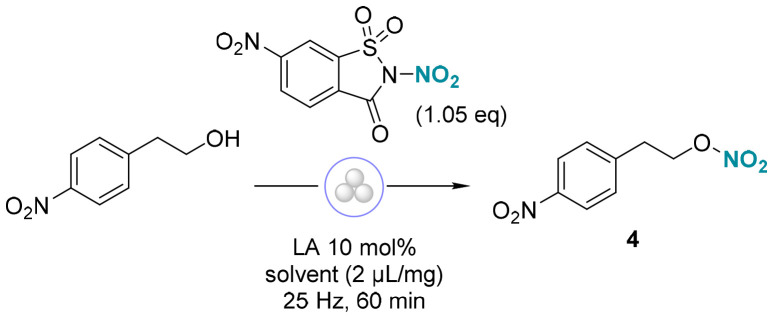		
Entry	Variation from standard conditions^a^	4 (%)
1	None	90
2	Reagent I-III^b^	—
3	Zn(OTf)_2_	24
4	Ca(OTf)_2_	53
5	No Lewis acid	19
6	No HFIP additive	12
7	MeOH^c^	62
8	Ground by hand (10 min)	32
9	PTFE jar	71
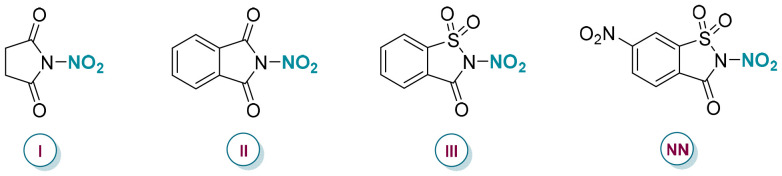		

a Standard reaction conditions: 4-nitrophenethyl alcohol (0.3 mmol, 1.0 eq.), Sc(OTf)_3_ (10 mol%), reagent NN (1.05 eq.), HFIP (2 μL mg^−1^), stainless steel jar (10 mL), stainless steel balls (3 × 12 mm), 25 Hz, 3 h.

b Instead of NN reagent.

c Instead of HFIP solvent additive.

With the optimized reaction conditions established, the ball milling nitration protocol was applied to various alcohol substrates. Initially, primary unactivated alcohols containing aromatic moieties were selected as targets and demonstrated high suitability as substrates (1–10, 15). Both electron-rich (5–6) and electron-poor (4) aryl-substituted alcohols yielded nitrated products in good to excellent yields, while the reaction tolerated alkyl (3, 6), methoxy (5), and halogen (7) substituents. The nitration of the aromatic ring occurred in the case of 6, which bears three methyl groups. Due to the resonance stabilization effect at the benzylic position, nitrated benzyl alcohols decomposed into the respective aldehydes and could not be isolated. Alcohols with nitro (8), bromide (9), or azide (10) substituents in the α-position underwent nitration with yields of up to 92%, indicating that these functional groups do not impede the reaction. Furthermore, a series of aliphatic alcohols (11–14, 16–17) were tested, and their nitration occurred with high efficiency, resulting in very good to excellent yields. Notably, functionalities such as chloride (11), bromide (14), alkyne (12), and alkene (13) groups, which are typically sensitive to conventional nitration methods, remain unaffected under the ball milling conditions. Secondary alcohols (15–17) were also successfully nitrated, yielding high returns. Despite the significant deactivation caused by the phthalimide moiety, we were pleased to observe the efficient nitration of compound 18. The nitration of tryptophol 19 yielded satisfactory results, likely constrained by interference from its free amine functional group. Complex molecules 20 and 21 were nitrated with yields reaching up to 77%. Interestingly, the primary alcohol in molecule 20 underwent nitration faster than the secondary alcohol, while the olefin moiety in 21 remained unreacted.

**Scheme 2:**
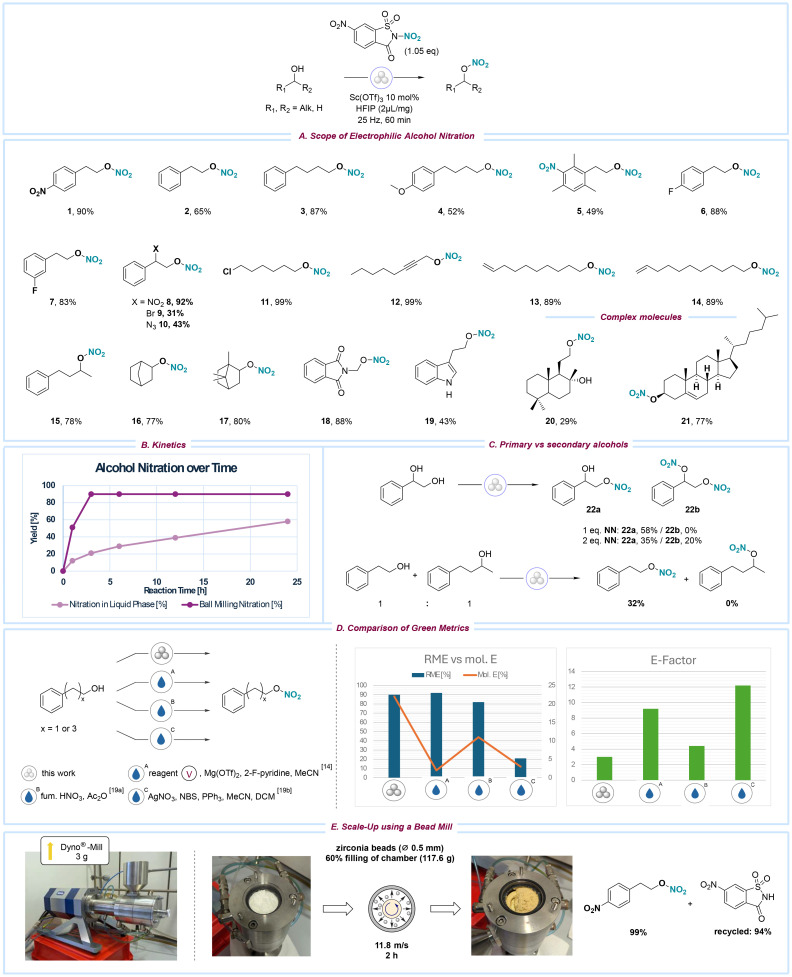
(A) Substrate scope for the electrophilic nitration of alcohols using standard reaction conditions. (B) Yield development of solution-phase and ball milling reaction over time. (C) Evaluation of selectivity between primary (1°) and secondary (2°) alcohols. (D) Evaluation and comparison of reaction mass efficiency (RME), molar efficiency (mol. E.), and *E*-factor of the ball milling nitration of alcohols against alternative solution-phase approaches.^15,20^ RME = 90%, mol. E. = 22%, *E*-factor = <3.0. (E) Depiction of WAB-GROUP® Dyno®-mill set-up used for scale-up.

When comparing reaction times, the ball milling conditions demonstrated a significantly reduced reaction time compared to the equivalent reaction conducted in acetonitrile, likely due to the elimination of solvent-induced dilution effects (Scheme 2B). Specifically, the ball milling approach achieved a higher maximum yield (90%) within 3 hours, whereas the solvent-based reaction required 24 hours to reach completion (58%). To evaluate the selectivity of the ball milling nitration protocol for primary *versus* secondary alcohols simultaneously present in the same environment, reactions were conducted with phenylethane-1,2-diol, as well as with a mixture of 2-phenylethanol and 4-phenylbutan-2-ol in a single reaction vessel (Scheme 2C). In both scenarios, nitration preferentially occurred at the primary alcohol. Functionalization of the secondary alcohol was observed only when the amount of the nitrating reagent exceeded one equivalent.

Next, to assess the environmental impact of our mechanochemical nitration methodology, we evaluated its performance using key green chemistry metrics, including reaction mass efficiency (RME), molar efficiency (mol. E.), and the environmental factor (*E*-factor) (Scheme 2D). These metrics were compared against established nitration protocols^15,20^ (for more details, see ESI, page S6†). The ball milling procedure demonstrated a high RME of 90%, comparable to the previously reported solvent-based nitration using NN (92%) and the traditional “mixed acid” method (82%). However, when considering molar efficiency, the ball milling approach significantly outperformed all other methods, achieving 22% compared to 2%, 11%, and 3% for the solvent-based NN, mixed acid, and AgNO_3_-based protocols, respectively, demonstrating a more efficient use of reactants.

Furthermore, the *E*-factor for the ball milling process was markedly lower (3.0) than those associated with other nucleophilic nitration methodologies (9.2, 4.4, and 12.2, respectively), primarily due to the substantial reduction in solvent usage—a key advantage of mechanochemical techniques. While these three green metrics provide valuable insights into the sustainability of the respective protocols, it is essential to note that they do not account for the intrinsic hazards associated with the reagents used, which is particularly relevant in the case of the mineral acid-based nitration concepts.

To assess the protocol's scalability, we explored alternative milling tools to evaluate the potential of mechanochemical processes at larger scales. Subsequently, the WAB-GROUP® Dyno®-mill, part of the bead-mill technologies, was employed to carry out the nitration of 4-nitrophenethyl alcohol, selected as the model substrate (Scheme 2E). It is hypothesized that although ball milling offers a rapid and efficient method for this transformation, scaling up with larger substrate quantities, more numerous and denser milling media, and the increased energy input of a bead mill may create more favourable conditions for the reaction. Indeed, when 3 g of the alcohol substrate was subjected to bead-mill conditions (60% filling chamber with ZrO_2_/Y_2_O_3_ beads), the reaction proceeded efficiently, delivering an excellent 99% in just 2 hours (Scheme 2E). This significant enhancement in yield and reaction time highlights the potential for further optimization and scalability of the process, paving the way for improved efficiency in laboratory research and industrial manufacturing settings. Notably, conducting the reaction on a larger scale offers the opportunity for HFIP recycling, further improving the sustainability of the protocol.^21^

**Scheme 3:**
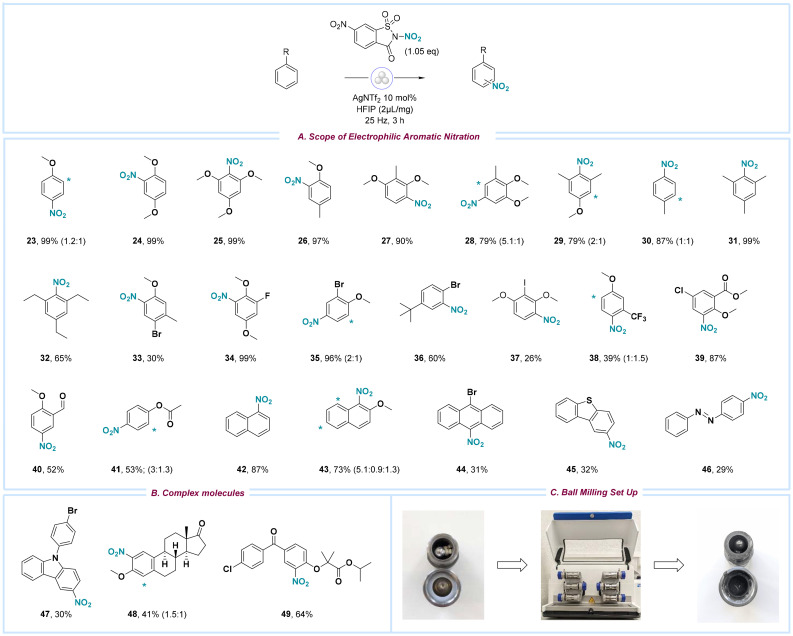
(A) Substrate scope for electrophilic aromatic nitration using NN as nitrating reagent. Standard reaction conditions: 4-nitrophenethyl alcohol (0.3 mmol, 1.0 eq.), AgNTf_2_ (10 mol%), reagent NN (1.05 eq.), HFIP (2 μL mg^−1^), stainless steel jar (10 mL), stainless steel balls (3 × 12 mm), 25 Hz, 3 h. (B) Scope of complex molecules. (C) Representative set-up with the Retsch Mixer Mill MM 500 vario.

Next, we focused on the possibility of performing aromatic nitration under ball milling reaction conditions. Similar to developing the alcohol nitration protocol, we started by optimizing the reaction conditions, primarily focusing on identifying an effective catalyst system for reagent activation. It was hypothesized that, unlike alcohols, the nitration of arenes—lacking coordinating groups to facilitate nitronium ion transfer—would require a catalyst with enhanced Lewis acidity to effectively promote the reaction (for details, see ESI page S5†). Following extensive screening, silver bis(trifluoromethane-sulfonyl)imide (AgNTf_2_) at a loading of 10 mol% was identified as the most effective catalyst, in combination with the nitrating reagent NN (1.05 equiv.), hexafluoroisopropanol (HFIP, 2 μL mg^−1^), and ball milling in a 10 mL stainless steel jar using three stainless steel balls (∅ = 12 mm) at 25 Hz for 3 hours. The optimized conditions were subsequently evaluated for their general applicability concerning the nucleophilicity of various aromatic scaffolds and their functional group tolerance (Scheme 3). As expected, electron-rich aromatic compounds with methoxy or alkyl substituents underwent efficient electrophilic nitration with common *o*-, *m*-, and *p*-patterns, resulting in good to excellent yields of the desired nitroarenes (23–32). Arenes with sensitive halide groups (33–37) were tolerated as well. Nevertheless, the *ipso*-substitution of iodide and the complex mixtures resulting from the nitration of an electron-rich bromoarene yielded low amounts of 37 and 33, respectively. In cases where the electronic characteristics of the substrate directed substitution to a single position, yields as high as 99% were achieved (34). Substrates containing electron-withdrawing groups, such as trifluoromethyl (38), ester (39), and aldehyde moieties (22), were nitrated only in the presence of electron-donating substituents on the same aromatic framework. Additionally, polycyclic aromatic hydrocarbons, including naphthalene (42–43) and anthracene derivatives (44), as well as diazobenzene (46) and the heterocycle dibenzothiophene (45), were successfully functionalized.

In comparison, electronically unconstrained scaffolds exhibited low toposelectivity, resulting in intricate mixtures of nitration products. Similarly, aromatic compounds bearing unprotected groups such as aniline, benzoic acid, and phenol resulted in low product yields along with the formation of by-products. To showcase the method's utility in late-stage functionalization, the protocol was implemented on complex and biologically active structures (47–49), yielding nitration rates as high as 64%.

**Scheme 4:**
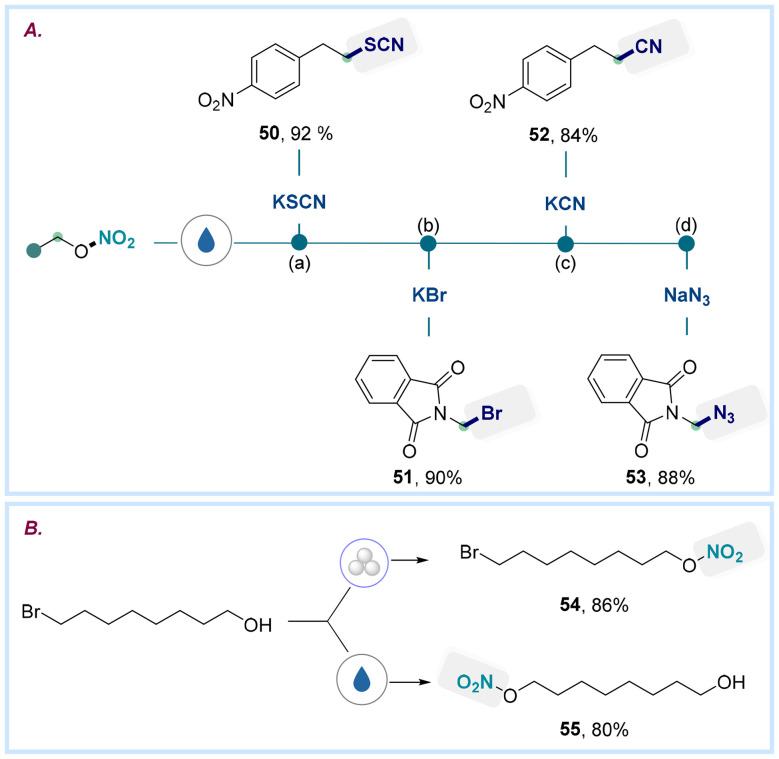
(A) Post-functionalizations of nitrate esters to access valuable functionalities. (B) Divergent functionalization of bromo alcohol 8-bromooctan-1-ol using mechanochemical (ball milling) and solution-phase methodologies.

Due to the crucial role of nitrate esters in pharmaceuticals, energetic materials, and atmospheric processes, developing efficient synthetic routes remains highly relevant. We conducted a series of derivatizations to explore their synthetic utility using carefully selected nucleophiles to access valuable building blocks (Scheme 4A). For example, the newly introduced nitrate group can be sequentially substituted with thiocyanide, yielding compound 50 in 92% yield. Additionally, nucleophiles such as azide (53), cyanide (52), and bromide (51) enable the direct transformation of nitrate esters into versatile synthetic handles under standard reaction conditions. As depicted in Scheme 4B, our alcohol nitration protocol, utilizing 8-bromooctan-1-ol and NN as the reagent, successfully produced the desired nitrate ester 54 with an 86% yield. In contrast, the traditional nucleophilic method employing silver nitrate yielded 8-(nitrooxy)octan-1-ol (55) at an 80% yield. This ability to modulate selectivity highlights the complementary nature of our alcohol nitration strategy to the conventional nucleophilic nitrate formation method.

## Conclusions

In summary, rapid and efficient protocols for the electrophilic nitration of various alcohols and aromatic compounds under mechanochemical conditions have been developed. A recyclable organic nitrating reagent based on an inexpensive saccharin core, which was previously employed only in solvent-based systems, has been successfully activated under ball mill conditions in the presence of a Lewis acid catalyst and a minimal amount of solvent. While the reaction mechanism and selectivity using this nitrating reagent under ball milling conditions are consistent with the liquid-phase protocols, this approach significantly enhances green metrics, shortens reaction times, and simplifies operational procedures. Additionally, bead mill technology has proven to be highly compatible with the nitrating reagent NN, allowing for the scaling up of nitration processes. This finding is particularly noteworthy as it highlights the method's potential for broad applicability in both industrial-scale and academic laboratory settings. Our group's ongoing research focuses on applying these bench-stable organic nitrating reagents to efficiently nitrate various molecules using different mechanochemical milling techniques.

## Author contributions

D. K. conceptualized the project. V. V., S. P., B. K., and M. G. M. performed the experiments and wrote the ESI.† V. V. & L. G. carried out the development of scale-up synthesis using bead mill technology. V. V. & D. K. wrote the manuscript.

## Conflicts of interest

There are no conflicts to declare.

## Supplementary Material

GC-027-D5GC02232K-s001

## Data Availability

The data supporting this article have been included as part of the ESI.†
