# Supplementary material for: Mechanochemical nitration of arenes and alcohols using a bench-stable organic nitrating reagent
Source: Green Chem. 2025 May 28;27(24):7122–8. doi: 10.1039/d5gc02232k (PMC12132030; doi:10.1039/d5gc02232k)
Supplement: GC-027-D5GC02232K-s001 [file GC-027-D5GC02232K-s001.pdf]

Supplementary Information (SI) for Green Chemistry.
This journal is © The Royal Society of Chemistry 2025

Supporting Information

Mechanochemical Nitration of Arenes and Alcohols Using a Bench-Stable Organic Nitrating Reagent

Vasiliki Valsamidou, Subrata Patra, Besa Kadriu, Michel Gaspard Metzger, Ludovic Gremaud and Dmitry Katayev*

Correspondence to: dmitry.katayev@unibe.ch

Contribution from:

Department of Chemistry, Biochemistry and Pharmaceutical Sciences, University of Bern, Freiestrasse 3, 3012 Bern, Switzerland

TABLE OF CONTENTS

1. General Information 3

2. Development of the Reaction Conditions for the Nitration of Alcohols 4

3. Development of the Reaction Conditions for the Nitration of Arenes 5

4. Green Metrics Calculations 6

5. Availability of Starting Materials 8

5.1. Commercially available starting materials 8

5.2. Synthesis of starting materials 10

6. General Procedures for the Nitration of Alcohols 13

7. General Procedure for the Nitration of Arenes 13

8. Scale-Up Synthesis 14

9. NMR Data 15

A. Alcohol Nitration 15

B. Aromatic Nitration 23

C. Post-Functionalizations of Nitrate Esters 35

10. NMR Spectra of Isolated Compounds 38

References 155

1. General Information

1.1. Material and methods

Starting materials were commercially available from Thermoscientific – Acros, Sigma Aldrich, Apollo Scientific, Fluorochem, and TCI, unless otherwise specified. All ball milling reactions were performed using a Mixer Mill (MM 400 and MM 500 Vario, Retsch Gmbh, Hann, Germany) equipped with 10 mL stainless steel grinding vessels and stainless steel balls, unless otherwise specified. Reaction progress was monitored by analytical Thin Layer Chromatography (TLC) on Merck silica gel 60 F254 TLC glass plates and visualized with 254 nm light and potassium permanganate or phosphomolybdic acid staining solutions followed by heating for detection. Reaction product purification was performed by flash chromatography using Brunschwig silica 32-63, 60Å under 0.3-0.5 bar overpressure. Medium pressure liquid chromatography (MPLC) was carried out on a CombiFlash Rf200 System from Teledyne ISCO with a built-in UV-detector and fraction collector, or manually using silica gel SilicaFlash P60, 40-63 µm. Teledyne ISCO RediSep Rf flash columns had particle sizes of 0.035–0.070 mm and 230–400 mesh. Normal phase preparatory HPLC purification was conducted on a Teledyne Isco CombiFlash EZ Prep system using a Macherey-Nagel VP 250/21 Nucleosil 50-5 column.

^{1}H- and ^{13}C-NMR spectra were recorded on a Bruker Ultrashield 300 (operating at 300.0 MHz and 75.0 MHz, respectively). Chemical shifts are reported in parts per million (ppm) and coupling constants (J) in Hertz (Hz). ^{1}H-NMR spectra are referenced to the solvent resonance unless noted otherwise ($CDCl_3$ at 7.26 ppm). Peaks are designated as (s = singlet, d = doublet, t = triplet, q = quartet, m = multiplet or unresolved) with coupling constant(s) in Hz and integration. ^{13}C-NMR spectra were recorded with ^{1}H-decoupling and referenced to the solvent resonance unless noted otherwise ($CDCl_3$ at 77.16 ppm). ^{19}F-NMR spectra were recorded with ^{1}H-decoupling unless noted otherwise. HR-MS (ESI+/EI+/NSI+) mass spectra were measured on a Bruker FTMS 4.7T BioAPEX II and Thermo Scientific LTQ Orbitrap XL equipped with a static nanospray ion source. Mass spectrometry service was operated on VG-TRIBRID for electron impact ionization (EI) or Varian IonSpec Spectrometer for electrospray ionization (ESI), and mass spectra are reported as (m/z). Electron impact ionization mass spectra (EI-MS) were obtained using an Agilent 8890 series GC system and Aglient 5977B GC/MSD.

2. Development of the Reaction Conditions for the Nitration of Alcohols

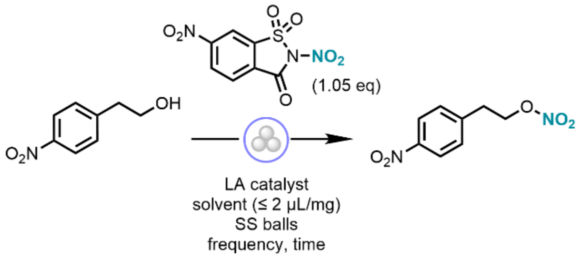

Without any precautions to exclude air or moisture, a Retsch stainless steel vessel (10 mL) with steel balls was charged with nitrating reagent, a Lewis acid catalyst, alcohol substrate, and solvent. The reaction vessel cap was locked and placed in the mixer mill. After the reaction was completed, a precise amount of dibromomethane was added as an internal standard, and the ^{1}H-NMR yield was calculated.

Entry[a]	***Variation from standard conditions***	***Yield (%)***[b]
1	none	90
2	*N*-nitrosaccharin	0
3	*N*-Nitrosuccinimide	0
4	*No catalyst*	35
5	*No solvent*	16
6	HFIP *(1 µL/mg)*	73
8	MeOH (2 µL/mg)	62
9	MeCN (2 µL/mg)	64
10	Cyclohexane (2 µL/mg)	0
11	$Mg(OTf)_2$	48
12	NaOTf	58
13	$Ca(OTf)_2$	56
14	$Fe(OTf)_3$	7
15	$Cu(OTf)_2$	74
16	$Zn(OTf)_2$	24
17	2 h	82
18	1 h	51
19	20 Hz	45
20	30 Hz	88
21	5 mol% catalyst loading	59
22	5 mL vessel, 3 x 7 mm balls	47
23	*1 ball*	14
24	*2 balls*	63
25	Ground by hand	32
26	PTFE-coated jar and balls	71

Table S1. a. Optimal reaction conditions: 4-*nitro-phenethyl alcohol* (0.3 mmol, 1.0 equiv), nitrating reagent (1.05 equiv), $Sc(OTf)_3$ (0.1 equiv), 25 Hz, 3 stainless steel balls (12 mm), 3 h. **b.** A yield of **4** was determined by ^{1}H-NMR against dibromomethane.

3. Development of the Reaction Conditions for the Nitration of Arenes

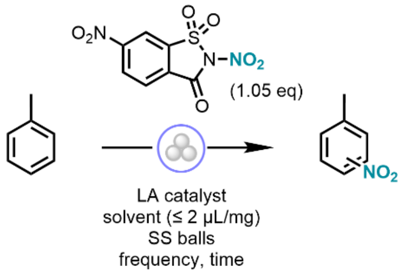

Without any precautions to exclude air or moisture, a Retsch stainless steel vessel (10 mL) with steel balls was charged with nitrating reagent, a Lewis acid catalyst, arene substrate, and solvent. The reaction vessel cap was locked and placed in the mixer mill. After the reaction was completed, an exact amount of dibromomethane was added as an internal standard, and the ^{1}H-NMR yield was calculated.

Entry[a]	***Variation from standard conditions***	***Yield (%)***[b]
1	none	87
2	*N*-nitrosaccharin	0
3	*N*-Nitrosuccinimide	0
4	*No catalyst*	4
5	*No solvent*	13
8	MeOH (2 µL/mg)	55
9	MeCN (2 µL/mg)	60
10	Cyclohexane (2 µL/mg)	7
12	NaOTf	27
13	$Ca(OTf)_2$	9
14	$Mg(OTf)_2$	14
15	$Fe(OTf)_3$	16
16	$Cu(OTf)_2$	12
17	$Zn(OTf)_2$	42
18	2 h	72
19	1 h	59
21	30 Hz	85
23	5 mol% catalyst loading	44
26	*2 balls*	67
28	PTFE-coated jar and balls	60

Table S2. a. Optimal reaction conditions: toluene (0.3 mmol, 1.0 equiv), nitrating reagent (1.05 equiv), $AgNTf_2$ (0.1 equiv), 25 Hz, 3 stainless steel balls (12 mm), 3 h. **b.** Yield of **30** was determined by ^{1}H-NMR against dibromomethane.

4. Green Metrics Calculations

Calculated Values:

	RME [%]	Mol. E [%]	E-factor
Ball Milling	90	22	3
Solution A	92	2	9.2
Solution B	82	11	4.4
Solution C	21	3	12.2

The calculations were based on the following equations:[1]

Reaction Mass Efficiency (RME) = $\frac{mass\ of\ product}{mass\ of\ reactants} \times 100\%$

Molar Efficiency (Mol. E) = $\frac{moles\ of\ product}{(moles\ reactants + moles\ catalyst + moles\ solvent + moles\ additives)} \times 100\%$

E-factor = $\frac{mass\ of\ waste}{mass\ of\ product}$

Ball Milling Reaction (this work):

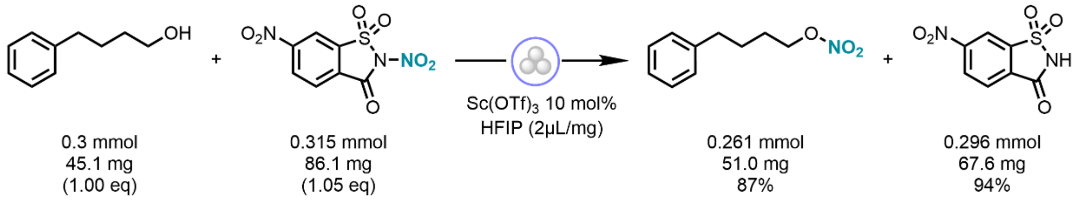

(6-Nitrosaccharin is recycled and considered a product)

RME = $\frac{51.0\ mg + 67.6\ mg}{45.1\ mg + 86.1\ mg}$ x 100% = 90%

Mol. E. = $\frac{(0.261\ mmol + 0.296\ mmol)}{(0.3\ mmol + 0.315\ mmol + 0.03\ mmol + 1.92\ mmol)}$ x 100% = 21.7%

E-factor = $\frac{(45.1\ mg + 86.1\ mg + 322.4\ mg + 14.8\ mg - 51.0\ mg - 67.6\ mg)}{(51.0\ mg + 67.6\ mg)}$ = 2.95

Solution Reaction A:[2]

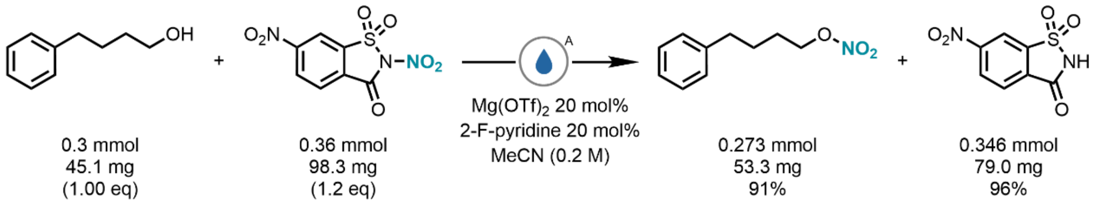

RME = $\frac{53.3\ mg + 79.0\ mg}{45.1\ mg + 98.3\ mg}$ x 100% = 92%

Mol. E. = $\frac{(0.273\ mmol + 0.346\ mmol)}{(0.3\ mmol + 0.36\ mmol + 0.06\ mmol + 0.06\ mmol + 28.6\ mmol)}$ x 100% = 2.1%

E-factor = $\frac{(45.1\ mg + 98.3\ mg + 19.3\ mg + 5.8\ mg + 1174.5\ mg - 53.3\ mg - 79.0\ mg)}{(53.3\ mg + 79.0\ mg)}$ = 9.2

Solution Reaction B:[3]

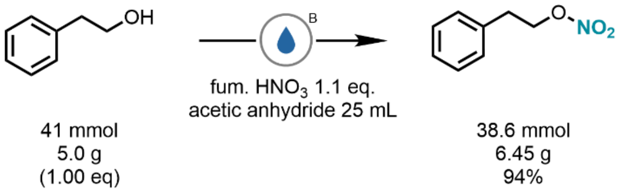

RME = $\frac{6.45\ g}{5.0\ g+2.84\ g}$ x 100% = 82%

Mol. E. = $\frac{(38.8\ mmol)}{(41\ mmol+41.5\ mmol+264.5\ mmol)}$ x 100% = 11.2%

E-factor = $\frac{(5\ g+2.84+27\ g-6.45\ g)}{(6.45\ g)}$ = 4.4

Solution Reaction C:[4]

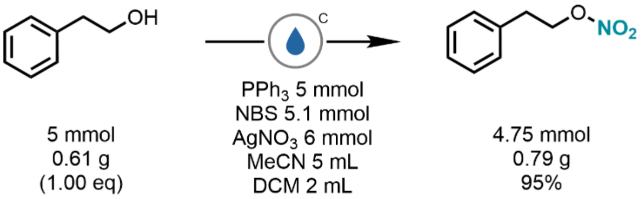

RME = $\frac{0.79\ g}{(0.61\ g+1.311\ g+0.91\ g+1.02\ g)}$ x 100% = 20.5%

Mol. E. = $\frac{(4.75\ mmol)}{(5\ mmol+5\ mmol+5.1\ mmol+6\ mmol+95.7\ mmol+31.3\ mmol)}$ x 100% = 3.2%

E-factor = $\frac{(0.61\ g+1.311\ g+0.91\ g+1.02\ g+3.93\ g+2.66\ g-0.79\ g)}{(0.79\ g)}$ = 12.2

5. AVAILABILITY OF STARTING MATERIALS

5.1. Commercially available starting materials

The commercially available starting materials were primarily purchased from Thermo Scientific–Acros, Sigma-Aldrich, Apollo Scientific, Fluorochem, and TCI. Upon receiving, the molecules were used without additional purification.

Alcohol-derived molecules:

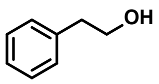

CAS
60-12-8

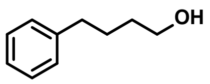

CAS
3360-41-6

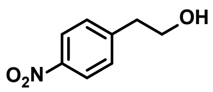

CAS
100-27-6

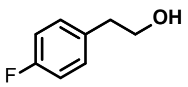

CAS
7589-27-7

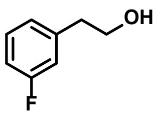

CAS
52059-53-7

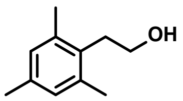

CAS
6950-92-1

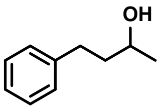

CAS
2344-70-9

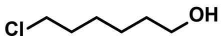

CAS
2009-83-8

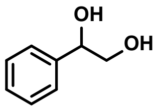

CAS
93-56-1

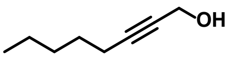

CAS
20739-58-6

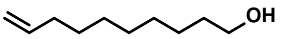

CAS
13019-22-2

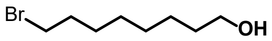

CAS
50816-19-8

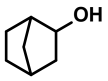

CAS
497-37-0

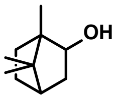

CAS
464-45-9

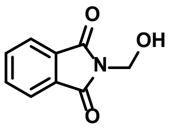

CAS
118-29-6

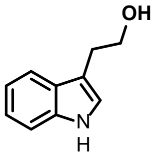

CAS
526-55-6

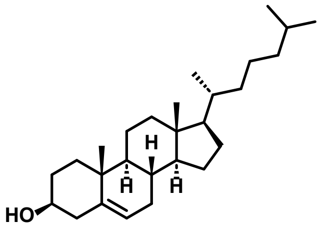

CAS
57-88-5

Aromatic compounds:

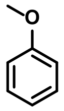

CAS
100-66-3

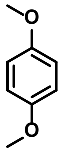

CAS
150-78-7

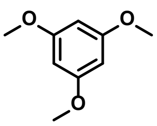

CAS
621-23-8

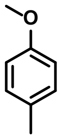

CAS
104-93-8

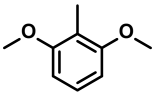

CAS
5673-07-4

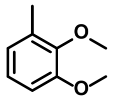

CAS
4463-33-6

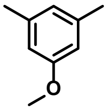

CAS
874-63-5

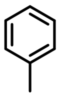

CAS
108-88-3

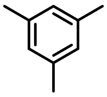

CAS
108-67-8

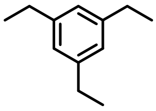

CAS
102-25-0

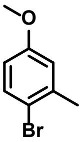

CAS
27060-75-9

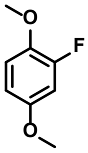

CAS
82830-49-7

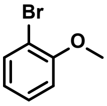

CAS
578-57-4

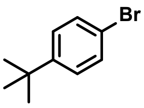

CAS
3972-65-4

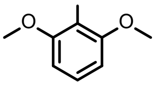

CAS
16932-44-8

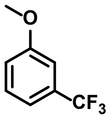

CAS
454-90-0

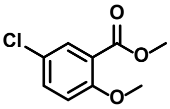

CAS
33924-48-0

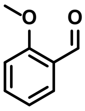

CAS
135-02-4

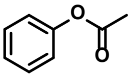

CAS
122-79-2

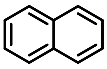

CAS
91-20-3

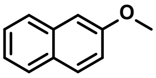

CAS
93-04-9

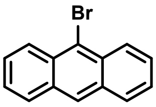

CAS
1564-64-3

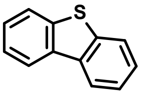

CAS
132-65-0

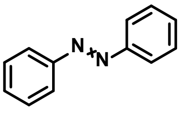

CAS
1227476-15-4

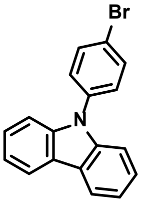

CAS
57102-42-8

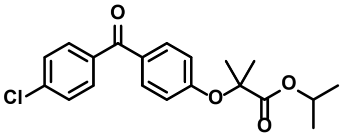

CAS
49562-28-9

5.2. Synthesis of starting materials

4-(4-Methoxyphenyl)butan-1-ol

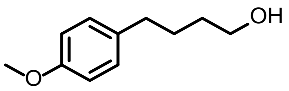

The titled compound was prepared according to the reported procedure.[5]

^{1}H-NMR (300 MHz, $CDCl_3$) δ 7.14 – 7.05 (m, 2H), 6.86 – 6.79 (m, 2H), 3.79 (s, 3H), 3.65 (t, *J* = 6.2 Hz, 2H), 2.59 (t, *J* = 7.2 Hz, 2H), 1.76 – 1.52 (m, 4H).

2-Azido-2-phenylethan-1-ol

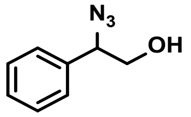

The titled compound was prepared according to the reported procedure.[6]

^{1}H-NMR (300 MHz, $CDCl_3$) δ 7.45 – 7.30 (m, 5H), 4.68 (dd, *J* = 7.1, 5.8 Hz, 1H), 3.78 – 3.72 (m, 2H).

2-Nitro-2-phenylethan-1-ol

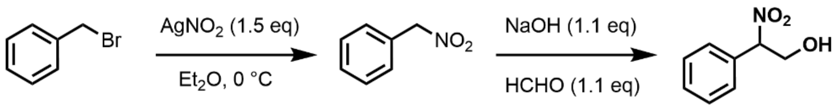

Silver nitrite (4.6 g, 1.5 eq.) was added to a round-bottom flask wrapped in aluminum foil, containing anhydrous diethyl ether (52 mL). The mixture was stirred at room temperature for 15 minutes, then cooled to 0 °C. A solution of benzyl bromide (20 mmol, 1.0 eq.) in diethyl ether (3.4 mL) was added dropwise via an addition funnel. The reaction mixture was stirred at 0 °C for 1 hour, followed by refluxing for 4 hours. After completion, the mixture was filtered through Celite using ethyl acetate as the eluent. The crude product was then purified by column chromatography on silica gel, eluting with a 90:10 mixture of hexane and ethyl acetate.

^{1}H-NMR (300 MHz, $CDCl_3$): δ 7.51 – 7.40 (m, 5H), 5.44 (s, 2H).

^{13}C-NMR (75 MHz, $CDCl_3$): δ 130.1, 130.1, 129.8, 129.2, 80.1.

A pre-cooled (0 °C) aqueous solution of sodium hydroxide (0.55 g, 1.1 eq.) in water (50 mL) was added slowly to neat nitroethane derivatives (1.0 eq.) at 23 °C. The mixture was stirred at 23 °C for 2 hours, then cooled to 0 °C, followed by dropwise addition of an aqueous formaldehyde solution (37 wt %, 1.1 eq.). After stirring at 0 °C for 30 minutes, the reaction was allowed to warm to 23 °C and stirred for an additional hour. The mixture was cooled again to 0 °C, and acetic acid (1.2 eq.) was added. The solution was warmed to 23 °C and extracted with ethyl acetate (3 × 100 mL). The combined organic layers were dried over anhydrous magnesium sulfate, filtered, and concentrated under reduced pressure. The crude

product was purified by flash column chromatography to yield the desired 2-nitro-2-phenylethan-1-ol as white solid.

^{1}H-NMR (300 MHz, $CDCl_3$): δ 7.41 (s, 5H), 5.64 (ddd, *J* = 9.9, 3.6, 1.5 Hz, 1H), 4.57 (ddd, *J* = 12.6, 9.9, 1.1 Hz, 1H), 3.95 (ddd, *J* = 12.7, 3.7, 1.3 Hz, 1H), 2.86 (s, 1H).

^{13}C-NMR (75 MHz, $CDCl_3$): δ 131.4, 130.3, 129.3, 127.7, 92.5, 63.8.

2-Bromo-2-phenylethan-1-ol

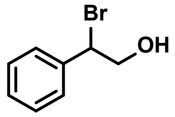

The titled compound was prepared according to reported procedure.[7]

^{1}H-NMR (300 MHz, $CDCl_3$) δ 7.45 – 7.32 (m, 5H), 5.07 (dd, *J* = 7.9, 5.7 Hz, 1H), 4.12 – 3.92 (m, 2H).

Estrone 3-methyl ether

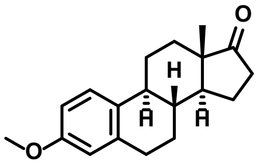

In a reaction flask, 2.0 g (7.5 mmol, 1.0 equiv) of estrone is measured out, and 200 mL of acetone is introduced as the solvent. Then, 5.2 g (37.5 mmol, 5.0 equiv) of potassium carbonate is incorporated, and the mixture is stirred at room temperature. After 10 minutes of stirring, 4.67 mL (75.0 mmol, 10.0 equiv) of methyl iodide is added, and stirring is maintained for 16 hours. The reaction progress is tracked with TLC until no estrone remains visible. Upon completion, the solvent is evaporated under reduced pressure. The remaining residue is extracted with ethyl acetate. The resulting organic layer is washed twice—first with water, then with saturated brine—and dried using anhydrous Na_2SO_4. The solvent is removed under reduced pressure, and the crude product is purified via column chromatography with a 0–50% ethyl acetate : hexane mixture employed as the eluent. The target white solid product is yielded at an 88% yield.

^{1}H-NMR (300 MHz, $CDCl_3$): δ 7.21 (d, *J* = 8.6 Hz, 1H), 6.72 (dd, *J* = 8.6, 2.9 Hz, 1H), 6.65 (d, *J* = 2.8 Hz, 1H), 3.78 (s, 3H), 2.90 (dd, *J* = 9.7, 5.0 Hz, 2H), 2.57 – 1.90 (m, 7H), 1.70 – 1.35 (m, 7H), 0.91 (s, 3H).

^{13}C-NMR (75 MHz, $CDCl_3$): δ 221.1, 137.9, 132.2, 126.5, 114.0, 111.7, 55.4, 50.6, 48.2, 44.1, 38.52, 36.0, 31.7, 29.8, 26.7, 26.1, 21.7, 14.0.

The spectra are consistent with the literature.[8]

(1R,2R,8aS)-1-(2-Hydroxyethyl)-2,5,5,8a-tetramethyldecahydronaphthalen-2-ol

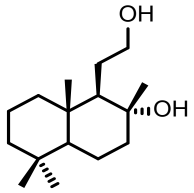

The titled compound was prepared according to reported procedure.[9]

^{1}H-NMR (300 MHz, $CDCl_3$): δ 3.81–3.76 (m, 3H), 3.46 (q, J = 7.7 Hz, 1H), 1.94 – 1.84 (m, 1H), 1.57–1.08 (m, 14H), 0.98 – 0.92 (m, 2H), 0.87 (s, 3H), 0.78 (s, 6H).

6. General Procedures for the Nitration of Alcohols

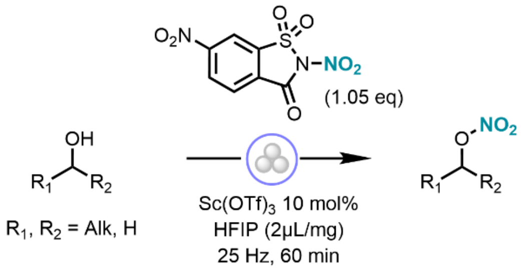

GP1: A mixture of alcohol (0.30 mmol, 1.0 equiv), $Sc(OTf)_3$ (0.03 mmol, 0.1 equiv), saccharin-derived reagent **NN** (0.315 mmol, 1.05 eq), and HFIP (2 µL/mg of solids) was placed in a stainless-steel vessel (10 mL) with a 3 stainless-steel ball (12 mm). Next, the ball milling vessel was placed in the mixer mill (Retsch MM400 or Retsch MM500 vario) for 3 h with a frequency of 25 Hz. After the reaction was completed, the contents of the vessel were transferred with EtOAc into a round-bottom flask and purified directly by column chromatography on silica gel using EtOAc/n-hexane.

7. General Procedure for the Nitration of Arenes

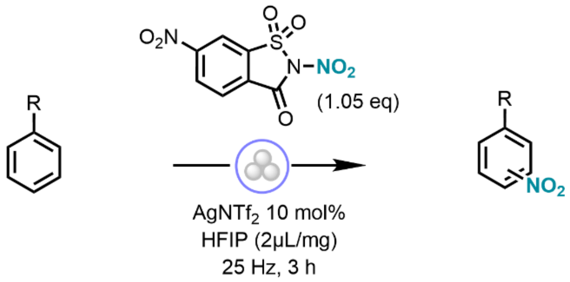

GP2: A mixture of arene (0.30 mmol, 1.0 equiv), $AgNTf_2$ (0.03 mmol, 0.1 equiv), saccharin derived reagent **NN** (0.315 mmol, 1.05 eq) and HFIP (2 µL/mg of solids) was placed in a stainless-steel vessel (10 mL) with a 3 stainless-steel ball (12 mm). Next, the ball milling vessel was placed in the mixer mill (Retsch MM400 or Retsch MM500 vario) for 3 h with a frequency of 25 Hz. After the reaction was completed, the contents of the vessel were transferred with EtOAc into a round-bottom flask and purified directly by column chromatography on silica gel using EtOAc/n-hexane.

8. Scale-Up Synthesis

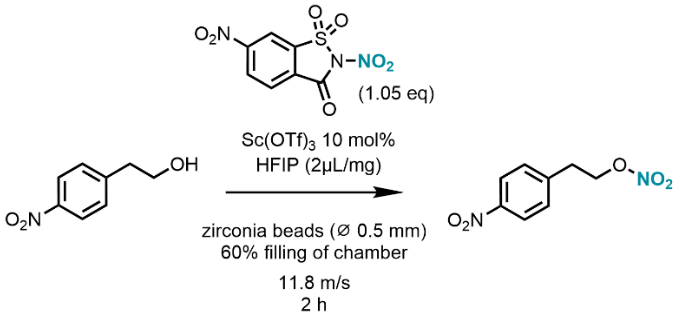

A mixture of alcohol (3.0 g, 18 mmol, 1.0 equiv), $Sc(OTf)_3$ (1.8 mmol, 0.1 equiv), saccharin-derived reagent NN (18.9 mmol, 1.05 eq) and HFIP (2 µL/mg of solids) was placed in a bead-mill chamber (80 mL) of a WAB DYNO®-MILL RESEARCH LAB with a 60 vol% fill of zirconia beads (0.5 mm, 177.6 g). The chamber was closed and operated for 2 h at 11.8 m/s. After the reaction was completed, the chamber was washed with EtOAc (3 × 100 mL), the solvent was evaporated, and the crude was purified by column chromatography on silica gel using EtOAc/n-hexane.

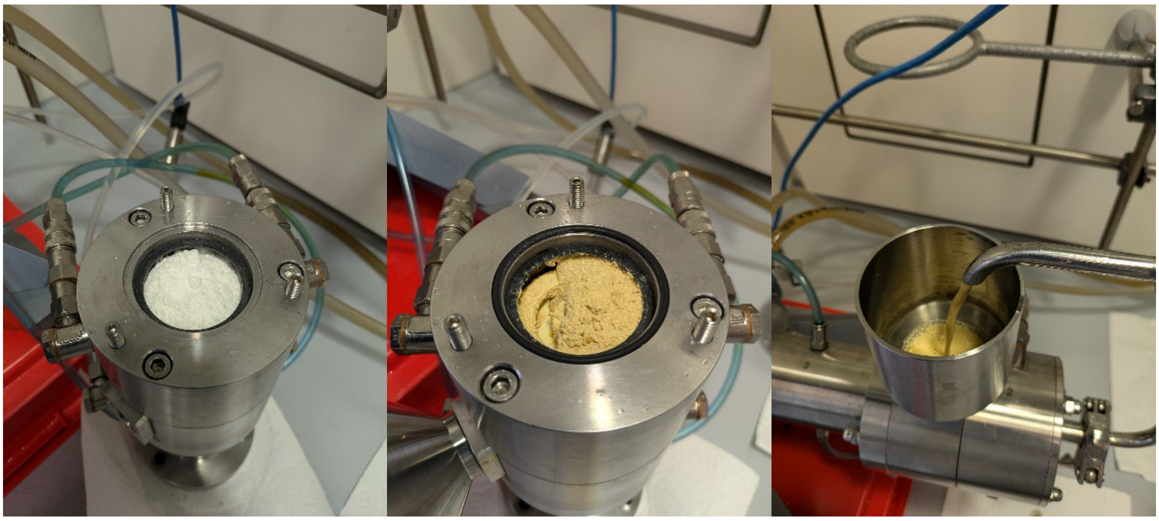

Figure 1. *Left:* Filled bead mill chamber prior to reaction. *Center:* Filled bead mill chamber after the reaction. *Right:* Process of rinsing the bead mill chamber with solvent to collect crude product.

9. NMR DATA

A. Alcohol Nitration

4-Nitrophenethyl nitrate (1)

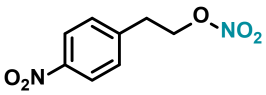

Compound **1** was obtained according to the general procedure **GP1** and **Scale-Up** from 2-(4-nitrophenyl)ethan-1-ol (0.3 mmol, 1.0 equiv). Isolated as an off-white solid (90% yield and 99% respectively).

^{1}H-NMR (300 MHz, $CDCl_3$) δ 8.24 – 8.16 (m, 2H), 7.45 – 7.37 (m, 2H), 4.70 (t, *J* = 6.7 Hz, 2H), 3.15 (t, *J* = 6.7 Hz, 2H).

^{13}C-NMR (75 MHz, $CDCl_3$) δ 147.4, 143.9, 129.9, 124.2, 72.2, 33.3.

FT-IR (ATR, neat; cm^{-1}): 3083, 2972, 1615, 1513, 1345, 1274, 1240, 857.

HRMS (EI): not found.

EA calcd for $C_8H_8N_2O_5$: C, 45.29; H, 3.80; N, 13.20. Found: C, 45.5; H, 3.8; N, 13.08.

Phenethyl nitrate (2)

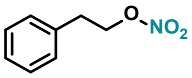

Compound **2** was obtained according to the general procedure **GP1** from 2-phenylethan-1-ol (0.3 mmol, 1.0 equiv). It was isolated as a colourless oil (65% yield).

^{1}H-NMR (300 MHz, $CDCl_3$) δ 7.40 – 7.28 (m, 3H), 7.24 (dd, *J* = 8.1, 1.8 Hz, 2H), 4.66 (t, *J* = 7.1 Hz, 2H), 3.04 (t, *J* = 7.1 Hz, 2H).

The spectra are consistent with the reported literature.[10]

4-Phenylbutyl nitrate (3)

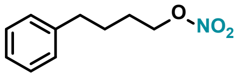

Compound **3** was obtained according to the general procedure **GP1** from 4-phenylbutan-1-ol (0.3 mmol, 1.0 equiv). It was isolated as a colourless oil (87% yield).

^{1}H-NMR (300 MHz, $CDCl_3$) δ 7.34 – 7.26 (m, 2H), 7.24 – 7.13 (m, 3H), 4.51 – 4.39 (m, 2H), 2.72 – 2.60 (m, 2H), 1.75 (p, *J* = 3.4 Hz, 4H).

^{13}C-NMR (75 MHz, $CDCl_3$) δ 141.5, 128.6, 128.5, 126.2, 73.3, 35.4, 27.5, 26.4.

FT-IR (ATR, neat; cm^{-1}): 3026, 2942, 1618, 1495, 1453, 1274, 858, 747, 697.

HRMS (EI): not found.

4-(4-Methoxyphenyl)butyl nitrate (4)

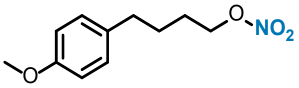

Compound **4** was obtained according to the general procedure **GP1** from 4-(4-methoxyphenyl)butan-1-ol (0.3 mmol, 1.0 equiv). Isolated as a colourless liquid (52% yield).

^{1}H-NMR (300 MHz, $CDCl_3$): δ 7.13 – 7.05 (m, 2H), 6.88 – 6.81 (m, 2H), 4.48 – 4.41 (m, 2H), 3.80 (s, 3H), 2.61 (t, J = 7.0 Hz, 2H), 1.79 – 1.63 (m, 4H).

The spectra are consistent with the literature.[2]

2,4,6-Trimethyl-3-nitrophenethyl nitrate (5)

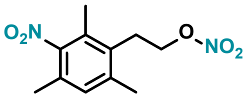

Compound **5** was obtained according to the general procedure **GP1** from 2-mesitylethan-1-ol (0.3 mmol, 1.0 equiv). It was isolated as a yellow oil (52% yield).

^{1}H-NMR (300 MHz, $CDCl_3$) δ 6.99 (s, 1H), 4.53 – 4.42 (m, 2H), 3.14 – 3.03 (m, 2H), 2.37 (s, 3H), 2.26 (s, 3H), 2.24 (s, 3H).

^{13}C-NMR (75 MHz, $CDCl_3$) δ 151.6, 139.3, 131.7, 131.0, 128.0, 127.9, 70.7, 27.5, 20.2, 17.2, 14.4.

HRMS (EI) *m/z,* calcd for $C_{11}H_{14}N_2O_5$: 254.0903; found 254.0901.

4-Fluorophenethyl nitrate (6)

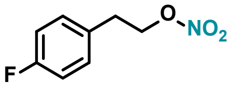

Compound **6** was obtained according to the general procedure **GP1** from 2-(4-fluorophenyl)ethan-1-ol (0.3 mmol, 1.0 equiv). It was isolated as a yellow oil (88% yield).

^{1}H-NMR (300 MHz, $CDCl_3$) δ 7.25 – 7.14 (m, 2H), 7.08 – 6.96 (m, 2H), 4.62 (t, J = 7.0 Hz, 2H), 3.00 (t, J = 7.0 Hz, 2H).

^{13}C-NMR (75 MHz, $CDCl_3$) δ 163.7, 160.5, 131.9, 131.9, 130.6, 130.5, 73.4, 32.7.

FT-IR (ATR, neat; cm^{-1}): 2919, 2850, 2359, 1616, 1511, 1464, 1343, 1276, 891, 856.

HRMS (ESI) *m/z,* calcd for $C_8H_8FNO_3$: 185.0488; found 185.0486.

3-Fluorophenethyl nitrate (7)

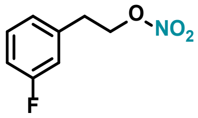

Compound **7** was obtained using **GP1** from 2-(3-fluorophenyl)ethan-1-ol (0.3 mmol, 1.0 equiv). Isolated as a yellow solid (83% yield).

^{1}H-NMR (300 MHz, $CDCl_3$) δ 7.30 (td, J = 8.6, 6.7 Hz, 1H), 7.08 – 6.88 (m, 3H), 4.64 (t, J = 6.9 Hz, 2H), 3.02 (t, J = 6.9 Hz, 2H).

^{13}C-NMR (75 MHz, $CDCl_3$) δ 164.7, 161.5, 138.8, 138.7, 130.5, 130.4, 124.7, 124.6, 116.1, 115.8, 114.4, 114.1, 72.9, 33.2, 33.2.

FT-IR (ATR, neat; cm^{-1}): 3733, 2360, 2341, 1624, 1276, 859, 669.

HRMS (EI) *m/z,* calcd for $C_8H_8FNO_3$: 185.0488; found 185.0485.

2-Nitro-2-phenylethyl nitrate (8)

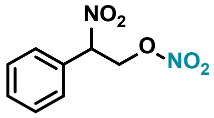

Compound **8** was obtained according to the general procedure **GP1** from 2-nitro-2-phenylethan-1-ol (0.3 mmol, 1.0 equiv). It was isolated as a yellow solid (92% yield).

^{1}H-NMR (300 MHz, $CDCl_3$) δ 7.47 (dtt, J = 5.1, 3.3, 1.3 Hz, 5H), 5.74 (dd, J = 10.3, 3.2 Hz, 1H), 5.42 (dd, J = 12.6, 10.3 Hz, 1H), 4.84 (dd, J = 12.6, 3.2 Hz, 1H).

^{13}C-NMR (75 MHz, $CDCl_3$) δ 131.1, 129.7, 129.7, 127.5, 86.7, 77.0, 70.9.

HRMS (EI) *m/z,* calcd for $C_8H_8N_2O_5$–HN_2O_4: 119.0493; found 119.0493.

2-Bromo-2-phenylethyl nitrate (9)

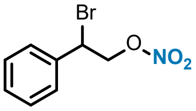

Compound **9** was obtained according to the general procedure **GP1** from 2-bromo-2-phenylethan-1-ol (0.3 mmol, 1.0 equiv). Isolated as a colourless liquid (31% yield).

^{1}H-NMR (300 MHz, $CDCl_3$): δ 7.40 (tdd, *J* = 7.7, 3.4, 2.2 Hz, 5H), 5.13 (t, *J* = 7.3 Hz, 1H), 4.97 (dd, *J* = 12.2, 7.1 Hz, 1H), 4.87 (dd, *J* = 12.2, 7.4 Hz, 1H).

^{13}C-NMR (75 MHz, $CDCl_3$): δ 136.9, 129.7, 129.3, 128.9, 128.7, 127.9, 74.8, 46.9.

HRMS (EI) *m/z,* calcd for $C_8H_8BrNO_3$–H_2NO_2: 196.9597; found 196.9600.

2-Azido-2-phenylethyl nitrate (10)

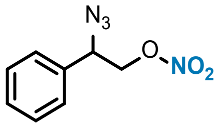

Compound **10** was obtained according to the general procedure **GP1** from 2-azido-2-phenylethan-1-ol (0.3 mmol, 1.0 equiv). It was isolated as a colourless liquid (43% yield).

^{1}H-NMR (300 MHz, $CDCl_3$): δ 7.49 – 7.40 (m, 3H), 7.36 (dd, *J* = 7.4, 2.2 Hz, 2H), 4.86 (dd, *J* = 8.0, 5.0 Hz, 1H), 4.63 – 4.52 (m, 2H).

^{13}C-NMR (75 MHz, $CDCl_3$): δ 134.7, 129.6, 129.4, 127.2, 74.4, 62.9.

HRMS (ESI) *m/z,* calcd for $C_8H_8N_4O_3$–$CH_2N_3O_3$: 104.0495; found 104.0497.

6-Chlorohexyl nitrate (11)

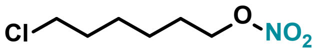

Compound **11** was obtained according to the general procedure **GP1** from 6-chlorohexan-1-ol (0.3 mmol, 1.0 equiv). It was isolated as a colourless oil (99% yield).

^{1}H-NMR (300 MHz, $CDCl_3$) δ 4.45 (t, *J* = 6.6 Hz, 2H), 3.54 (t, *J* = 6.6 Hz, 2H), 1.84 – 1.68 (m, 4H), 1.47 (qd, *J* = 6.5, 2.6 Hz, 4H).

^{13}C-NMR (75 MHz, $CDCl_3$) δ 73.3, 44.9, 32.4, 26.8, 26.5, 25.2.

FT-IR (ATR, neat; cm^{-1}): 2942, 1619, 1274, 858, 747, 697.

HRMS (EI): not found.

Oct-2-yn-1-yl nitrate (12)

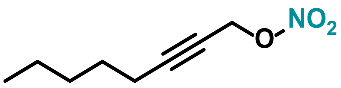

Compound **12** was obtained from oct-2-yn-1-ol (0.3 mmol, 1.0 equiv) according to the general procedure **GP1**. It was isolated as a colourless liquid (99% yield).

^{1}H-NMR (300 MHz, $CDCl_3$) δ 4.99 (t, *J* = 2.2 Hz, 2H), 2.23 (tt, *J* = 7.1, 2.2 Hz, 2H), 1.52 (ddt, *J* = 10.9, 9.2, 5.5 Hz, 2H), 1.41 – 1.24 (m, 4H), 0.97 – 0.82 (m, 3H).

^{13}C-NMR (75 MHz, $CDCl_3$) δ 90.8, 71.1, 61.4, 31.1, 28.0, 22.3, 18.8, 14.0.

HRMS (EI): not found.

Dec-9-en-1-yl nitrate (13)

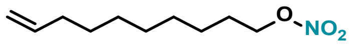

Compound **13** was obtained from dec-9-en-1-ol (0.3 mmol, 1.0 equiv) according to the general procedure **GP1**. It was isolated as a colourless oil (89% yield).

^{1}H-NMR (300 MHz, $CDCl_3$) δ 5.81 (ddt, J = 16.9, 10.2, 6.7 Hz, 1H), 5.04 – 4.89 (m, 2H), 4.44 (t, J = 6.7 Hz, 2H), 2.04 (tdd, J = 6.6, 5.3, 1.5 Hz, 2H), 1.70 (dt, J = 8.0, 6.5 Hz, 2H), 1.46 – 1.23 (m, 10H).

^{13}C-NMR (75 MHz, $CDCl_3$) δ 139.2, 114.4, 73.6, 33.9, 29.4, 29.2, 29.1, 29.0, 26.9, 25.8.

HRMS (EI) *m/z,* calcd for $C_{10}H_{19}NO_3$-HNO_2: 154.1358; found 154.1355.

Undec-10-en-1-yl nitrate (14)

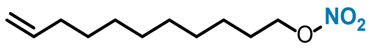

Compound **14** was obtained using the general procedure **GP1** from undec-10-en-1-ol (0.3 mmol, 1.0 equiv). Isolated as a colourless liquid (89% yield).

^{1}H-NMR (300 MHz, $CDCl_3$) δ 5.81 (ddt, J = 16.9, 10.1, 6.6 Hz, 1H), 5.05 – 4.88 (m, 2H), 4.44 (t, J = 6.7 Hz, 2H), 2.04 (tdd, J = 6.6, 5.3, 1.4 Hz, 2H), 1.71 (dq, J = 8.1, 6.6 Hz, 2H), 1.46 – 1.22 (m, 12H).

^{13}C-NMR (75 MHz, $CDCl_3$) δ 139.3, 114.3, 73.6, 33.9, 29.5, 29.2, 29.2, 29.0, 26.9, 25.8.

HRMS (ESI) *m/z,* calcd for $C_{11}H_{21}NO_3$–NO_2: 169.1587; found 169.1590.

4-Phenylbutan-2-yl nitrate (15)

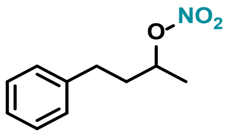

Compound **15** was obtained from 4-phenylbutan-2-ol (0.3 mmol, 1.0 equiv) according to the general procedure **GP1**. It was isolated as a colourless oil (78% yield).

^{1}H-NMR (300 MHz, $CDCl_3$) δ 7.36 – 7.13 (m, 5H), 5.07 (ddt, J = 12.1, 7.5, 6.1 Hz, 1H), 2.84 – 2.62 (m, 2H), 2.05 (dddd, J = 13.7, 9.2, 7.6, 6.1 Hz, 1H), 1.90 (dddd, J = 14.4, 9.5, 6.9, 5.3 Hz, 1H), 1.39 (d, J = 6.2 Hz, 3H).

^{13}C-NMR (75 MHz, $CDCl_3$) δ 140.6, 128.8, 128.4, 126.4, 80.6, 35.8, 31.5, 18.7.

The spectra are consistent with the literature data.[2]

Bicyclo[2.2.1]heptan-2-yl nitrate (16)

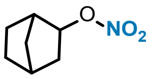

Compound **16** was obtained as a colourless oil (77% yield) according to the general procedure **GP1** after purification using column chromatography (SiO_2, hexane/EA=20:1).

^{1}H-NMR (300 MHz, $CDCl_3$): δ 4.81 (dt, *J* = 7.2, 2.1 Hz, 1H), 2.46 (d, *J* = 5.0 Hz, 1H), 2.35 (t, *J* = 1.3 Hz, 1H), 1.79 (d, *J* = 2.6 Hz, 1H), 1.62 – 1.47 (m, 4H), 1.23 – 1.12 (m, 3H).

^{13}C-NMR (75 MHz, $CDCl_3$): δ 87.0, 40.4, 37.9, 35.4, 35.3, 28.0, 24.3.

The spectra are consistent with the literature.[2]

1,7,7-Trimethylbicyclo[2.2.1]heptan-2-yl nitrate (17)

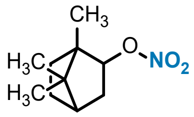

Compound **17** was obtained as a colourless oil (80% yield) after purification by column chromatography (SiO2, hexane/EA=20:1) according to general procedure **GP1**.

^{1}H-NMR (300 MHz, $CDCl_3$): δ 5.03 (ddd, *J* = 9.6, 3.5, 2.0 Hz, 1H), 2.45 (dddd, *J* = 14.1, 9.6, 4.5, 3.5 Hz, 1H), 1.94 – 1.70 (m, 3H), 1.43 – 1.13 (m, 3H), 0.94 (d, *J* = 1.6 Hz, 6H), 0.89 (s, 3H).

^{13}C-NMR (75 MHz, $CDCl_3$): δ 89.4, 49.1, 48.5, 44.7, 36.2, 27.9, 26.9, 19.5, 18.9, 13.7.

The spectra are consistent with the literature data.[2]

(1,3-Dioxoisoindolin-2-yl)methyl nitrate (18)

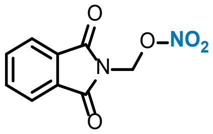

Compound **18** was obtained according to the general procedure **GP1** as a yellow solid (88% yield) after purification by column chromatography (SiO_2, hexane/EA=20:1).

^{1}H-NMR (300 MHz, $CDCl_3$): δ 4.37 (t, *J* = 7.0 Hz, 2H), 3.40 (t, *J* = 6.8 Hz, 2H), 2.00 (p, *J* = 7.1 Hz, 2H), 1.89 – 1.75 (m, 2H), 1.47 – 1.29 (m, 8H).

^{13}C-NMR (75 MHz, $CDCl_3$): δ 166.1, 135.2, 131.5, 124.4, 66.8.

The spectra are consistent with the literature.[2]

2-(1H-Indol-3-yl)ethyl nitrate (19)

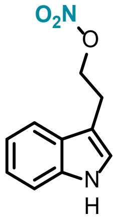

Compound **19** was obtained from tryptophol (0.3 mmol, 1.0 equiv) according to the general procedure **GP1**. It was isolated as a yellow liquid (43% yield).

^{1}H-NMR (300 MHz, $CDCl_3$): δ 8.08 (s, 1H), 7.61 (d, *J* = 7.8 Hz, 1H), 7.39 (d, *J* = 8.1 Hz, 1H), 7.24 – 7.05 (m, 3H), 4.71 (t, *J* = 7.1 Hz, 2H), 3.20 (t, *J* = 7.2 Hz, 2H).

^{13}C-NMR (75 MHz, $CDCl_3$): δ 136.4, 127.2, 122.7, 122.6, 119.9, 118.5, 111.5, 110.4, 73.1, 23.3.

HRMS (EI) *m/z,* calcd for $C_{10}H_{10}N2O_3$–HNO_2: 159.0679; found 159.0681.

**2-((*1R,2R,8aS*)-2-Hydroxy-2,5,5,8a-tetramethyldecahydronaphthalen-1-yl)-
ethyl nitrate (20)**

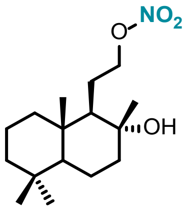

Compound **20** was obtained according to the general procedure **GP1** from 1-phenylethane-1,2-diol (0.3 mmol, 1.0 equiv). It was isolated as a colourless liquid (29% yield).

^{1}H-NMR (300 MHz, $CDCl_3$): δ 3.98 – 3.75 (m, 2H), 1.94 (dt, *J* = 10.6, 2.7 Hz, 1H), 1.78 – 1.71 (m, 2H), 1.45 – 1.35 (m, 5H), 1.33 – 1.20 (m, 5H), 1.08 (d, *J* = 0.9 Hz, 3H), 1.05 – 0.92 (m, 3H), 0.87 (s, 3H), 0.85 – 0.81 (m, 6H).

^{13}C-NMR (75 MHz, $CDCl_3$): δ 80.1, 65.1, 60.3, 57.4, 42.6, 40.1, 39.9, 36.4, 33.7, 33.2, 29.8, 22.8, 21.3, 20.8, 18.6, 15.2.

HRMS (EI) *m/z,* calcd for $C_{16}H_{29}NO_4$–HNO_3: 236.2135; found 236.2139.

(*3S,8S,9S,10R,13R,14S,17R*)-10,13-Dimethyl-17-((*R*)-6-methylheptan-2-yl)-2,3,4,7,8,9,10,11,12,13,14,15,16,17-tetradecahydro-1H-cyclopenta[a]phenanthren-3-yl nitrate (21)

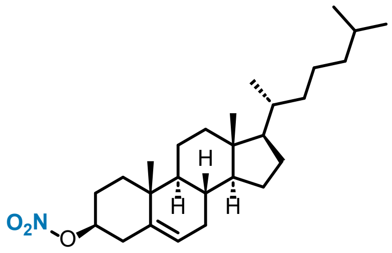

Compound **21** was obtained as a white solid (77% yield) after purification by column chromatography (SiO_2, hexane/EA=20:1) according to general procedure **GP1**.

^{1}H-NMR (300 MHz, $CDCl_3$): δ 5.47 – 5.40 (m, 1H), 4.80 (tdd, *J* = 11.3, 5.4, 4.1 Hz, 1H), 2.50 – 2.32 (m, 2H), 2.08 – 0.96 (m, 31H), 0.92 (d, *J* = 6.5 Hz, 3H), 0.87 (dd, *J* = 6.6, 1.3 Hz, 6H), 0.68 (s, 3H).

^{13}C-NMR (75 MHz, $CDCl_3$): δ 138.5, 124.1, 83.5, 56.8, 56.3, 50.1, 42.5, 39.8, 39.7, 37.0, 36.8, 36.4, 36.3, 35.9, 32.1, 32.0, 28.4, 28.2, 26.1, 24.4, 24.0, 23.0, 22.7, 21.2, 19.4, 18.9, 12.0.

The spectra are consistent with the literature.[2]

2-Hydroxy-2-phenylethyl nitrate (22a)

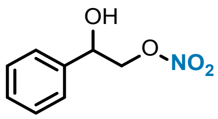

Compound **22a** was obtained according to the general procedure **GP1** from 1-phenylethane-1,2-diol (0.3 mmol, 1.0 equiv). Isolated as a colourless liquid (58% yield).

^{1}H-NMR (300 MHz, $CDCl_3$): δ 7.47 – 7.38 (m, 5H), 5.08 (dd, *J* = 8.2, 3.6 Hz, 1H), 4.66 – 4.52 (m, 2H), 2.38 (s, 1H).

The spectra are consistent with the literature.[2]

1-Phenylethane-1,2-diyl dinitrate (22b)

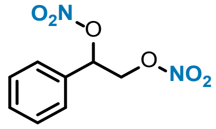

Compound **22b** was obtained according to a variation of the general procedure **GP1,** in which 2.0 eq of the saccharin-derived reagent **NN** were used**,** from 1-phenylethane-1,2-diol (0.3 mmol, 1.0 equiv). Isolated as a light-yellow liquid (20% yield).

^{1}H-NMR (300 MHz, $CDCl_3$): δ 7.47 – 7.39 (m, 5H), 6.12 (dd, J = 7.2, 5.0 Hz, 1H), 4.79 – 4.70 (m, 2H).

^{13}C-NMR (75 MHz, $CDCl_3$): δ 132.9, 130.3, 129.5, 126.8, 80.7, 71.4.

The spectra are consistent with the literature.[11]

B. Aromatic Nitration

1-Methoxy-4-nitrobenzene (23a)

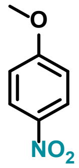

Compound **23a** was obtained according to the general procedure **GP2** from anisole (0.3 mmol, 1.0 equiv). Isolated as a white solid (42% yield).

^{1}H-NMR (300 MHz, $CDCl_3$) δ 8.25 – 8.17 (m, 2H), 7.00 – 6.92 (m, 2H), 3.91 (s, 3H).

^{13}C-NMR (75 MHz, $CDCl_3$): δ 164.7, 126.1, 114.2, 56.1.

The spectra are consistent with the literature.[12]

1-Methoxy-2-nitrobenzene (23b)

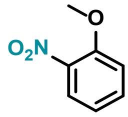

Compound **23b** was obtained according to the general procedure **GP2** from anisole (0.3 mmol, 1.0 equiv). Isolated as a colourless liquid (56% yield).

^{1}H-NMR (300 MHz, $CDCl_3$) δ 7.84 (dd, J = 8.1, 1.7 Hz, 1H), 7.54 (ddd, J = 8.4, 7.4, 1.7 Hz, 1H), 7.09 (dd, J = 8.5, 1.2 Hz, 1H), 7.03 (ddd, J = 8.4, 7.5, 1.2 Hz, 1H), 3.96 (s, 3H).

^{13}C-NMR (75 MHz, $CDCl_3$): δ 153.1, 134.3, 125.8, 120.4, 113.6, 56.6.

The spectra are consistent with the literature.[12]

1,4-Dimethoxy-2-nitrobenzene (24)

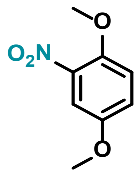

Compound **24** was obtained from 1,4-dimethoxybenzene (0.3 mmol, 1.0 equiv) according to the general procedure **GP2**. It was isolated as a yellow liquid (99% yield).

^{1}H-NMR (300 MHz, $CDCl_3$) δ 7.39 (d, J = 3.0 Hz, 1H), 7.11 (dd, J = 9.2, 3.1 Hz, 1H), 7.02 (d, J = 9.2 Hz, 1H), 3.91 (s, 3H), 3.81 (s, 3H).

^{13}C-NMR (75 MHz, $CDCl_3$) δ 153.0, 147.5, 121.1, 115.3, 110.1, 57.3, 56.2.

The spectra are consistent with the literature.[13]

1,3,5-Trimethoxy-2-nitrobenzene (25)

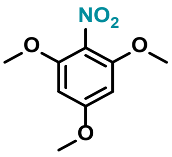

Compound **25** was obtained using the general procedure **GP2** from 1,3,5-trimethoxybenzene (0.3 mmol, 1.0 equiv). It was isolated as a yellow oil (99% yield).

^{1}H-NMR (300 MHz, $CDCl_3$) δ 6.12 (s, 2H), 3.85 (s, 6H), 3.83 (s, 3H).

^{13}C-NMR (75 MHz, $CDCl_3$) δ 162.4, 153.5, 90.9, 56.6, 55.9.

The spectra are consistent with the literature.[14]

1-Methoxy-4-methyl-2-nitrobenzene (26)

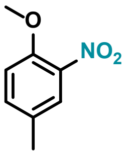

Compound **26** was obtained from 1-methoxy-4-methylbenzene (0.3 mmol, 1.0 equiv) according to the general procedure **GP2**. It was isolated as a yellow oil (97% yield).

^{1}H-NMR (300 MHz, $CDCl_3$) δ 7.70 – 7.59 (m, 1H), 7.37 – 7.28 (m, 1H), 6.98 (d, J = 8.6 Hz, 1H), 3.93 (s, 3H), 2.34 (s, 3H).

^{13}C-NMR (75 MHz, $CDCl_3$) δ 151.1, 134.9, 130.3, 126.0, 117.6, 113.6, 56.7, 20.3.

The spectra are consistent with the literature.[15]

1,3-Dimethoxy-2-methyl-4-nitrobenzene (27)

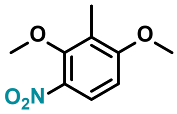

Compound **27** was obtained from 1,3-dimethoxy-2-methylbenzene (0.3 mmol, 1.0 equiv) according to the general procedure **GP2**. It was isolated as a yellow liquid (90% yield).

^{1}H-NMR (300 MHz, $CDCl_3$) δ 7.84 (d, J = 9.2 Hz, 1H), 6.66 (d, J = 9.2 Hz, 1H), 3.90 (s, 3H), 3.87 (s, 3H), 2.18 (s, 3H).

^{13}C-NMR (75 MHz, $CDCl_3$) δ 162.7, 153.6, 137.4, 124.7, 122.5, 105.4, 62.0, 56.2, 9.2.

The spectra are consistent with the literature.[16]

1,2-Dimethoxy-3-methyl-5-nitrobenzene (28a)

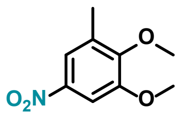

Compound **28a** was obtained using the general procedure **GP2** from 1,2-dimethoxy-3-methylbenzene (0.3 mmol, 1.0 equiv). Isolated as a yellow solid (66% yield).

^{1}H-NMR (300 MHz, $CDCl_3$) δ 7.72 – 7.69 (m, 1H), 7.62 (d, J = 2.7 Hz, 1H), 3.92 (s, 3H), 3.89 (s, 3H), 2.30 (s, 3H).

^{13}C-NMR (75 MHz, $CDCl_3$) δ 153.0, 152.5, 143.4, 132.5, 118.7, 105.7, 60.5, 56.2, 16.3.

FT-IR (ATR, neat; cm^{-1}): 3105, 2943, 2360, 2341, 1486, 1346, 1288.

The spectra are consistent with the literature.[17]

1,2-Dimethoxy-3-methyl-4-nitrobenzene (28b)

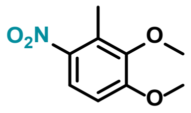

Compound **28b** was obtained according to the general procedure **GP2** from 1,2-dimethoxy-3-methylbenzene (0.3 mmol, 1.0 equiv). Isolated as a yellow solid (13% yield).

^{1}H-NMR (300 MHz, $CDCl_3$) δ 7.30 (d, J = 9.2 Hz, 1H), 6.28 (d, J = 9.2 Hz, 1H), 3.41 (s, 3H), 3.27 (s, 3H), 1.98 (s, 3H).

^{13}C-NMR (75 MHz, $CDCl_3$) δ 157.0, 147.7, 143.3, 129.4, 122.0, 108.8, 60.7, 56.2, 12.8.

FT-IR (ATR, neat; cm^{-1}): 2939, 2843, 2360, 2341, 1510, 1340, 1274.

HRMS (NSI) *m/z,* calcd for $C_9H_{11}NO_4+H^+$: 198.0761; found 198.0763.

5-Methoxy-1,3-dimethyl-2-nitrobenzene (29a)

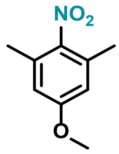

Compound **29a** was obtained according to the general procedure **GP2** from 1-methoxy-3,5-dimethylbenzene (0.3 mmol, 1.0 equiv). Isolated as a colourless solid (53% yield).

^{1}H-NMR (300 MHz, $CDCl_3$) δ 6.60 (s, 2H), 3.81 (s, 3H), 2.32 (s, 6H).

^{13}C-NMR (75 MHz, $CDCl_3$) δ 160.2, 132.6, 117.6, 114.0, 55.6, 18.5.

The spectra are consistent with the literature.[18]

1-Methoxy-3,5-dimethyl-2-nitrobenzene (29b)

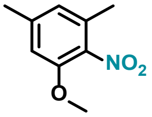

Compound **29b** was obtained according to the general procedure **GP2** from 1-methoxy-3,5-dimethylbenzene (0.3 mmol, 1.0 equiv). It was isolated as a yellow liquid (26% yield).

^{1}H-NMR (300 MHz, $CDCl_3$) δ 6.65 (d, *J* = 4.0 Hz, 2H), 3.84 (s, 3H), 2.34 (s, 3H), 2.25 (s, 3H).

^{13}C-NMR (75 MHz, $CDCl_3$) δ 150.9, 141.5, 130.9, 123.3, 110.8, 56.4, 21.8, 17.1.

The spectra are consistent with the literature.[19]

1-Methyl-4-nitrobenzene and 1-methyl-2-nitrobenzene (30)

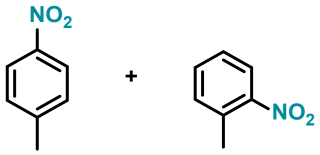

Compounds **30a** and **30b** were obtained according to the general procedure **GP2** from toluene (0.3 mmol, 1.0 equiv). Isolated as a yellow liquid (87% yield) (**30a** *ortho*, 50% and **30b** *para*, 50%, GC-MS yield).

^{1}H-NMR (300 MHz, $CDCl_3$): δ 8.15 – 8.08 (m, 2H), 7.96 (dd, J = 8.4, 1.4 Hz, 1H), 7.50 (td, J = 7.5, 1.4 Hz, 1H), 7.38 – 7.28 (m, 4H), 2.60 (s, 3H), 2.46 (s, 3H).

^{13}C-NMR (75 MHz, $CDCl_3$): δ 146.3, 146.1, 133.7, 133.1, 132.9, 129.9, 127.0, 124.8, 123.7, 117.6, 21.7, 20.5.

The spectra are consistent with the literature. [12]

1,3,5-Trimethyl-2-nitrobenzene (31)

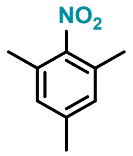

Compound **31** was obtained according to the general procedure **GP2** from mesitylene (0.3 mmol, 1.0 equiv). Isolated as a yellow solid (99% yield).

^{1}H-NMR (300 MHz, $CDCl_3$) δ 6.91 (s, 2H), 2.31 (s, 3H), 2.27 (s, 6H).

^{13}C-NMR (75 MHz, $CDCl_3$) δ 149.9, 140.4, 129.7, 129.5, 21.1, 17.6.

The spectra are consistent with the literature.[12]

1,3,5-Triethyl-2-nitrobenzene (32)

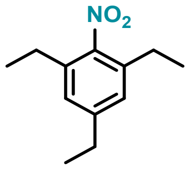

Compound **32** was obtained according to the general procedure **GP2** from 1,3,5-triethylbenzene (0.3 mmol, 1.0 equiv). Isolated as a yellow solid (65% yield).

^{1}H-NMR (300 MHz, $CDCl_3$) δ 6.97 (s, 2H), 2.61 (dq, J = 21.0, 7.6 Hz, 6H), 1.24 (td, J = 7.6, 3.4 Hz, 9H).

^{13}C-NMR (75 MHz, $CDCl_3$) δ 149.4, 146.7, 135.2, 126.8, 28.8, 24.7, 15.5, 15.2.

The spectra are consistent with the literature.[20]

1-Bromo-4-methoxy-2-methyl-5-nitrobenzene (33)

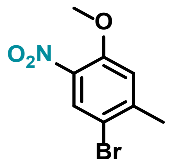

Compound **33** was obtained from 1-bromo-4-methoxy-2-methylbenzene (0.3 mmol, 1.0 equiv) according to the general procedure **GP2**. It was isolated as a yellow liquid (30% yield).

^{1}H-NMR (300 MHz, $CDCl_3$) δ 8.06 (d, J = 1.5 Hz, 1H), 6.96 (s, 1H), 3.94 (s, 3H), 2.45 (s, 3H).

The spectra are consistent with the literature.[21]

1-Fluoro-2,5-dimethoxy-3-nitrobenzene (34)

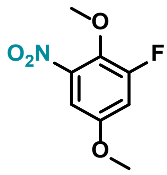

Compound **34** was obtained using **GP2** from 2-fluoro-1,4-dimethoxybenzene (0.3 mmol, 1.0 equiv). Isolated as a yellow solid (99% yield).

^{1}H-NMR (300 MHz, $CDCl_3$) δ 7.62 (d, J = 8.7 Hz, 1H), 6.88 (d, J = 12.1 Hz, 1H), 3.91 (d, J = 6.4 Hz, 6H).

^{13}C-NMR (75 MHz, $CDCl_3$) δ 157.6, 154.2, 149.1, 149.0, 141.3, 141.1, 111.6, 111.6, 103.3, 102.9, 57.4, 57.1.

^{19}F-NMR (282 MHz, $CDCl_3$) δ -120.80.

HRMS (EI) *m/z,* calcd for $C_8H_8O_4NF$: 201.0432; found 201.0434.

2-Bromo-1-methoxy-4-nitrobenzene (35a)

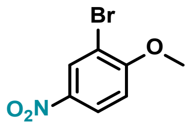

Compound **35a** was obtained according to the general procedure **GP2** from 1-bromo-2-methoxybenzene (0.3 mmol, 1.0 equiv). Isolated as a yellow liquid (64% yield).

^{1}H-NMR (300 MHz, $CDCl_3$) δ 8.47 (d, J = 2.7 Hz, 1H), 8.22 (dd, J = 9.1, 2.7 Hz, 1H), 6.96 (d, J = 9.1 Hz, 1H), 4.01 (s, 3H).

^{13}C-NMR (75 MHz, $CDCl_3$) δ 161.1, 141.7, 129.3, 124.9, 112.0, 110.9, 57.1.

The spectra are consistent with the literature.[22]

1-Bromo-2-methoxy-3-nitrobenzene (35b)

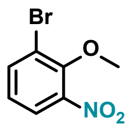

Compound **35b** was obtained according to the general procedure **GP2** from 1-bromo-2-methoxybenzene (0.3 mmol, 1.0 equiv). Isolated as a yellow liquid (32% yield).

^{1}H-NMR (300 MHz, $CDCl_3$) δ 7.78 (ddd, J = 10.2, 8.2, 1.6 Hz, 2H), 7.12 (t, J = 8.1 Hz, 1H), 4.03 (s, 3H).

^{13}C-NMR (75 MHz, CDCl3) δ 151.7, 151.0, 138.0, 125.2, 124.5, 120.0, 62.8.

The spectra are consistent with the literature[23]

1-Bromo-4-(tert-butyl)-2-nitrobenzene (36)

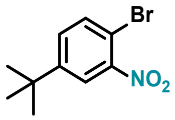

Compound **36** was obtained according to the general procedure **GP2** from 1-bromo-4-(tert-butyl)benzene (0.3 mmol, 1.0 equiv). It was isolated as a yellow liquid (60% yield).

^{1}H-NMR (300 MHz, $CDCl_3$): δ 7.83 (d, *J* = 2.4 Hz, 1H), 7.64 (d, *J* = 8.5 Hz, 1H), 7.44 (dd, *J* = 8.5, 2.3 Hz, 1H), 1.34 (s, 9H).

The spectra are consistent with the literature.[24]

2-Iodo-1,3-dimethoxy-4-nitrobenzene (37)

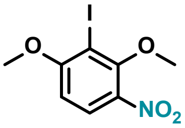

Compound **37** was obtained according to the general procedure **GP2** from 2-iodo-1,3-dimethoxybenzene (0.3 mmol, 1.0 equiv). It was isolated as a yellow solid (26% yield).

^{1}H-NMR (300 MHz, $CDCl_3$): δ 8.03 (d, *J* = 9.2 Hz, 1H), 6.68 (d, *J* = 9.2 Hz, 1H), 3.98 (d, *J* = 1.7 Hz, 6H).

^{13}C-NMR (75 MHz, $CDCl_3$): δ 163.6, 155.8, 127.7, 117.6, 106.0, 87.0, 62.6, 57.3.

HRMS (EI) *m/z,* calcd for $C_8H_8INO_4$: 308.9498; found 308.9496.

4-Methoxy-1-nitro-2-(trifluoromethyl)benzene (38a)

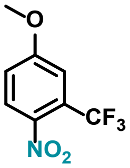

Compound **38a** was obtained using general procedure **GP2** from 1-methoxy-3-(trifluoromethyl)benzene (0.3 mmol, 1.0 equiv). Isolated as a colourless liquid (15% yield).

^{1}H-NMR (300 MHz, $CDCl_3$) δ 8.02 (d, J = 9.0 Hz, 1H), 7.30 (d, J = 2.8 Hz, 1H), 7.12 (dd, J = 9.0, 2.8 Hz, 1H), 3.94 (s, 3H).

^{13}C-NMR (75 MHz, $CDCl_3$) δ 128.3, 117.6, 116.4, 114.5, 114.4, 56.4.

^{19}**F-NMR** (282 MHz, $CDCl_3$) δ -60.18.

HRMS (EI) *m/z,* calcd for C8H6O3NF3: 221.0294; found 221.0297.

2-Methoxy-1-nitro-4-(trifluoromethyl)benzene (38b)

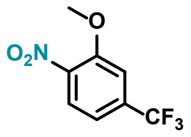

Compound **38b** was obtained using the general procedure **GP2** from 1-methoxy-3-(trifluoromethyl)benzene (0.3 mmol, 1.0 equiv). Isolated as a colourless liquid (24% yield).

^{1}**H-NMR** (300 MHz, $CDCl_3$) δ 7.90 (dq, J = 8.8, 0.9 Hz, 1H), 7.36 – 7.28 (m, 2H), 4.02 (s, 3H).

^{13}**C-NMR** (75 MHz, $CDCl_3$) δ 153.0, 135.9, 135.4, 126.1, 124.8, 121.2, 117.4, 110.9, 57.0.

^{19}**F-NMR** (282 MHz, $CDCl_3$) δ -63.26.

The spectra are consistent with the literature.[25]

Methyl 5-chloro-2-methoxy-3-nitrobenzoate (39)

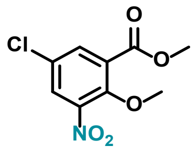

Compound **39** was obtained according to the general procedure **GP2** from methyl 5-chloro-2-methoxybenzoate (0.3 mmol, 1.0 equiv). Isolated as a yellow solid (87% yield).

^{1}**H-NMR** (300 MHz, $CDCl_3$) δ 7.99 (d, J = 2.7 Hz, 1H), 7.89 (d, J = 2.7 Hz, 1H), 3.99 (s, 3H), 3.96 (s, 3H).

^{13}**C-NMR** (75 MHz, $CDCl_3$) δ 163.7, 152.1, 145.9, 135.5, 129.2, 128.8, 128.4, 64.7, 53.2.

FT-IR (ATR, neat; cm^{-1}): 3087, 2962, 2359, 2342, 1730, 1533, 1248.

HRMS (EI) *m/z,* calcd for $C_9H_8O_5NCl$: 245.0091; found 245.0090.

2-Methoxy-5-nitrobenzaldehyde (40)

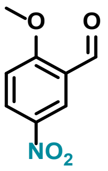

Compound **40** was obtained according to the general procedure **GP2** from 2-methoxybenzaldehyde (0.3 mmol, 1.0 equiv). Isolated as an off-white solid (22% yield).

^{1}**H-NMR** (300 MHz, $CDCl_3$): δ 10.45 (s, 1H), 8.71 (d, *J* = 2.9 Hz, 1H), 8.45 (dd, *J* = 9.2, 2.9 Hz, 1H), 7.12 (d, *J* = 9.2 Hz, 1H), 4.08 (s, 3H).

The spectra are consistent with the literature.[26]

4-Nitrophenyl acetate and 2-nitrophenyl acetate (41)

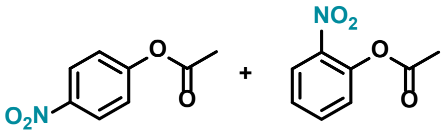

Compound **41** was obtained using the general procedure **GP2** from phenyl acetate (0.3 mmol, 1.0 equiv). Isolated as a yellow liquid (**41a** 4-nitro: 37% yield and **41b** 2-nitro: 16% yield).

^{1}**H-NMR** (300 MHz, $CDCl_3$): δ 8.31 – 8.21 (m, 2H), 7.27 (dd, *J* = 8.9, 1.9 Hz, 2H), 2.34 (s, 3H).

^{1}**H-NMR** (300 MHz, $CDCl_3$): δ 8.09 (dd, *J* = 8.2, 1.6 Hz, 1H), 7.65 (ddd, *J* = 8.2, 7.5, 1.7 Hz, 1H), 7.39 (ddd, *J* = 8.2, 7.5, 1.4 Hz, 1H), 7.23 (dd, *J* = 8.1, 1.4 Hz, 1H), 2.37 (s, 3H).

The spectra are consistent with the literature.[27] [27b]

1-Nitronaphthalene (42)

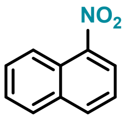

Compound **42** was obtained according to the general procedure **GP2** from naphthalene (0.3 mmol, 1.0 equiv). Isolated as a yellow solid (87% yield).

^{1}**H-NMR** (300 MHz, $CDCl_3$): δ 8.57 (dq, *J* = 8.7, 1.0 Hz, 1H), 8.23 (dd, *J* = 7.7, 1.3 Hz, 1H), 8.12 (dd, *J* = 8.3, 1.2 Hz, 1H), 8.00 – 7.91 (m, 1H), 7.72 (ddd, *J* = 8.6, 6.9, 1.4 Hz, 1H), 7.62 (ddd, *J* = 8.2, 6.9, 1.2 Hz, 1H), 7.54 (t, *J* = 7.9 Hz, 1H).

^{13}**C-NMR** (75 MHz, $CDCl_3$): δ 146.7, 134.8, 134.5, 129.6, 128.7, 127.5, 125.3, 124.3, 124.1, 123.3.

The spectra are consistent with the literature.[14]

2-Methoxy-1-nitronaphthalene (43a)

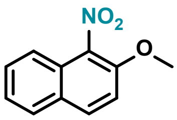

Compound **43a** was obtained according to the general procedure **GP2** from 2-methoxynaphthalene (0.3 mmol, 1.0 equiv). Isolated as a yellow solid (51% yield).

^{1}H-NMR (300 MHz, $CDCl_3$): δ 7.97 (d, *J* = 9.2 Hz, 1H), 7.84 (d, *J* = 8.2 Hz, 1H), 7.71 – 7.65 (m, 1H), 7.60 (ddd, *J* = 8.5, 6.7, 1.3 Hz, 1H), 7.45 (ddd, *J* = 8.1, 6.7, 1.3 Hz, 1H), 7.34 (d, *J* = 9.2 Hz, 1H), 4.04 (s, 3H).

The spectra are consistent with the literature.[13]

2-Methoxy-6-nitronaphthalene (43b)

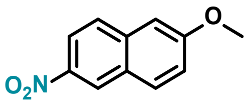

Compound **43b** was obtained according to the general procedure **GP2** from 2-methoxynaphthalene (0.3 mmol, 1.0 equiv). Isolated as a yellow solid (9% yield).

^{1}H-NMR (300 MHz, $CDCl_3$): δ 8.73 (d, *J* = 2.3 Hz, 1H), 8.21 (dd, *J* = 9.0, 2.3 Hz, 1H), 7.92 (d, *J* = 9.0 Hz, 1H), 7.81 (d, *J* = 9.1 Hz, 1H), 7.32 – 7.27 (m, 1H), 7.21 (d, *J* = 2.5 Hz, 1H), 3.98 (s, 3H).

The spectra are consistent with the literature.[28]

2-Methoxy-8-nitronaphthalene (43c)

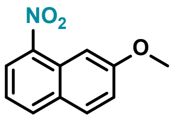

Compound **43c** was obtained according to the general procedure **GP2** from 2-methoxynaphthalene (0.3 mmol, 1.0 equiv). Isolated as a yellow solid (13% yield).

^{1}H-NMR (300 MHz, $CDCl_3$): δ 8.30 (dd, *J* = 7.8, 1.3 Hz, 1H), 8.10 – 8.00 (m, 2H), 7.85 (d, *J* = 9.0 Hz, 1H), 7.40 (t, *J* = 7.9 Hz, 1H), 7.29 (d, *J* = 2.5 Hz, 1H), 3.98 (s, 3H).

The spectra are consistent with the literature.[29]

9-Bromo-10-nitroanthracene (44)

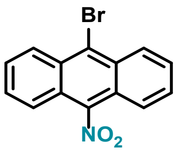

Compound **44** was obtained according to the general procedure **GP2** from 9-bromoanthracene (0.3 mmol, 1.0 equiv). Isolated as a yellow solid (31% yield).

^{1}H-NMR (300 MHz, $CDCl_3$): δ 8.66 – 8.58 (m, 2H), 7.89 (ddd, *J* = 6.8, 4.0, 2.9 Hz, 2H), 7.72 – 7.65 (m, 4H).

The spectra are consistent with the literature.[30]

2-Nitrodibenzo[b,d]thiophene (45)

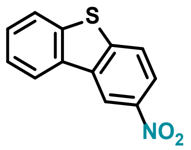

Compound **45** was obtained according to the general procedure **GP2** from dibenzo[b,d]thiophene (0.3 mmol, 1.0 equiv). Isolated as an off-white solid (32% yield).

^{1}**H-NMR** (300 MHz, $CDCl_3$): δ 8.99 (d, *J* = 2.2 Hz, 1H), 8.31 (dd, *J* = 8.8, 2.2 Hz, 1H), 8.27 – 8.21 (m, 1H), 7.95 (d, *J* = 8.8 Hz, 1H), 7.92 – 7.87 (m, 1H), 7.60 – 7.52 (m, 2H).

^{13}**C-NMR** (75 MHz, $CDCl_3$): δ 146.2, 145.6, 140.4, 136.1, 134.7, 128.4, 125.5, 123.3, 123.2, 122.4, 121.2, 117.2.

The spectra are consistent with the literature.[31]

1-(4-Nitrophenyl)-2-phenyldiazene (46)

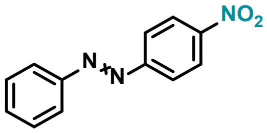

Compound **46** was obtained according to the general procedure **GP2** from 1,2-diphenyldiazene (0.3 mmol, 1.0 equiv). Isolated as an orange solid (29% yield).

^{1}**H-NMR** (300 MHz, $CDCl_3$) δ 8.43 – 8.35 (m, 2H), 8.08 – 8.01 (m, 2H), 8.00 – 7.94 (m, 2H), 7.59 – 7.53 (m, 3H).

The spectra are consistent with the literature.[32]

9-(4-Bromophenyl)-3-nitro-9H-carbazole (47)

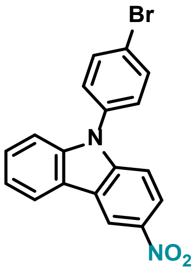

Compound **47** was obtained according to the general procedure **GP2** from 9-(4-bromophenyl)-9H-carbazole (0.3 mmol, 1.0 equiv). Isolated as a yellow solid (30% yield).

^{1}**H-NMR** (300 MHz, $CDCl_3$): δ 9.06 (dd, *J* = 2.3, 0.5 Hz, 1H), 8.33 (dd, *J* = 9.1, 2.3 Hz, 1H), 8.21 (ddd, *J* = 7.8, 1.3, 0.8 Hz, 1H), 7.83 – 7.75 (m, 2H), 7.52 (ddd, *J* = 8.3, 7.3, 1.3 Hz, 1H), 7.47 – 7.40 (m, 3H), 7.40 – 7.32 (m, 2H).

^{13}C-NMR (75 MHz, $CDCl_3$): δ 143.8, 142.2, 141.8, 135.6, 133.7, 128.9, 128.0, 123.4, 123.2, 122.5, 122.1, 122.0, 121.2, 117.5, 110.6, 109.5.

The spectra are consistent with the literature.[33]

(*8R,9S,13S,14S*)-3-Methoxy-13-methyl-2-nitro-6,7,8,9,11,12,13,14,15,16-decahydro-17H-cyclopenta[a]phenanthren-17-one (48a)

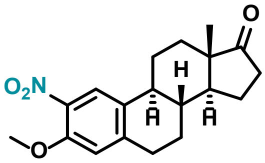

Compound **48a** was obtained according to general procedure **GP2** from (8R,9S,13S,14S)-3-methoxy-13-methyl-6,7,8,9,11,12,13,14,15,16-decahydro-17H-cyclopenta[a]phenanthren-17-one (0.3 mmol, 1.0 equiv). Isolated as a green liquid (25% yield).

^{1}H-NMR (300 MHz, $CDCl_3$) δ 7.81 (s, 1H), 6.78 (s, 1H), 3.92 (s, 3H), 3.00 – 2.90 (m, 2H), 2.51 (dd, *J* = 18.3, 8.4 Hz, 1H), 2.44 – 2.34 (m, 1H), 2.29 – 1.93 (m, 5H), 1.68 – 1.41 (m, 6H), 0.91 (s, 3H).

^{13}C-NMR (75 MHz, $CDCl_3$) δ 151.3, 144.5, 137.5, 132.6, 123.2, 113.7, 56.6, 50.4, 48.0, 47.2, 43.6, 38.0, 35.9, 31.5, 29.9, 26.2, 25.8, 21.6, 13.9.

HRMS (ESI) *m/z,* calcd for $C_{19}H_{23}NO_4$+Na: 352.1519; found 352.1514.

(*8R,9S,13S,14S*)-3-Methoxy-13-methyl-4-nitro-6,7,8,9,11,12,13,14,15,16-decahydro-17H-cyclopenta[a]phenanthren-17-one (48b)

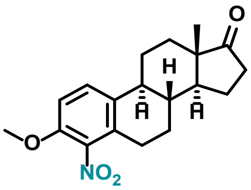

Compound **48b** was obtained according to general procedure **GP2** from (8R,9S,13S,14S)-3-methoxy-13-methyl-6,7,8,9,11,12,13,14,15,16-decahydro-17H-cyclopenta[a]phenanthren-17-one (0.3 mmol, 1.0 equiv). Isolated as a light green solid (16% yield).

^{1}H-NMR (300 MHz, $CDCl_3$) δ 7.34 (dd, *J* = 8.9, 1.2 Hz, 1H), 6.85 (d, *J* = 8.8 Hz, 1H), 3.86 (s, 3H), 2.80 (dd, *J* = 7.7, 3.3 Hz, 2H), 2.51 (dd, *J* = 18.6, 8.4 Hz, 1H), 2.45 – 2.35 (m, 1H), 2.32 – 1.93 (m, 5H), 1.69 – 1.35 (m, 6H), 0.91 (s, 3H).

^{13}C-NMR (75 MHz, $CDCl_3$) δ 148.7, 141.8, 133.3, 129.0, 127.8, 110.1, 56.5, 50.3, 48.0, 44.0, 37.7, 35.9, 31.6, 29.8, 26.1, 25.6, 24.0, 21.6, 13.9.

HRMS (ESI) *m/z,* calcd for $C_{19}H_{23}NO_4$+Na: 352.1519; found 352.1519.

Isopropyl 2-(4-(4-chlorobenzoyl)-2-nitrophenoxy)-2-methylpropanoate (49)

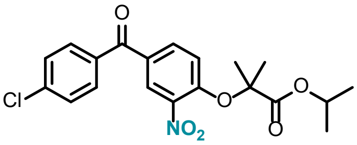

Compound **49** was obtained according to the general procedure **GP2** from Fenofibrate (0.3 mmol, 1.0 equiv). Isolated as a colourless liquid (64% yield).

^{1}H-NMR (300 MHz, $CDCl_3$) δ 8.17 (d, J = 2.2 Hz, 1H), 7.89 (dd, J = 8.8, 2.2 Hz, 1H), 7.73 – 7.65 (m, 2H), 7.50 – 7.43 (m, 2H), 6.98 (d, J = 8.8 Hz, 1H), 5.08 (hept, J = 6.3 Hz, 1H), 1.70 (s, 6H), 1.21 (d, J = 6.3 Hz, 6H).

^{13}C NMR (75 MHz, $CDCl_3$) δ 192.2, 172.2, 152.5, 141.8, 139.5, 135.1, 134.2, 131.2, 130.2, 129.1, 127.4, 118.4, 82.2, 70.0, 25.3, 21.6.

The spectra are consistent with the literature.[12]

8-Bromooctyl nitrate (55)

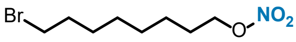

Compound **55** was obtained from 8-bromooctan-1-ol (0.3 mmol, 1.0 equiv) according to the general procedure **GP1**. It was isolated as a colourless liquid (86% yield).

^{1}H-NMR (300 MHz, $CDCl_3$): δ 4.44 (t, J = 6.7 Hz, 2H), 3.40 (t, J = 6.8 Hz, 2H), 1.85 (dt, J = 14.7, 6.8 Hz, 2H), 1.70 (dt, J = 8.7, 7.1 Hz, 2H), 1.49 – 1.28 (m, 8H).

^{13}C-NMR (75 MHz, $CDCl_3$): δ 73.5, 34.0, 32.8, 29.0, 28.6, 28.1, 26.8, 25.7.

HRMS (ESI) *m/z,* calcd for $C_8H_{16}BrNO_3$–HNO_3: 192.0514; found 192.0512.

8-Hydroxyoctyl nitrate (56)

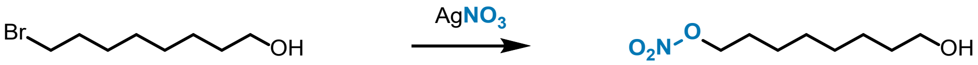

Compound **56** was obtained according to the reported procedure[34] from 8-bromooctan-1-ol (0.3 mmol, 1.0 equiv). Isolated as a colourless liquid (80% yield).

^{1}H-NMR (300 MHz, $CDCl_3$): δ 4.40 (t, J = 6.7 Hz, 2H), 3.58 (t, J = 6.6 Hz, 2H), 1.99 (s, 1H), 1.74 – 1.61 (m, 2H), 1.52 (t, J = 6.9 Hz, 2H), 1.39 – 1.24 (m, 8H).

^{13}C-NMR (75 MHz, $CDCl_3$): δ 73.5, 62.8, 32.7, 29.2, 29.1, 26.8, 25.7, 25.6.

HRMS (ESI) *m/z,* calcd for $C_8H_{17}NO_4$+Na^+: 214.1055; found 214.1055.

C. Post-Functionalizations of Nitrate Esters

1-Nitro-4-(2-thiocyanatoethyl)benzene (51)

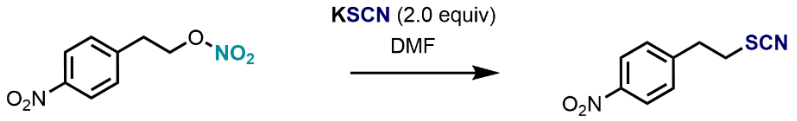

Nitronitrooxylated compound (0.5 mmol) was dissolved in DMF (2 mL), and KSCN (1.0 mmol, 2 equiv) was added. The mixture was stirred for 12 h at 70°C and was then washed with a saturated Na_2CO_3 solution (2 mL) and extracted with diethyl ether (3 × 5 mL). The organic layer was washed with water (3 × 10 mL) and brine and then dried over magnesium sulfate, filtered, and concentrated under vacuum. The crude mixture was purified with column chromatography on silica gel (Hex/EtOAc). Compound **51** was isolated with a 92%.

^{1}H-NMR (300 MHz, $CDCl_3$): δ 8.22 (d, *J* = 8.7 Hz, 2H), 7.42 (d, *J* = 8.6 Hz, 2H), 3.30 – 3.15 (m, 4H).

^{13}C-NMR (75 MHz, $CDCl_3$): δ 145.0, 129.8, 124.3, 117.6, 111.5, 35.8, 34.4.

HRMS (EI) *m/z,* calcd for $C_9H_8N_2O_2S$: 208.0306; found 208.0304.

2-(Bromomethyl)isoindoline-1,3-dione (52)

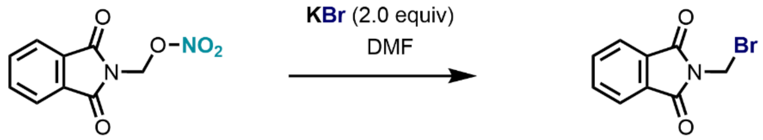

Nitronitrooxylated compound (0.5 mmol) was dissolved in DMF (2 mL), and KBr (1.0 mmol, 2 equiv) was added. The mixture was stirred for 12 h at 70°C and was then washed with a saturated Na_2CO_3 solution (2 mL) and extracted with diethyl ether (3 × 5 mL). The organic layer was washed with water (3 × 10 mL) and brine and then dried over magnesium sulfate, filtered, and concentrated under vacuum. The crude mixture was purified with column chromatography on silica gel (Hex/EtOAc). Compound **52** was isolated in 90%.

^{1}H-NMR (300 MHz, $CDCl_3$): δ 7.95 – 7.88 (m, 2H), 7.78 (dd, *J* = 5.5, 3.1 Hz, 2H), 5.47 (s, 2H).

^{13}C-NMR (75 MHz, $CDCl_3$): δ 165.8, 134.9, 132.0, 124.1, 31.3.

The spectra are consistent with the literature.[35]

3-(4-Nitrophenyl)propanenitrile synthesis (53)

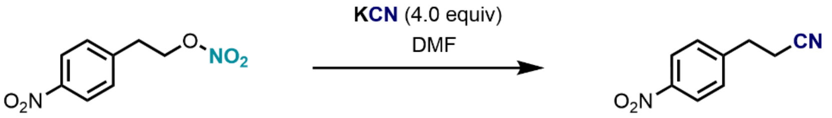

Nitronitrooxylated compound (0.5 mmol) was dissolved in DMF (2 mL), and KCN (2.0 mmol, 4 equiv) was added. The mixture was stirred for 12 h at 50°C and was then washed with a saturated Na_2CO_3 solution (2 mL) and extracted with diethyl ether (3 × 5 mL). The organic layer was washed with water (5 × 10 mL) and brine and then dried over magnesium sulfate, filtered, and concentrated under vacuum. The crude mixture was purified with column chromatography on silica gel (Hex/EtOAc). Compound **53** was isolated in 84%.

^{1}H-NMR (300 MHz, $CDCl_3$): δ 8.25 – 8.15 (m, 2H), 7.47 – 7.38 (m, 2H), 3.08 (t, *J* = 7.2 Hz, 2H), 2.70 (t, *J* = 7.2 Hz, 2H).

The spectra are consistent with the literature.[36]

2-(Azidomethyl)isoindoline-1,3-dione (54)

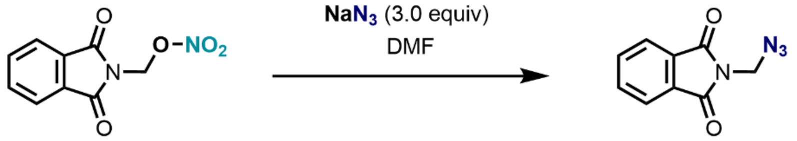

Nitronitrooxylated compound (0.5 mmol) was dissolved in DMF (2 mL), and NaN_3 (1.5 mmol, 3 equiv) was added. The mixture was stirred for 12 h at 60°C and was then washed with a saturated Na_2CO_3 solution (2 mL) and extracted with diethyl ether (3 × 5 mL). The organic layer was washed with water (3 × 10 mL) and brine and then dried over magnesium sulfate, filtered, and concentrated under vacuum. The crude mixture was purified with column chromatography on silica gel (Hex/EtOAc). Yield of isolated compound is 88%.

^{1}H-NMR (300 MHz, $CDCl_3$): δ 7.88 (dd, *J* = 5.6, 3.0 Hz, 2H), 7.77 (td, *J* = 5.3, 2.1 Hz, 2H), 5.04 (s, 2H).

^{13}C-NMR (75 MHz, $CDCl_3$): δ 167.1, 134.7, 131.7, 124.0, 52.3.

The spectra are consistent with the literature.[37]

10. NMR Spectra of Isolated Compounds

4-nitrophenethyl nitrate (1)

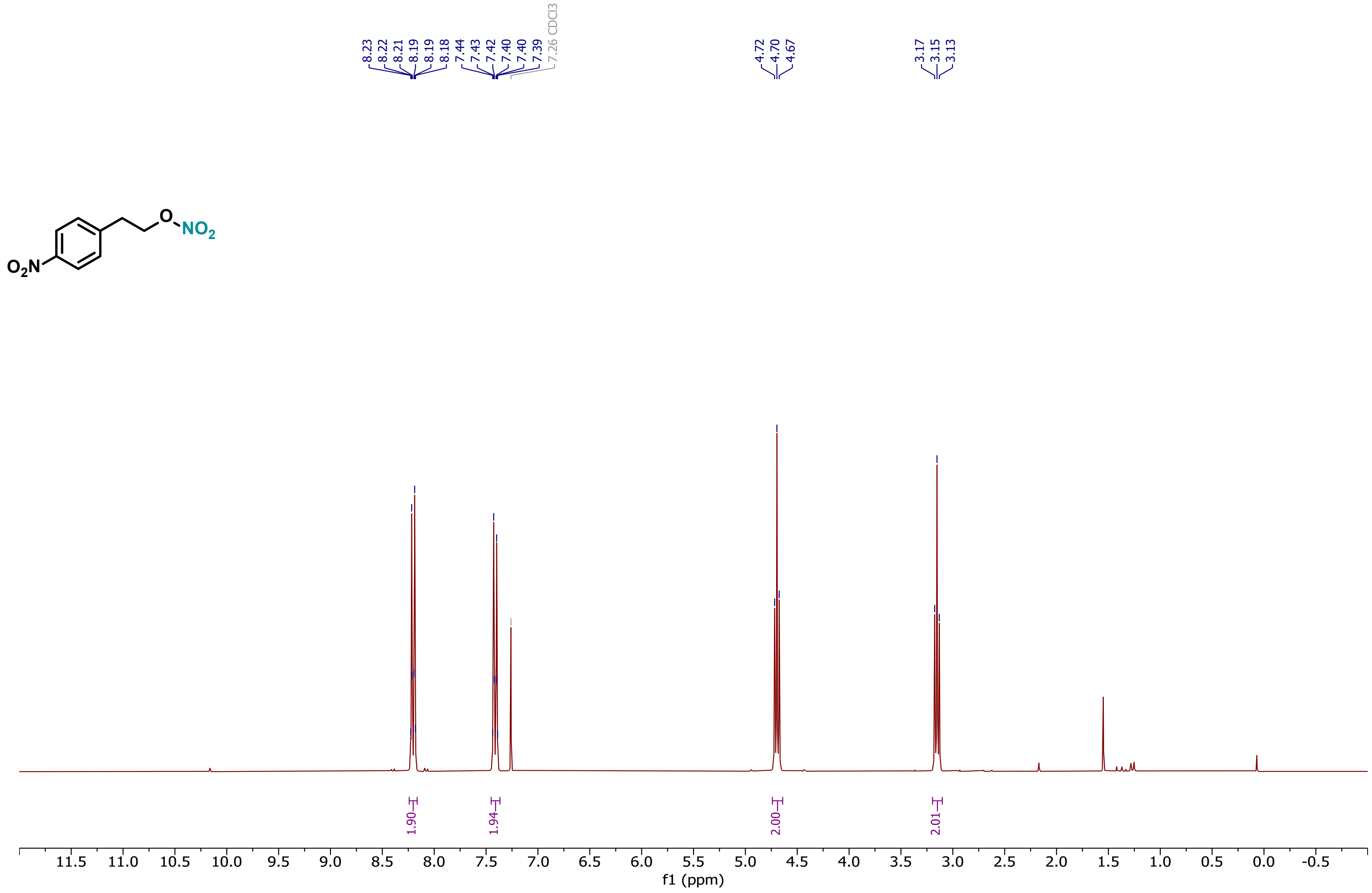

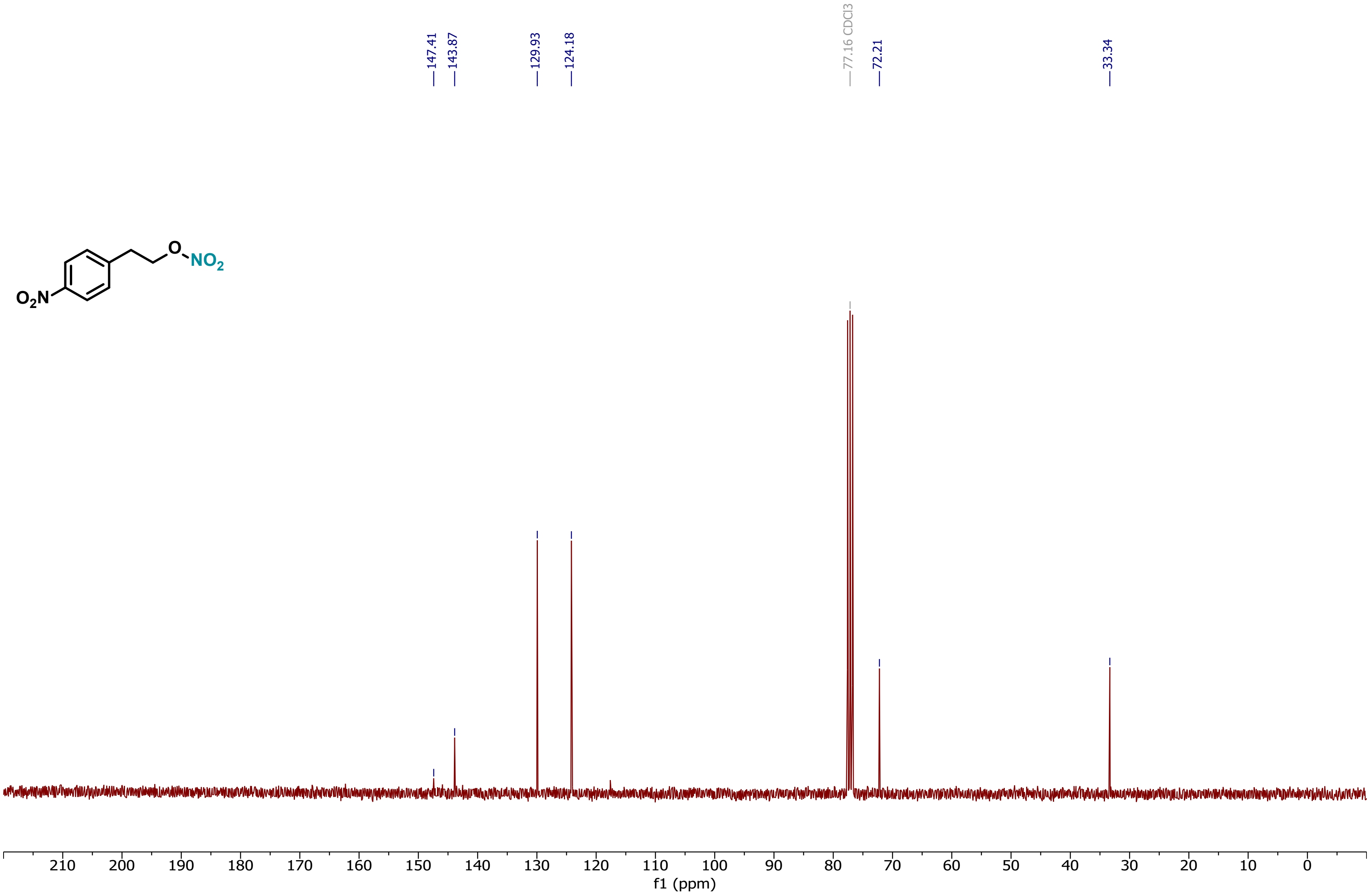
147.41
143.87
129.93
124.18
77.16 CDCl3
72.21
33.34
O
NO2
O2N
210
200
190
180
170
160
150
140
130
120
110
100
90
80
70
60
50
40
30
20
10
0
f1 (ppm)

phenethyl nitrate (2)

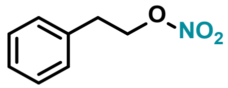

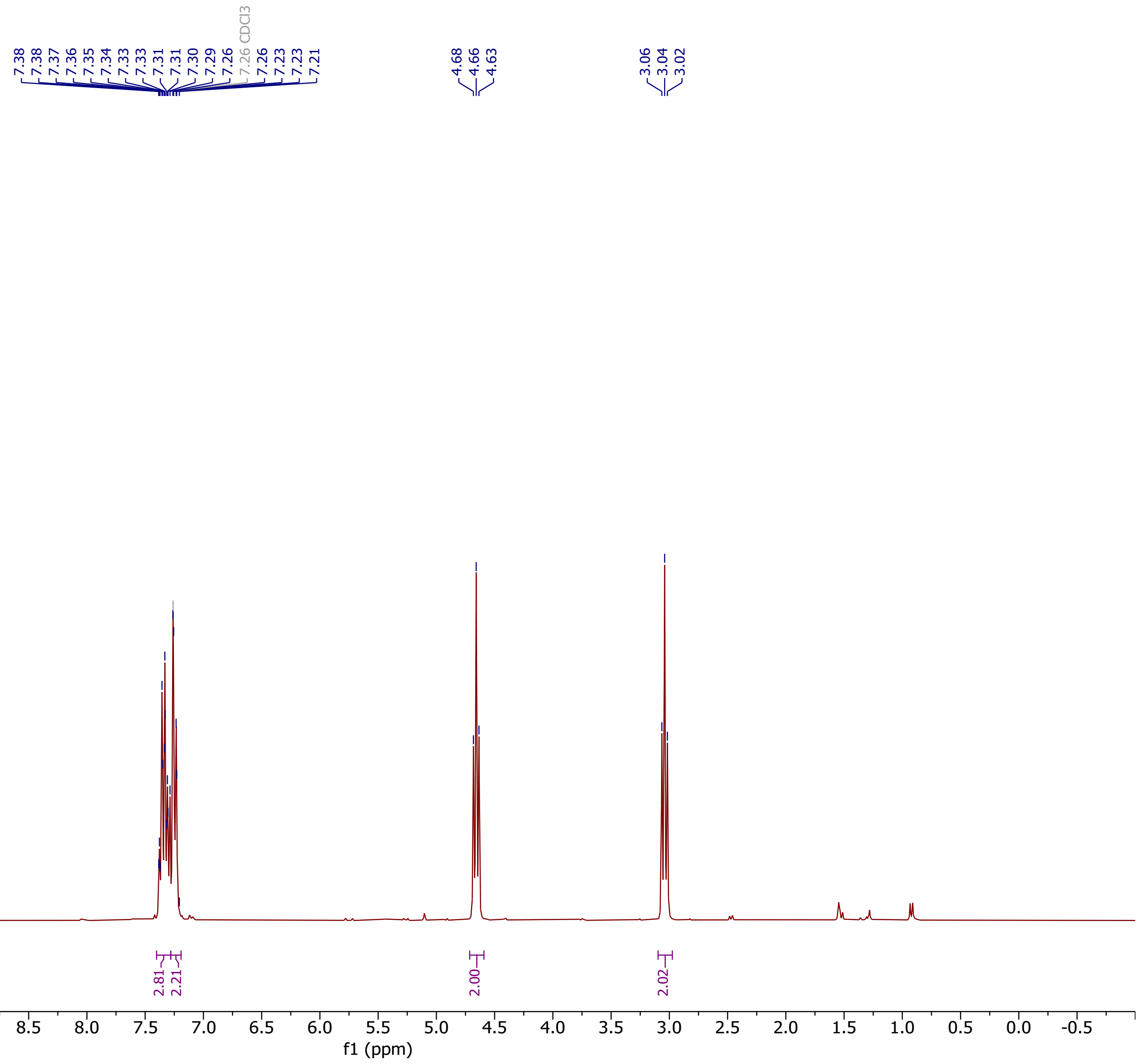

4-phenylbutyl nitrate (3)

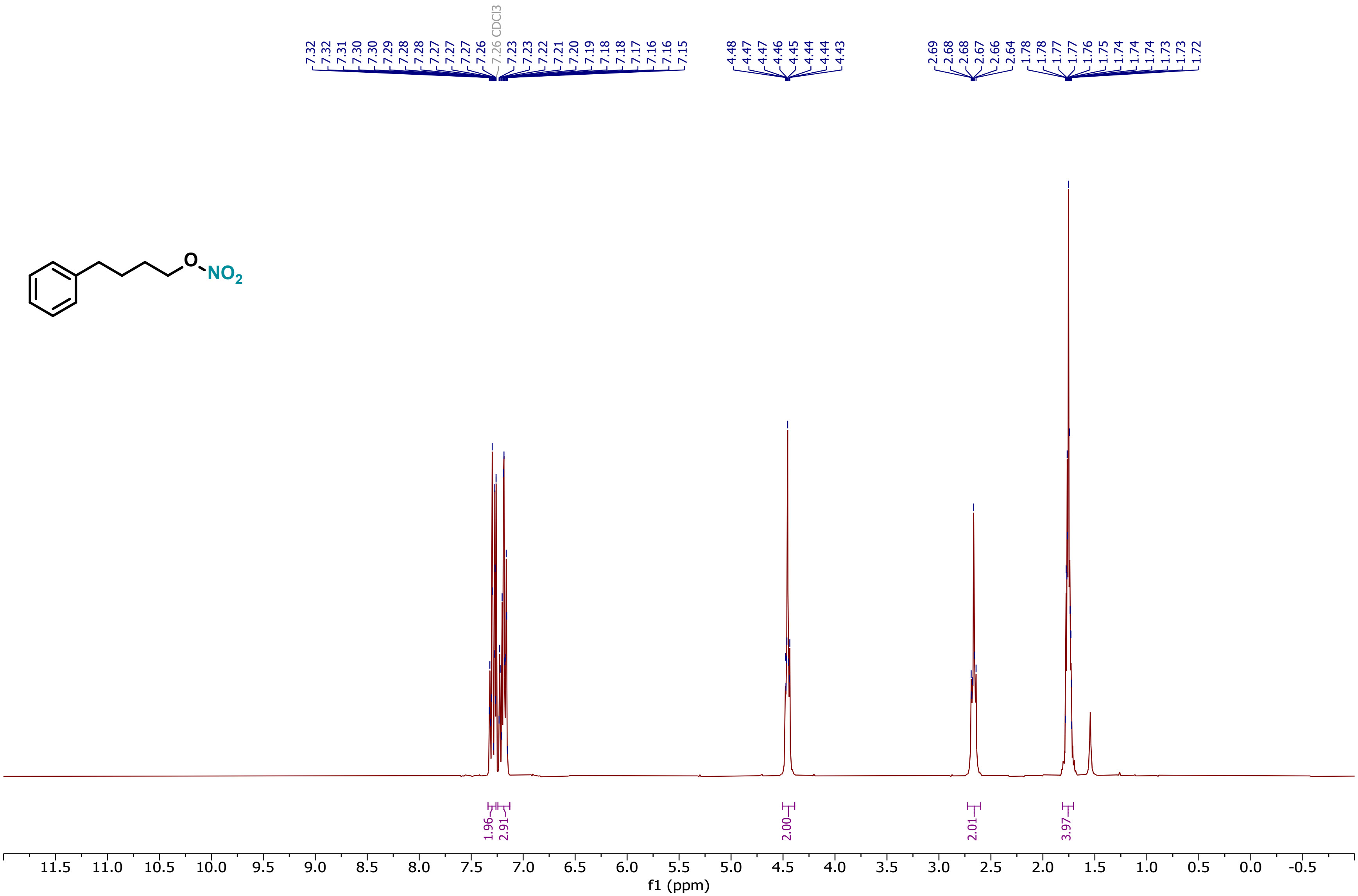

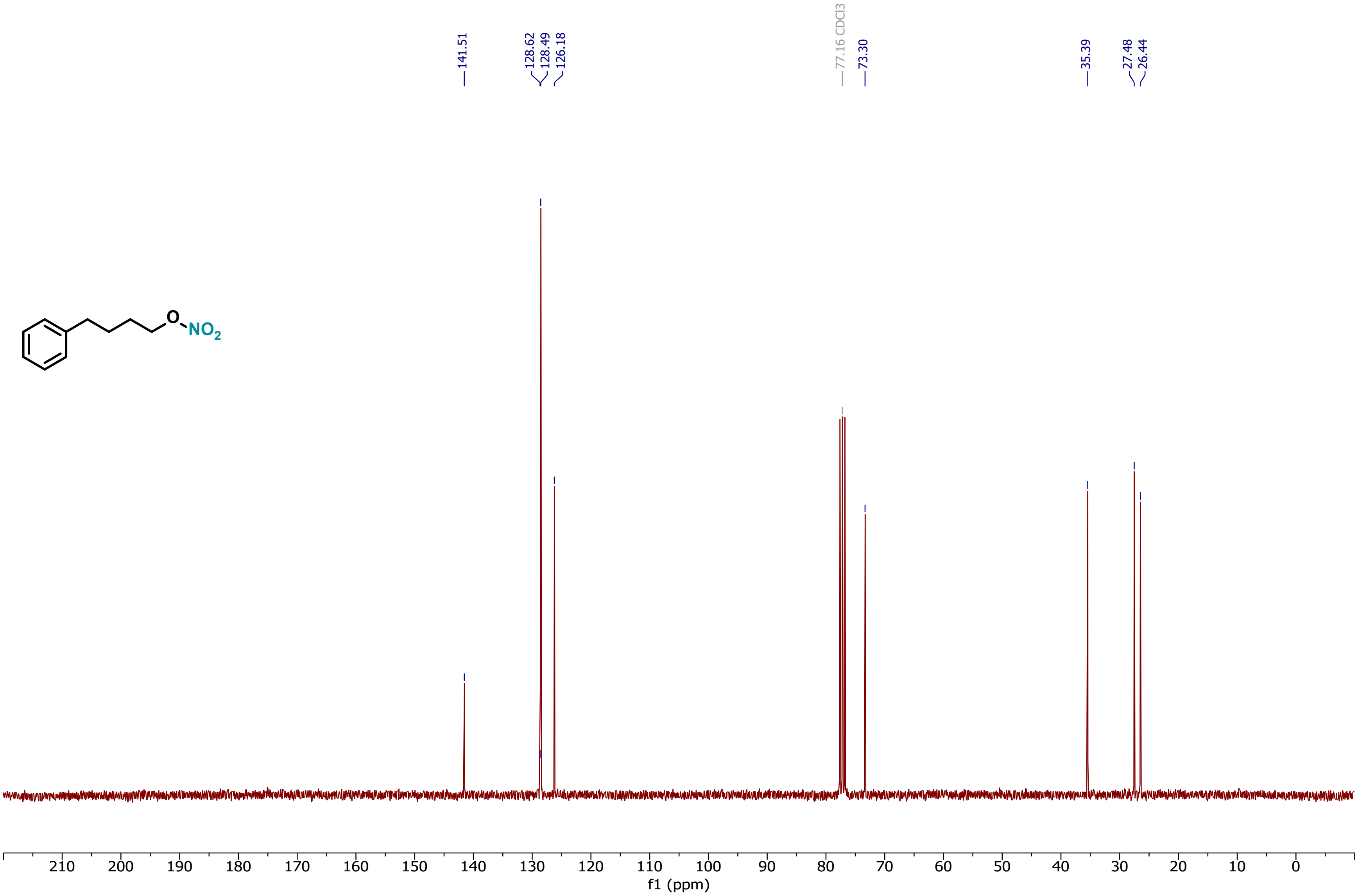
141.51
128.62
128.49
126.18
77.16 CDCl3
73.30
35.39
27.48
26.44
NO2
O
210 200 190 180 170 160 150 140 130 120 110 100 90 80 70 60 50 40 30 20 10 0
f1 (ppm)

4-(4-methoxyphenyl)butyl nitrate (4)

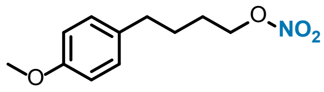

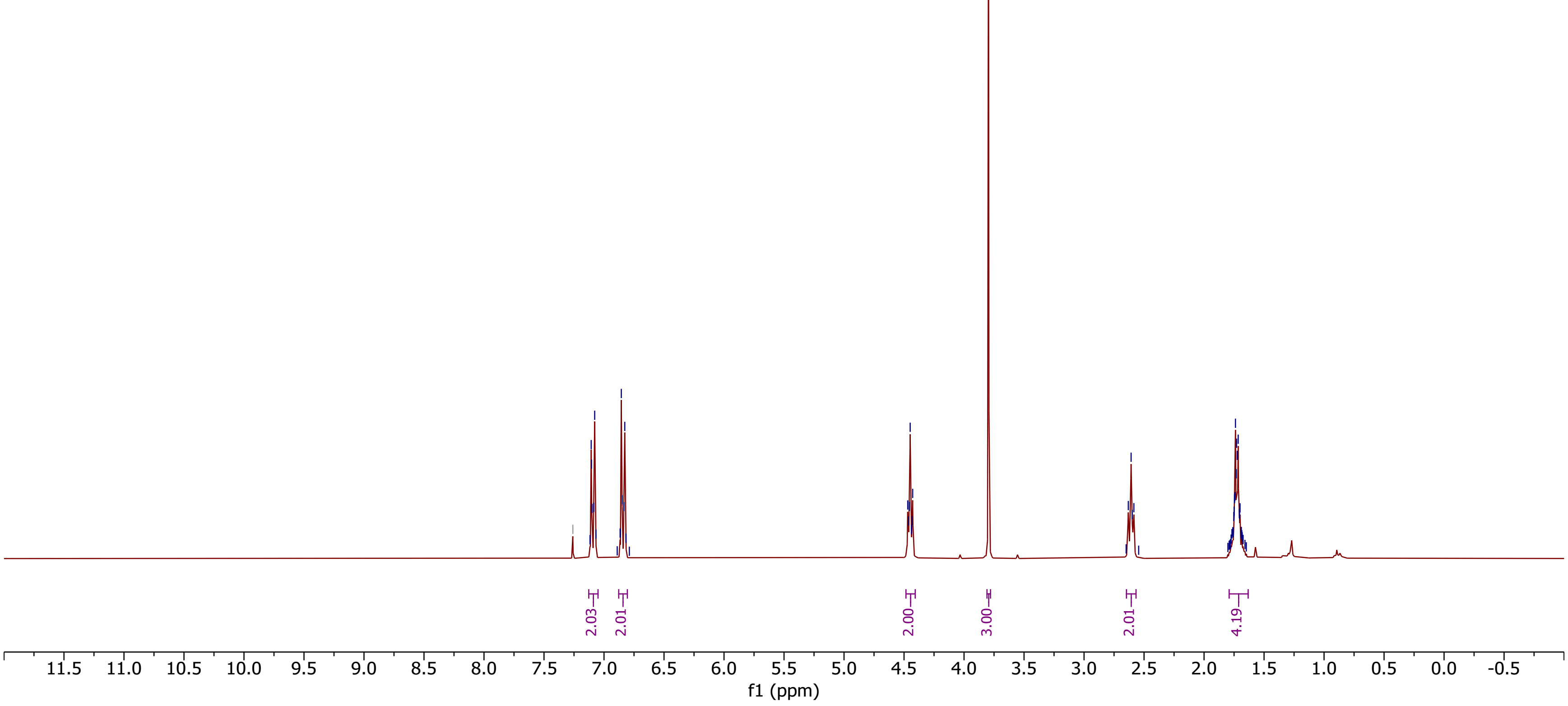

2,4,6-trimethyl-3-nitrophenethyl nitrate (5)

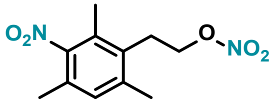

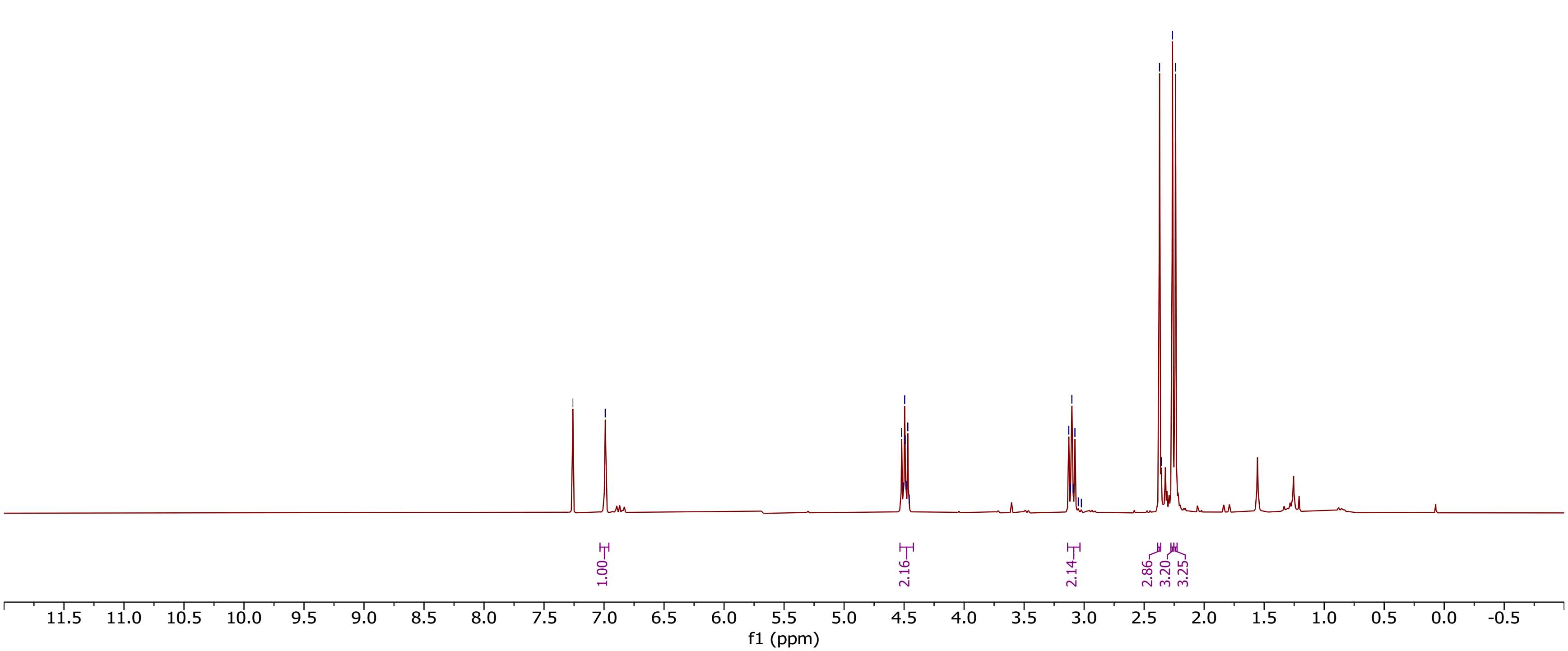

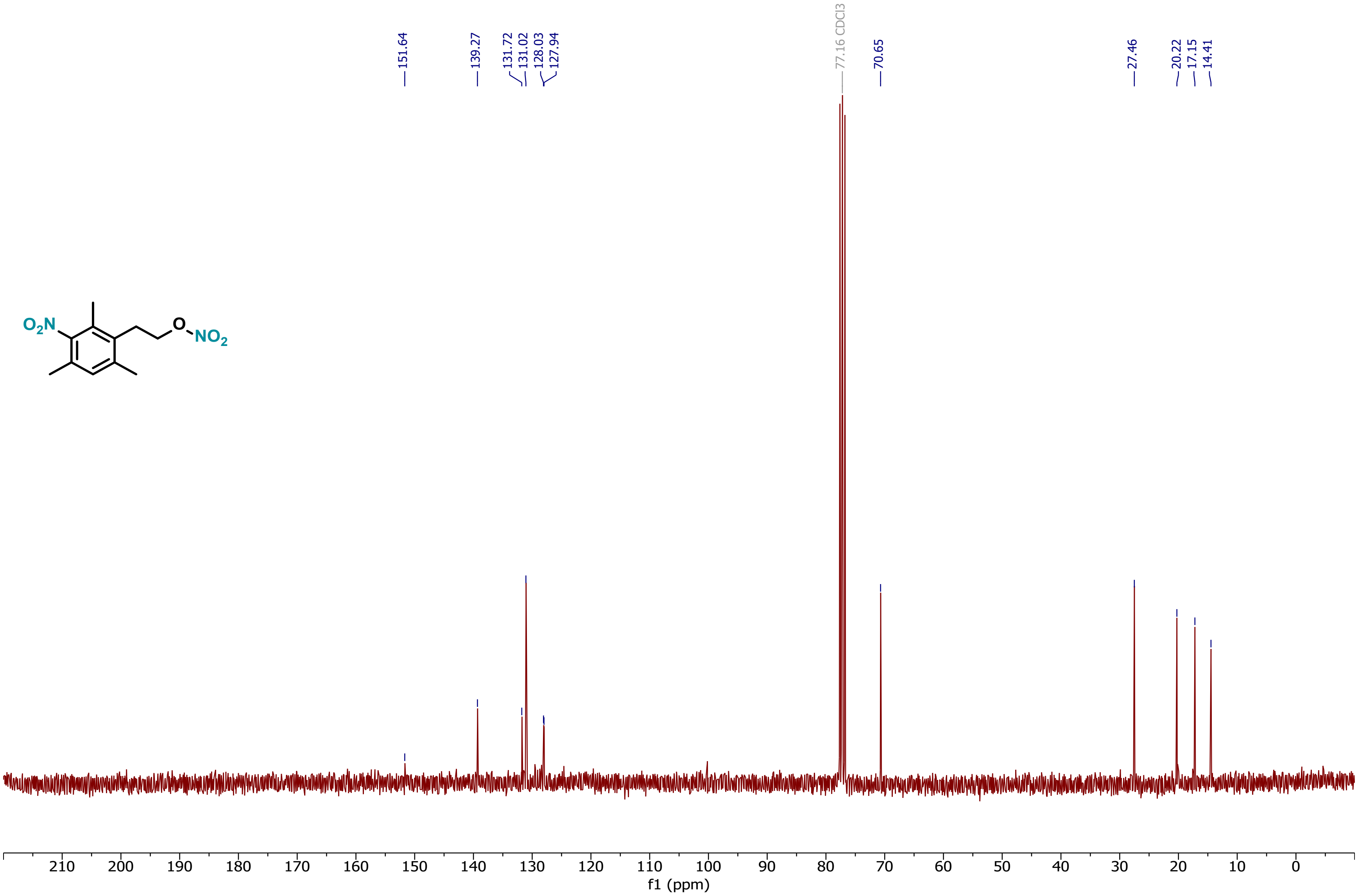
151.64
139.27
131.72
131.02
128.03
127.94
77.16 CDCl3
70.65
27.46
20.22
17.15
14.41
210
200
190
180
170
160
150
140
130
120
110
100
90
80
70
60
50
40
30
20
10
0
f1 (ppm)

4-fluorophenethyl nitrate (6)

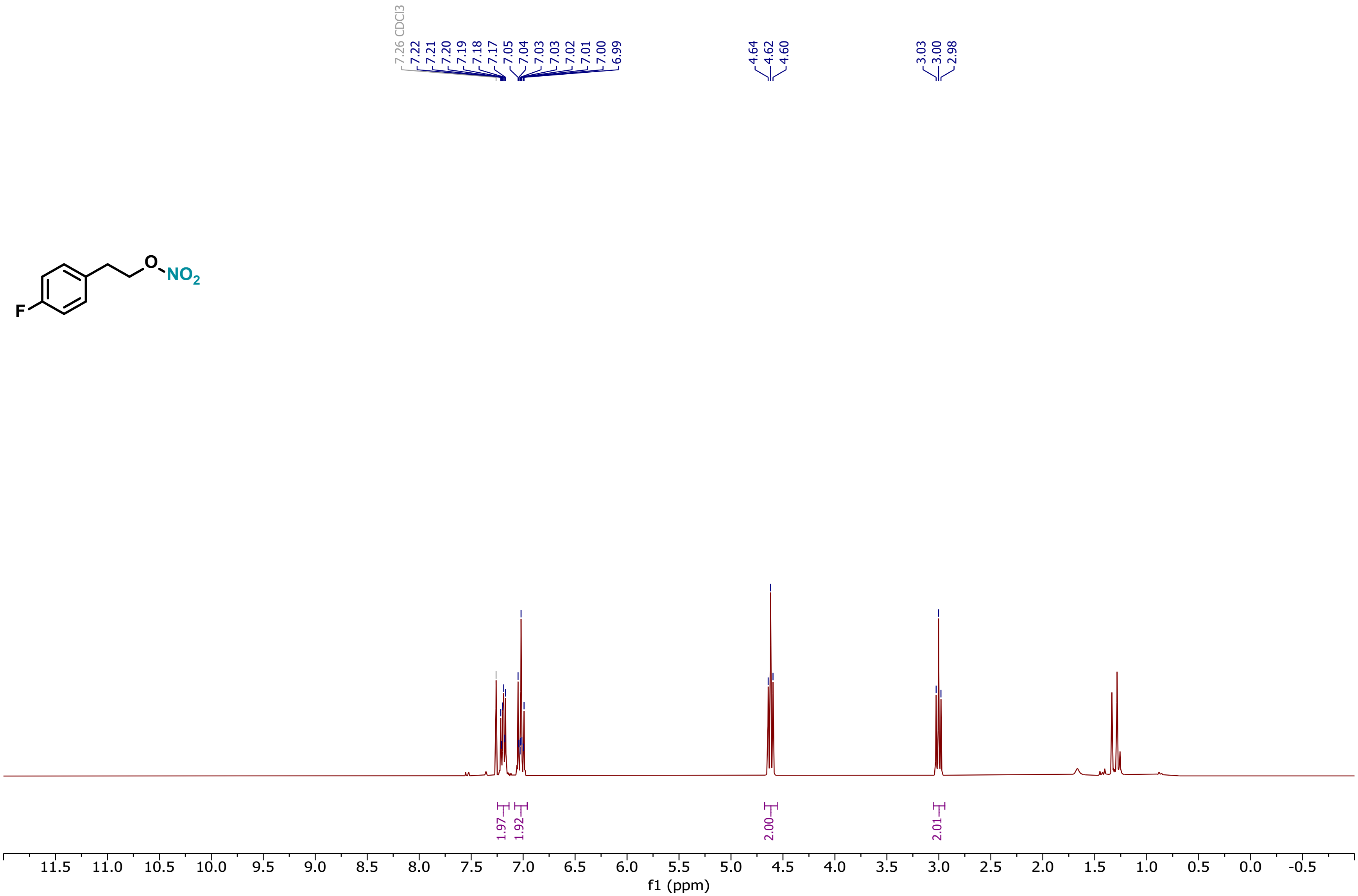

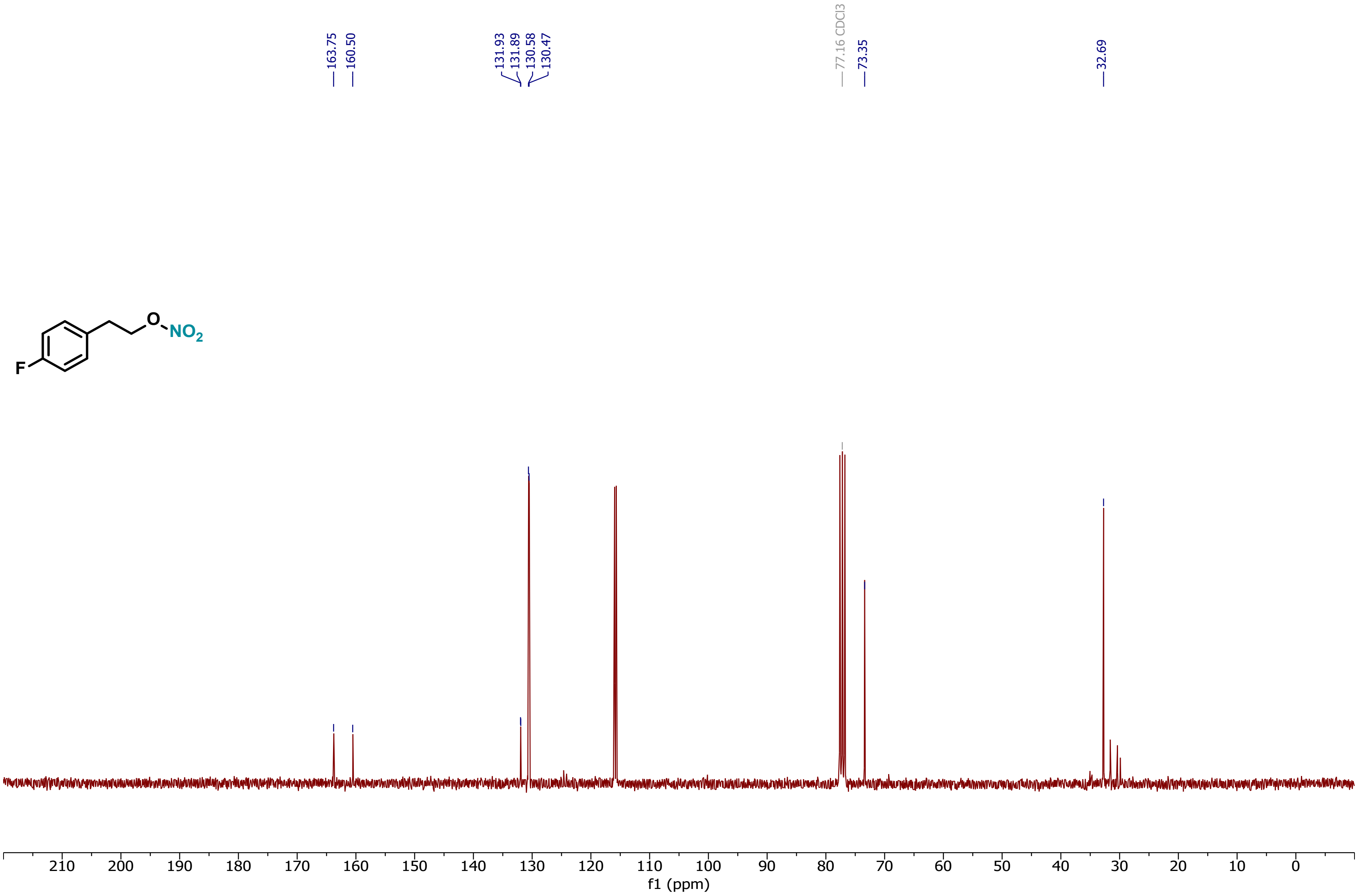

163.75
160.50
131.93
131.89
130.58
130.47
77.16 CDCl3
73.35
32.69
210
200
190
180
170
160
150
140
130
120
110
100
90
80
70
60
50
40
30
20
10
0
f1 (ppm)

3-fluorophenethyl nitrate (7)

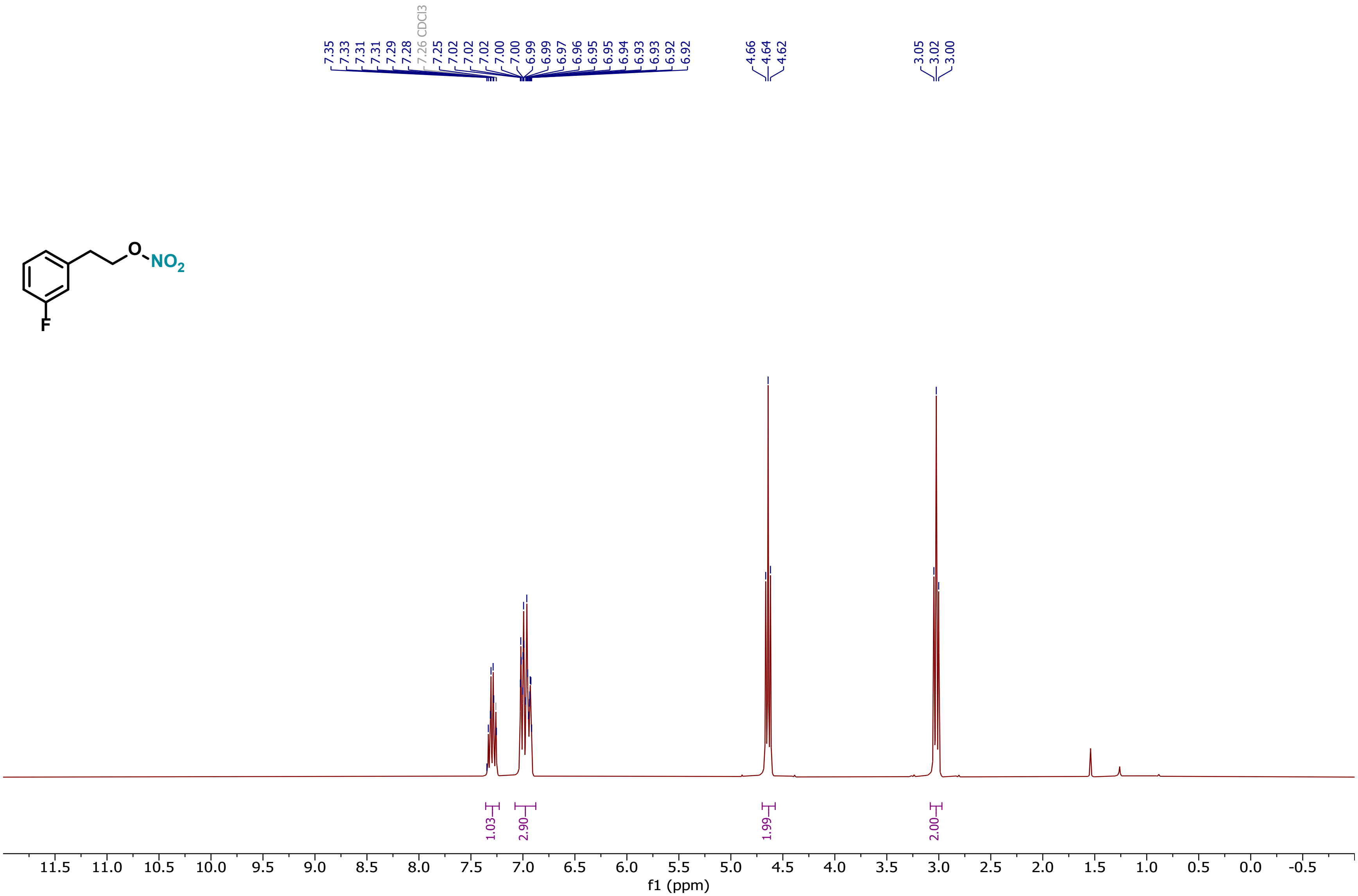

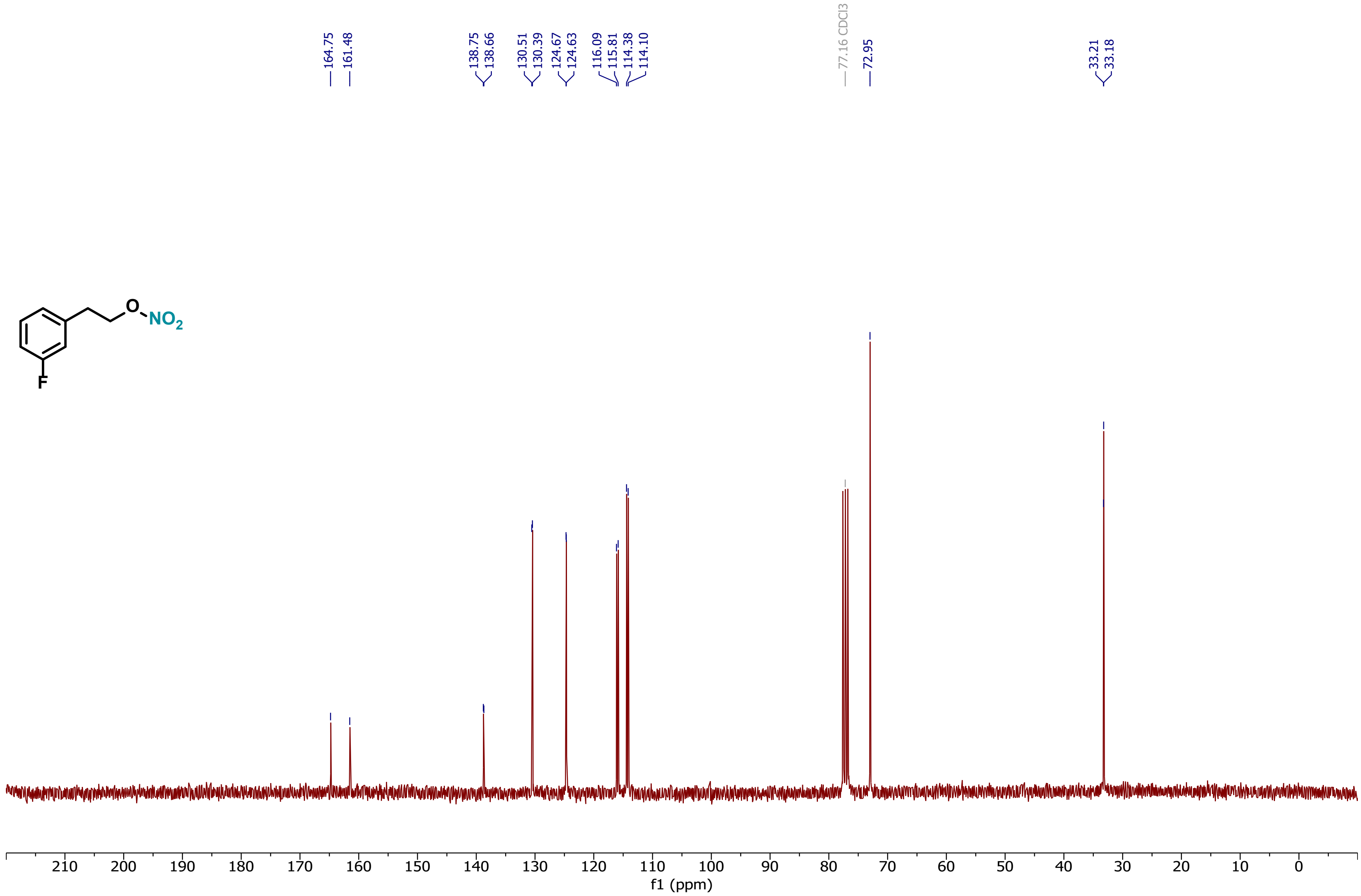

164.75
161.48
138.75
138.66
130.51
130.39
124.67
124.63
116.09
115.81
114.38
114.10
77.16 CDCl3
72.95
33.21
33.18
O
NO2
F
210 200 190 180 170 160 150 140 130 120 110 100 90 80 70 60 50 40 30 20 10 0
f1 (ppm)

2-nitro-2-phenylethyl nitrate (8)

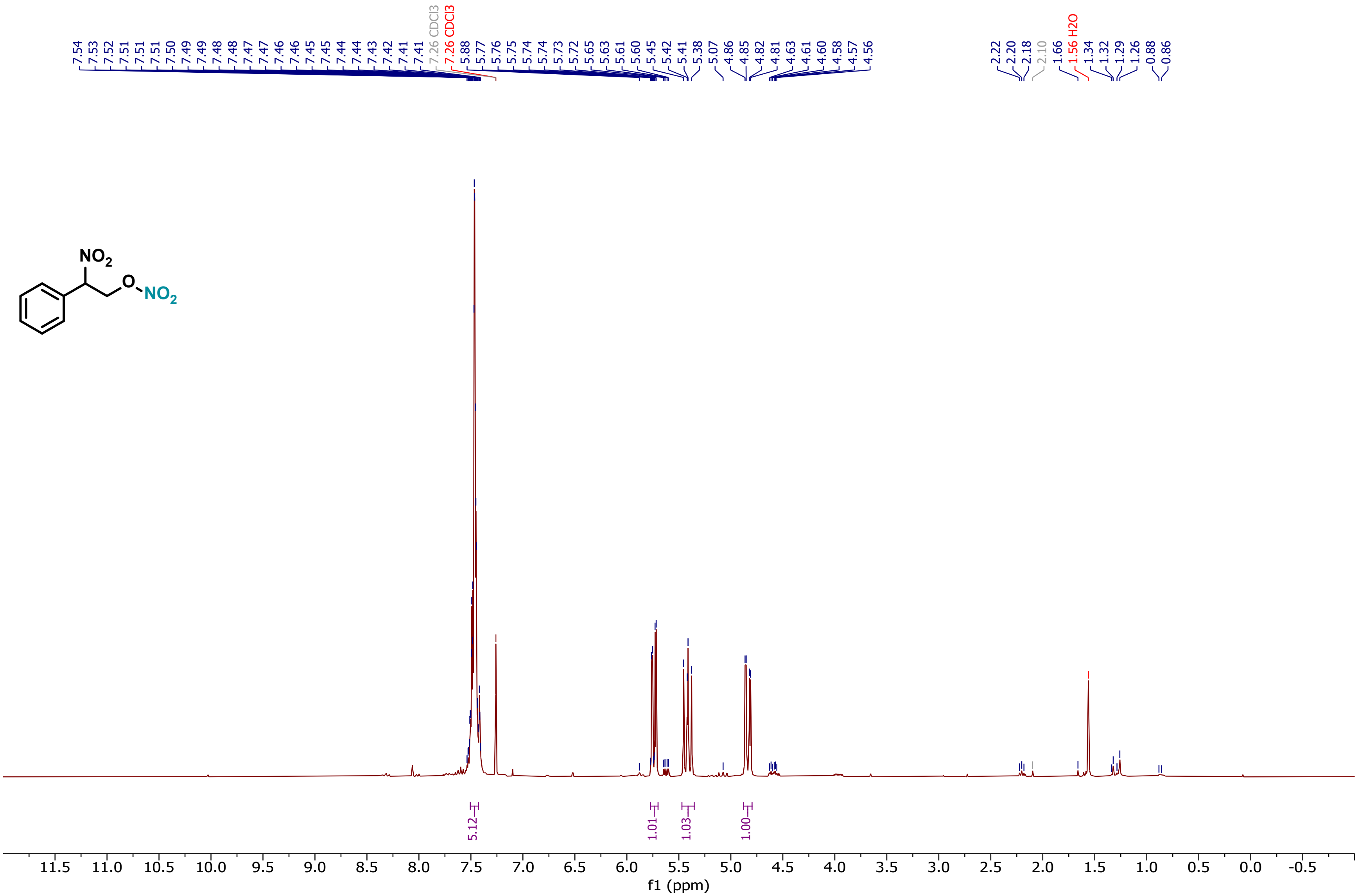

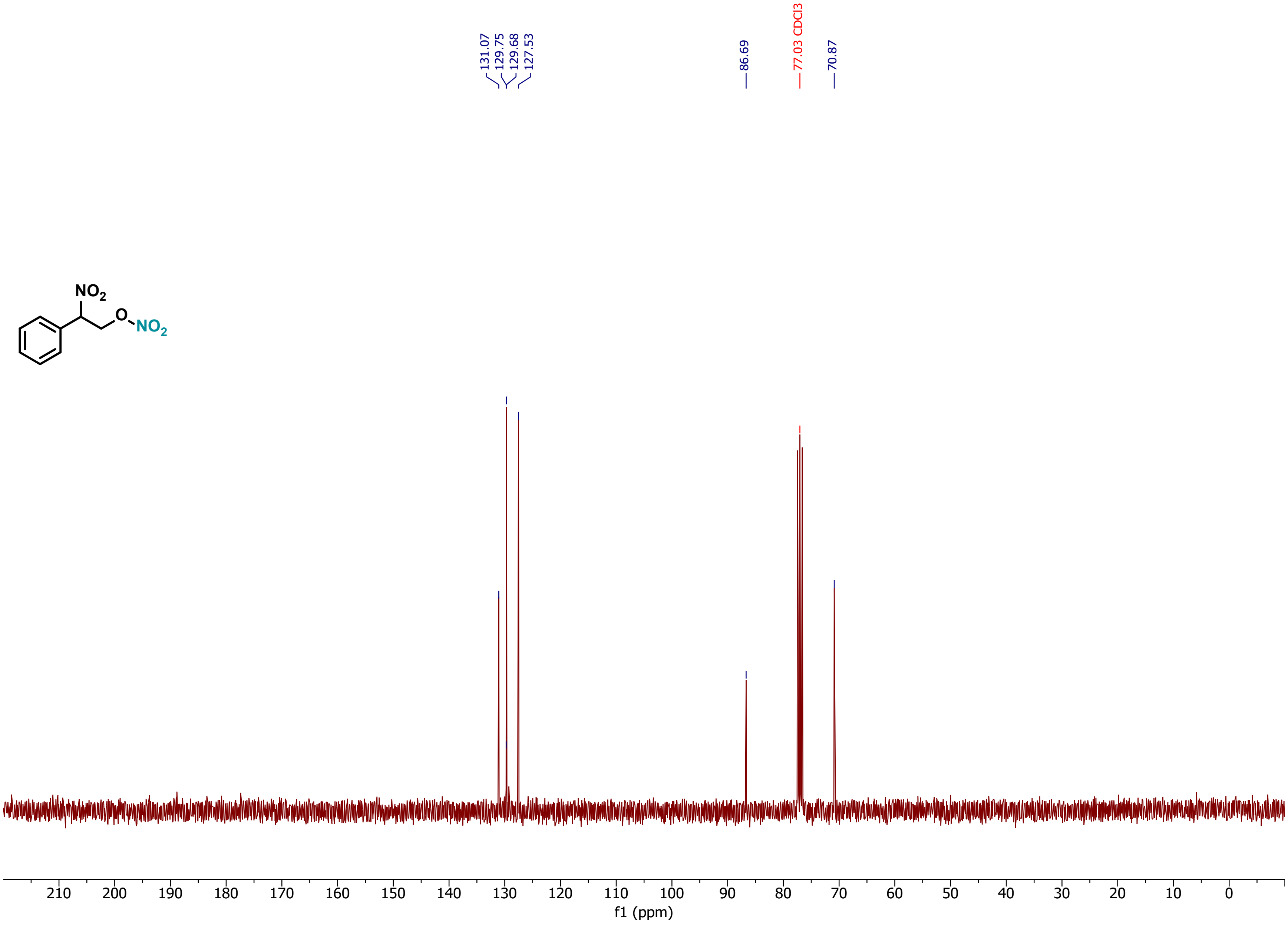

NO2
O
NO2
131.07
129.75
129.68
127.53
86.69
77.03 CDCl3
70.87
210
200
190
180
170
160
150
140
130
120
110
100
90
80
70
60
50
40
30
20
10
0
f1 (ppm)

2-bromo-2-phenylethyl nitrate (9)

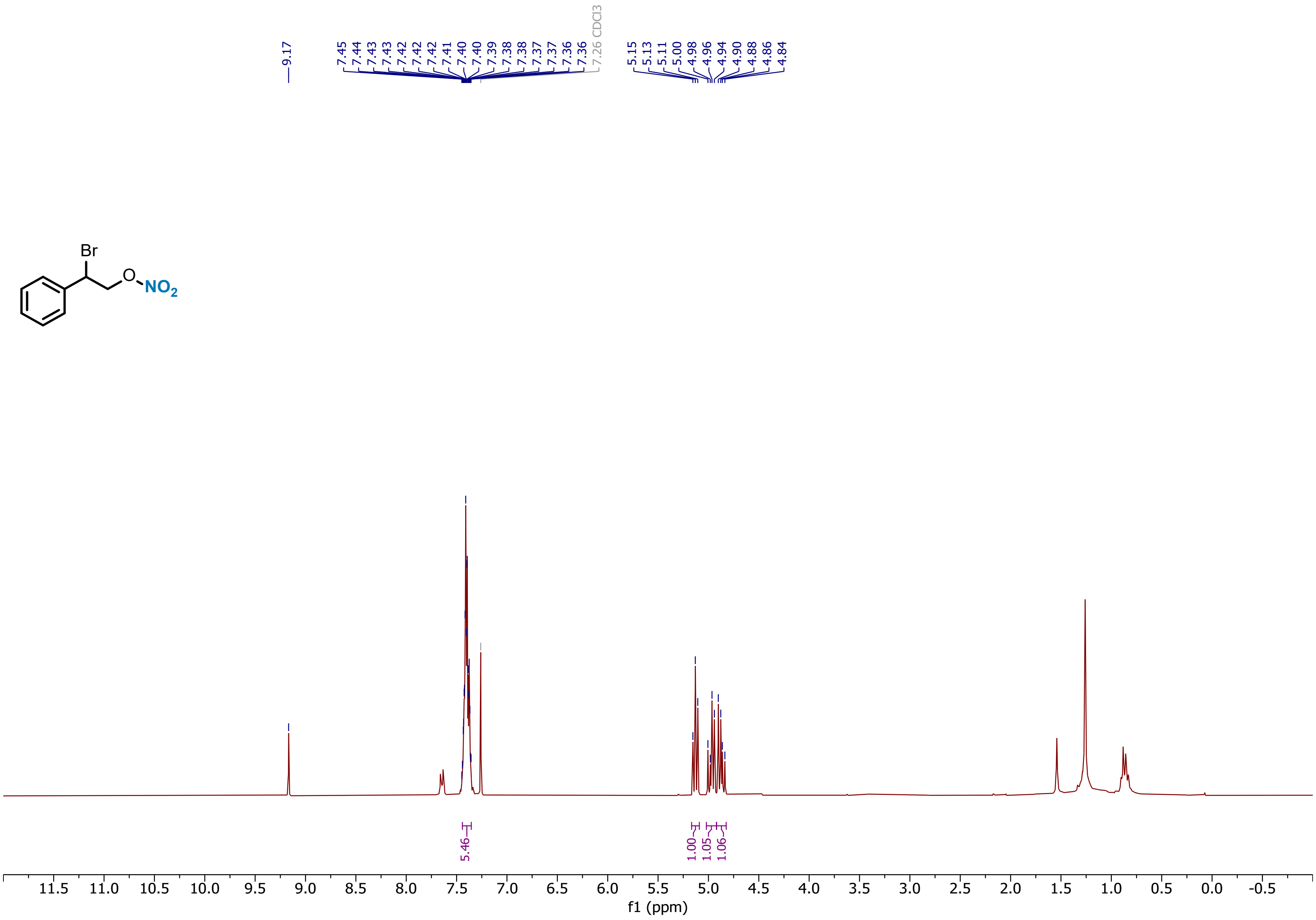

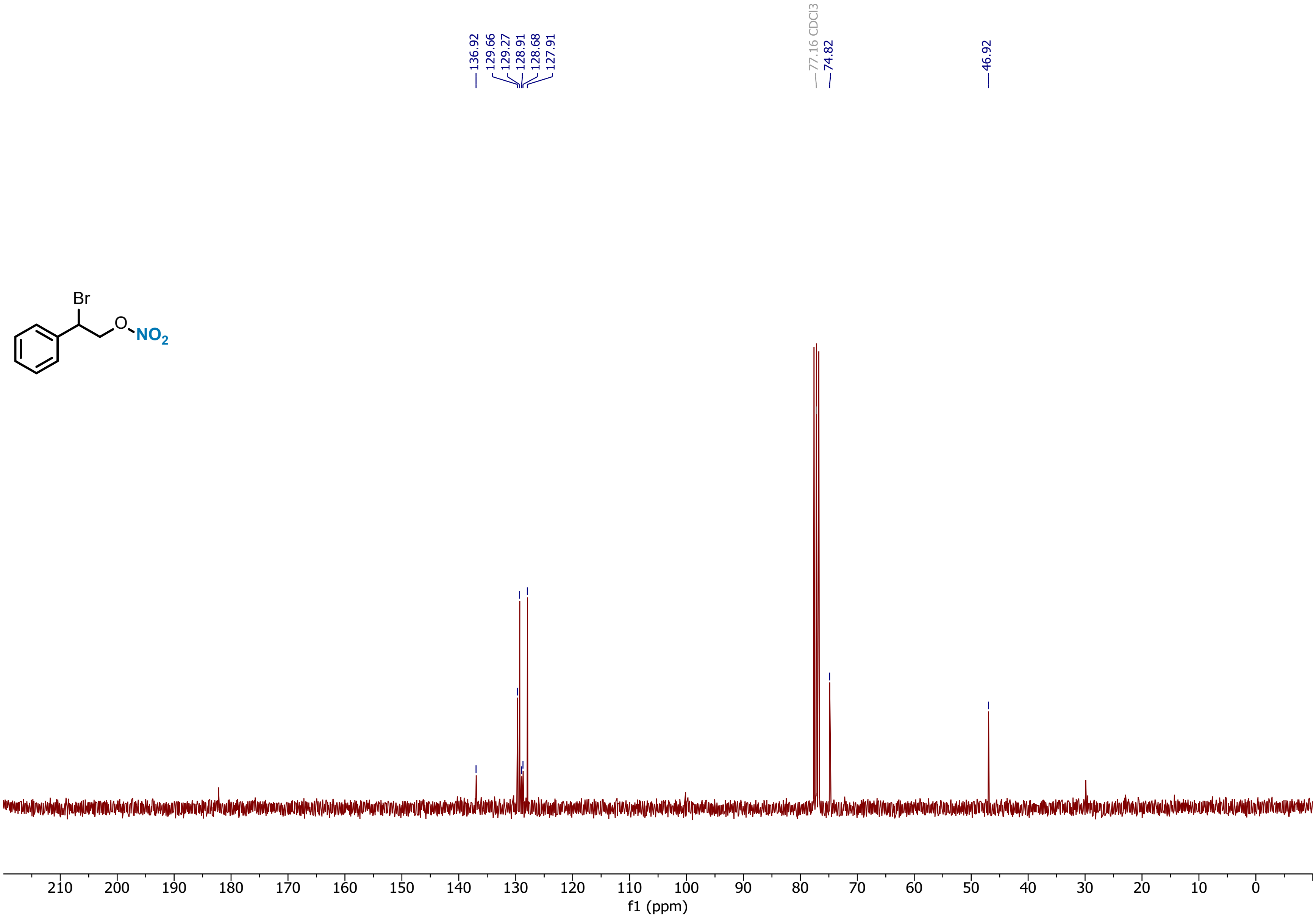

136.92
129.66
129.27
128.91
128.68
127.91
77.16 CDCl3
74.82
46.92
Br
O
NO2
210
200
190
180
170
160
150
140
130
120
110
100
90
80
70
60
50
40
30
20
10
0
f1 (ppm)

2-azido-2-phenylethyl nitrate (10)

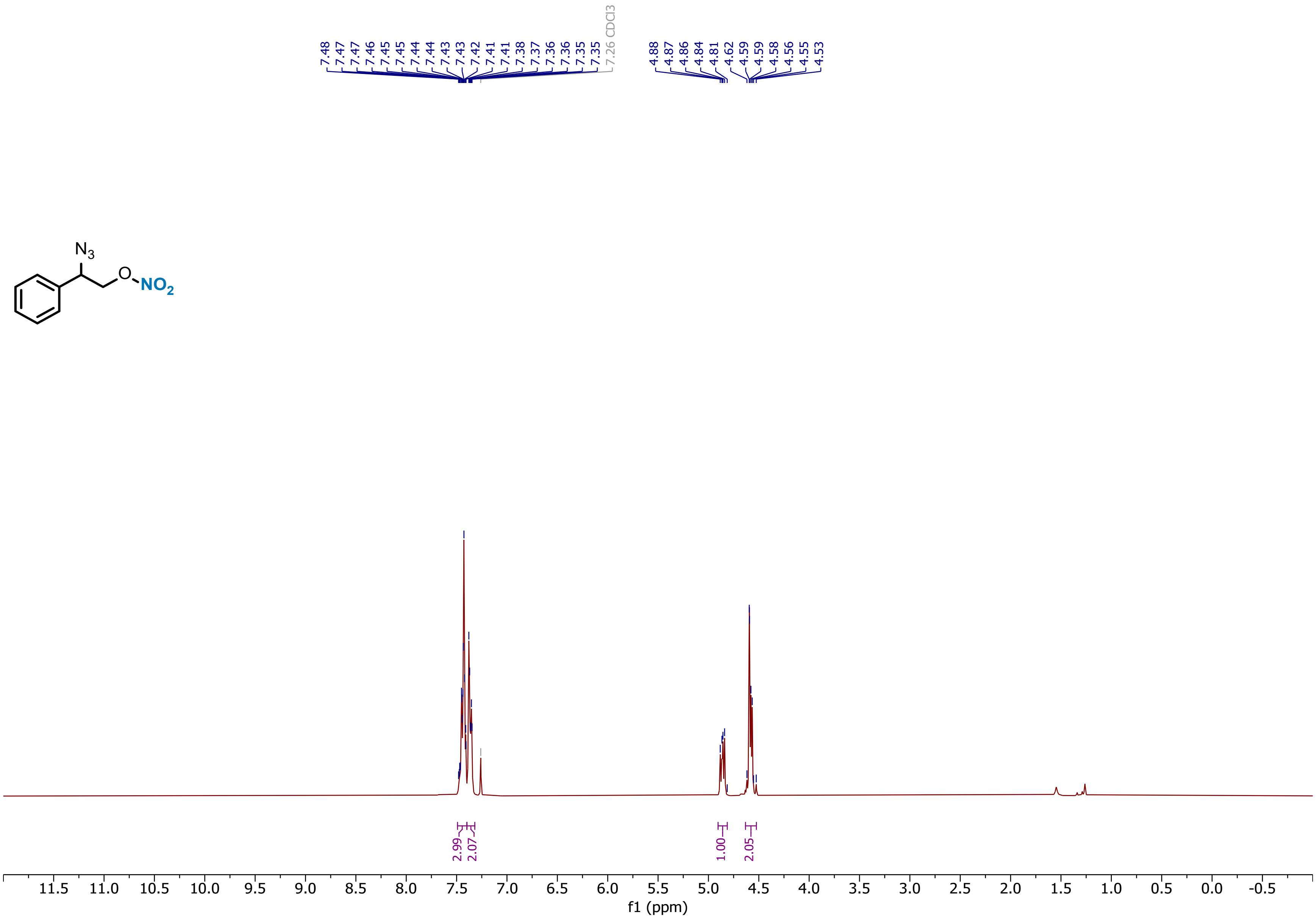

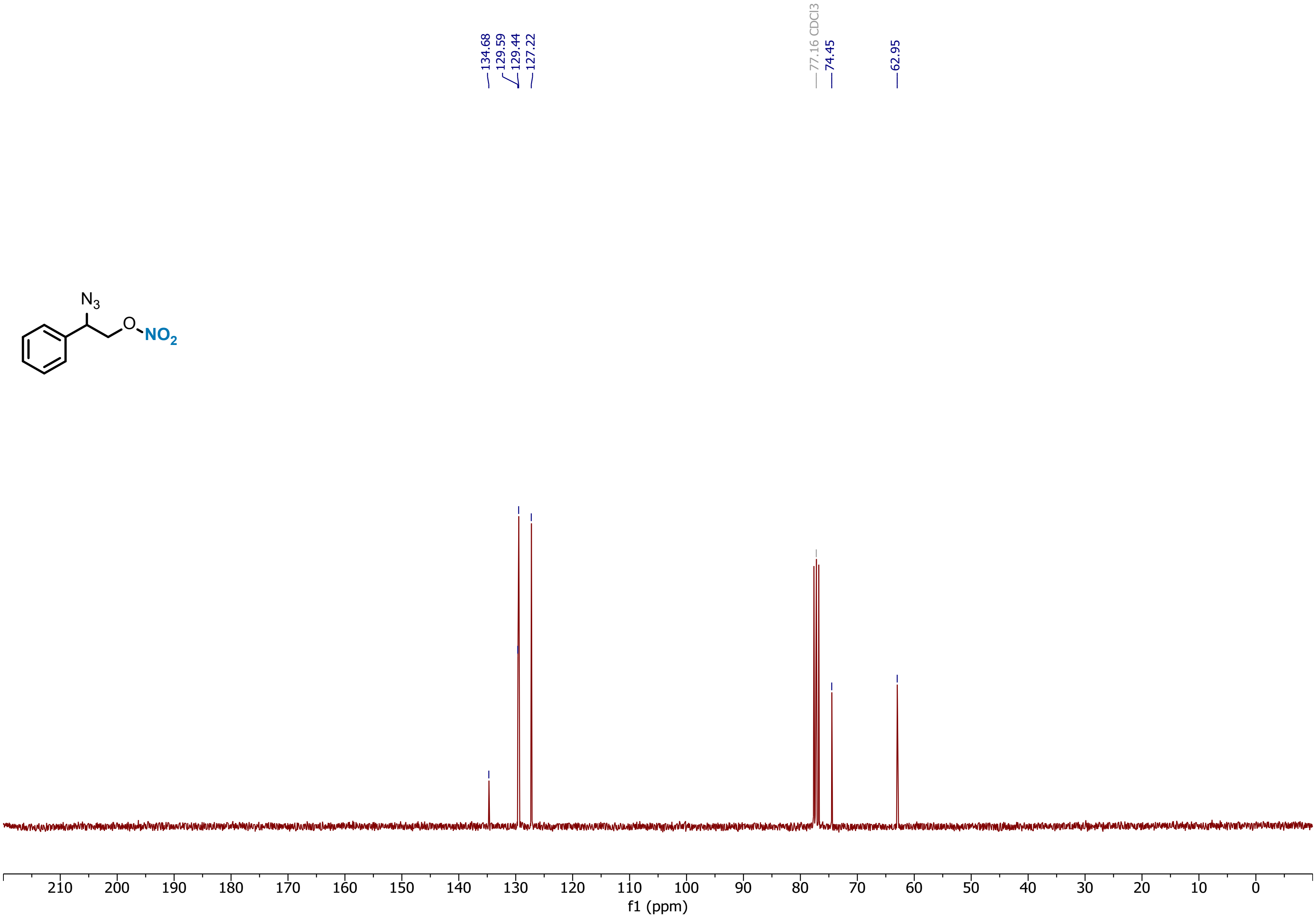
134.68
129.59
129.44
127.22
77.16 CDCl3
74.45
62.95
N3
O
NO2
210
200
190
180
170
160
150
140
130
120
110
100
90
80
70
60
50
40
30
20
10
0
f1 (ppm)

6-chlorohexyl nitrate (11)

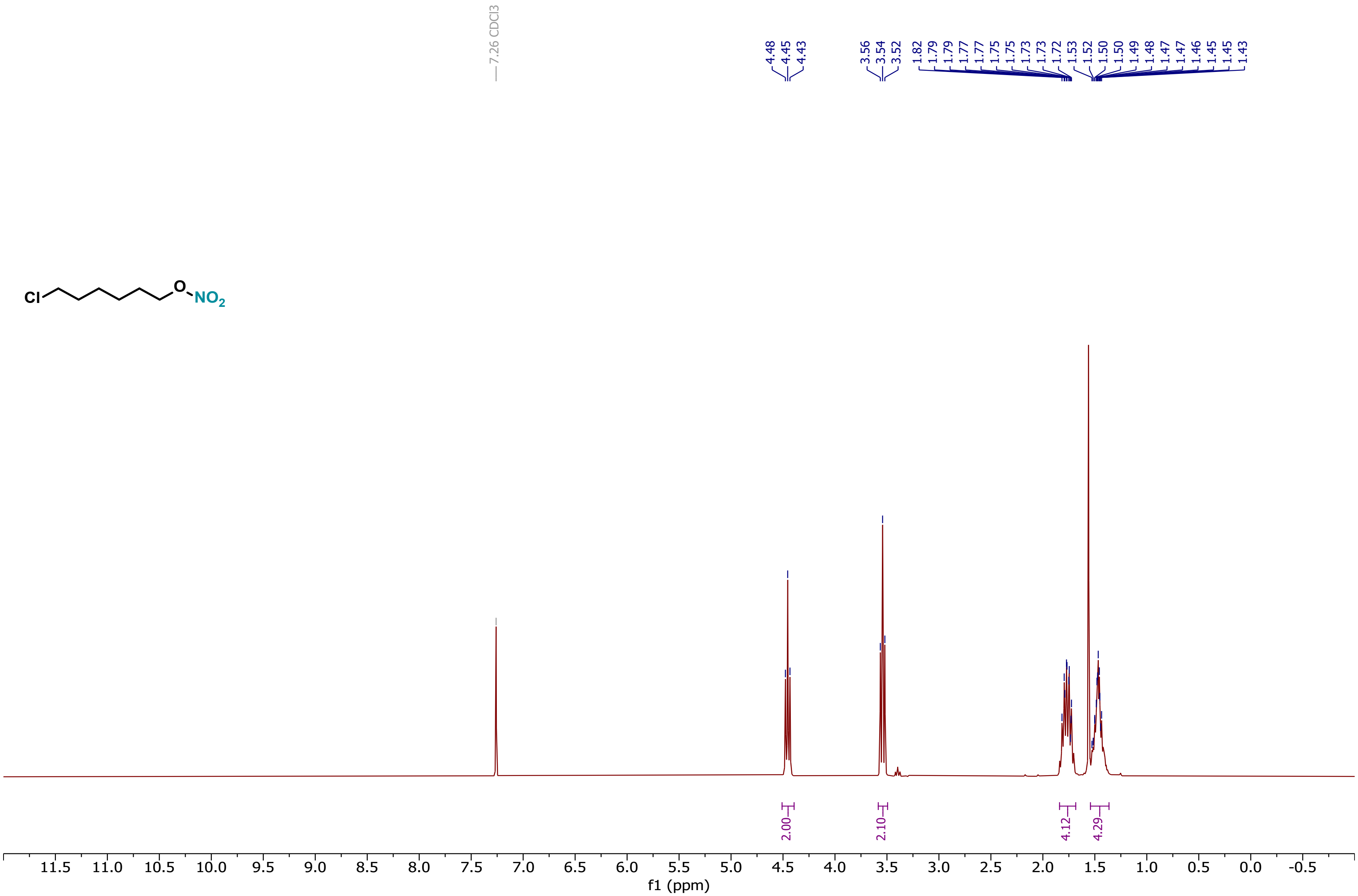

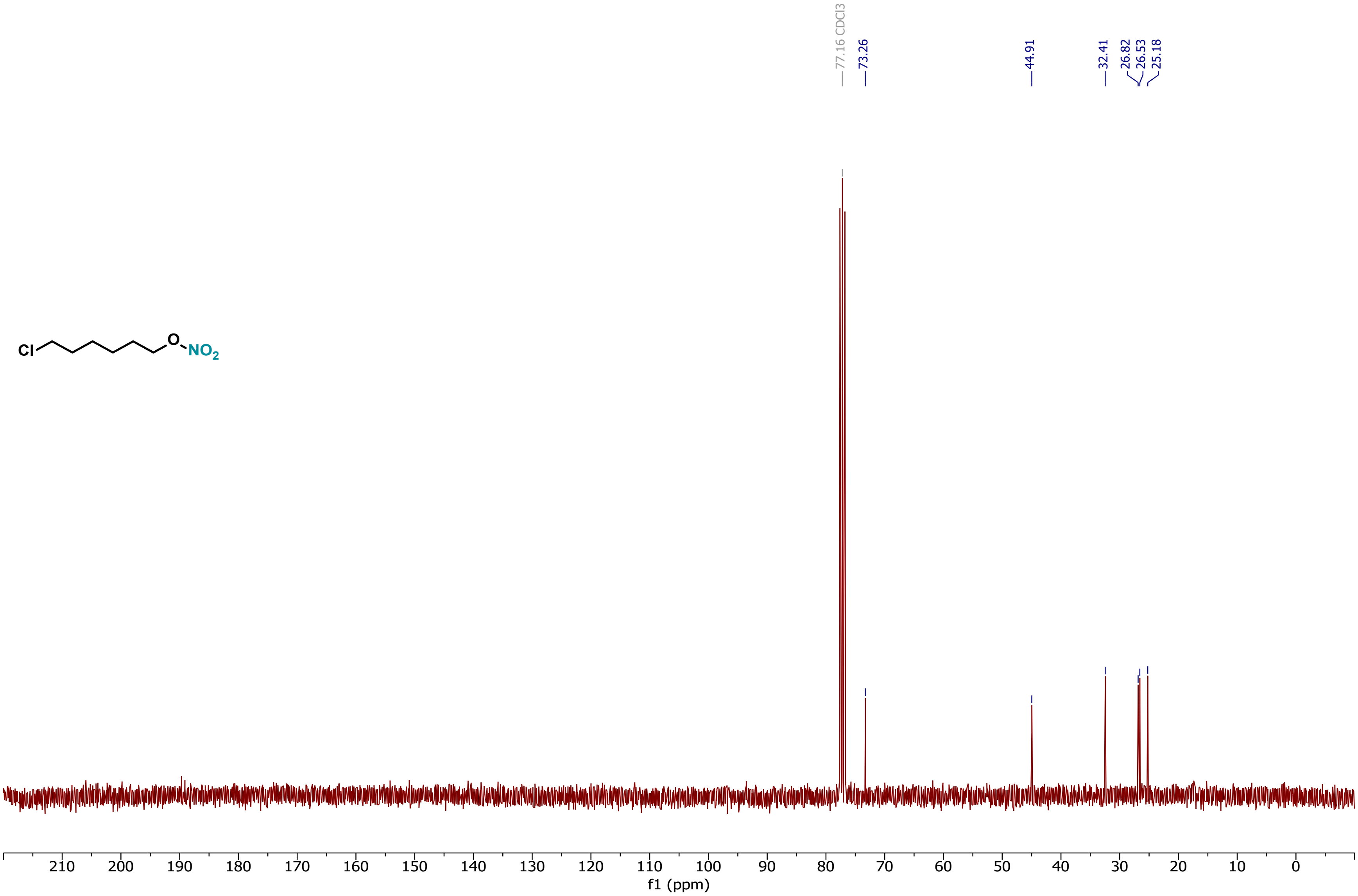
77.16 CDCl3
73.26
44.91
32.41
26.82
26.53
25.18
Cl
O
NO2
210 200 190 180 170 160 150 140 130 120 110 100 90 80 70 60 50 40 30 20 10 0
f1 (ppm)

oct-2-yn-1-yl nitrate (12)

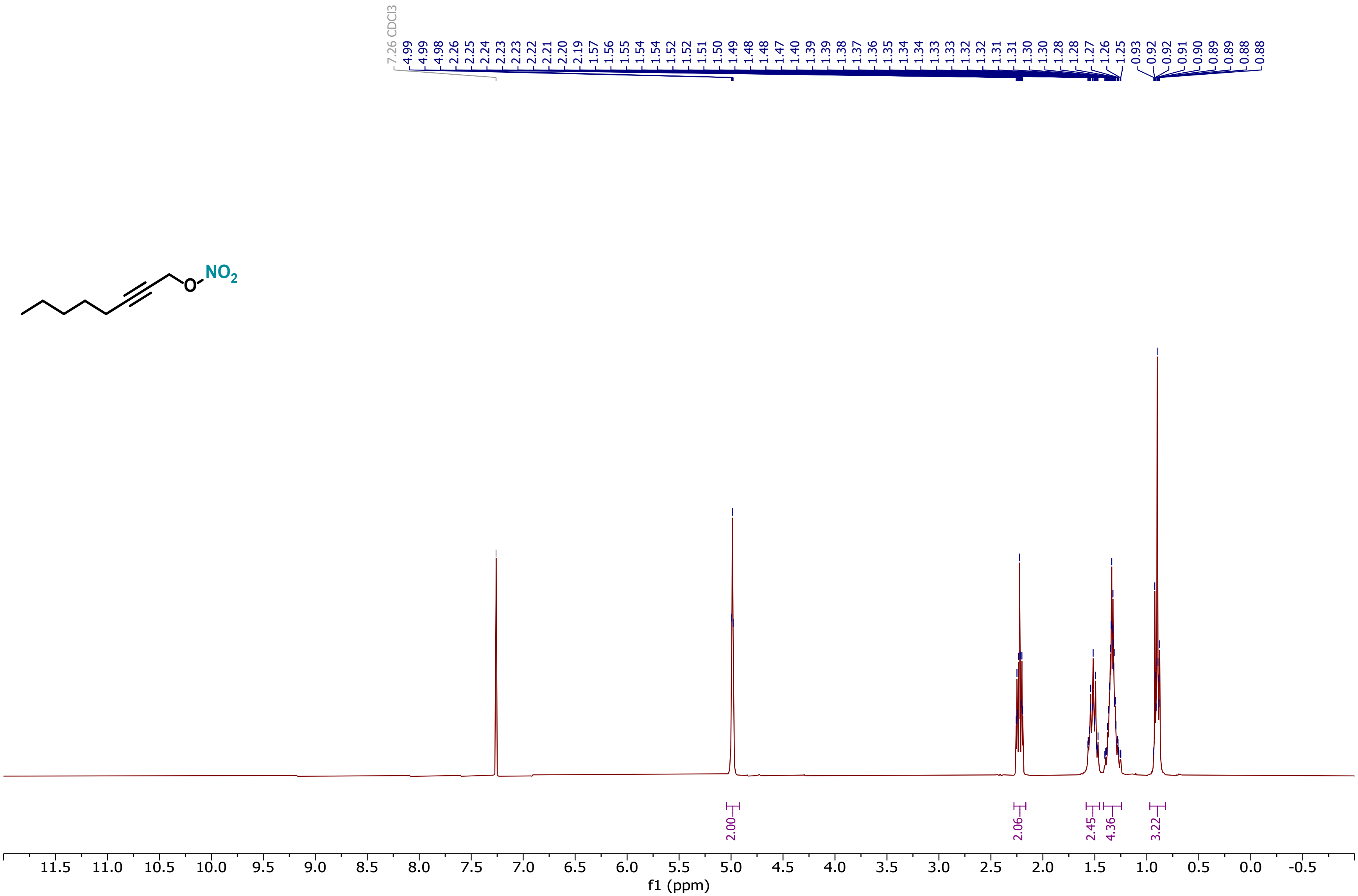

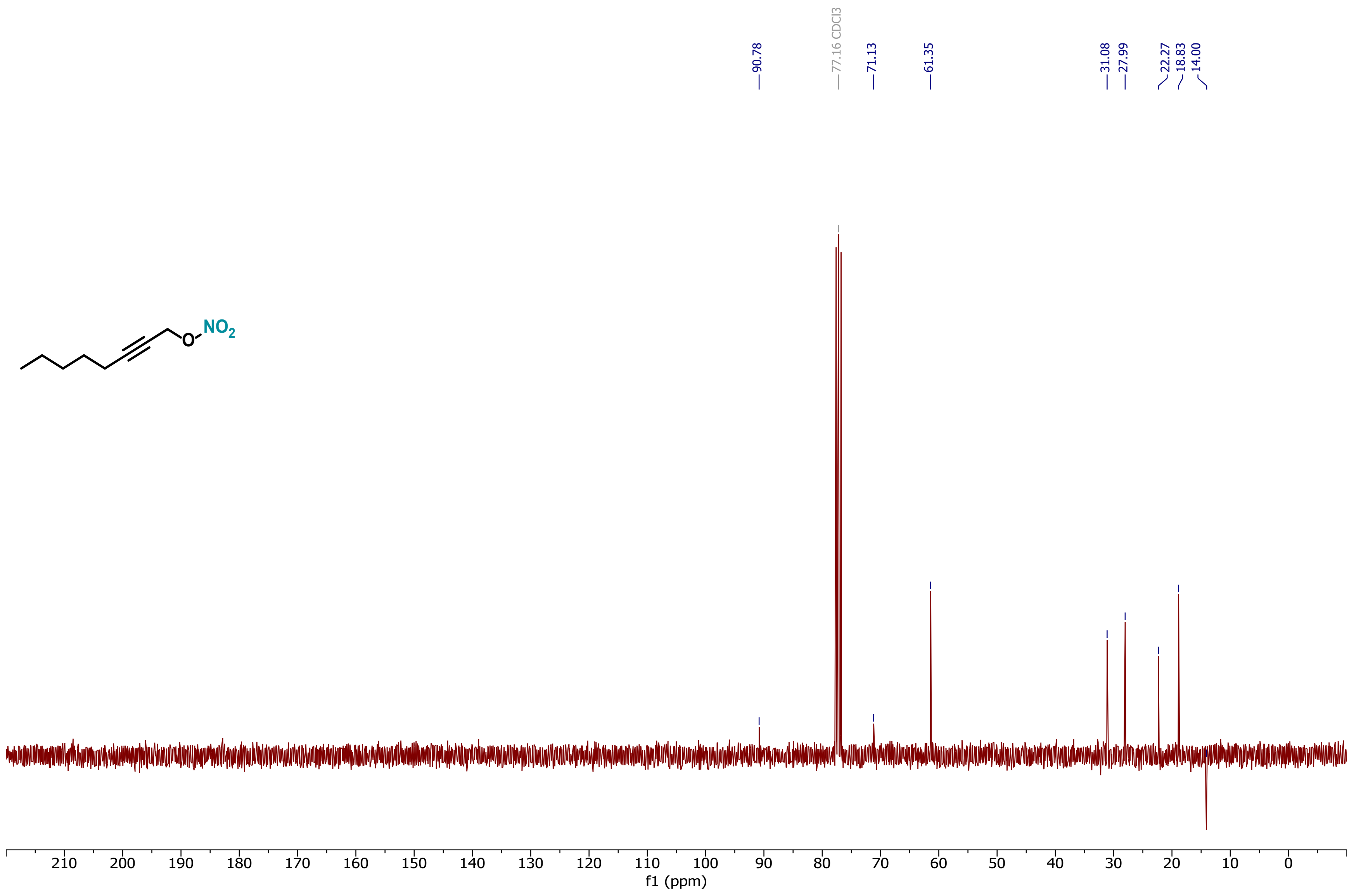

90.78
77.16 CDCl3
71.13
61.35
31.08
27.99
22.27
18.83
14.00
NO2
O
210
200
190
180
170
160
150
140
130
120
110
100
90
80
70
60
50
40
30
20
10
0
f1 (ppm)

dec-9-en-1-yl nitrate (13)

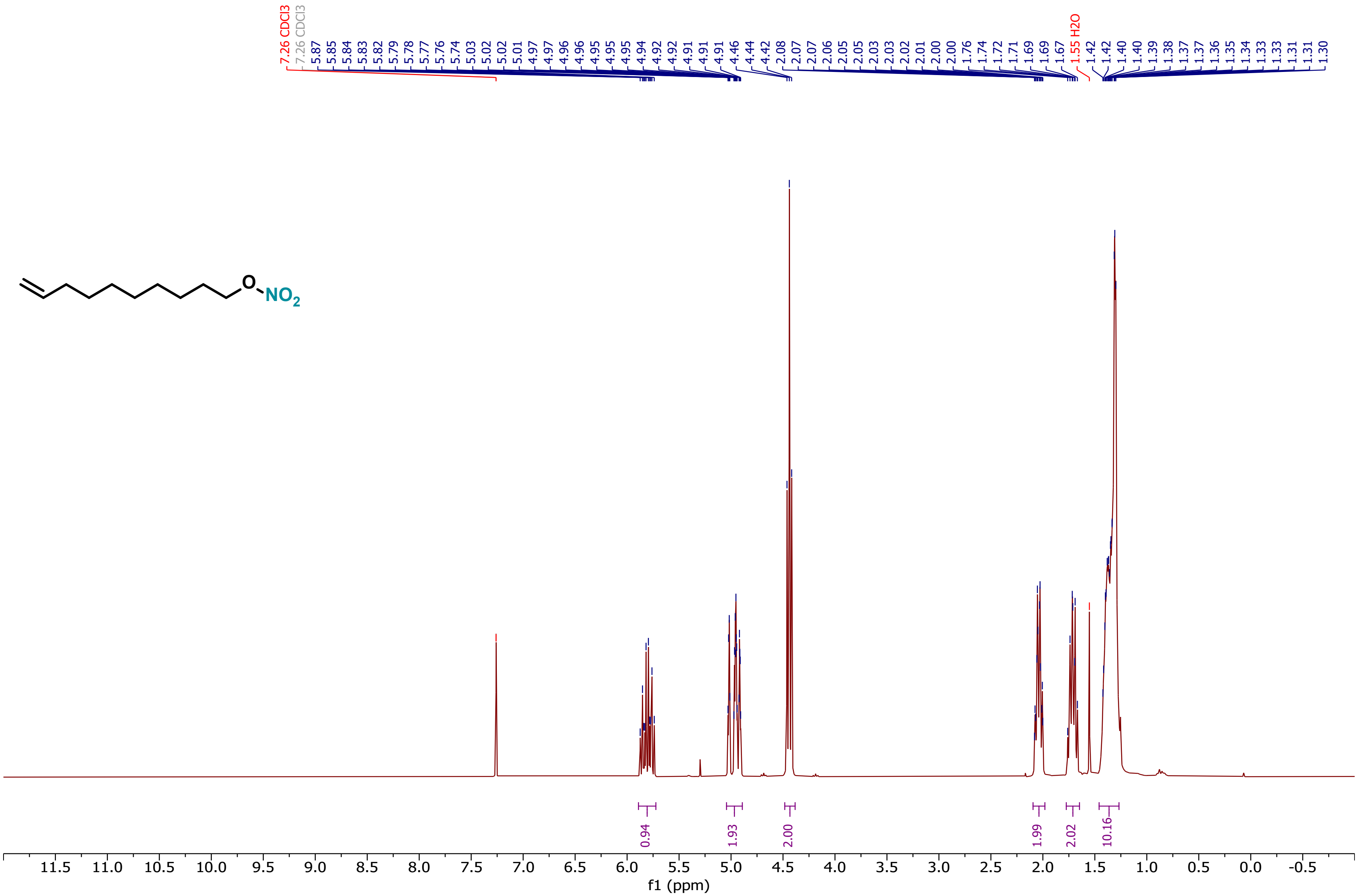

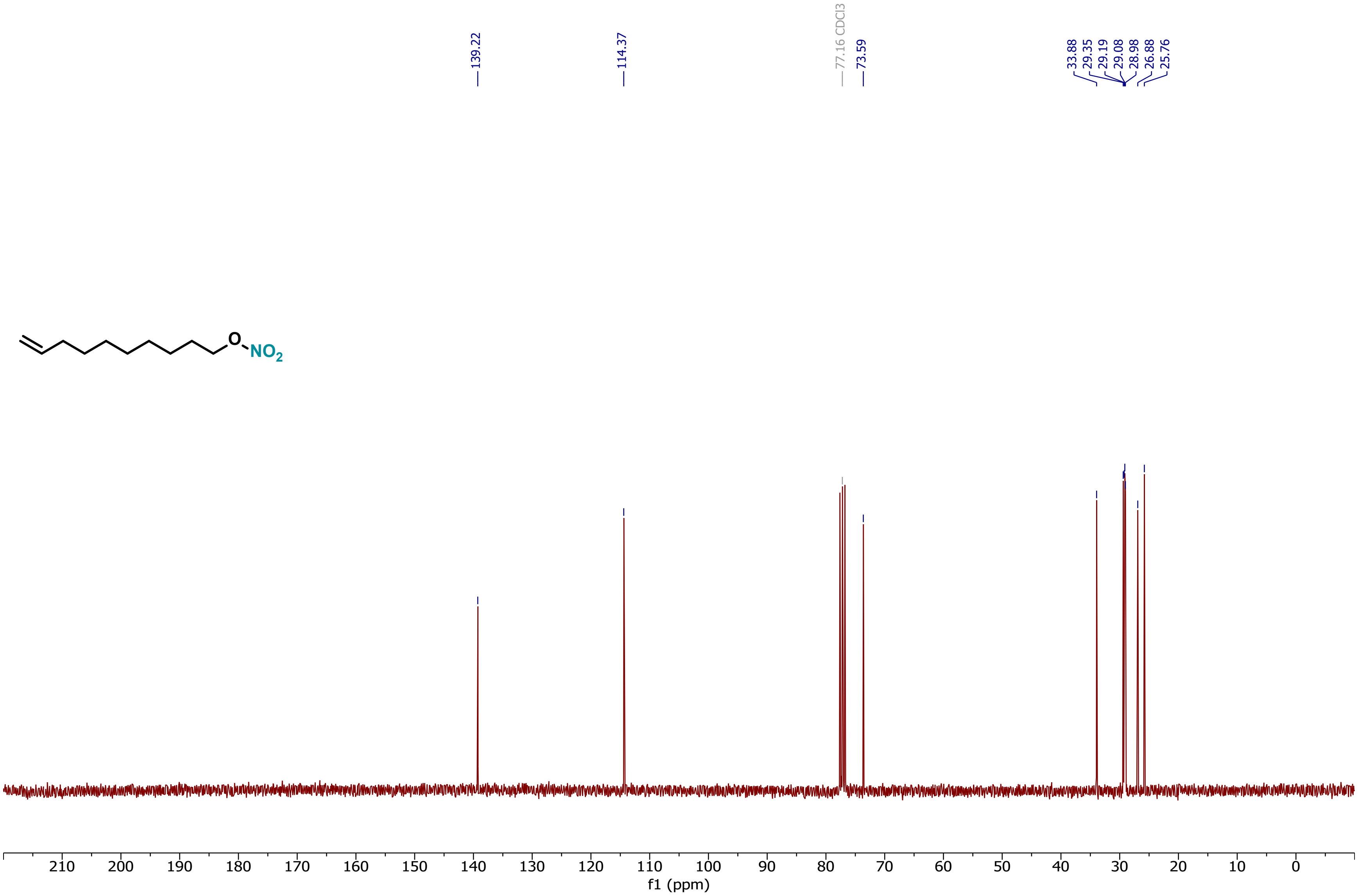
139.22
114.37
77.16 CDCl3
73.59
33.88
29.35
29.19
29.08
28.98
26.88
25.76
O
NO2
210 200 190 180 170 160 150 140 130 120 110 100 90 80 70 60 50 40 30 20 10 0
f1 (ppm)

undec-10-en-1-yl nitrate (14)

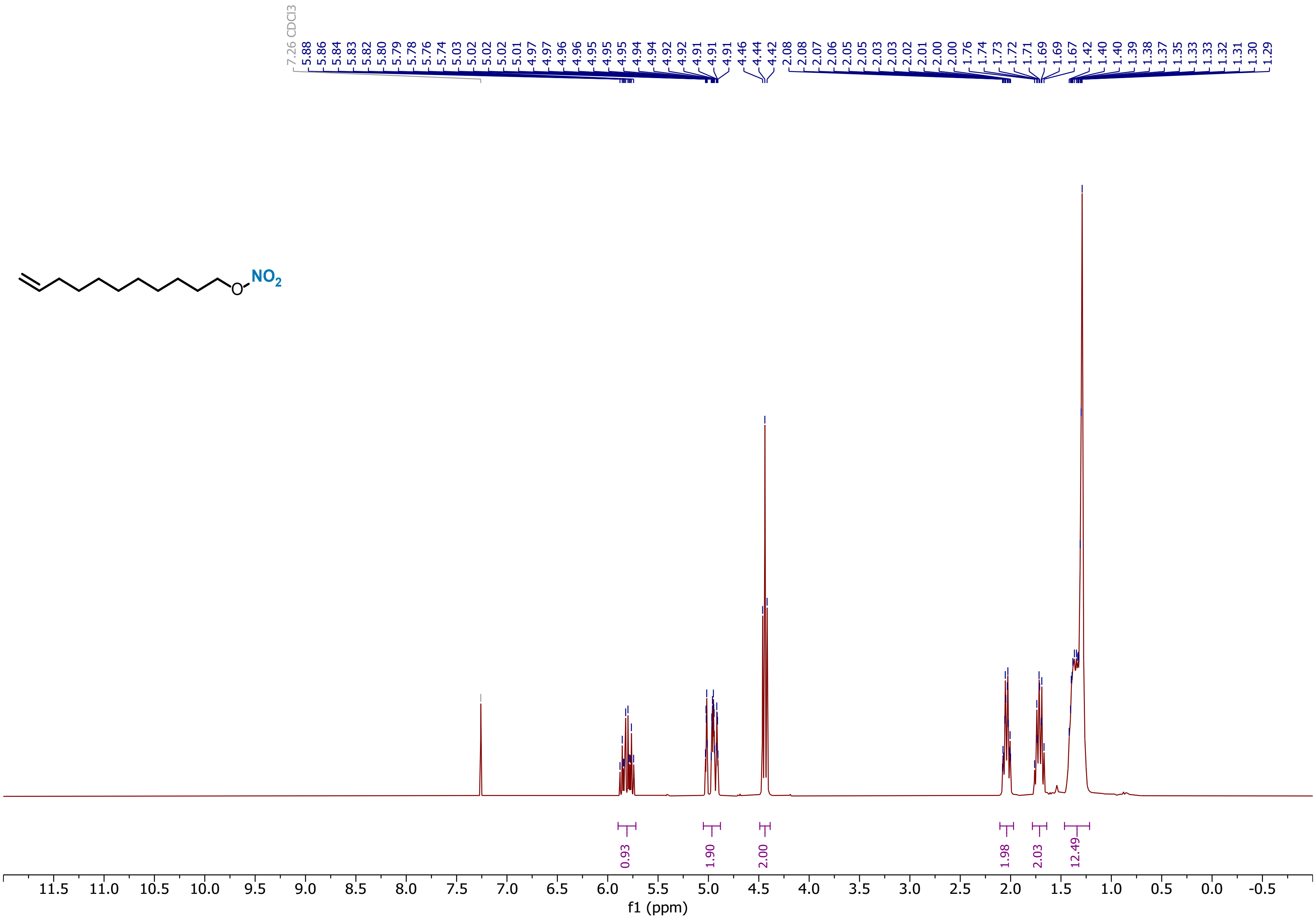

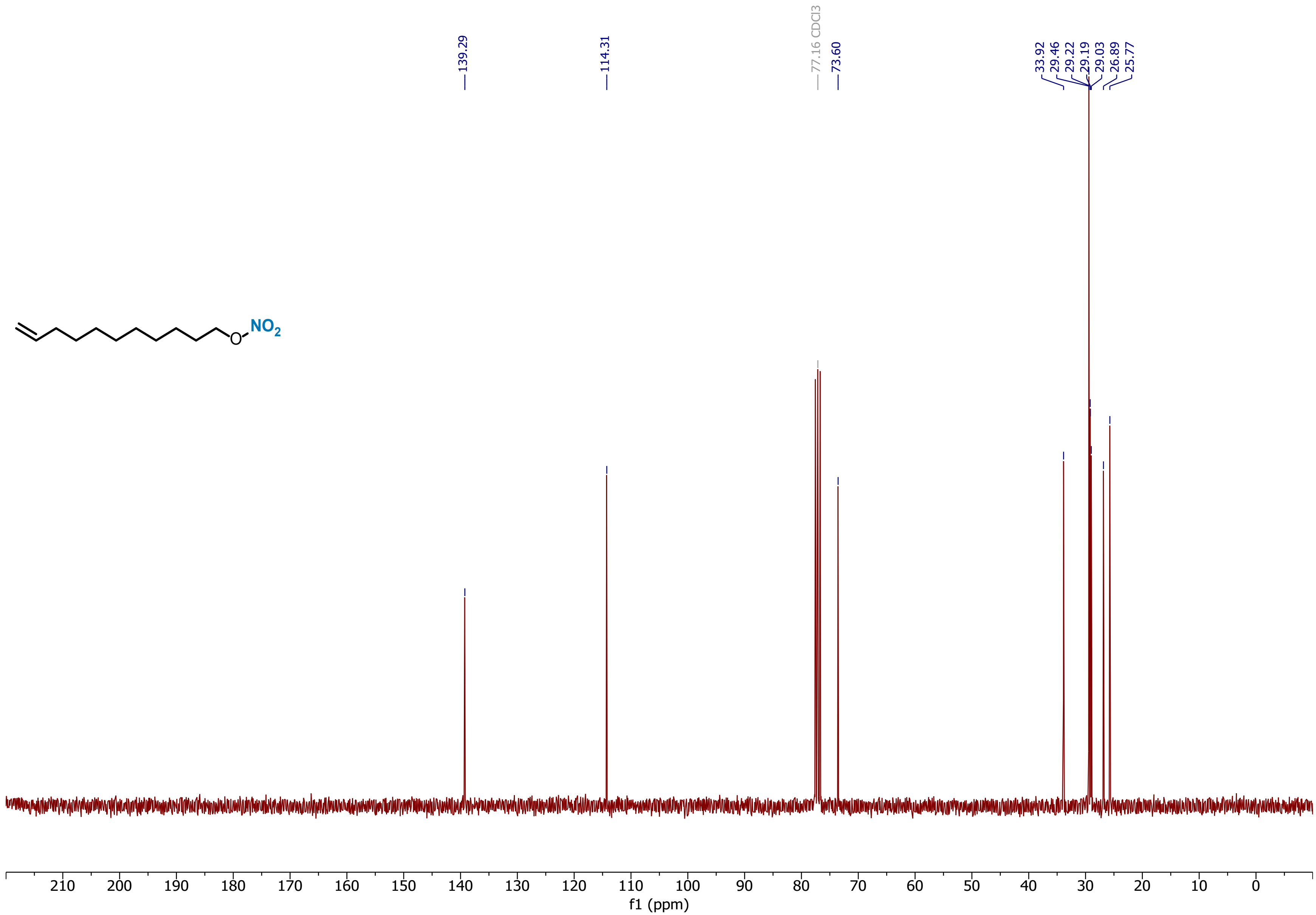
139.29
114.31
77.16 CDCl3
73.60
33.92
29.46
29.22
29.19
29.03
26.89
25.77
210
200
190
180
170
160
150
140
130
120
110
100
90
80
70
60
50
40
30
20
10
0
f1 (ppm)

4-phenylbutan-2-yl nitrate (15)

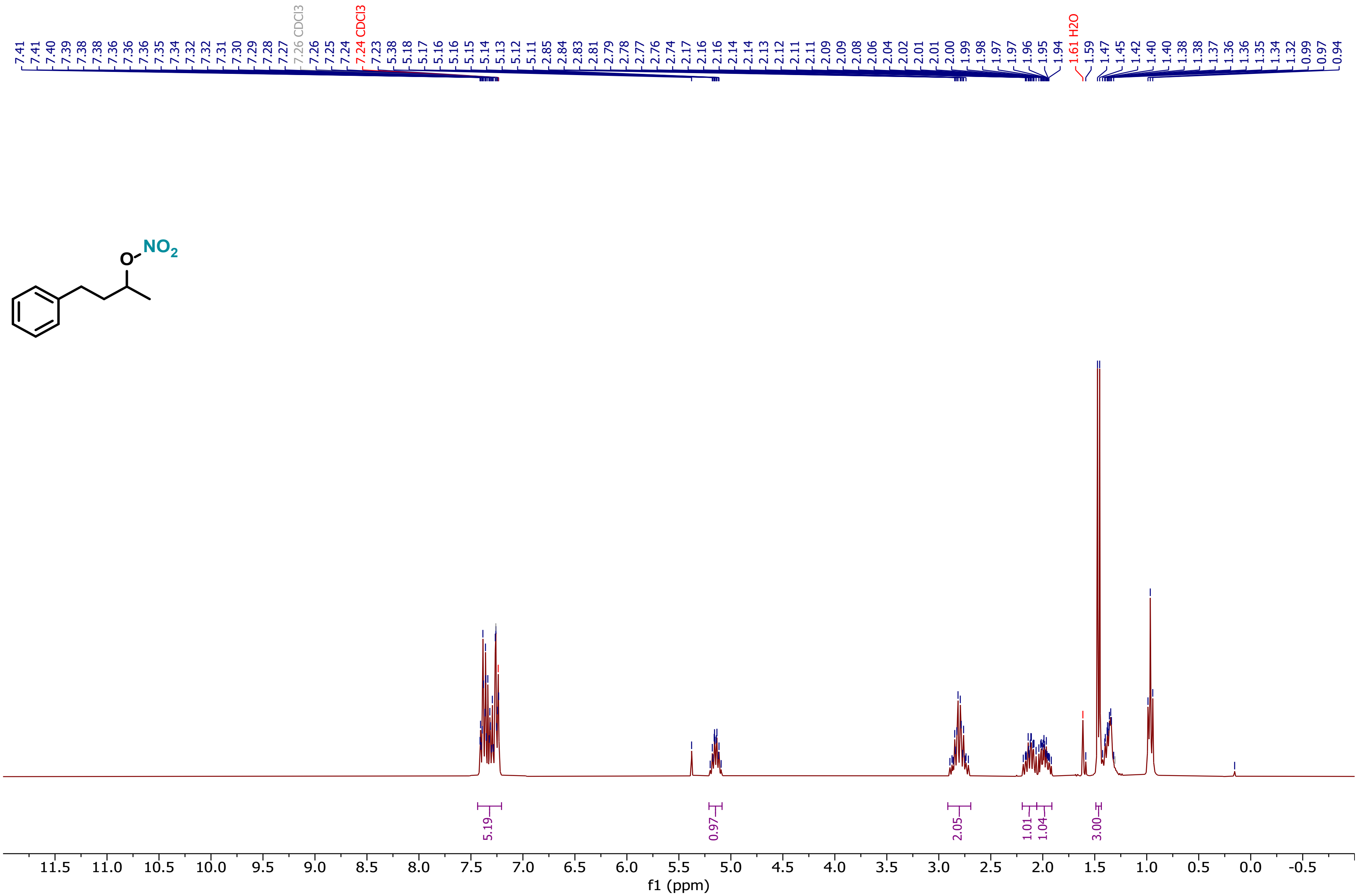

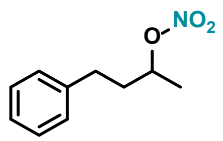

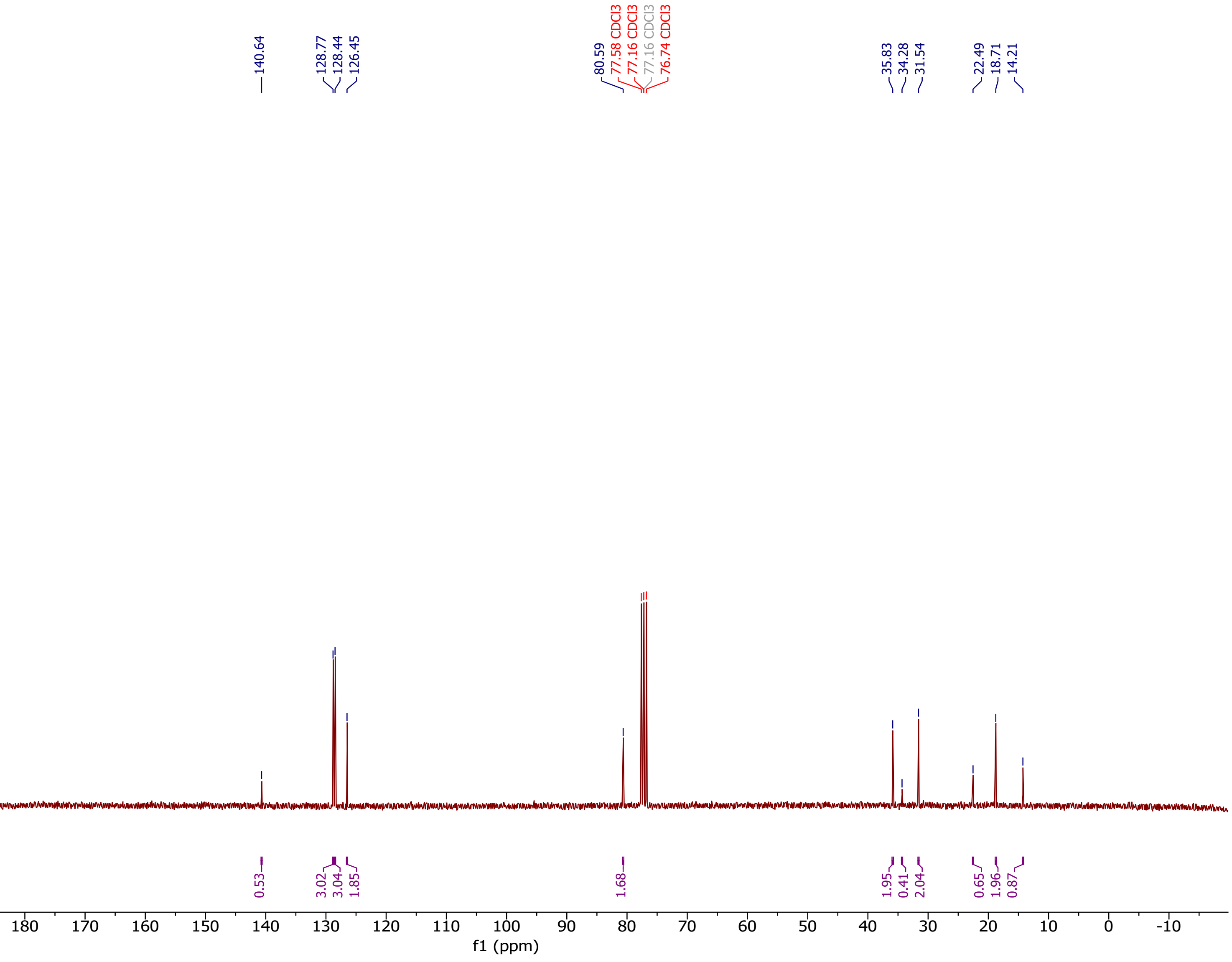
140.64
128.77
128.44
126.45
80.59
77.58 CDCl3
77.16 CDCl3
77.16 CDCl3
76.74 CDCl3
35.83
34.28
31.54
22.49
18.71
14.21
0.53
3.02
3.04
1.85
1.68
1.95
0.41
2.04
0.65
1.96
0.87
f1 (ppm)

bicyclo[2.2.1]heptan-2-yl nitrate (16)

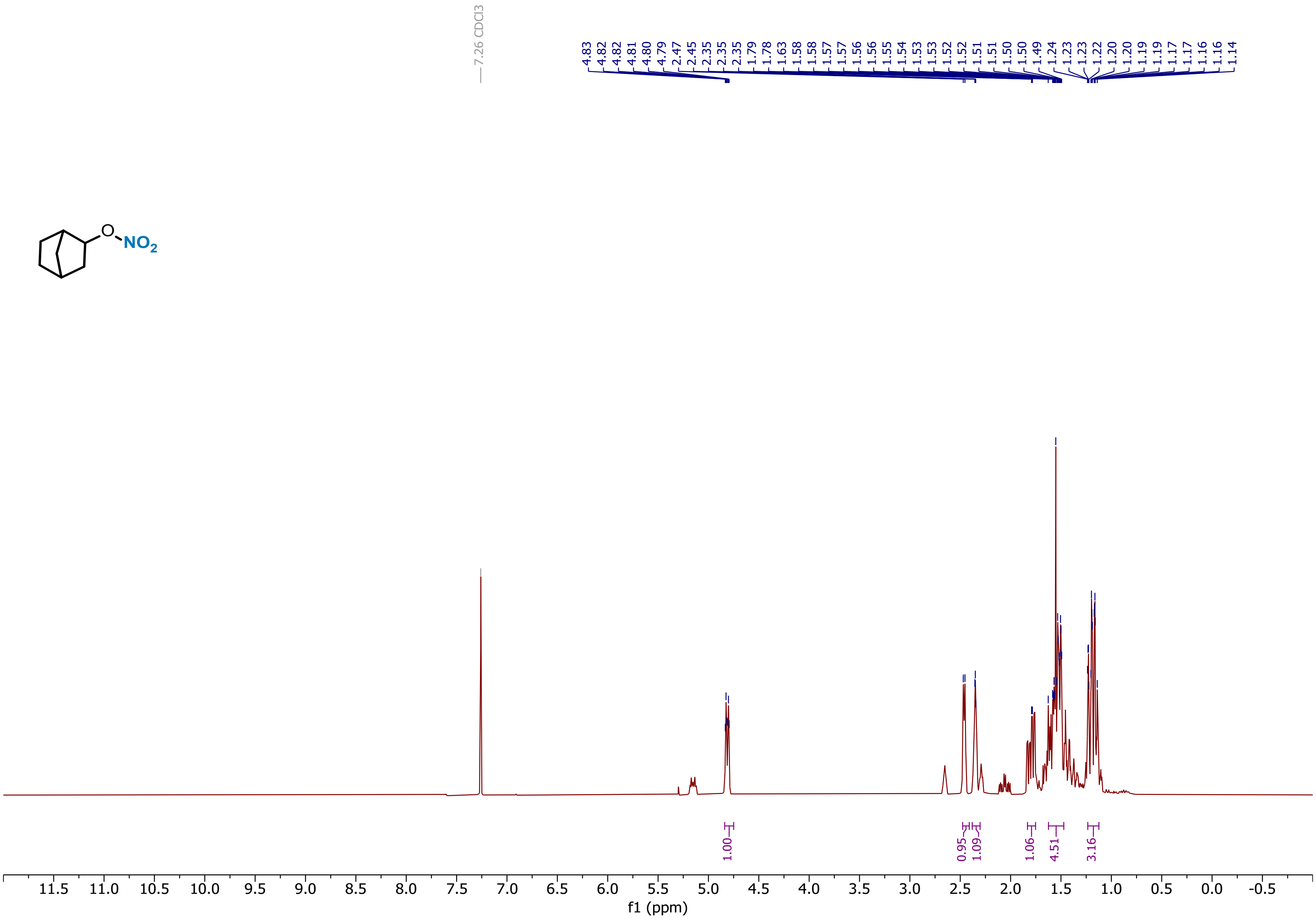

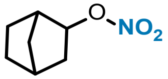

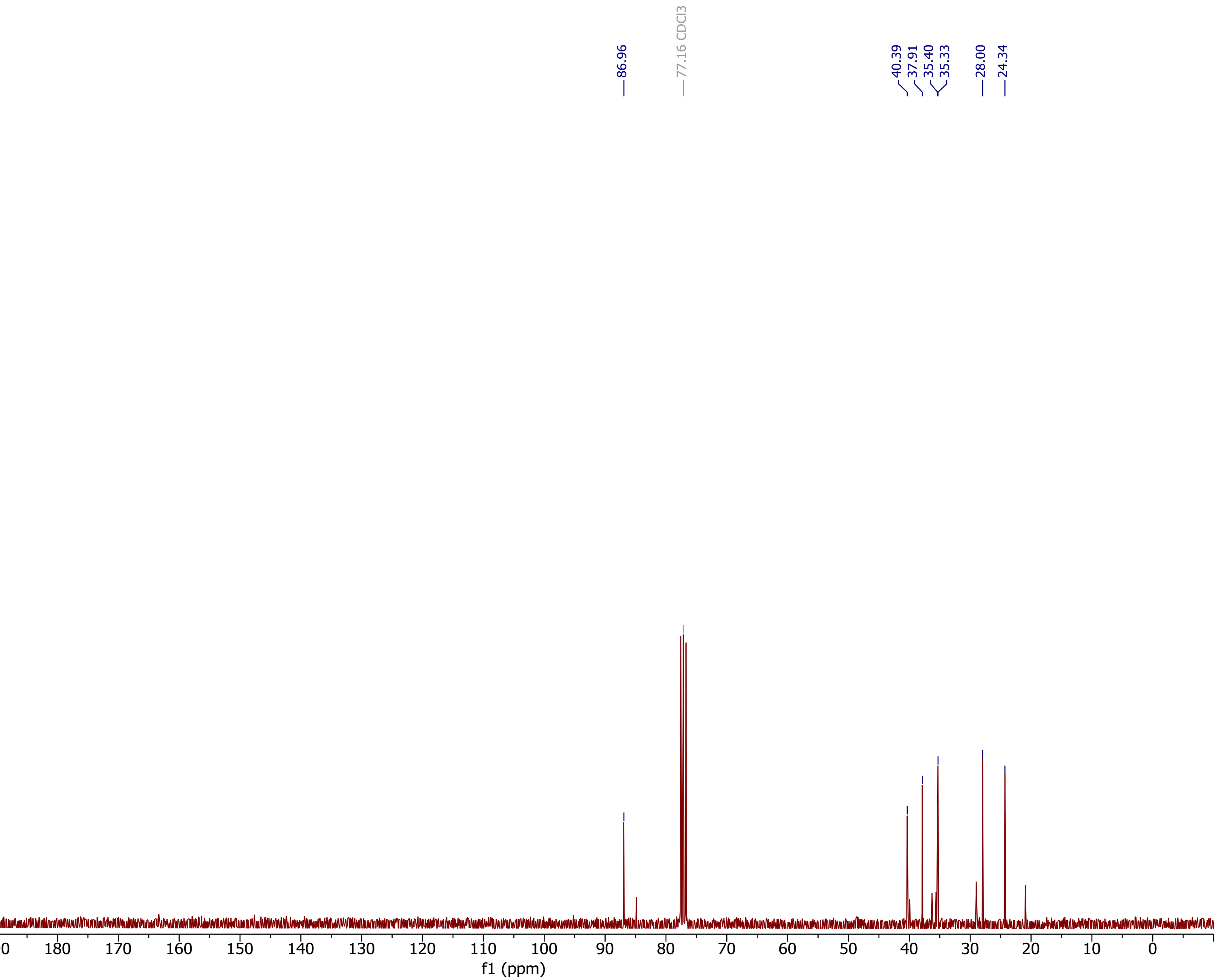

86.96
77.16 CDCl3
40.39
37.91
35.40
35.33
28.00
24.34
210
200
190
180
170
160
150
140
130
120
110
100
90
80
70
60
50
40
30
20
10
0
f1 (ppm)

1,7,7-trimethylbicyclo[2.2.1]heptan-2-yl nitrate (17)

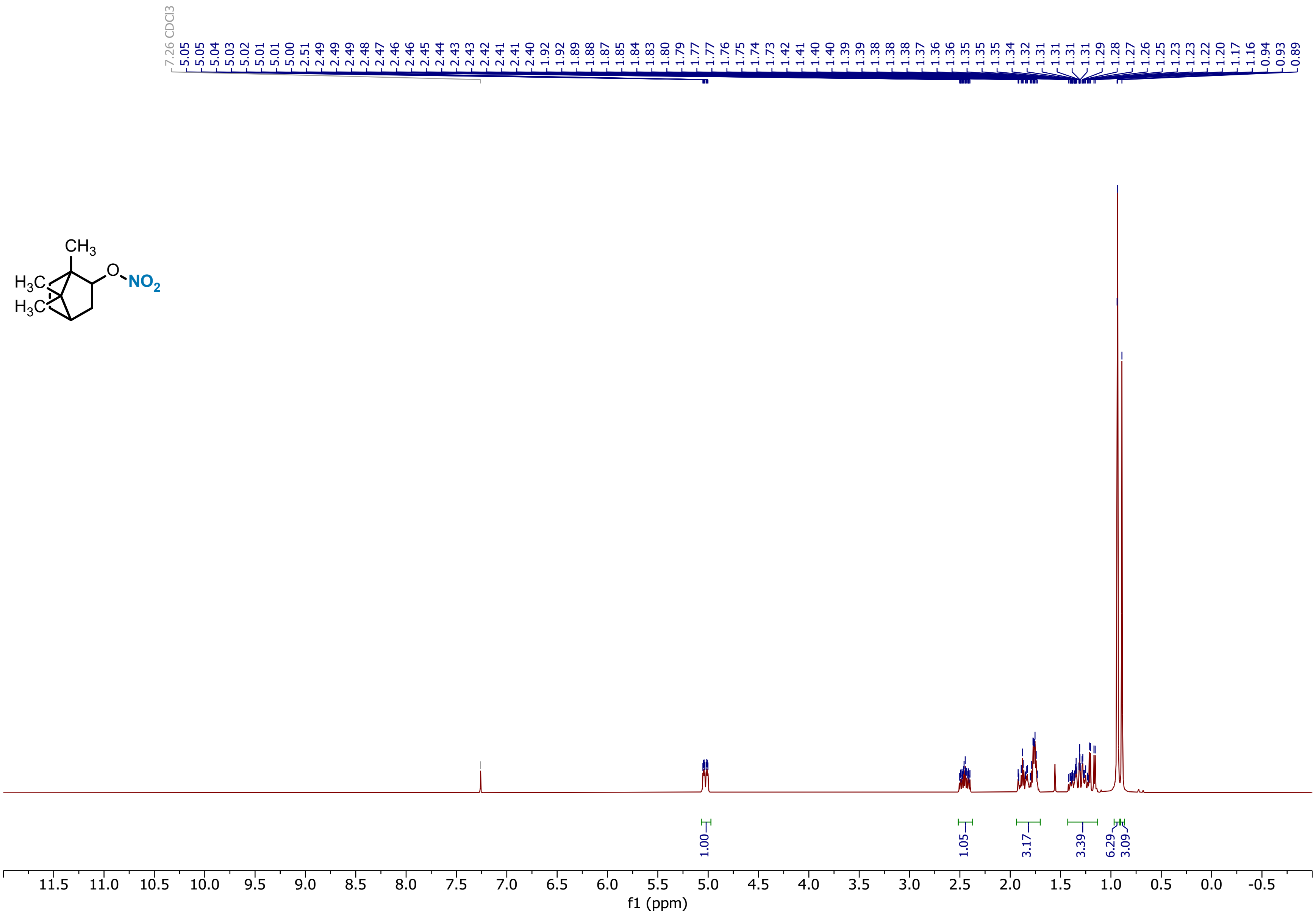

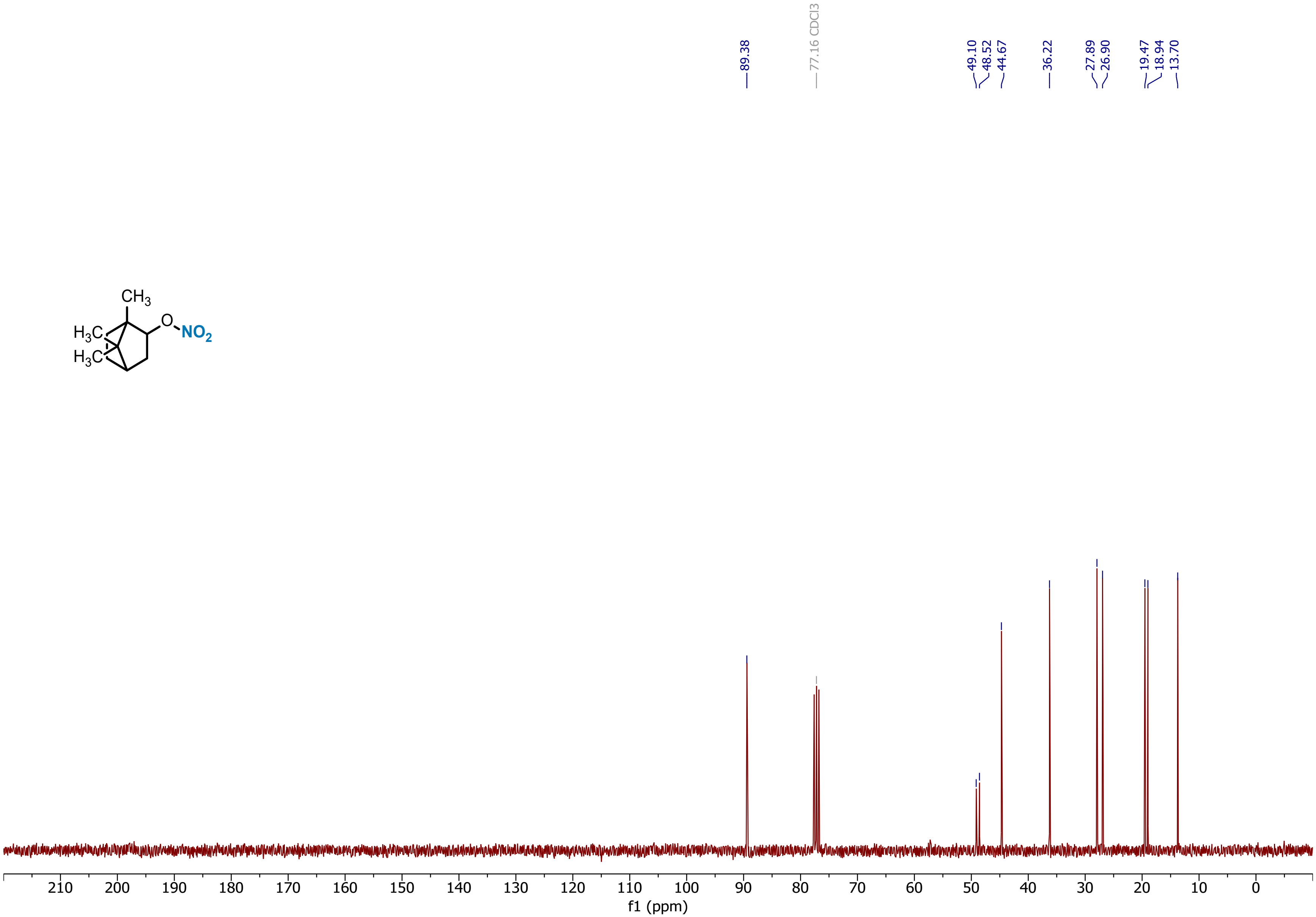
89.38
77.16 CDCl3
49.10
48.52
44.67
36.22
27.89
26.90
19.47
18.94
13.70
CH3
H3C
H3C
O
NO2
210 200 190 180 170 160 150 140 130 120 110 100 90 80 70 60 50 40 30 20 10 0
f1 (ppm)

(1,3-dioxoisoindolin-2-yl)methyl nitrate (18)

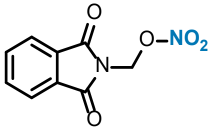

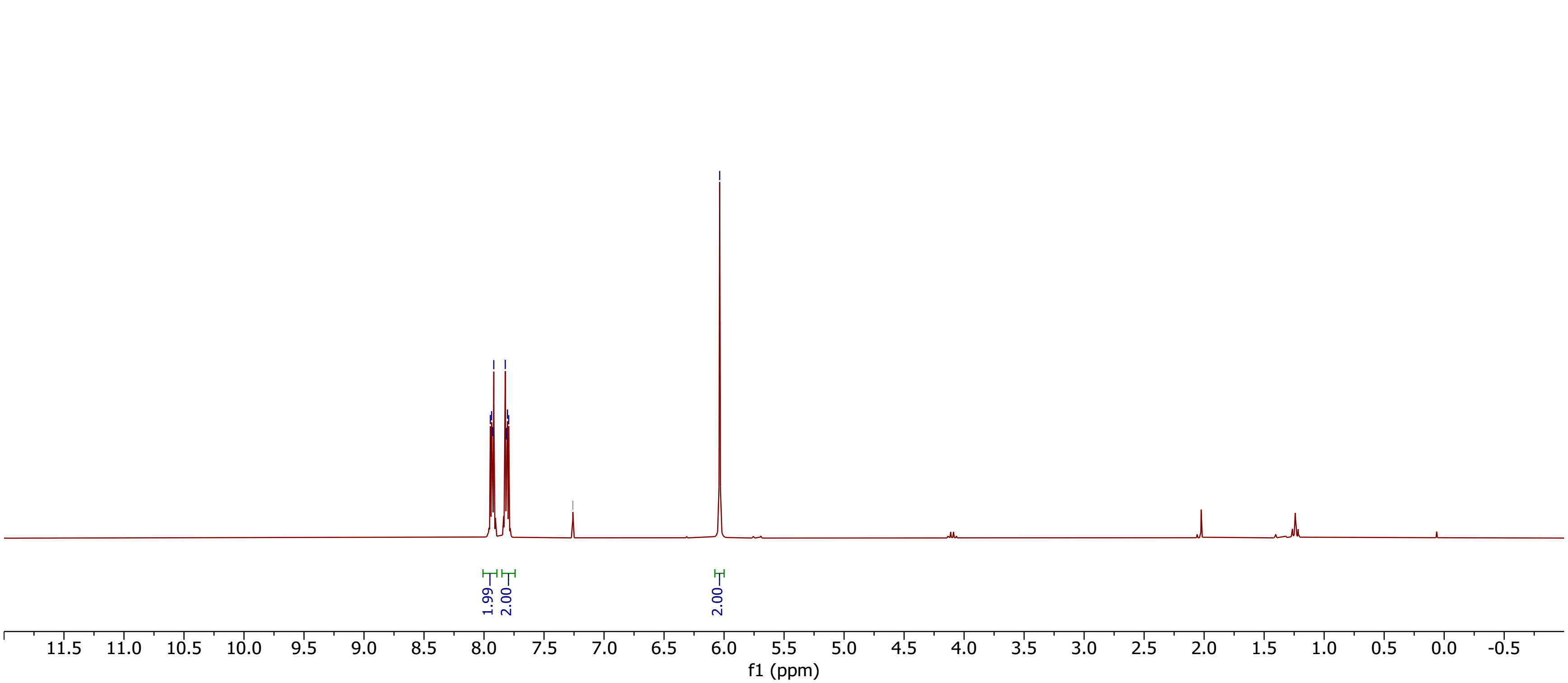

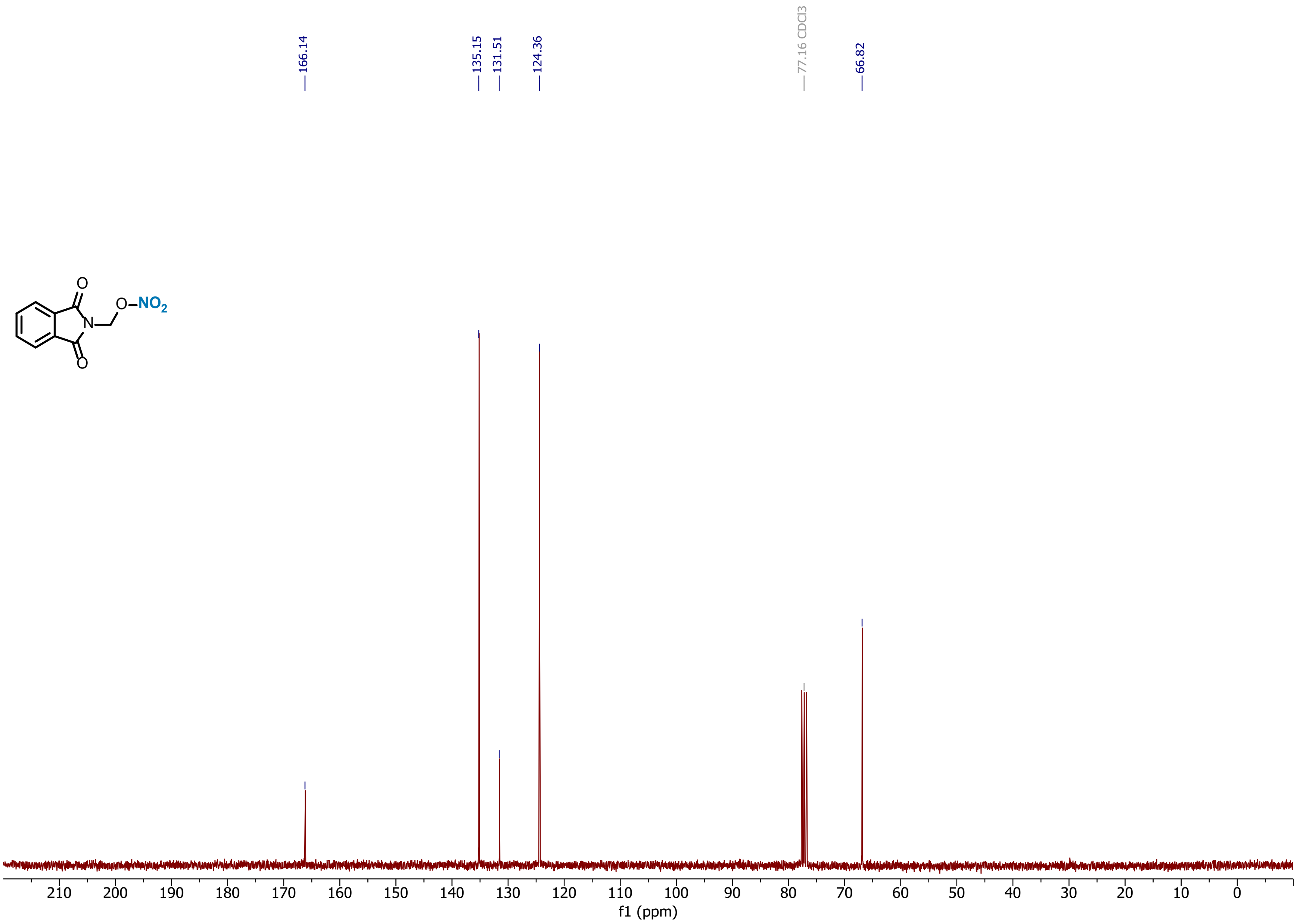

166.14
135.15
131.51
124.36
77.16 CDCl3
66.82
O
N
O
O
NO2
210
200
190
180
170
160
150
140
130
120
110
100
90
80
70
60
50
40
30
20
10
0
f1 (ppm)

2-(1H-Indol-3-yl)ethyl nitrate (19)

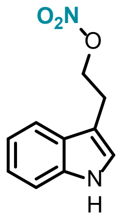

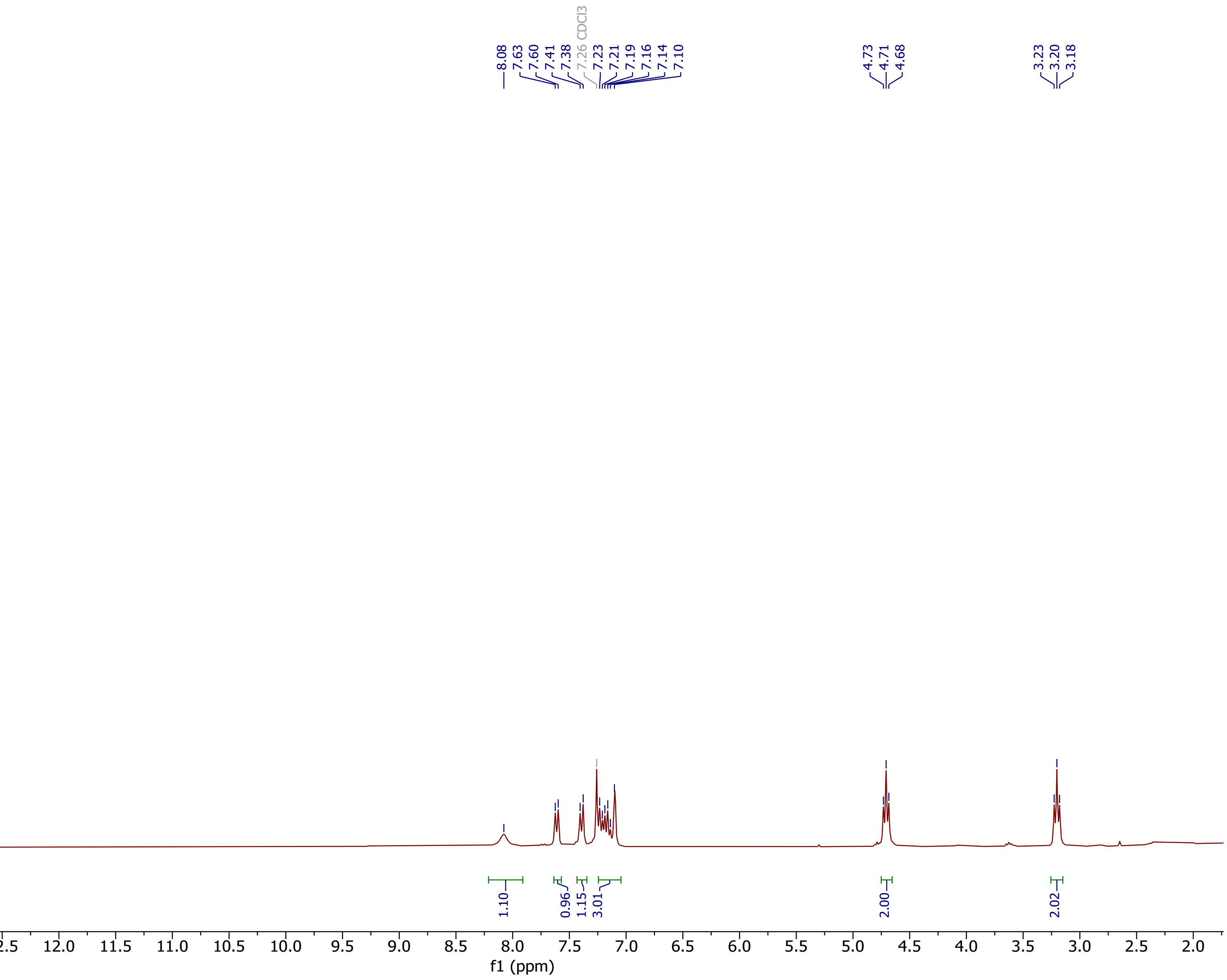

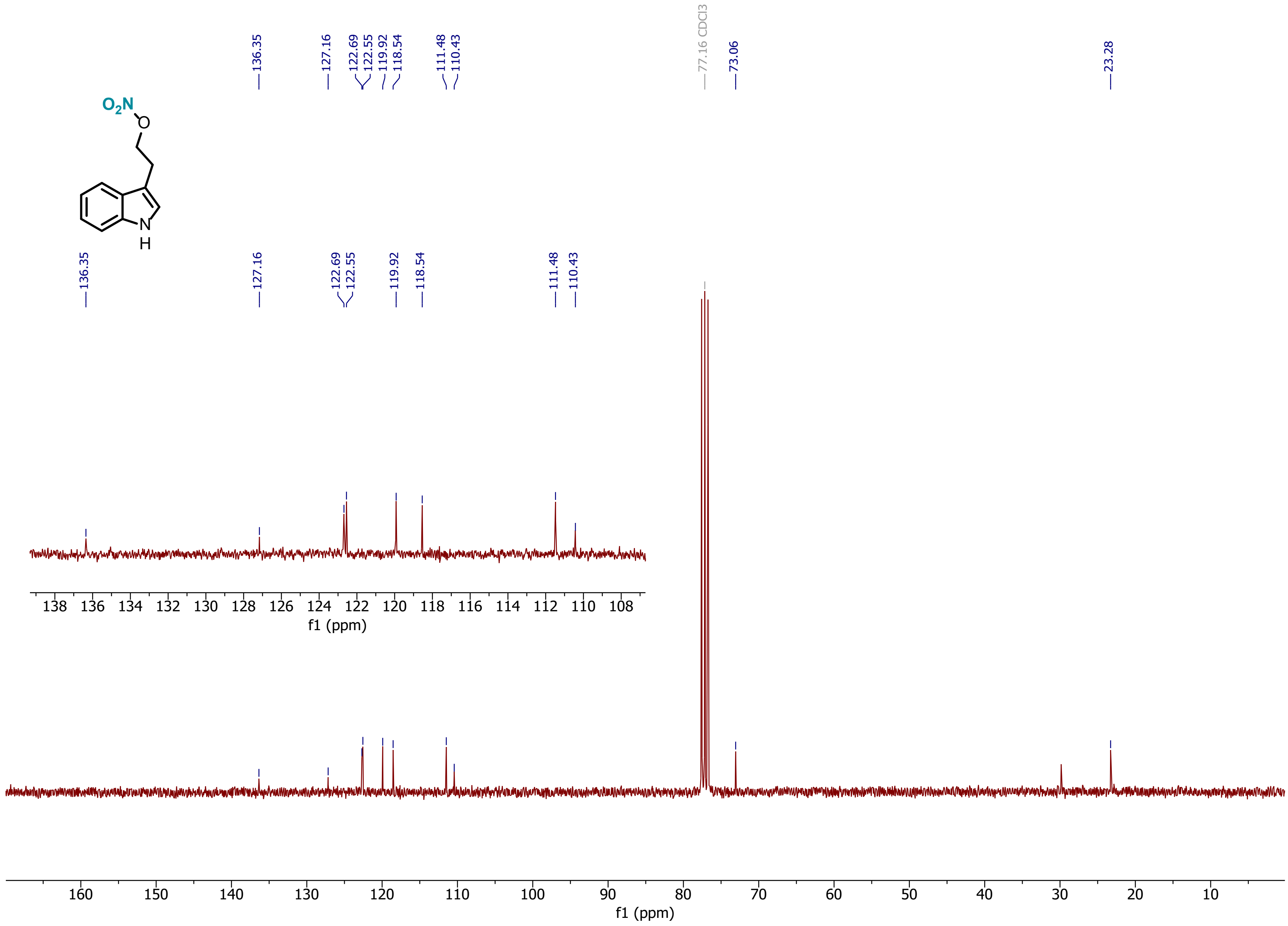
136.35
127.16
122.69
122.55
119.92
118.54
111.48
110.43
77.16 CDCl3
73.06
23.28
O2N
O
N
H
138 136 134 132 130 128 126 124 122 120 118 116 114 112 110 108
f1 (ppm)
160 150 140 130 120 110 100 90 80 70 60 50 40 30 20 10
f1 (ppm)

2-((1R,2R,8aS)-2-hydroxy-2,5,5,8a-tetramethyldecahydronaphthalen-1-yl)ethyl nitrate (20)

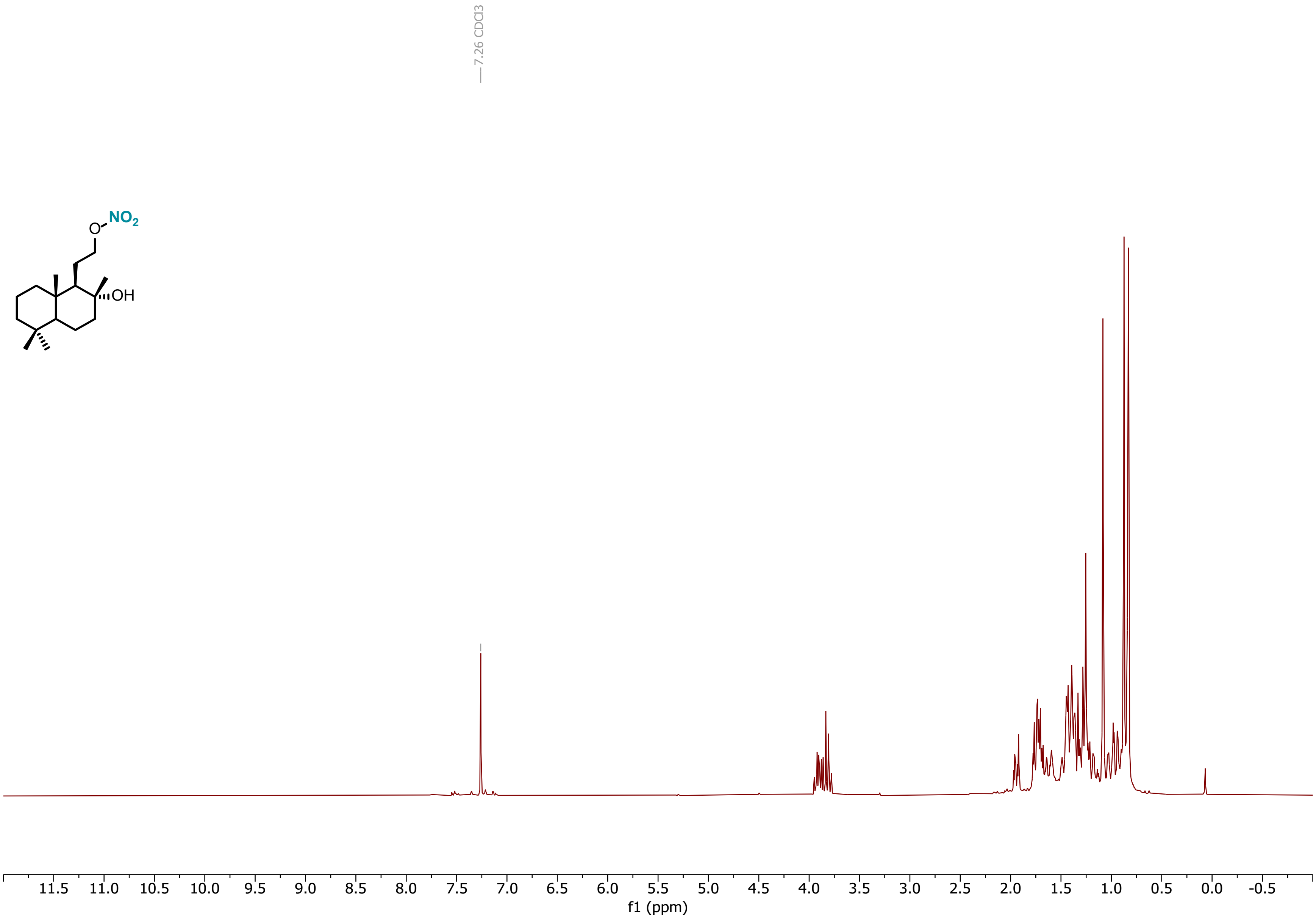

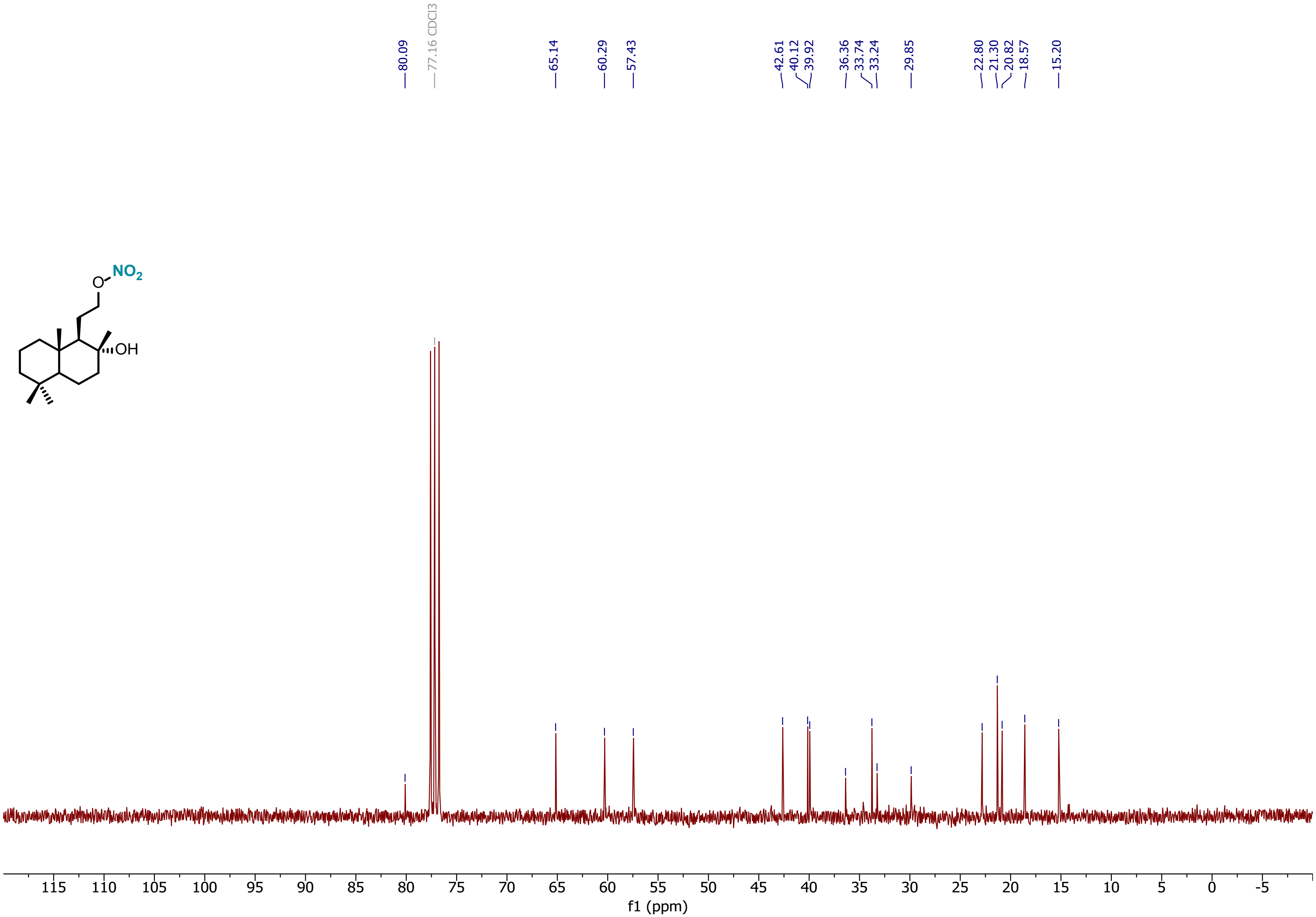

80.09
77.16 CDCl3
65.14
60.29
57.43
42.61
40.12
39.92
36.36
33.74
33.24
29.85
22.80
21.30
20.82
18.57
15.20
O
NO2
OH
115 110 105 100 95 90 85 80 75 70 65 60 55 50 45 40 35 30 25 20 15 10 5 0 -5
f1 (ppm)

(3S,8S,9S,10R,13R,14S,17R)-10,13-dimethyl-17-((R)-6-methylheptan-2-yl)-2,3,4,7,8,9,10,11,12,13,14,15,16,17-tetradecahydro-1H-cyclopenta[a]phenanthren-3-yl nitrate (21)

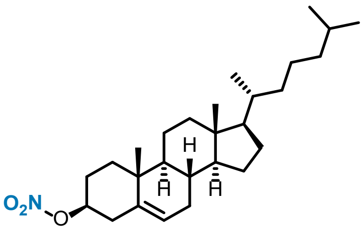

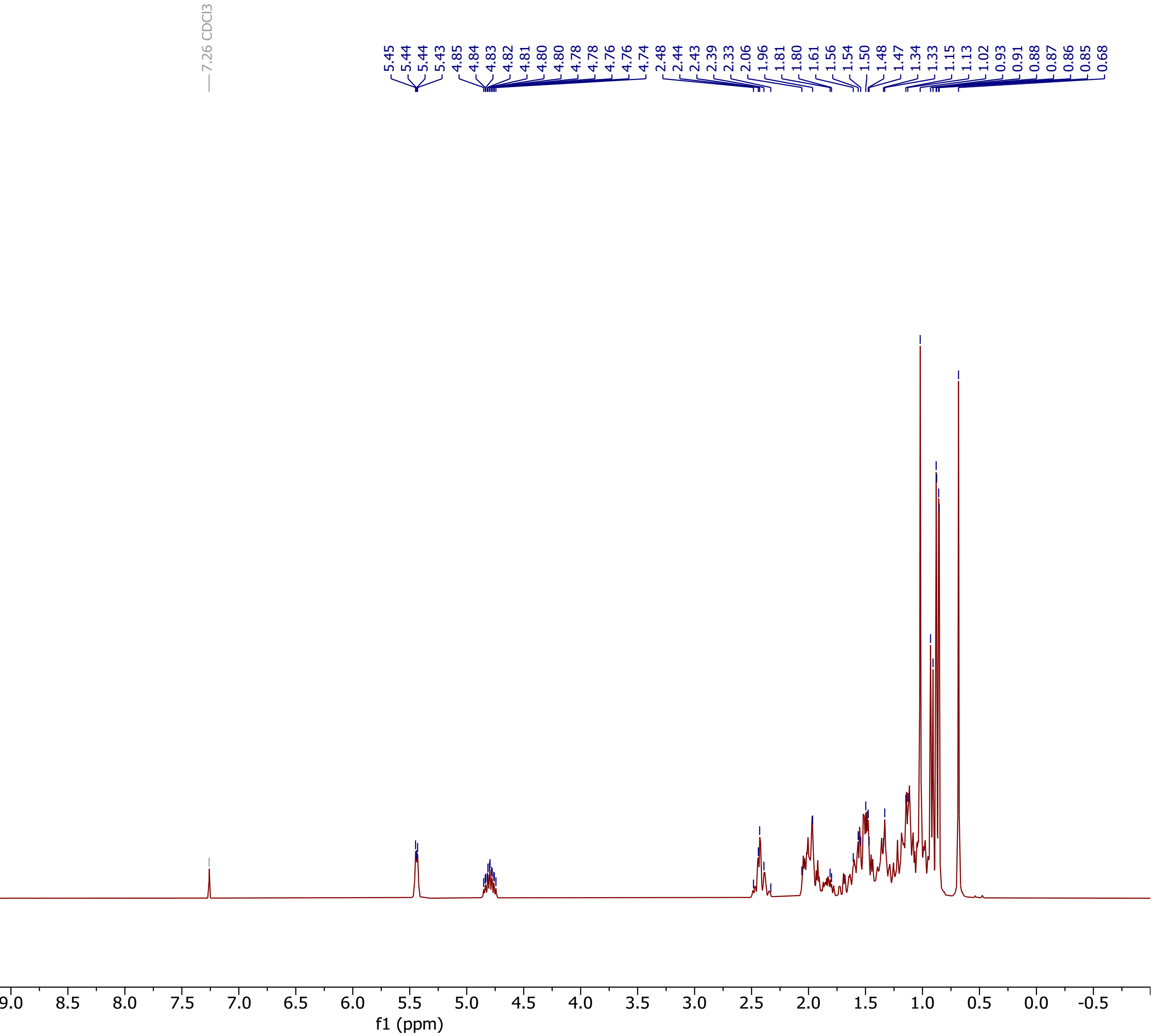

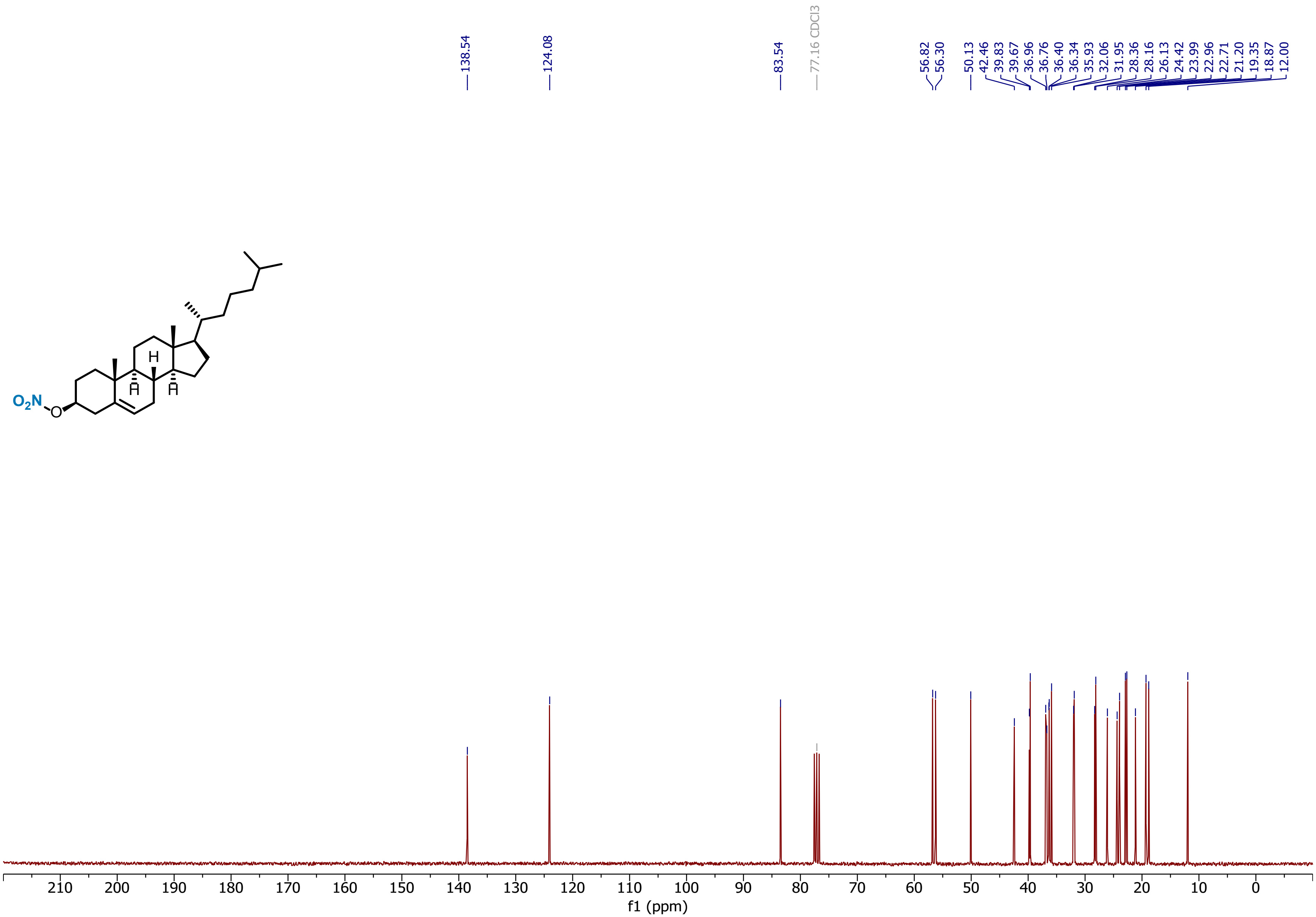
O2N
O
H
H
H
138.54
124.08
83.54
77.16 CDCl3
56.82
56.30
50.13
42.46
39.83
39.67
36.96
36.76
36.40
36.34
35.93
32.06
31.95
28.36
28.16
26.13
24.42
23.99
22.96
22.71
21.20
19.35
18.87
12.00
210
200
190
180
170
160
150
140
130
120
110
100
90
80
70
60
50
40
30
20
10
0
f1 (ppm)

1-phenylethane-1,2-diyl dinitrate (22b)

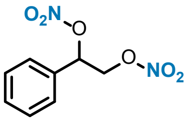

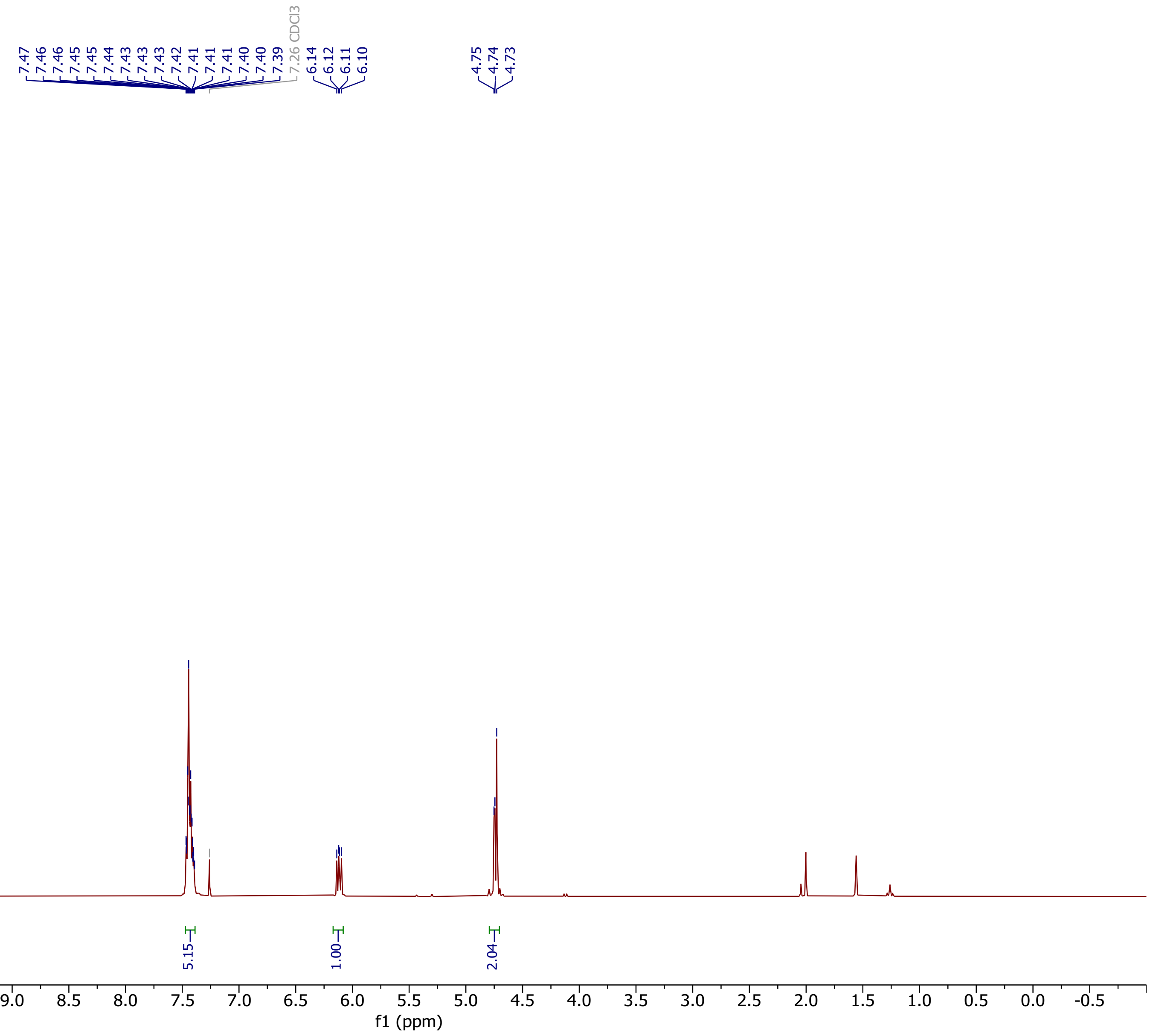

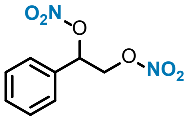
O2N
O
O
NO2

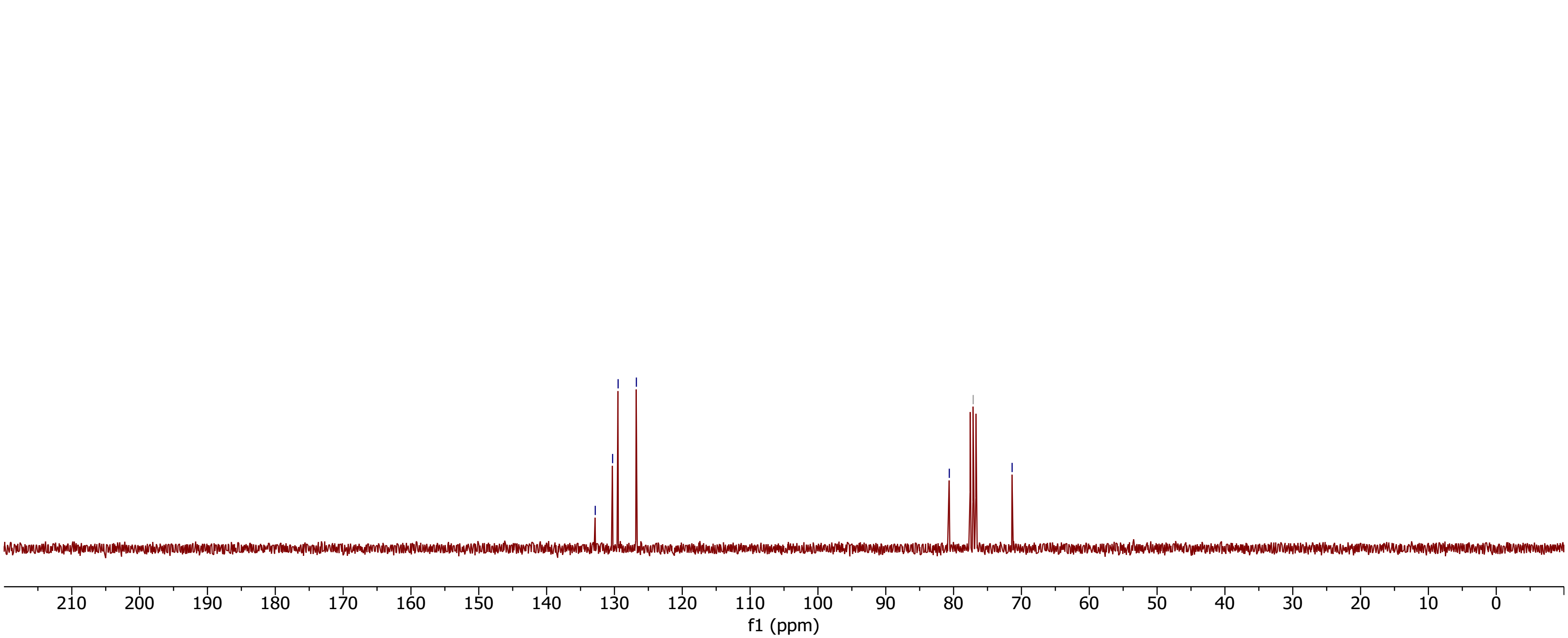
132.88
130.32
129.51
126.81
80.68
77.16 CDCl3
71.42
210
200
190
180
170
160
150
140
130
120
110
100
90
80
70
60
50
40
30
20
10
0
f1 (ppm)

1-methoxy-4-nitrobenzene (23a)

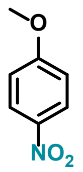

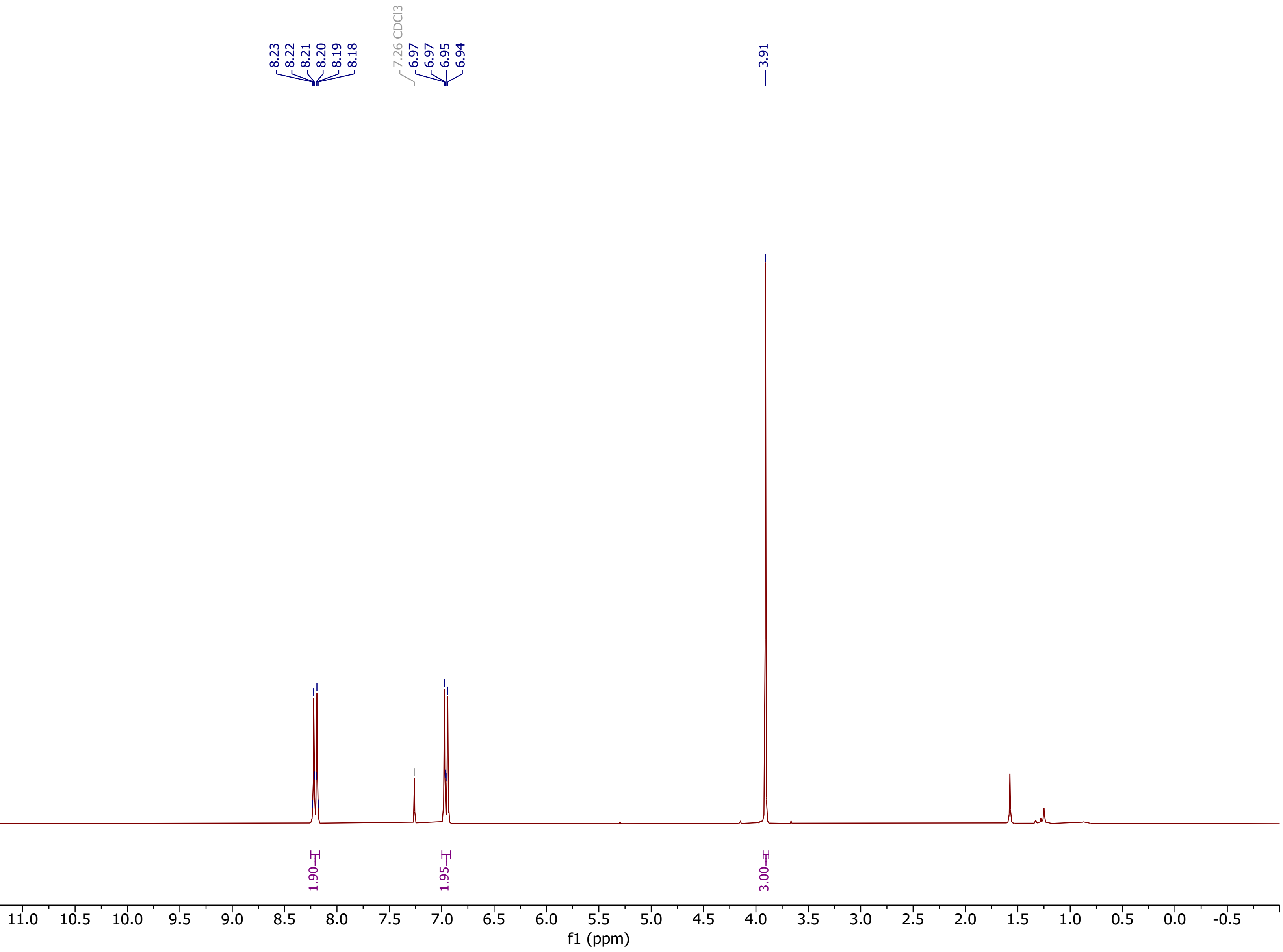

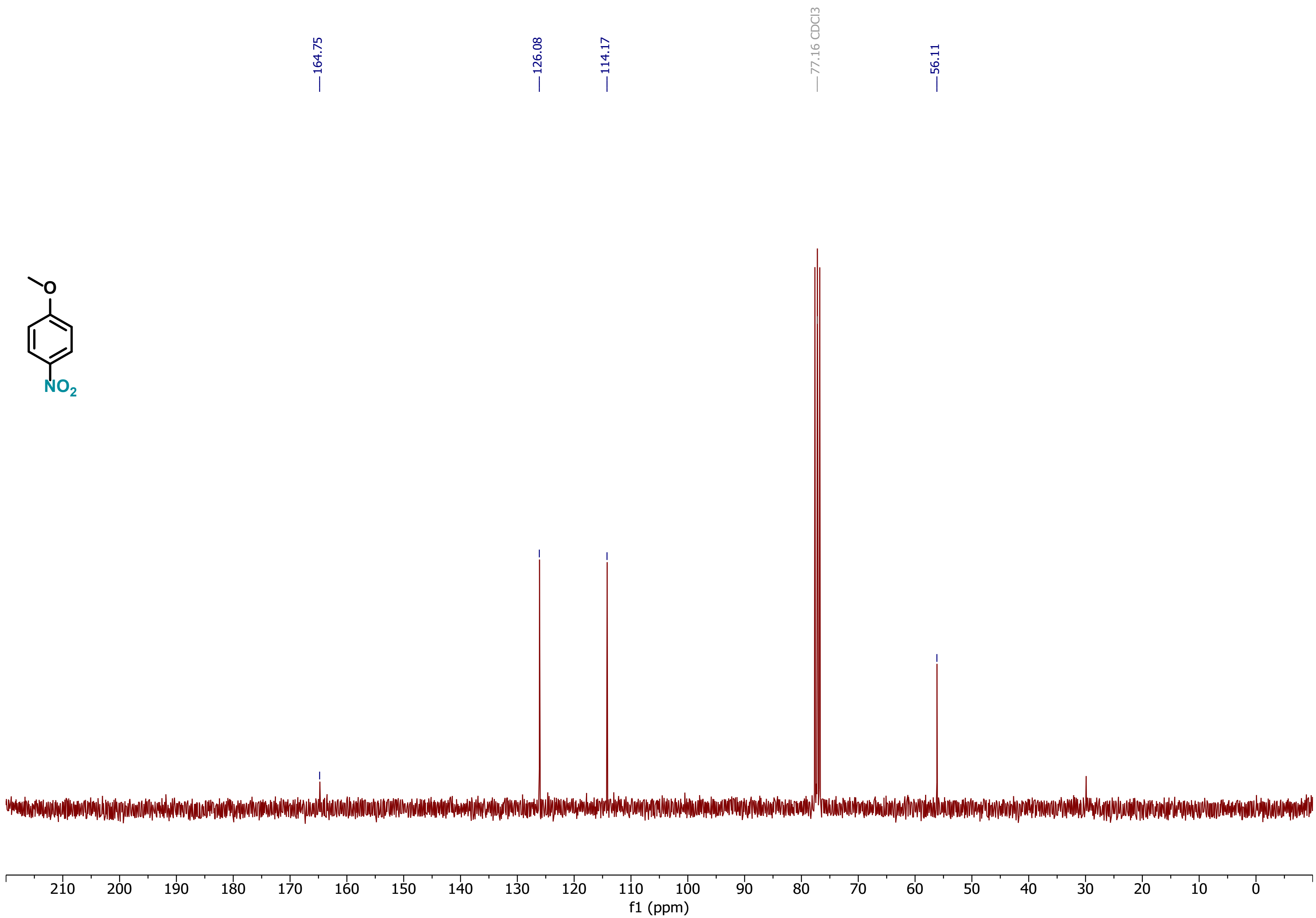

164.75
126.08
114.17
77.16 CDCl3
56.11
O
NO_2
210 200 190 180 170 160 150 140 130 120 110 100 90 80 70 60 50 40 30 20 10 0
f1 (ppm)

1-methoxy-2-nitrobenzene (23b)

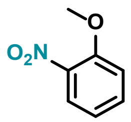

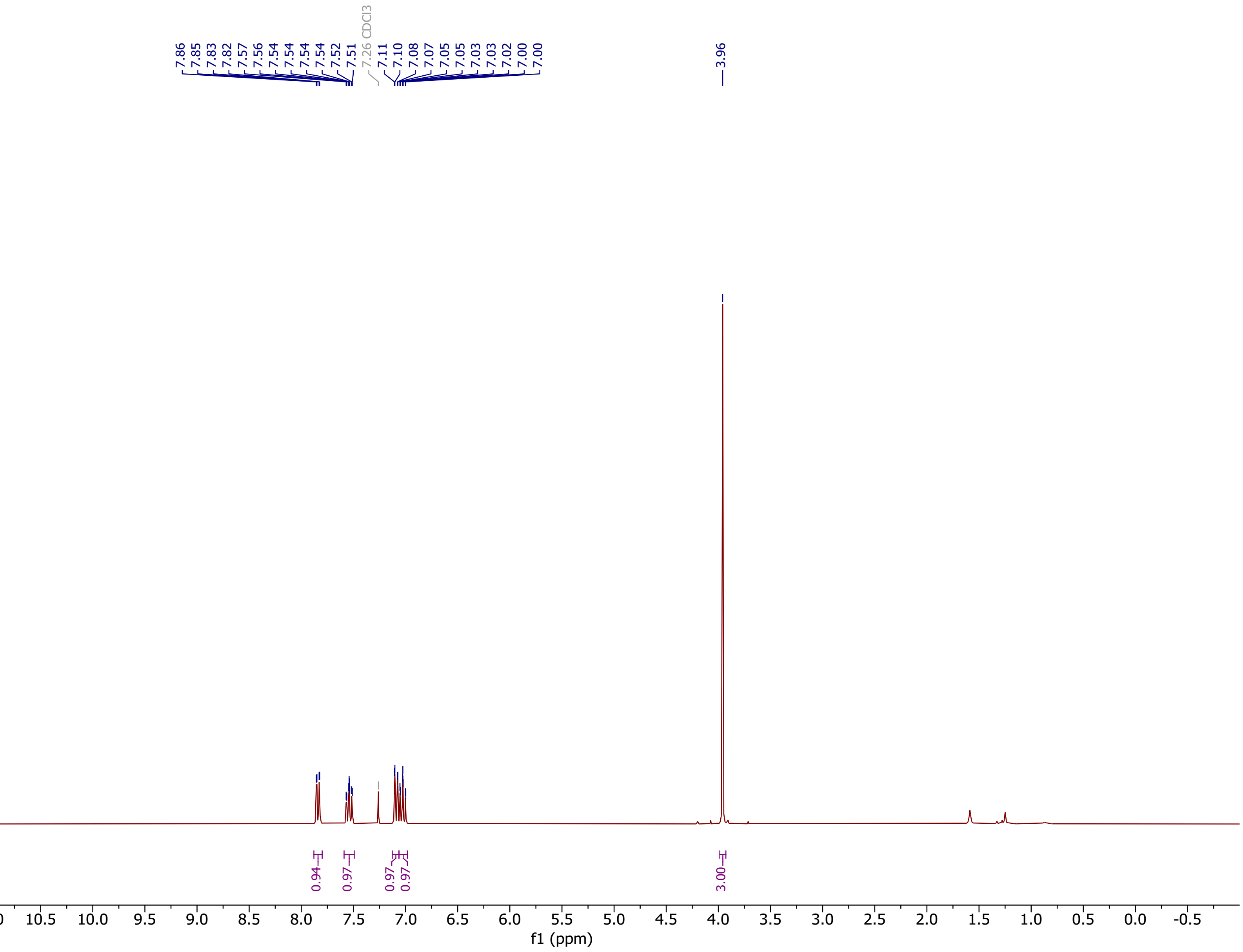

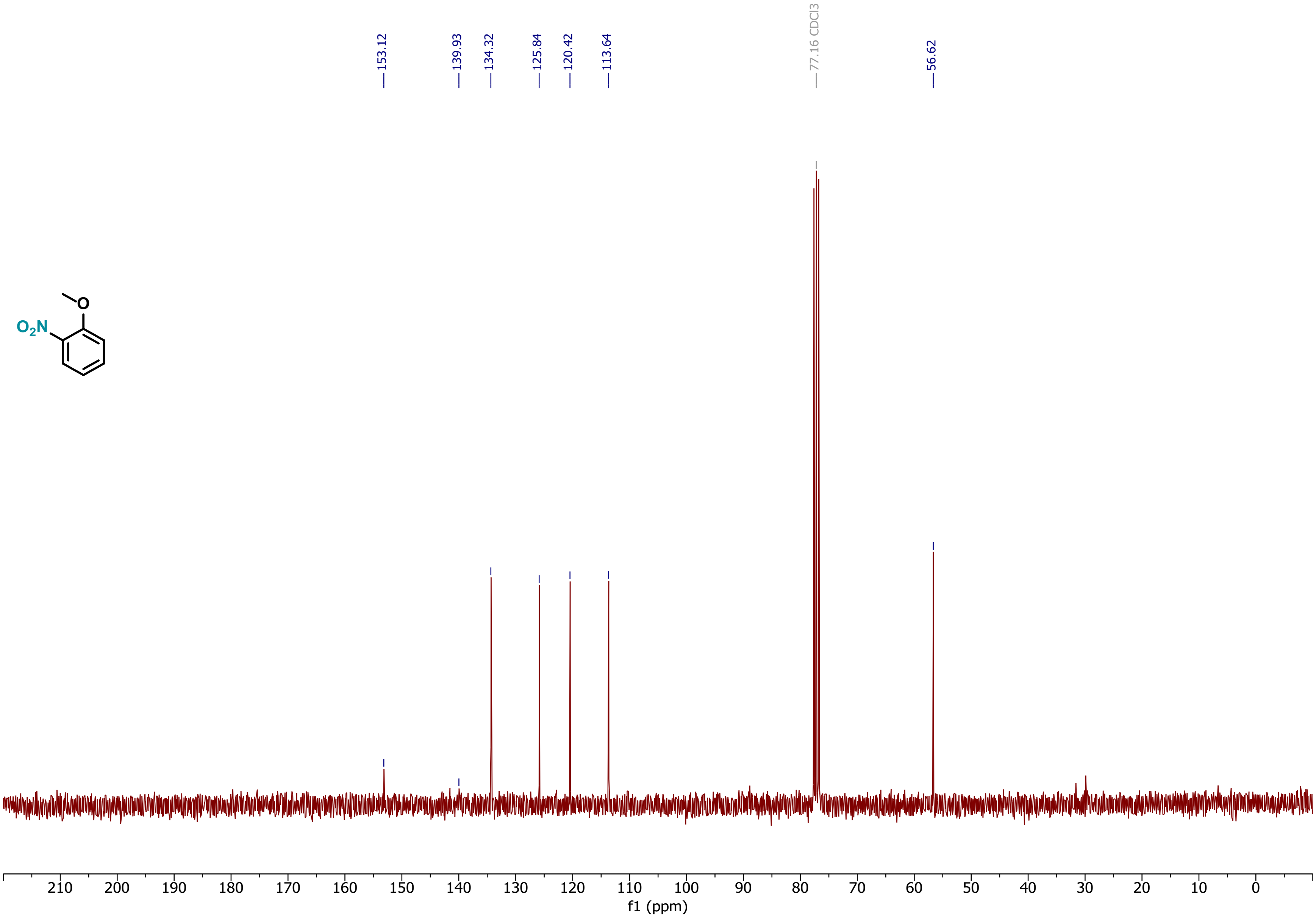
153.12
139.93
134.32
125.84
120.42
113.64
77.16 CDCl3
56.62
O_2N
O
210 200 190 180 170 160 150 140 130 120 110 100 90 80 70 60 50 40 30 20 10 0
f1 (ppm)

1,4-dimethoxy-2-nitrobenzene (24)

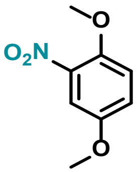

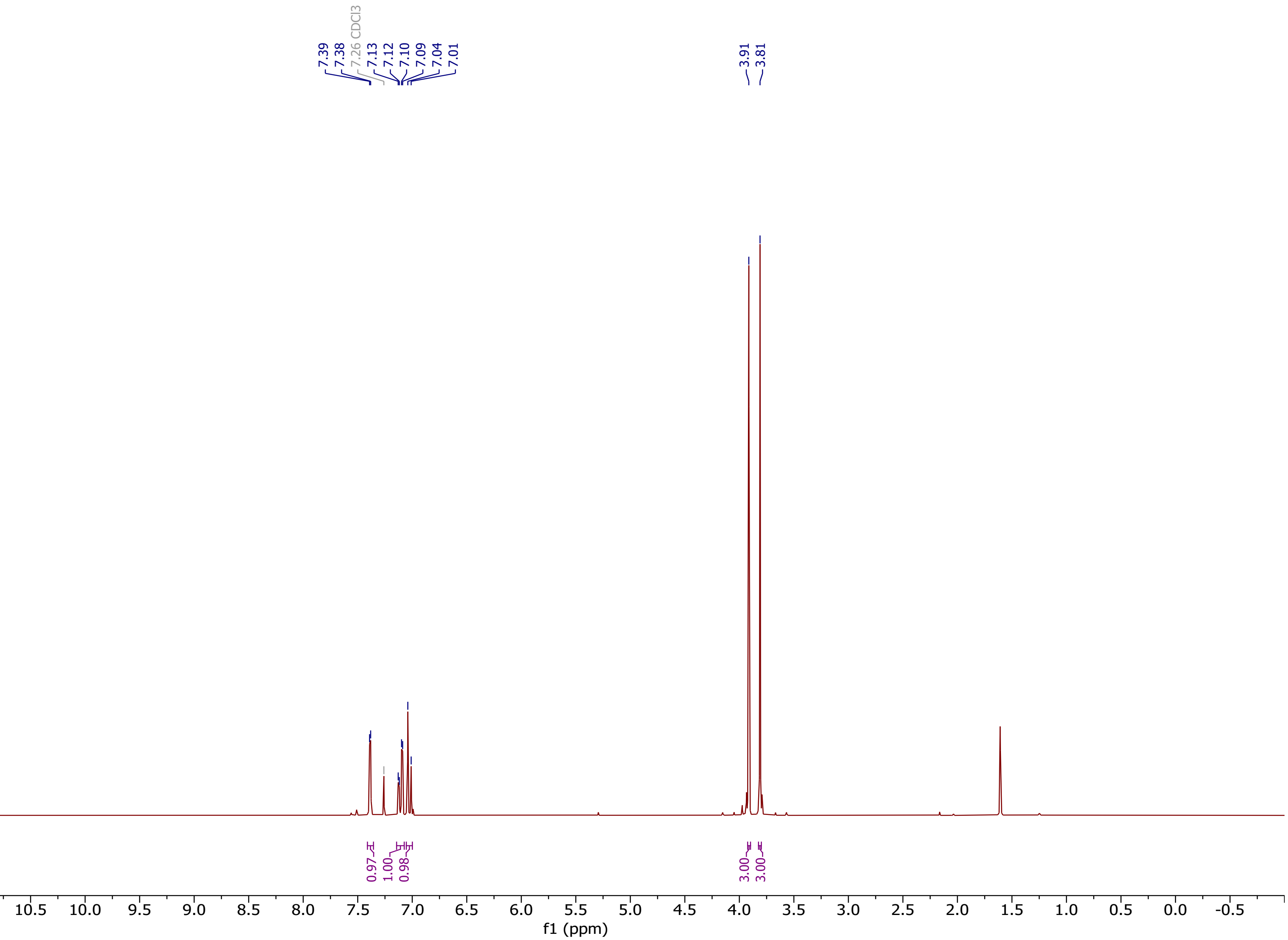

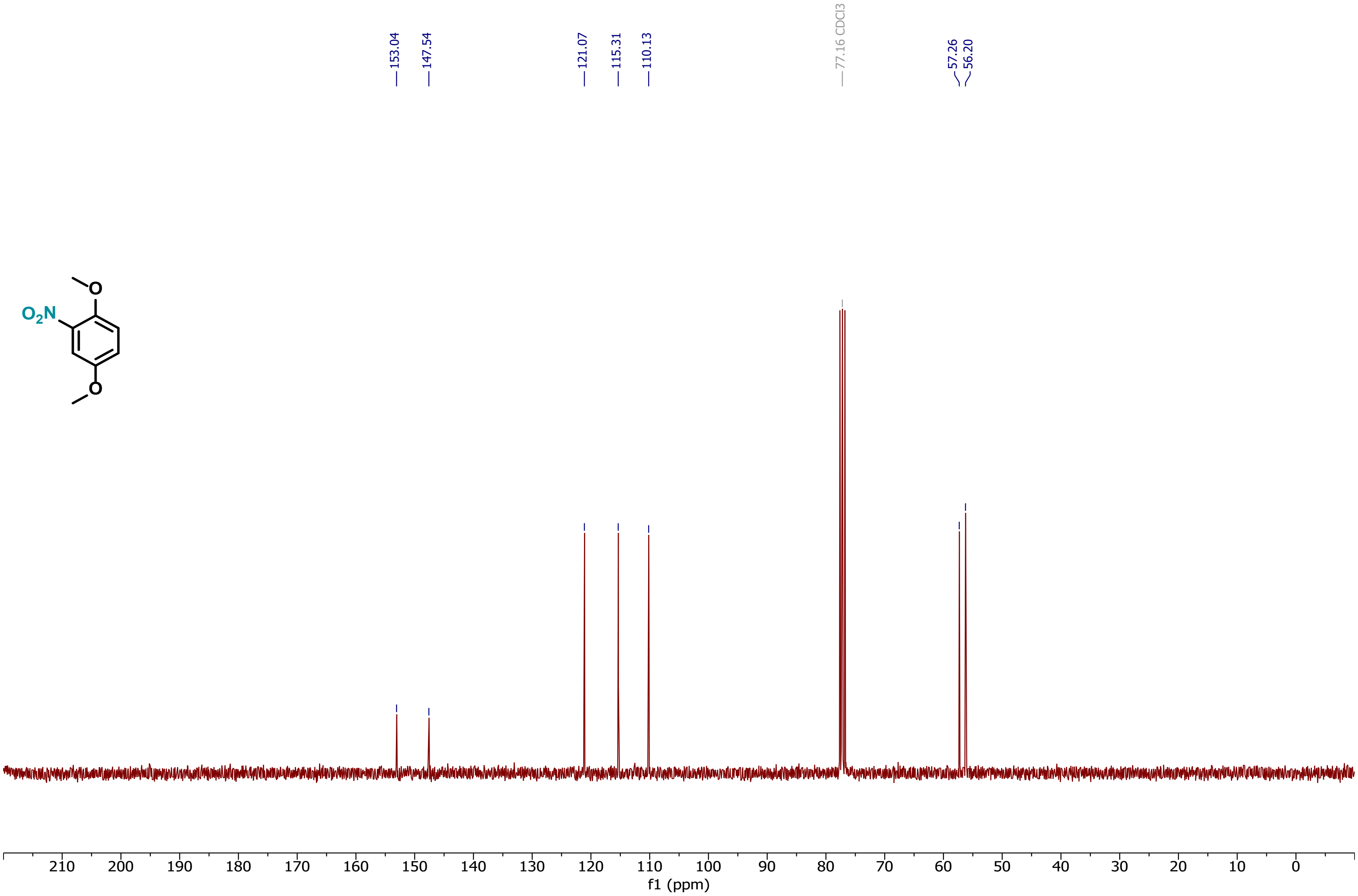

O2N
O
O
153.04
147.54
121.07
115.31
110.13
77.16 CDCl3
57.26
56.20
210
200
190
180
170
160
150
140
130
120
110
100
90
80
70
60
50
40
30
20
10
0
f1 (ppm)

1,3,5-trimethoxy-2-nitrobenzene (25)

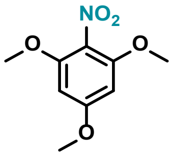

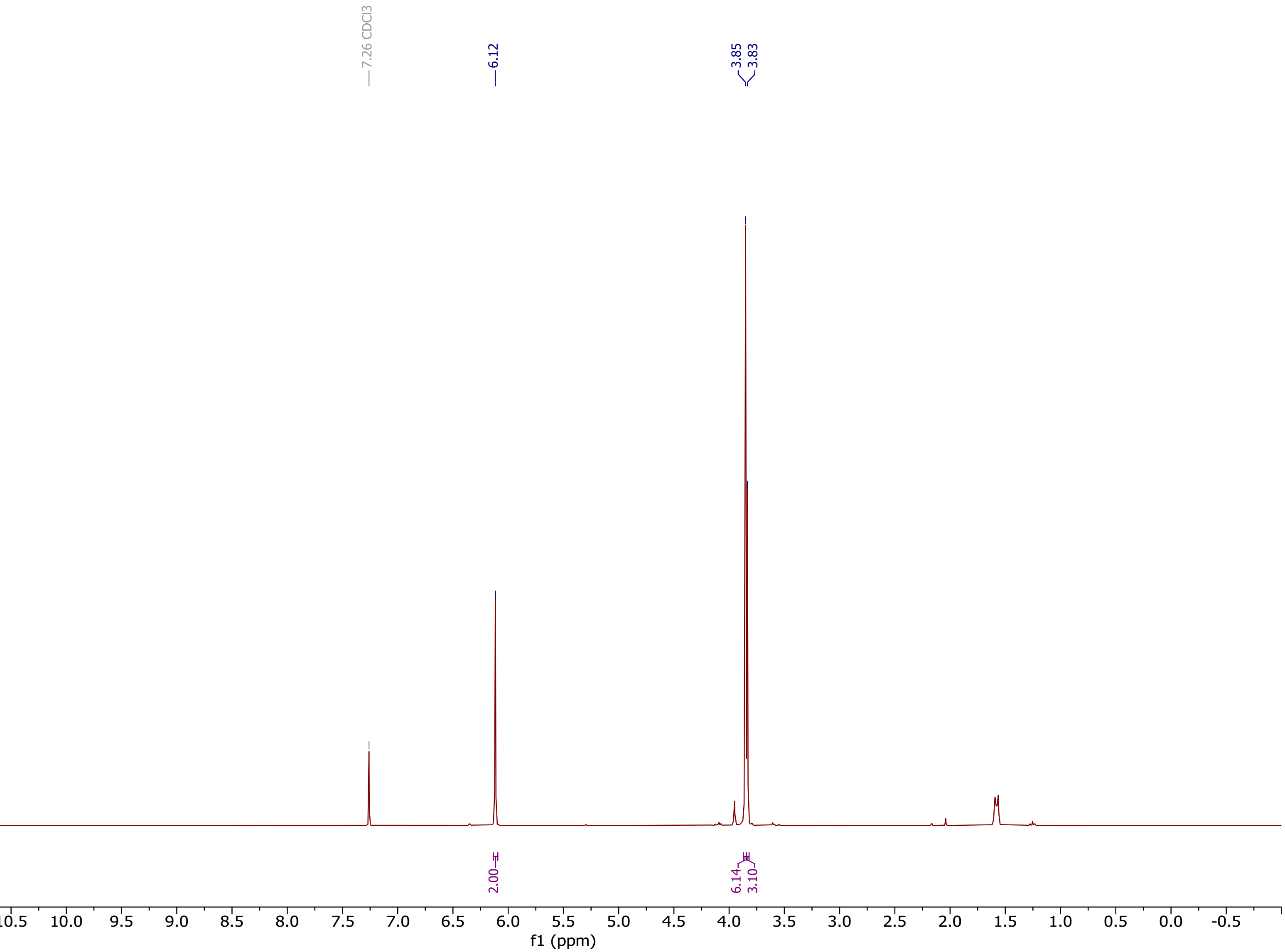

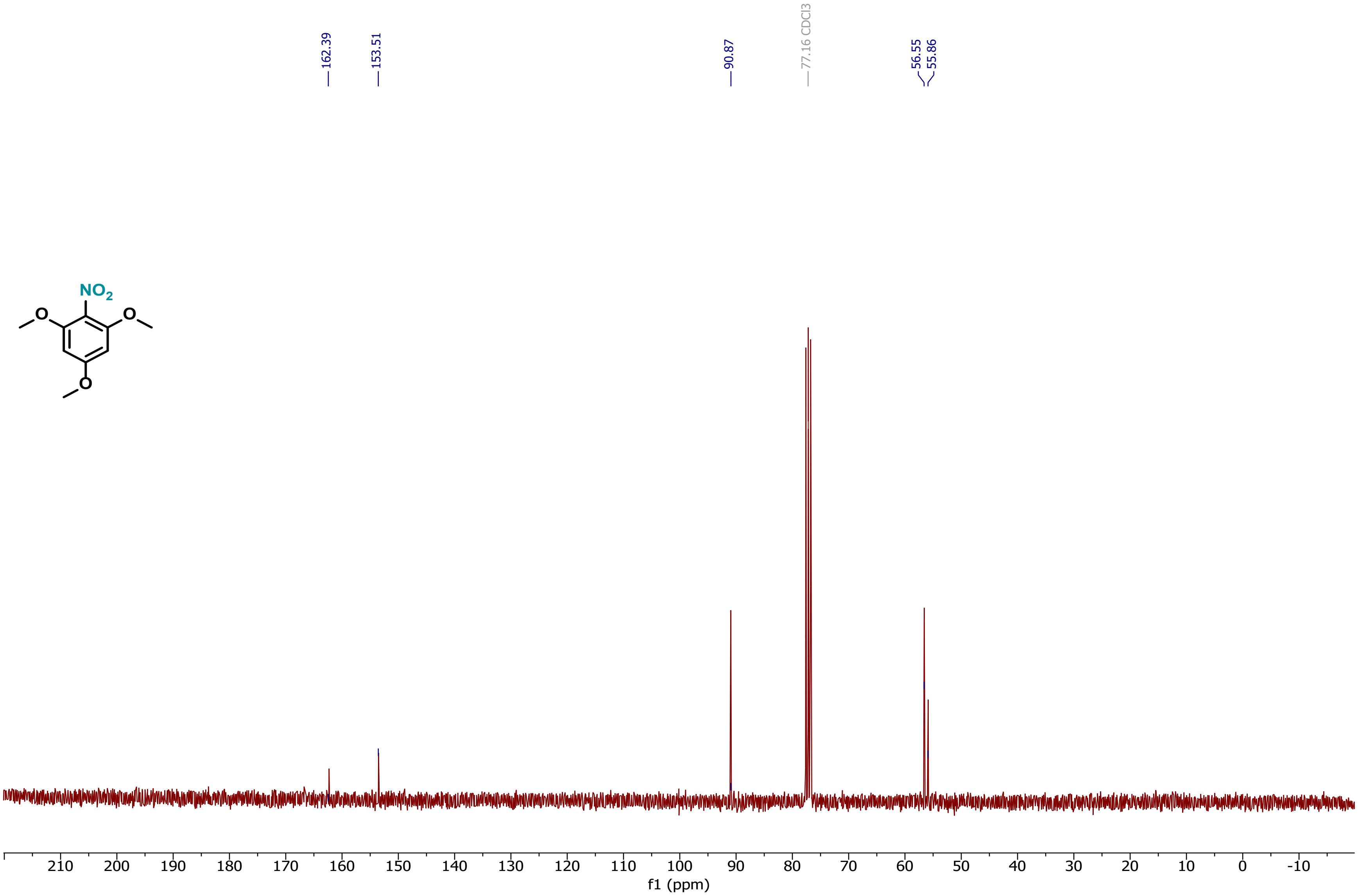
NO2
O
O
O
162.39
153.51
90.87
77.16 CDCl3
56.55
55.86
210
200
190
180
170
160
150
140
130
120
110
100
90
80
70
60
50
40
30
20
10
0
-10
f1 (ppm)

1-methoxy-4-methyl-2-nitrobenzene (26)

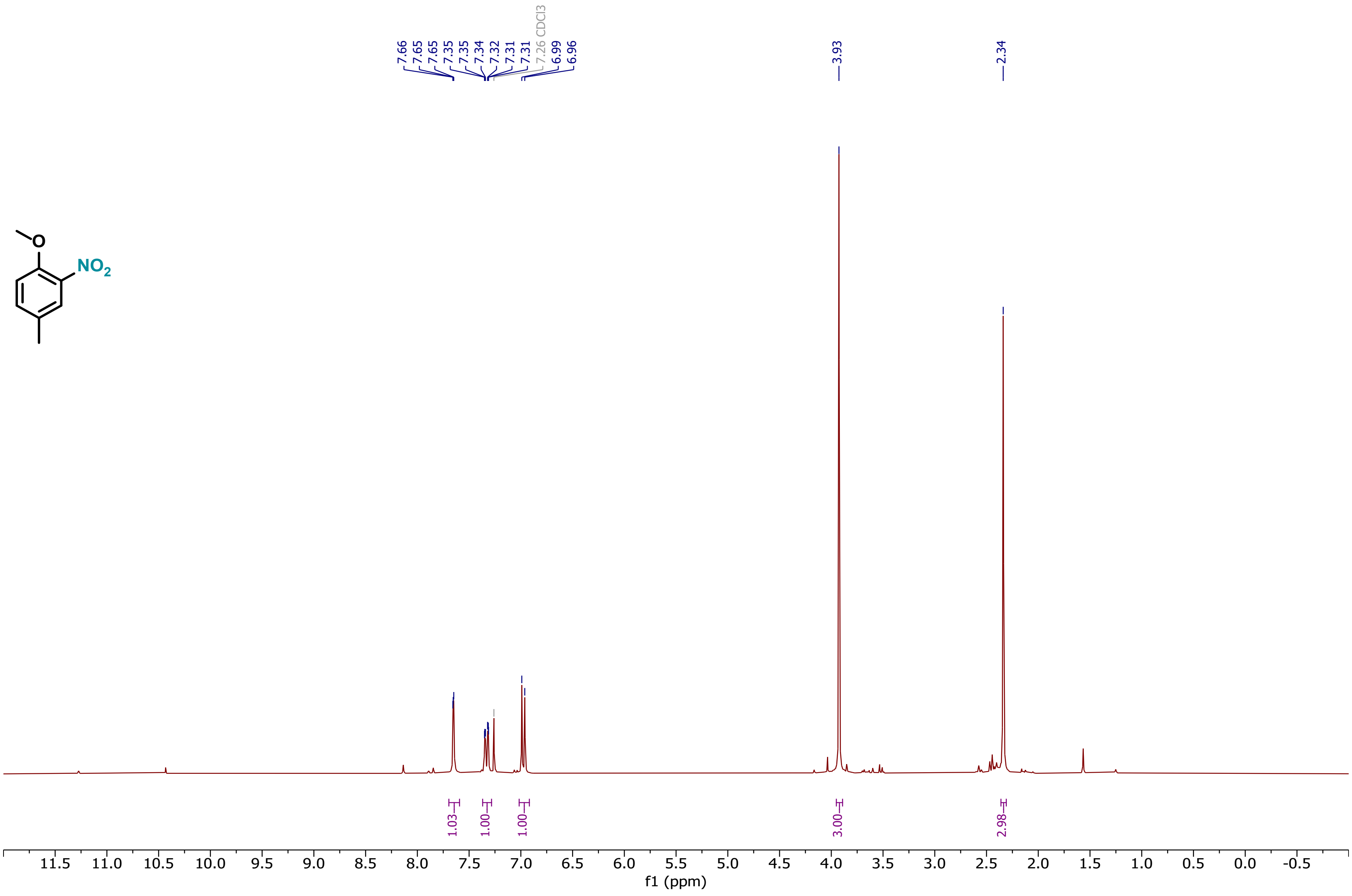

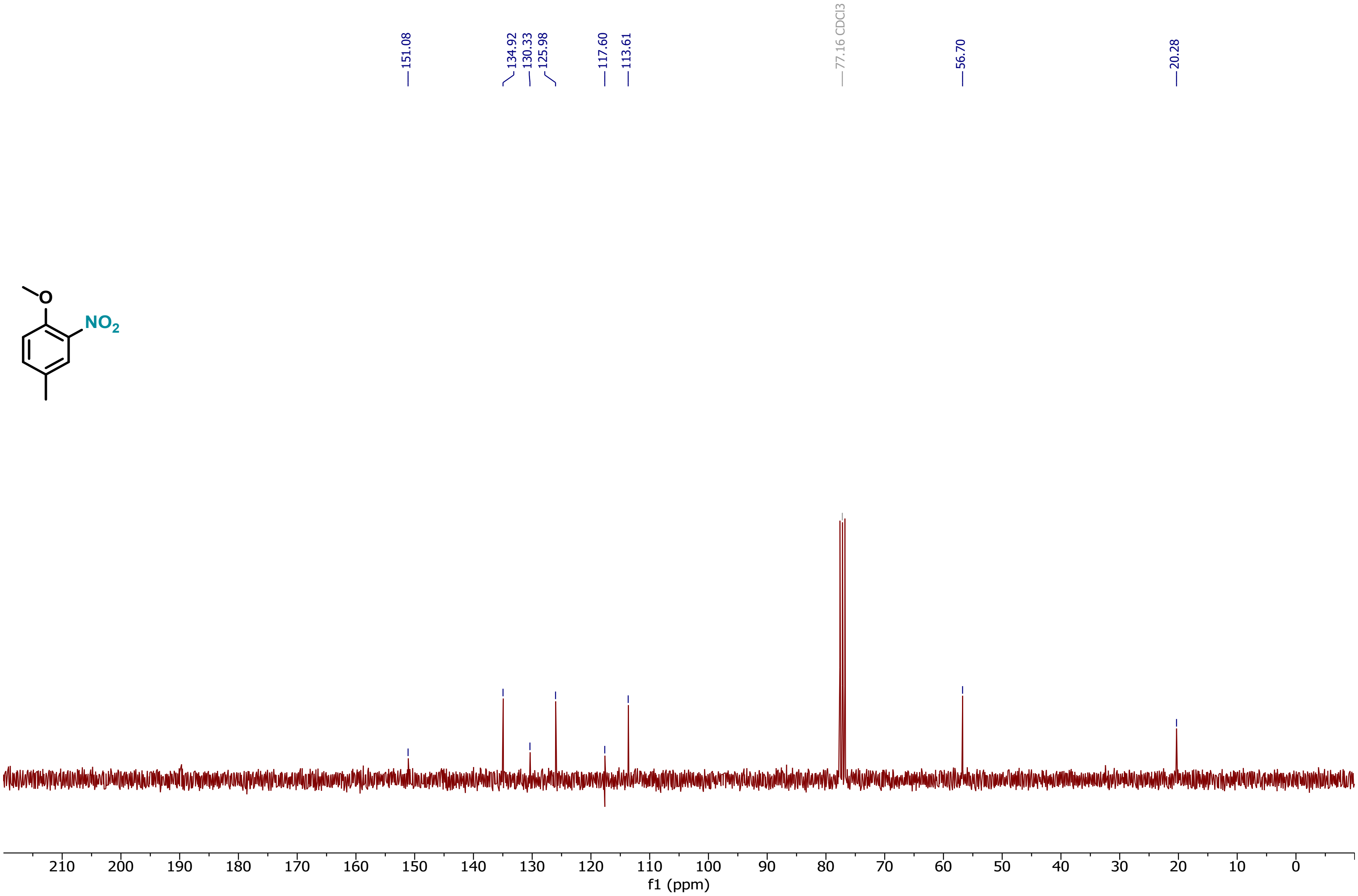

NO2
151.08
134.92
130.33
125.98
117.60
113.61
77.16 CDCl3
56.70
20.28
210
200
190
180
170
160
150
140
130
120
110
100
90
80
70
60
50
40
30
20
10
0
f1 (ppm)

1,3-dimethoxy-2-methyl-4-nitrobenzene (27)

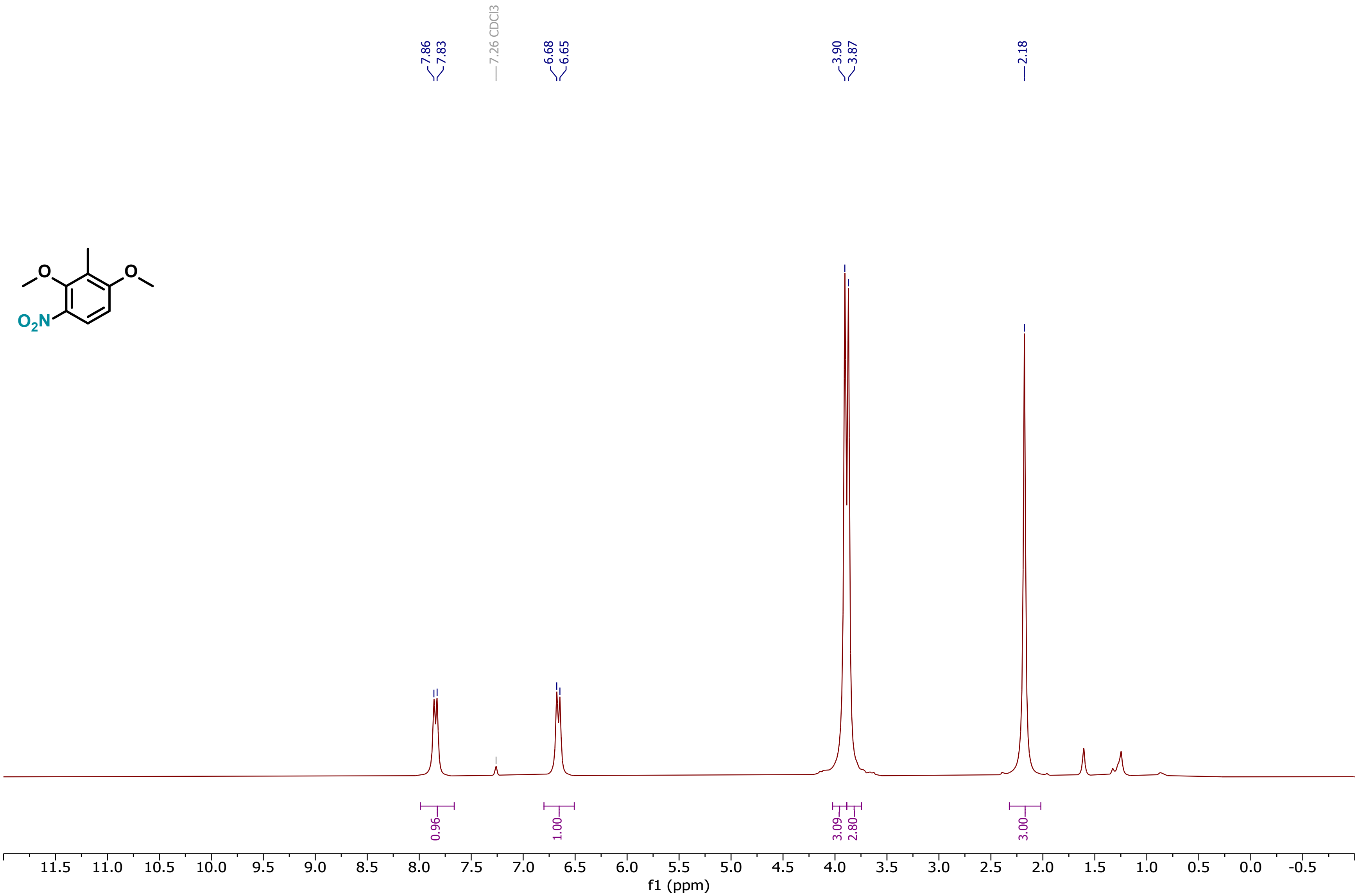

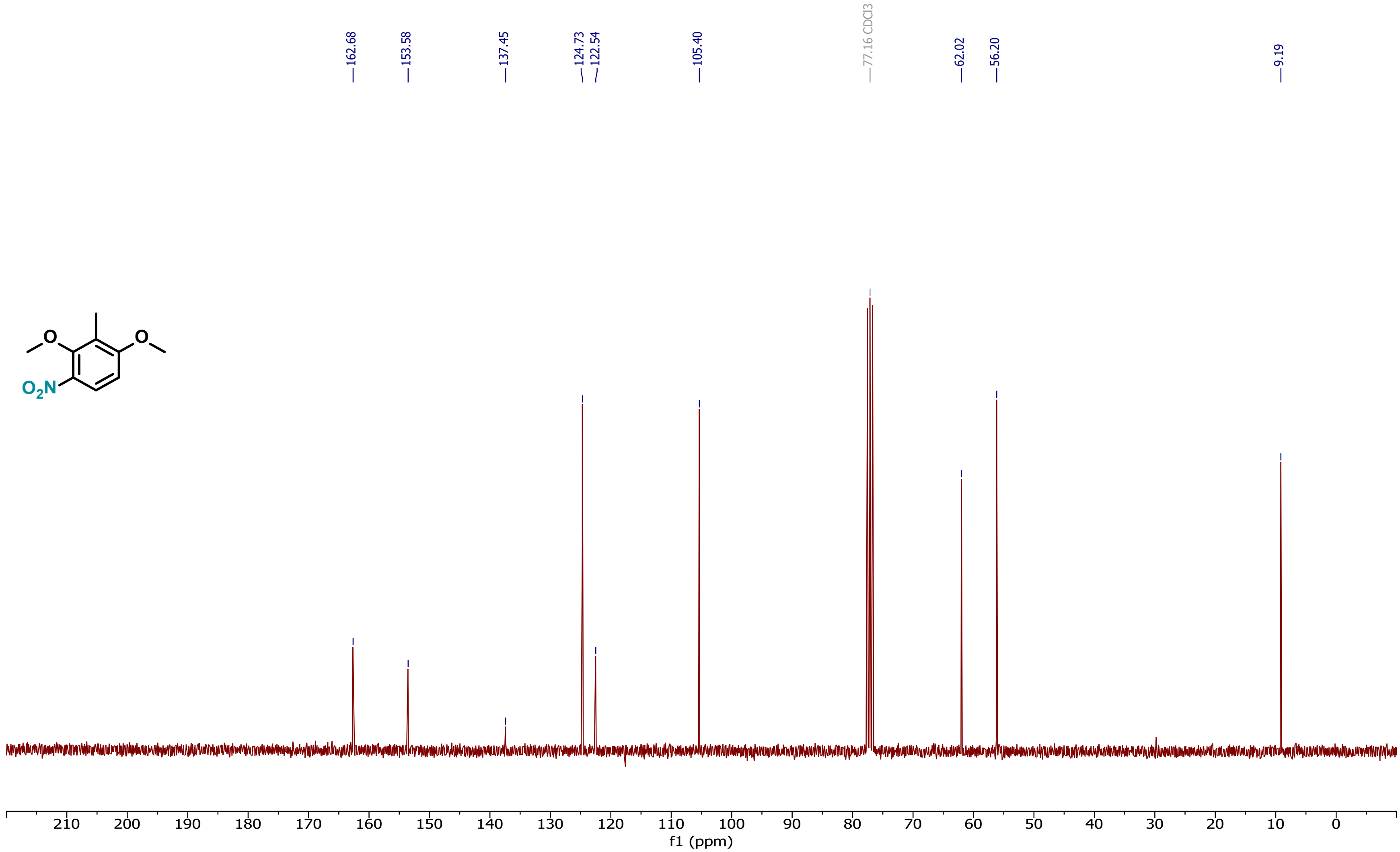
162.68
153.58
137.45
124.73
122.54
105.40
77.16 CDCl3
62.02
56.20
9.19
O
O
O2N
210
200
190
180
170
160
150
140
130
120
110
100
90
80
70
60
50
40
30
20
10
0
f1 (ppm)

1,2-dimethoxy-3-methyl-5-nitrobenzene (28a)

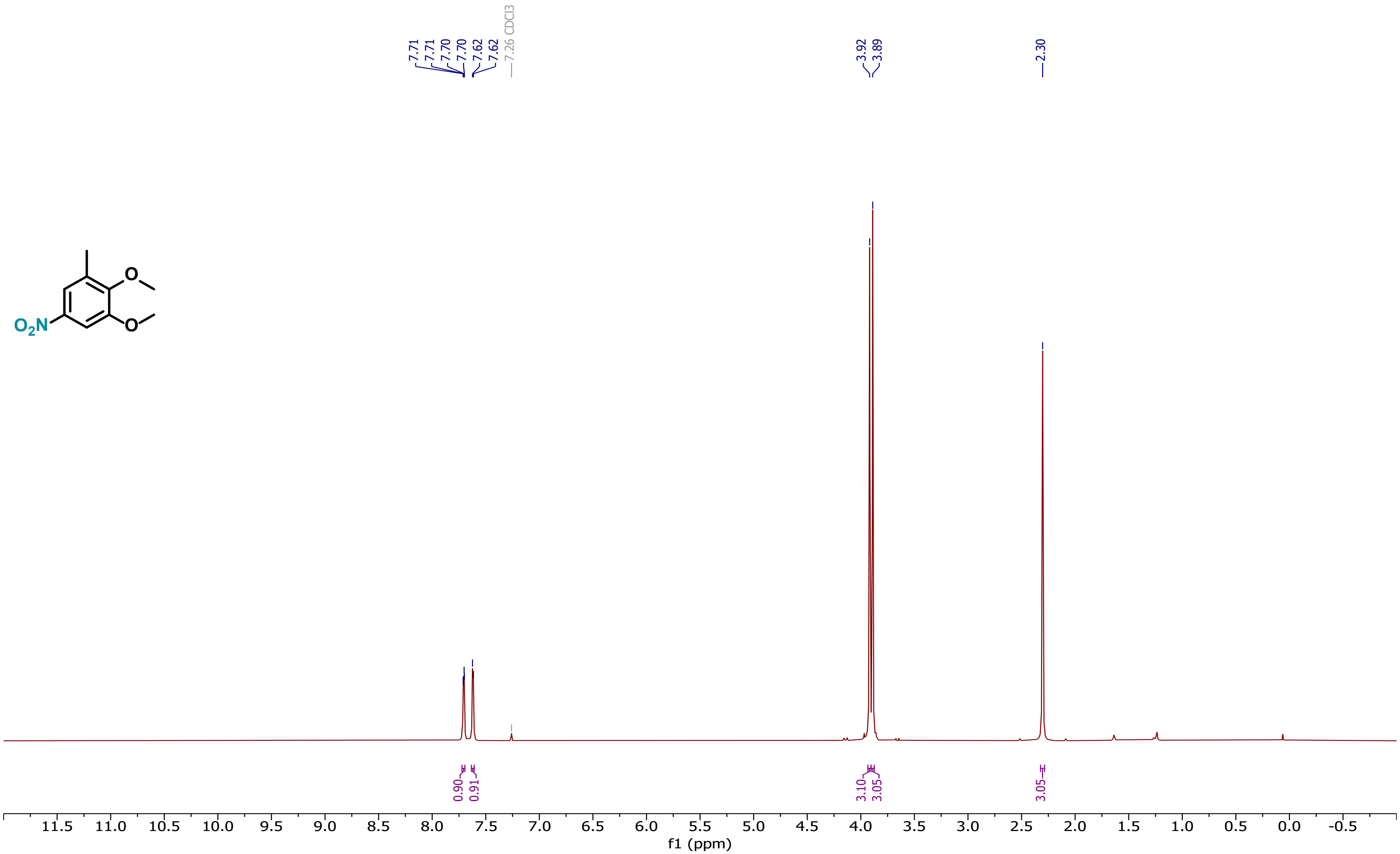

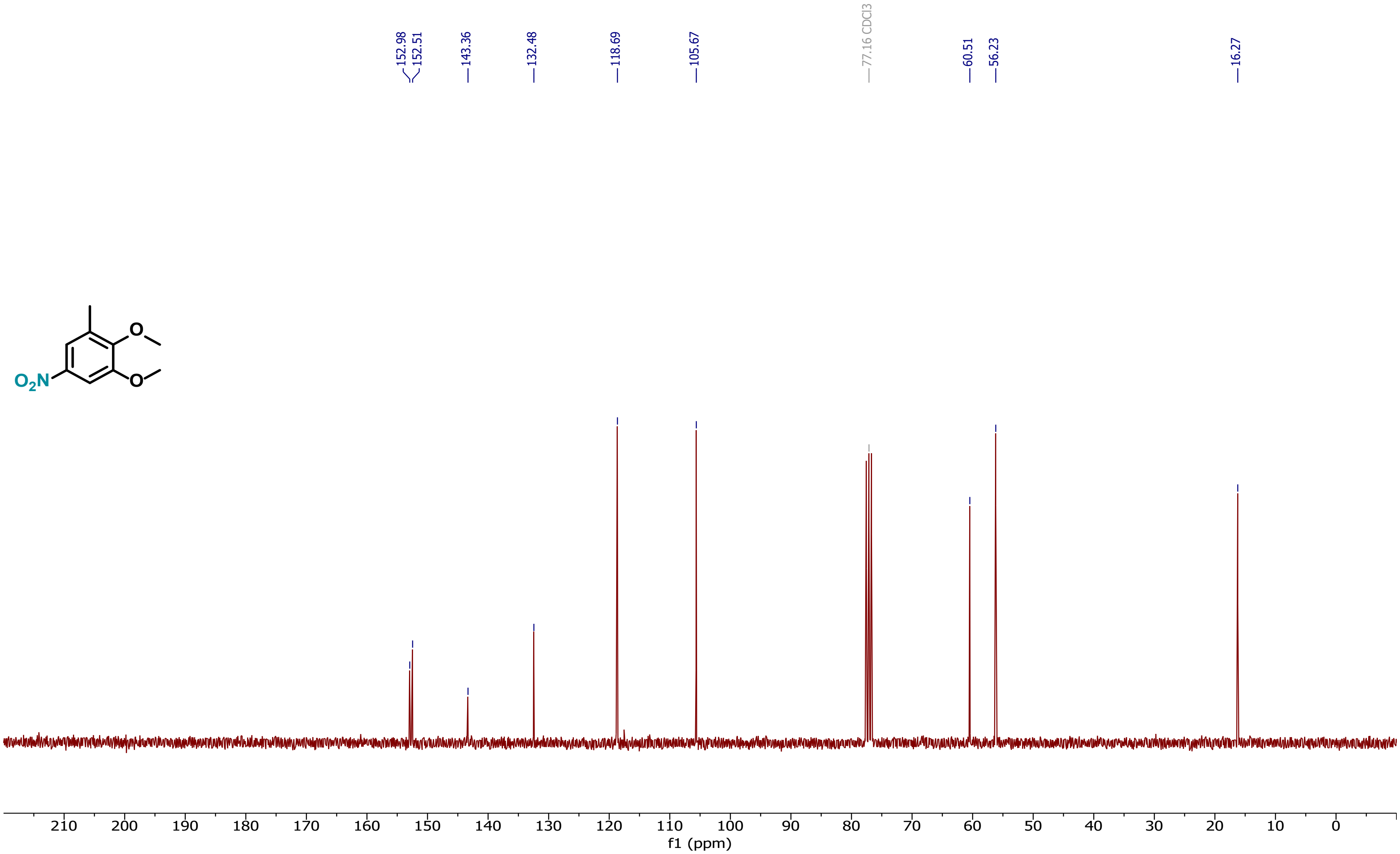

152.98
152.51
143.36
132.48
118.69
105.67
77.16 CDCl3
60.51
56.23
16.27
O
O
O2N
210
200
190
180
170
160
150
140
130
120
110
100
90
80
70
60
50
40
30
20
10
0
f1 (ppm)

1,2-dimethoxy-3-methyl-4-nitrobenzene (28b)

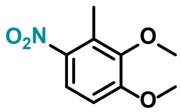

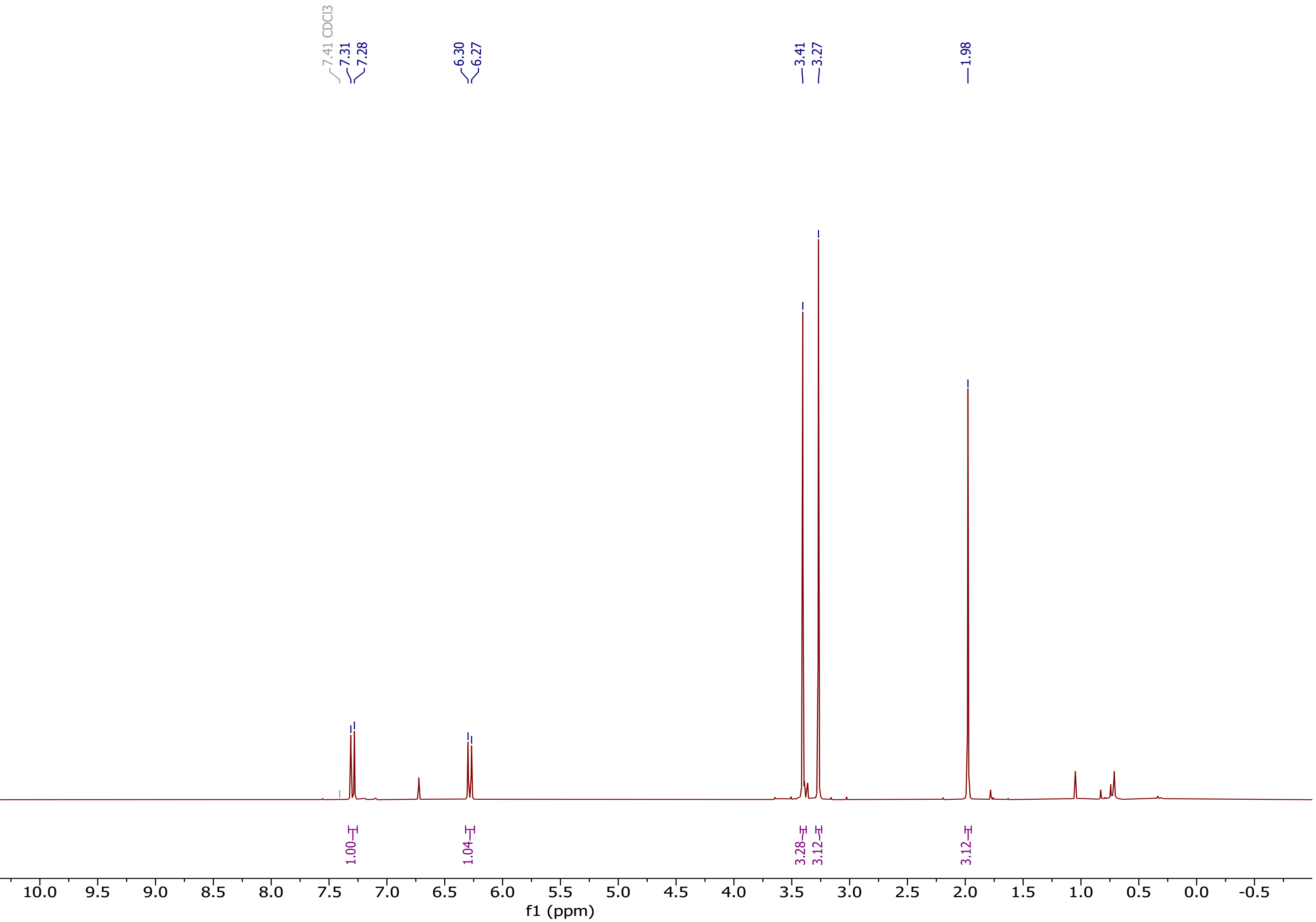

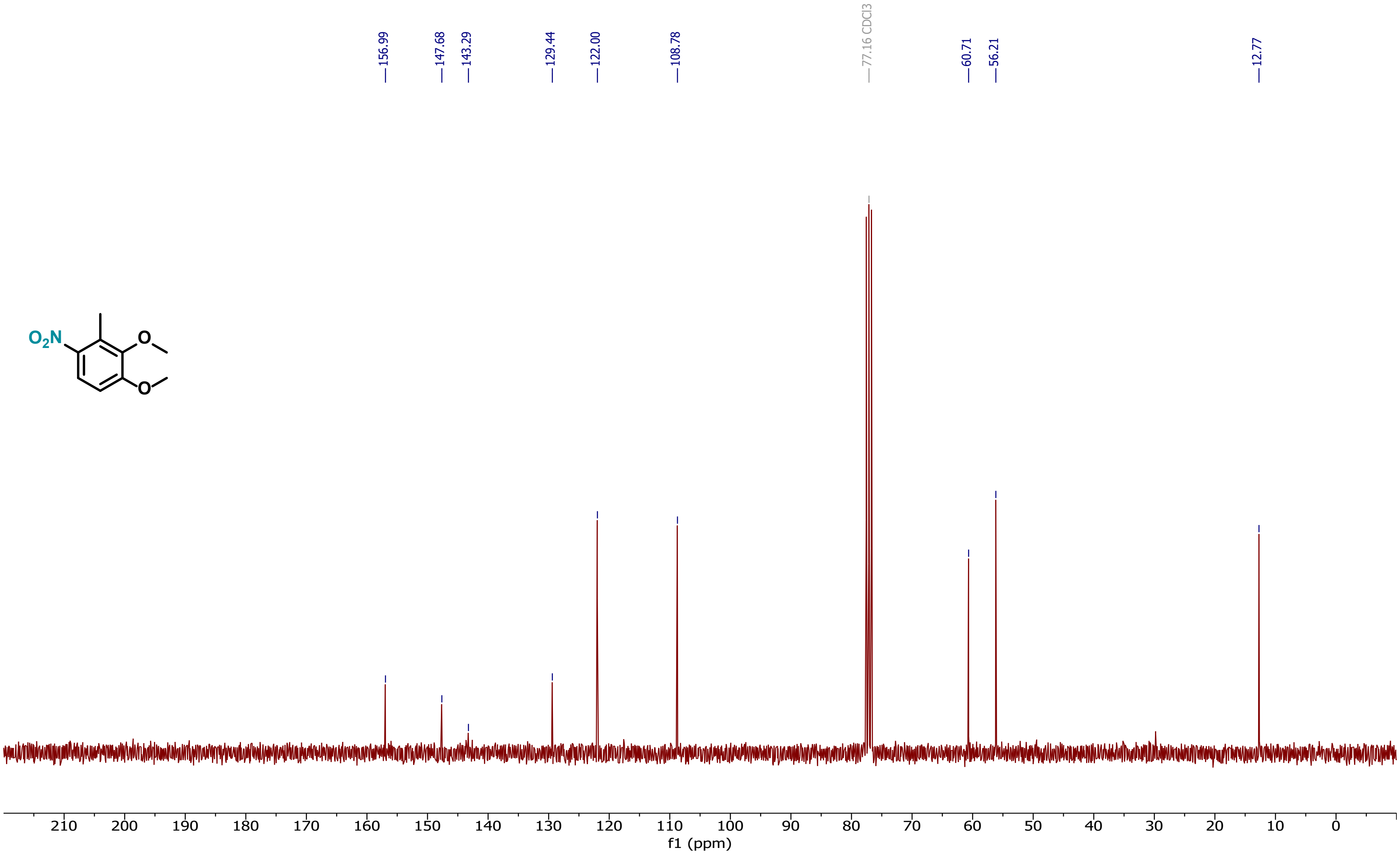

156.99
147.68
143.29
129.44
122.00
108.78
77.16 CDCl3
60.71
56.21
12.77
O2N
O
O
210 200 190 180 170 160 150 140 130 120 110 100 90 80 70 60 50 40 30 20 10 0
f1 (ppm)

5-methoxy-1,3-dimethyl-2-nitrobenzene (29a)

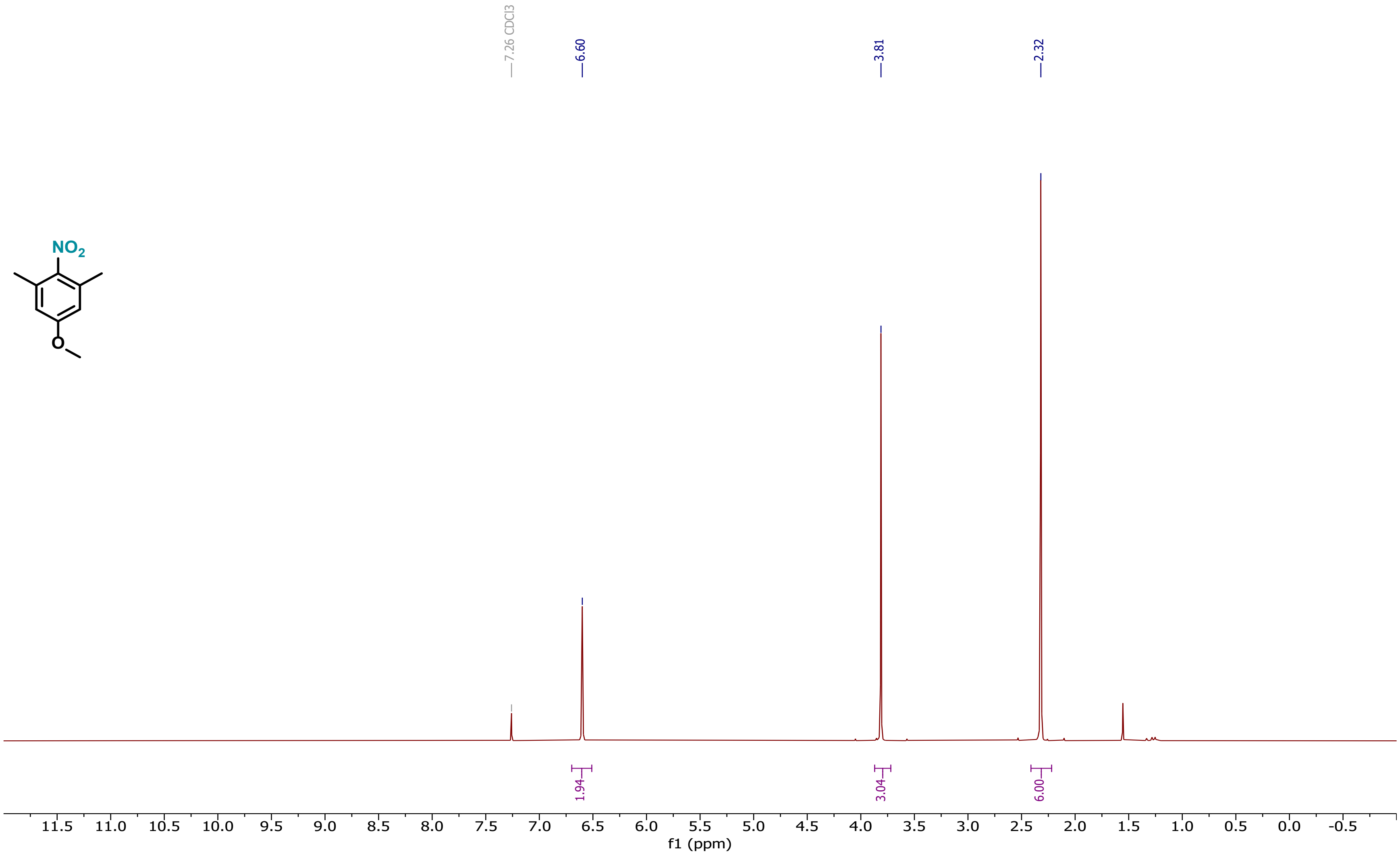

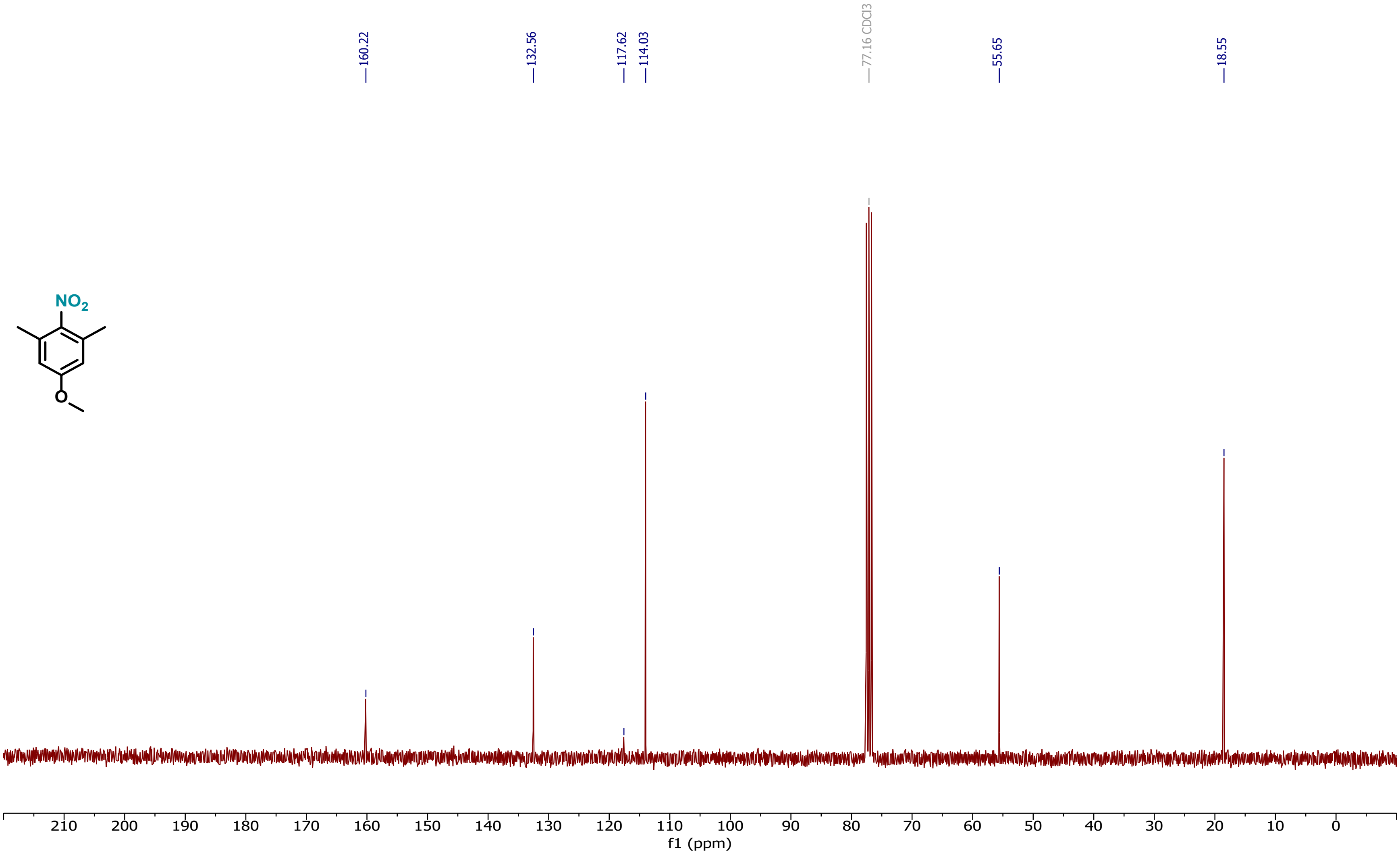
NO2
O
160.22
132.56
117.62
114.03
77.16 CDCl3
55.65
18.55
210
200
190
180
170
160
150
140
130
120
110
100
90
80
70
60
50
40
30
20
10
0
f1 (ppm)

1-methoxy-3,5-dimethyl-2-nitrobenzene 5-methoxy-1,3-dimethyl-2-nitrobenzene (29a)

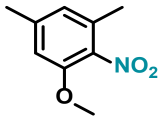

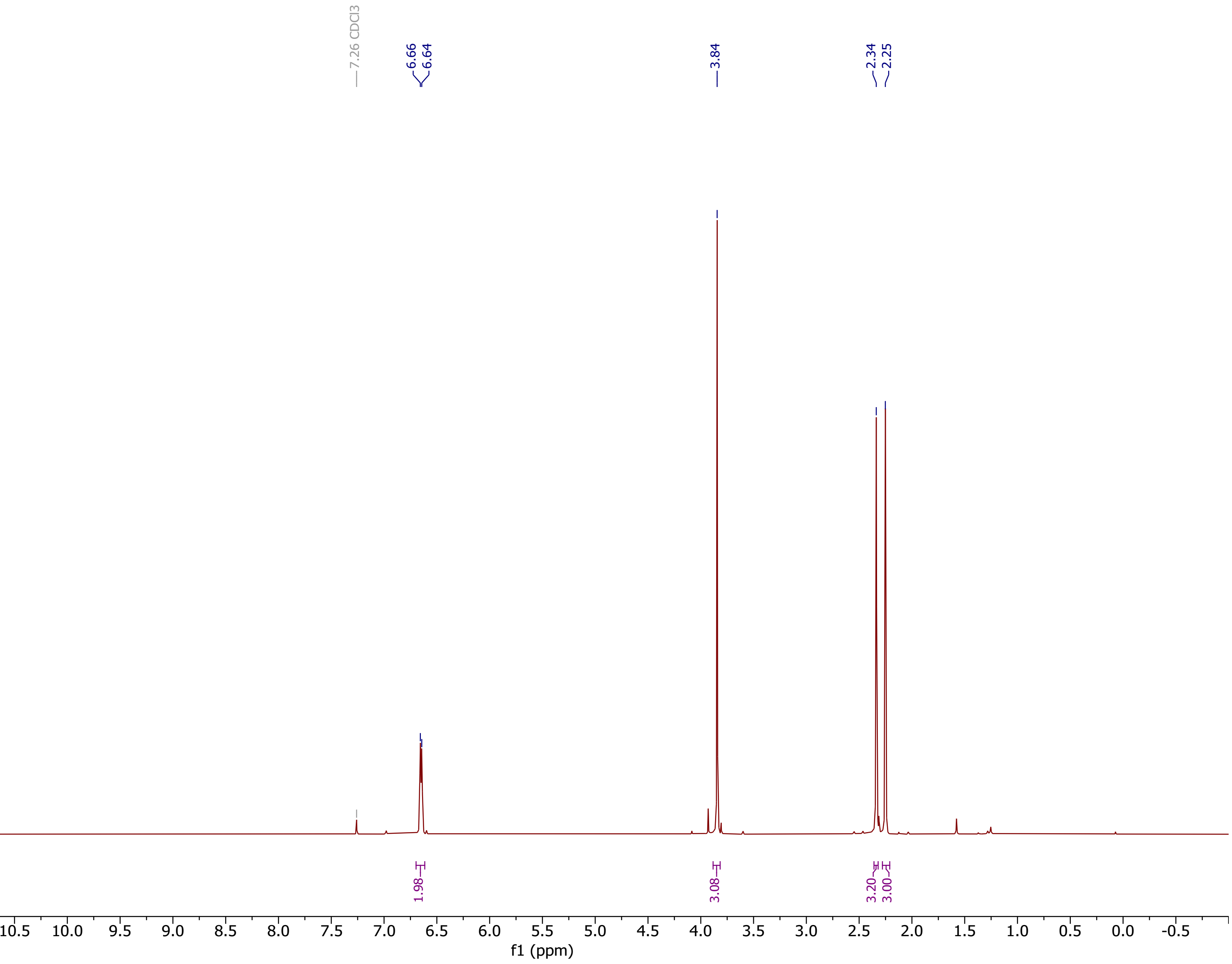

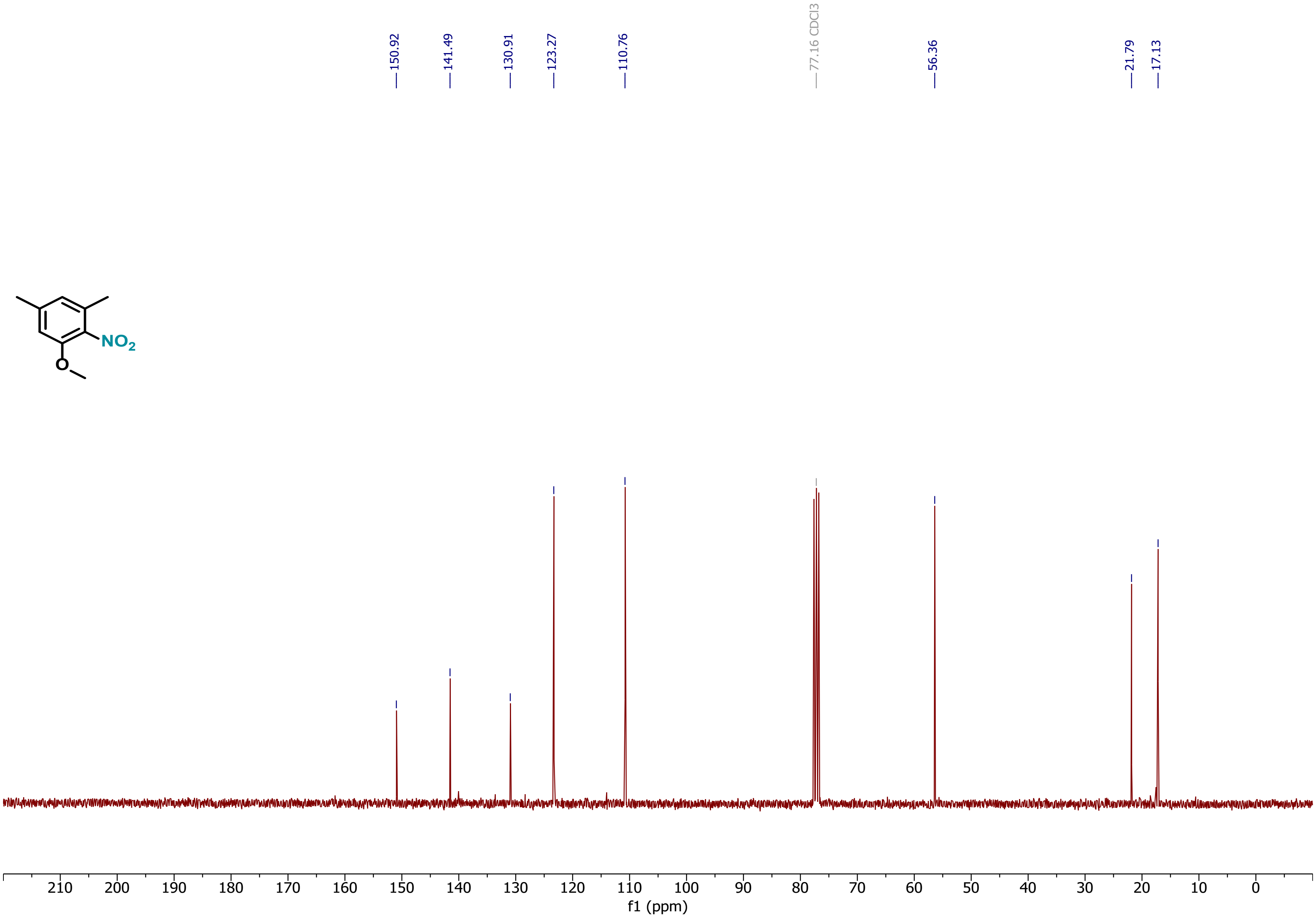
150.92
141.49
130.91
123.27
110.76
77.16 CDCl3
56.36
21.79
17.13
NO2
O
210 200 190 180 170 160 150 140 130 120 110 100 90 80 70 60 50 40 30 20 10 0
f1 (ppm)

1-methyl-4-nitrobenzene and 1-methyl-2-nitrobenzene (30)

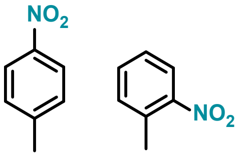

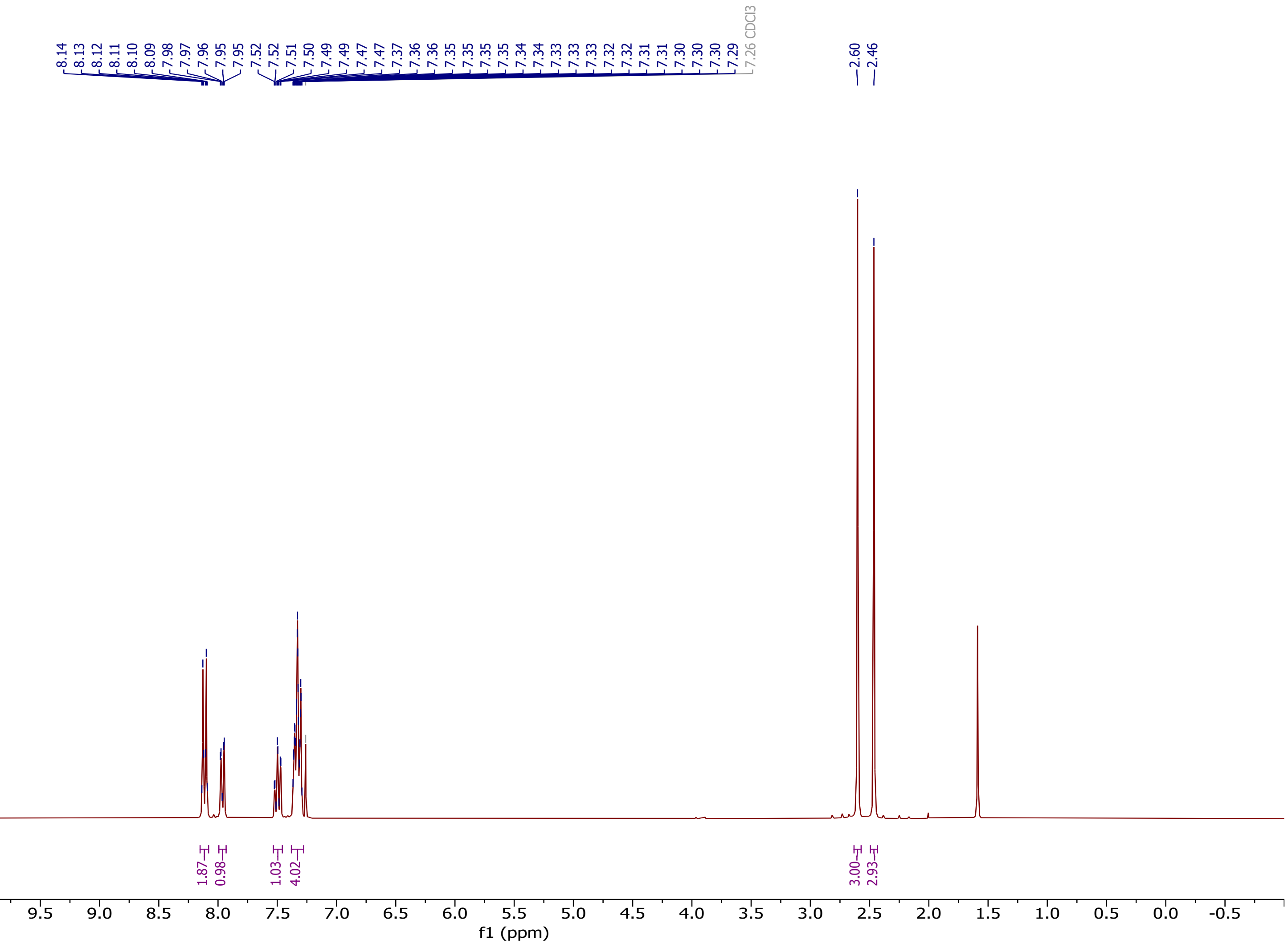

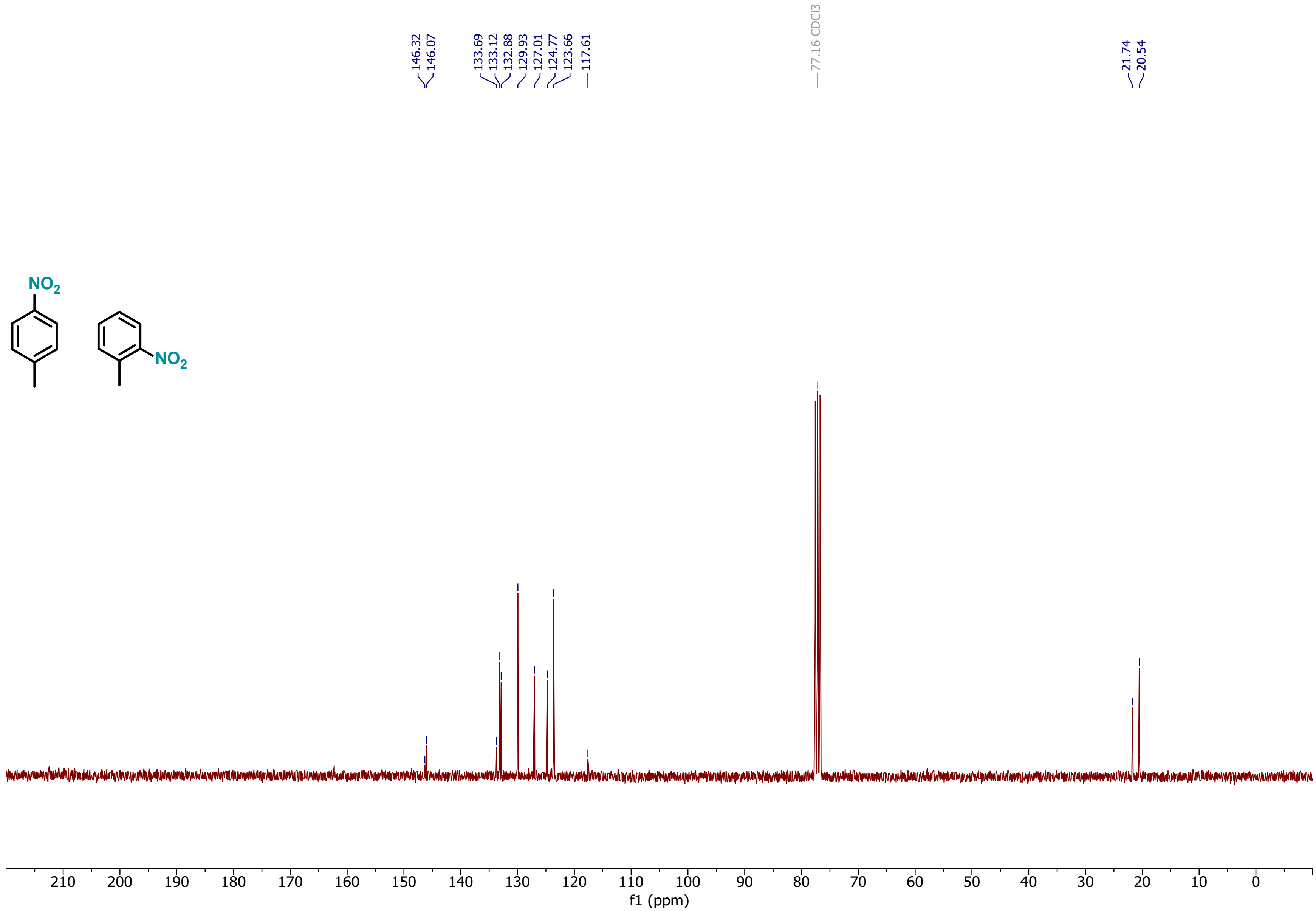

146.32
146.07
133.69
133.12
132.88
129.93
127.01
124.77
123.66
117.61
77.16 CDCl3
21.74
20.54
NO2
NO2
210
200
190
180
170
160
150
140
130
120
110
100
90
80
70
60
50
40
30
20
10
0
f1 (ppm)

1,3,5-trimethyl-2-nitrobenzene (31)

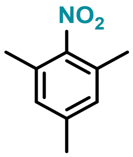

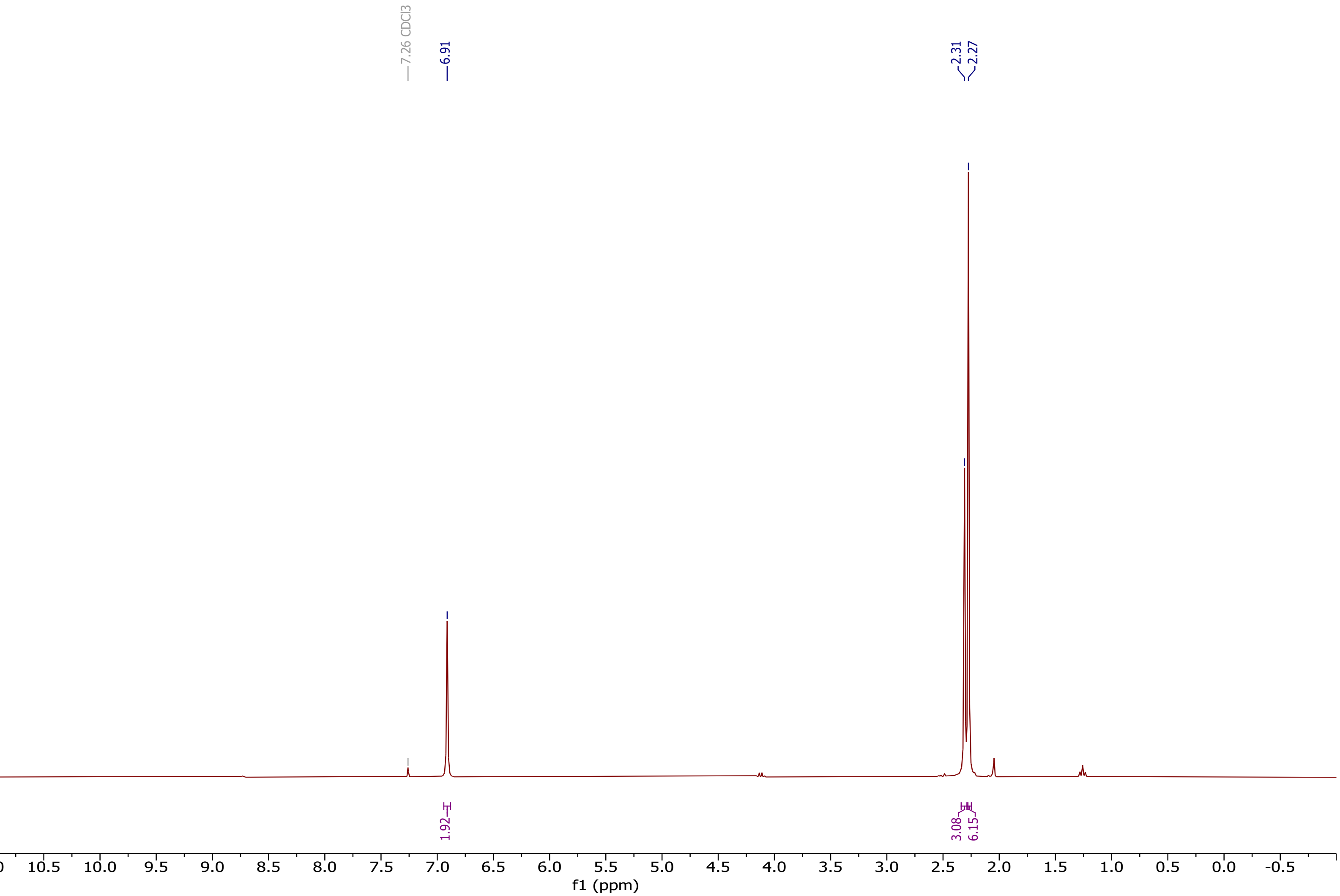

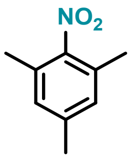
NO2

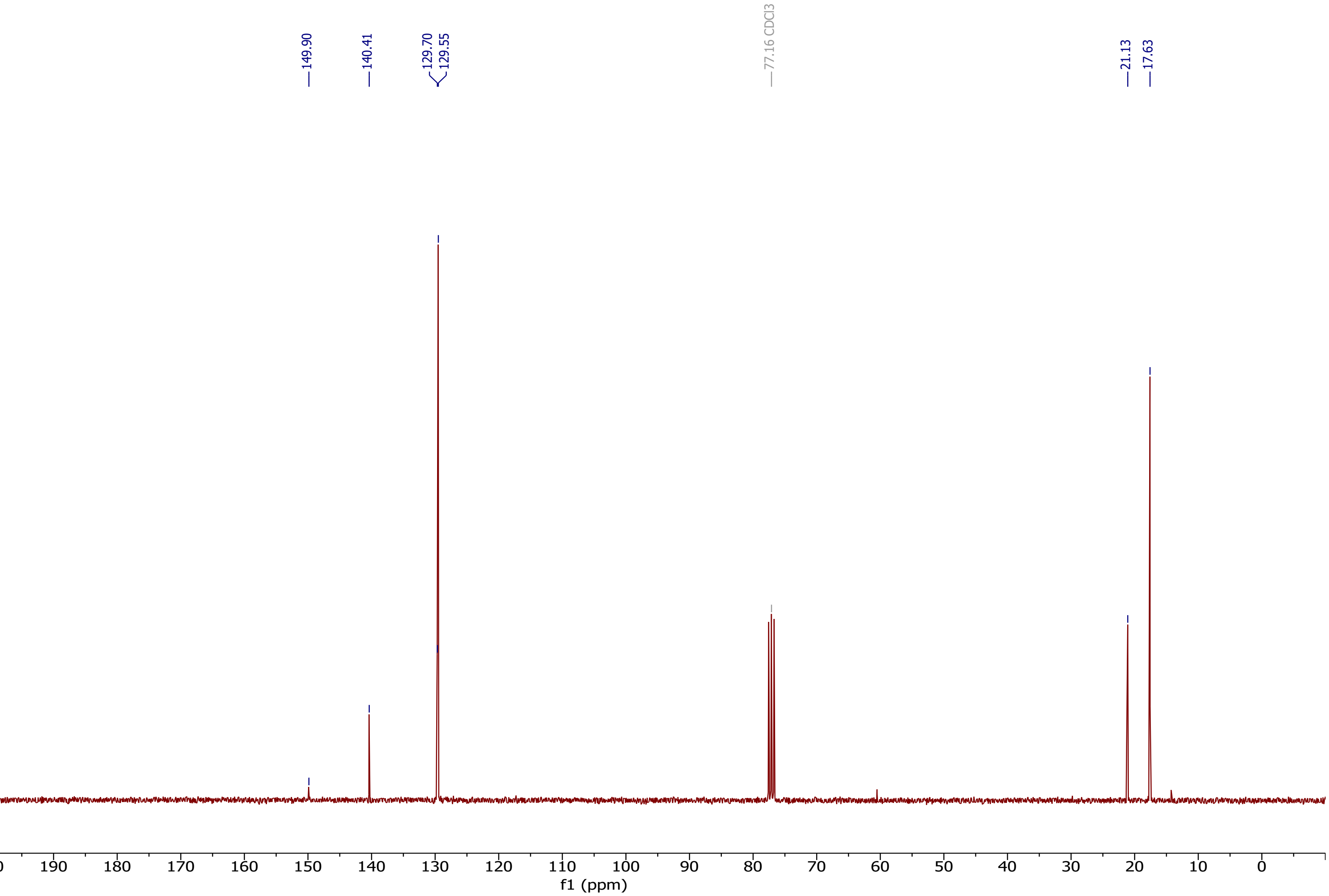
149.90
140.41
129.70
129.55
77.16 CDCl3
21.13
17.63
210
200
190
180
170
160
150
140
130
120
110
100
90
80
70
60
50
40
30
20
10
0
f1 (ppm)

1,3,5-triethyl-2-nitrobenzene (32)

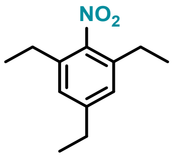

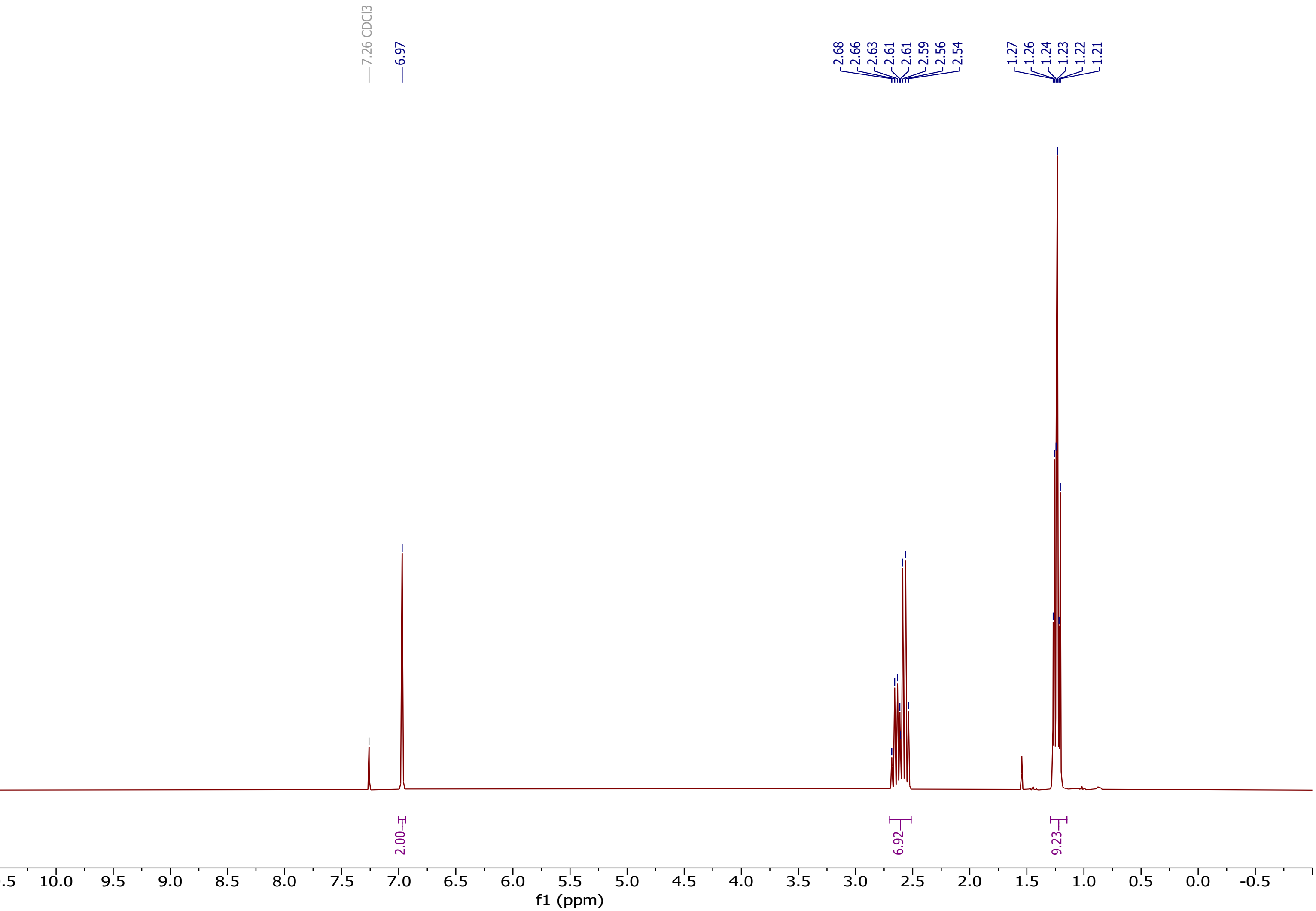

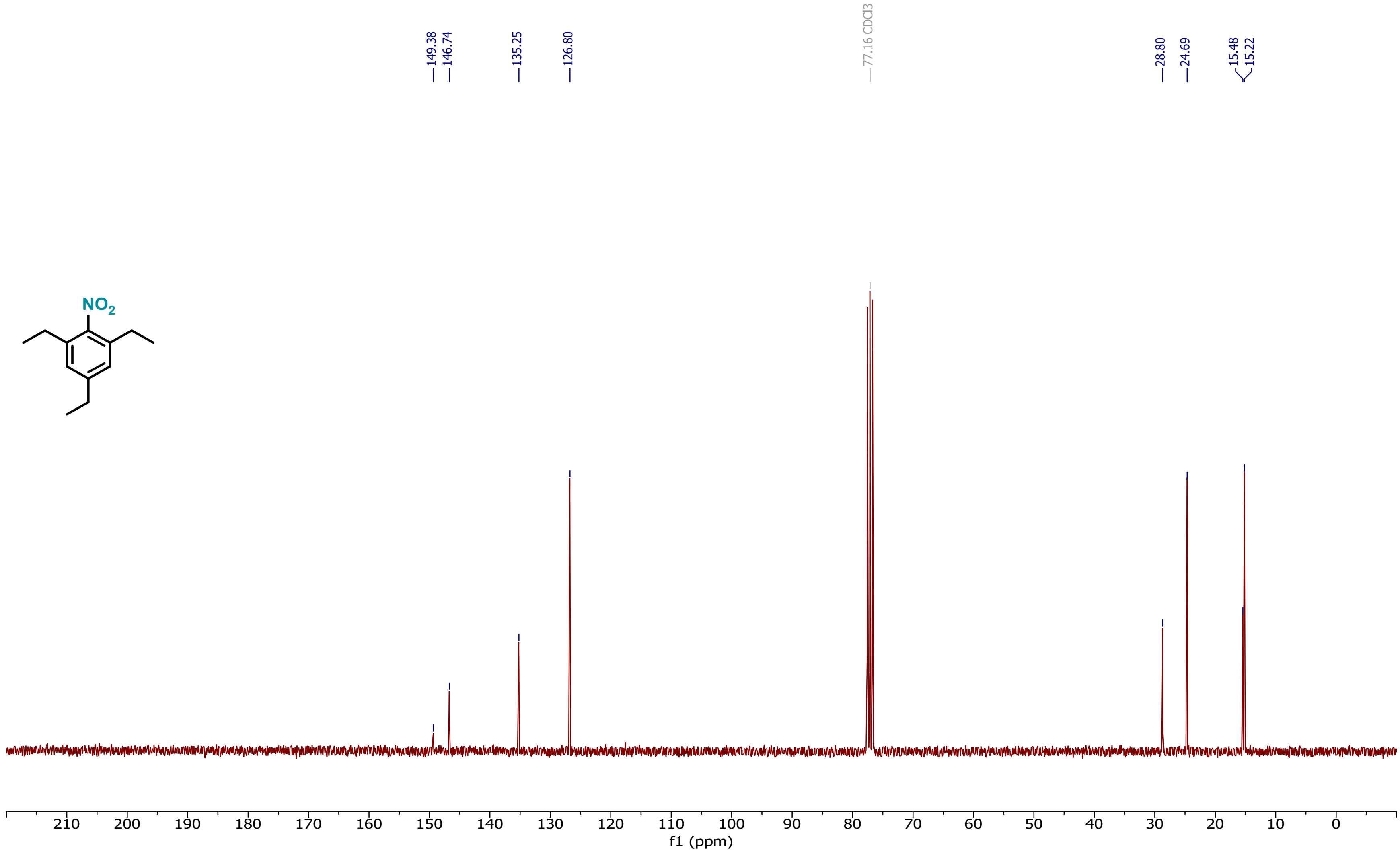

149.38
146.74
135.25
126.80
77.16 CDCl3
28.80
24.69
15.48
15.22
210
200
190
180
170
160
150
140
130
120
110
100
90
80
70
60
50
40
30
20
10
0
f1 (ppm)

1-bromo-4-methoxy-2-methyl-5-nitrobenzene (33)

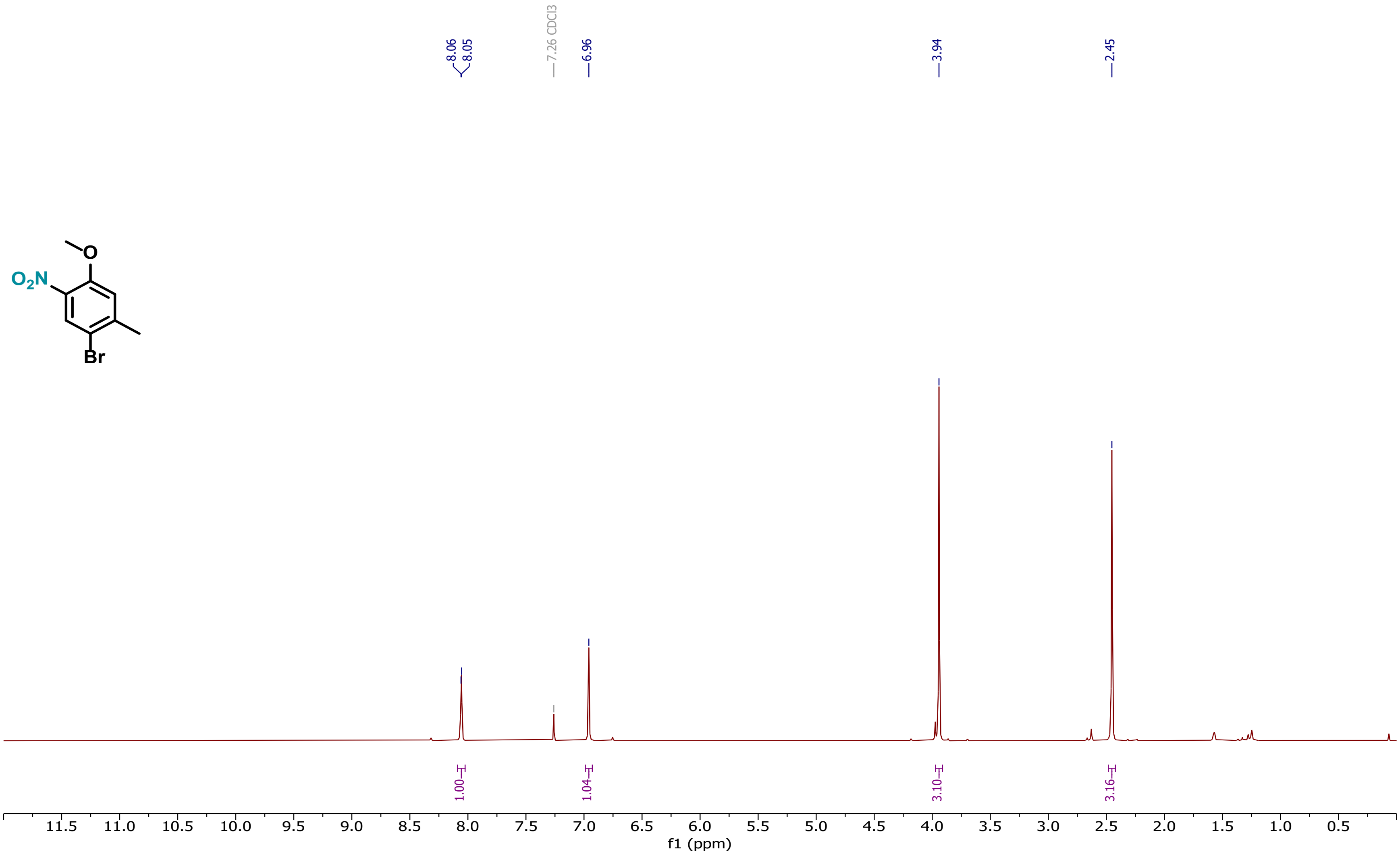

1-fluoro-2,5-dimethoxy-4-nitrobenzene (34)

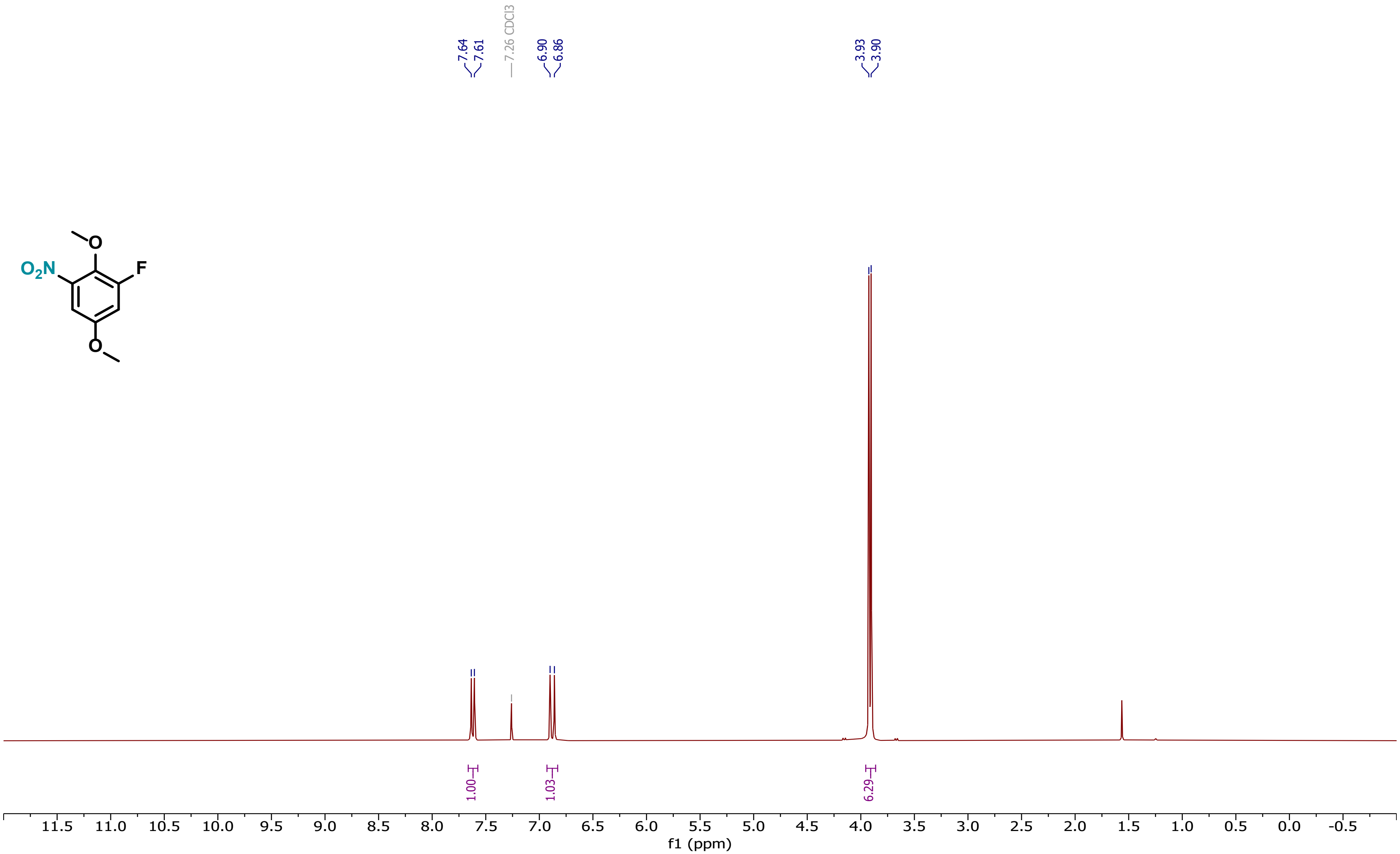

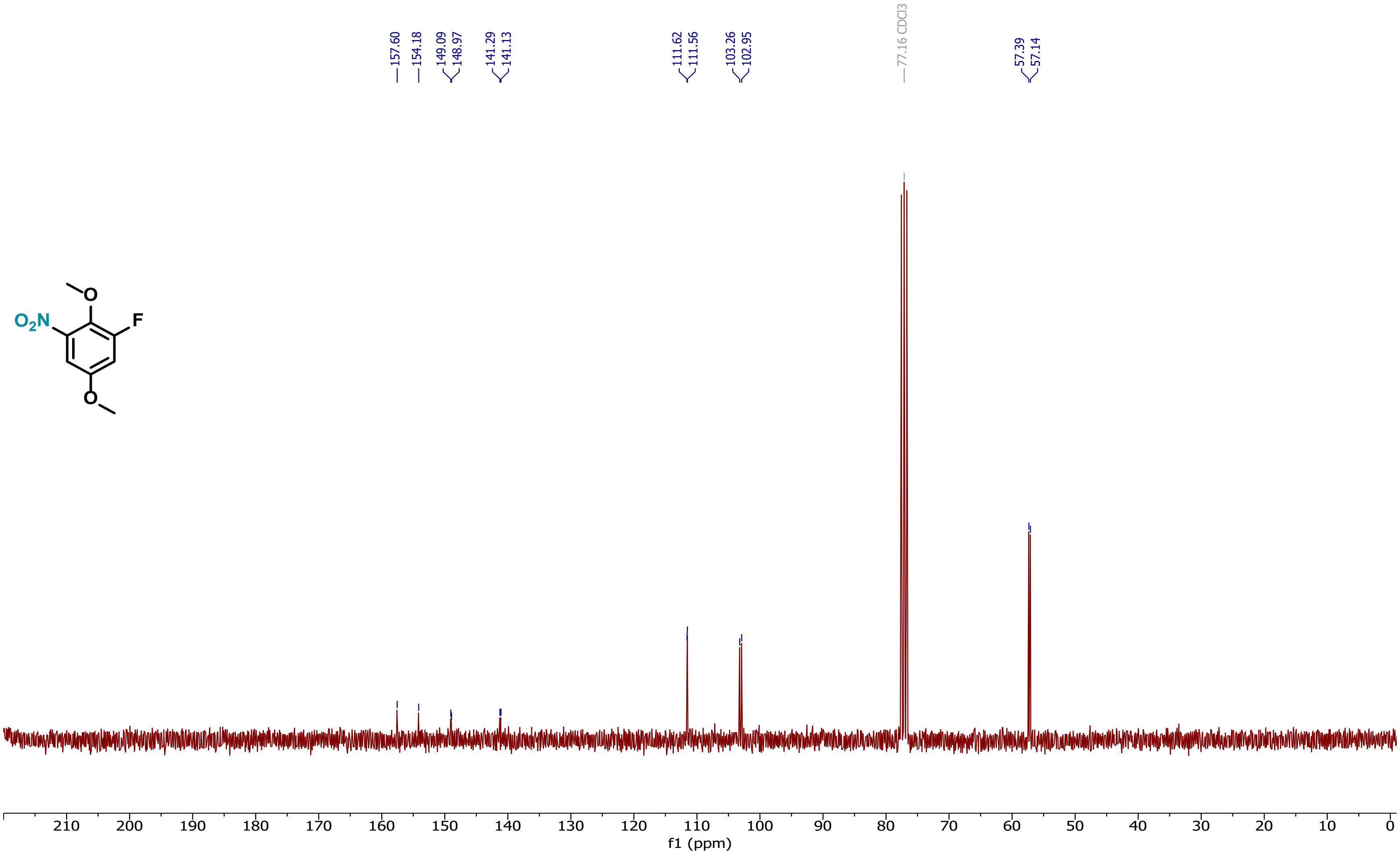
157.60
154.18
149.09
148.97
141.29
141.13
111.62
111.56
103.26
102.95
77.16 CDCl3
57.39
57.14
O
O2N
F
O
210
200
190
180
170
160
150
140
130
120
110
100
90
80
70
60
50
40
30
20
10
0
f1 (ppm)

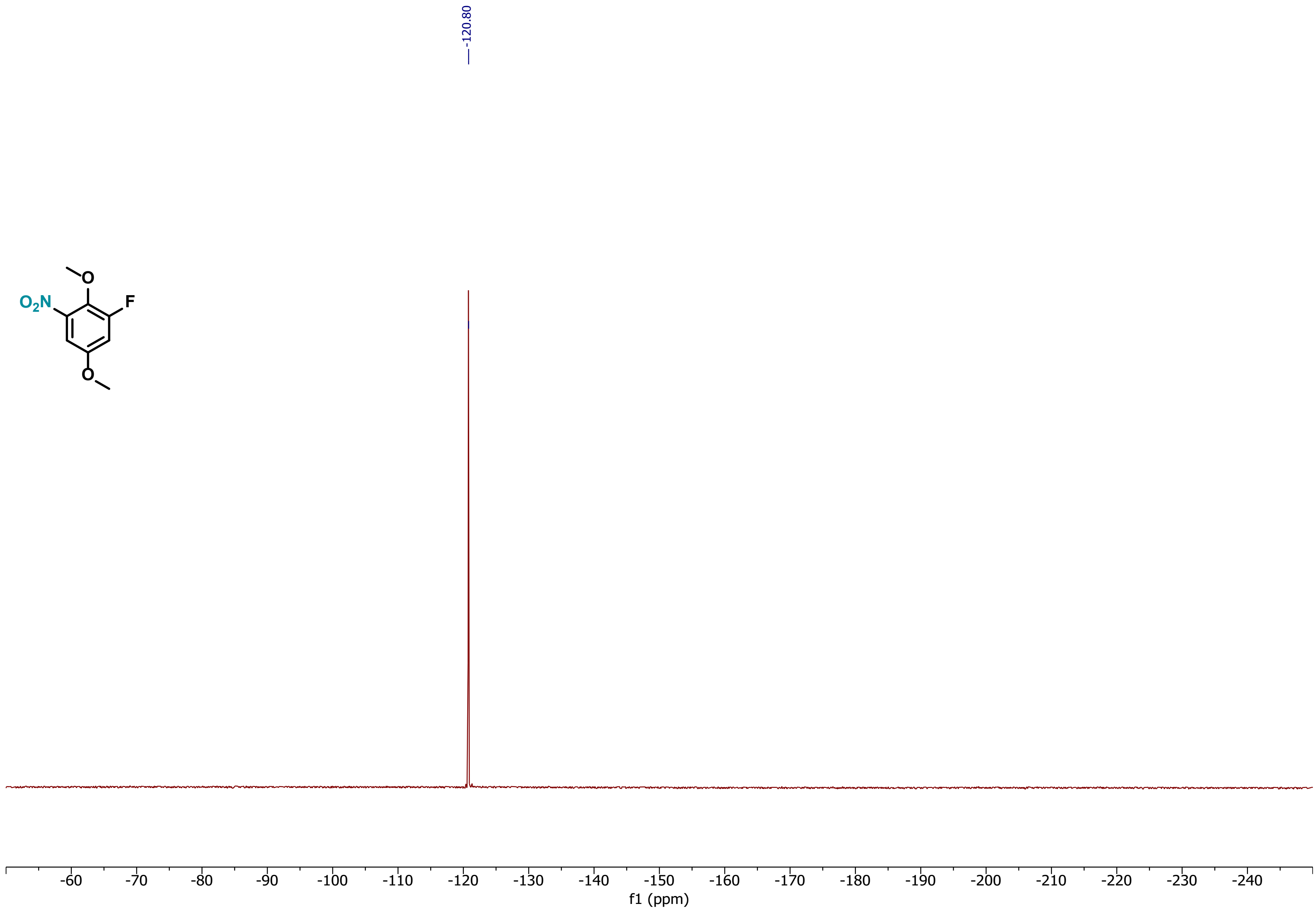

-120.80
O$_2$N
O
F
O
-60
-70
-80
-90
-100
-110
-120
-130
-140
-150
-160
-170
-180
-190
-200
-210
-220
-230
-240
f1 (ppm)

2-bromo-1-methoxy-4-nitrobenzene (35a)

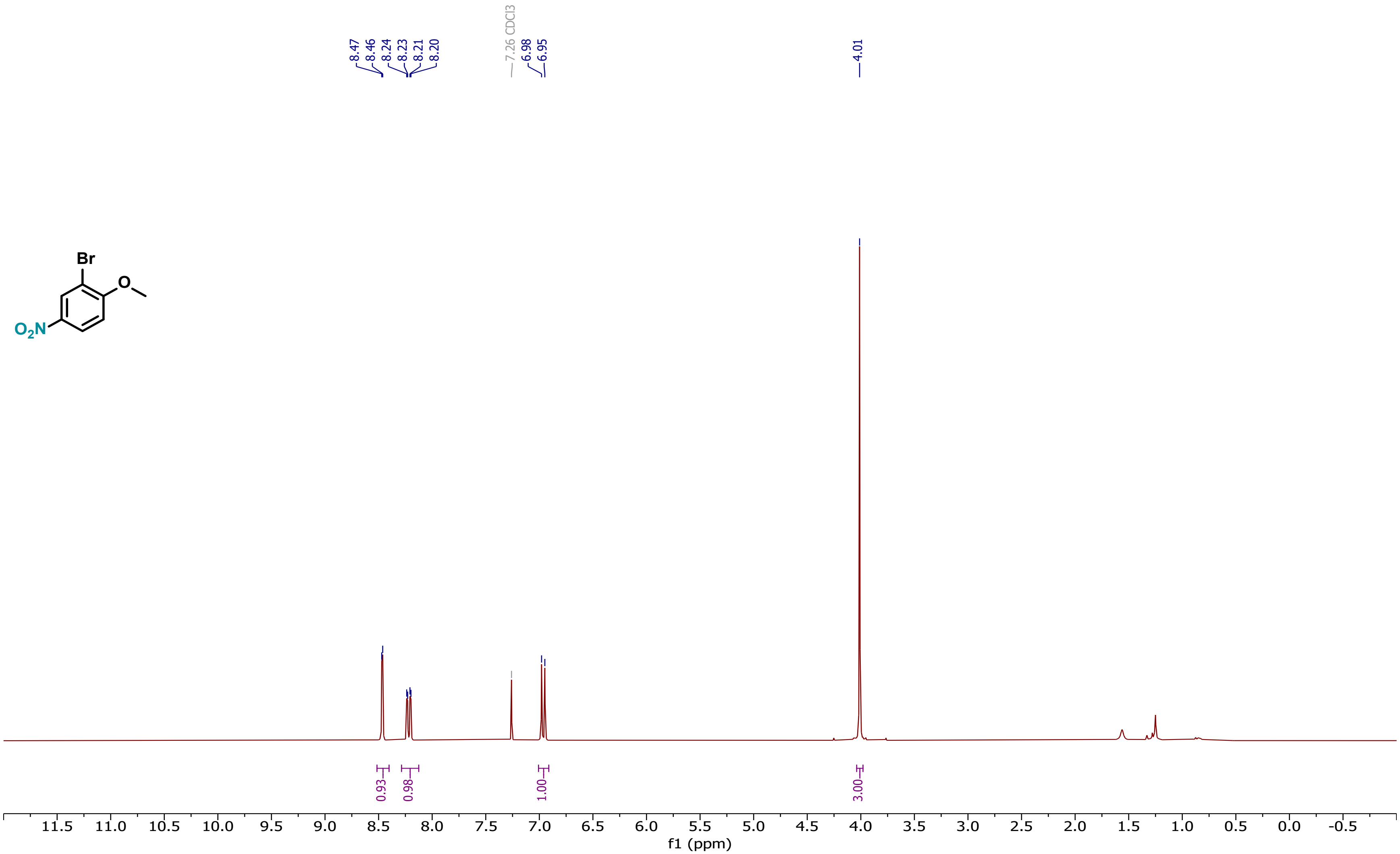

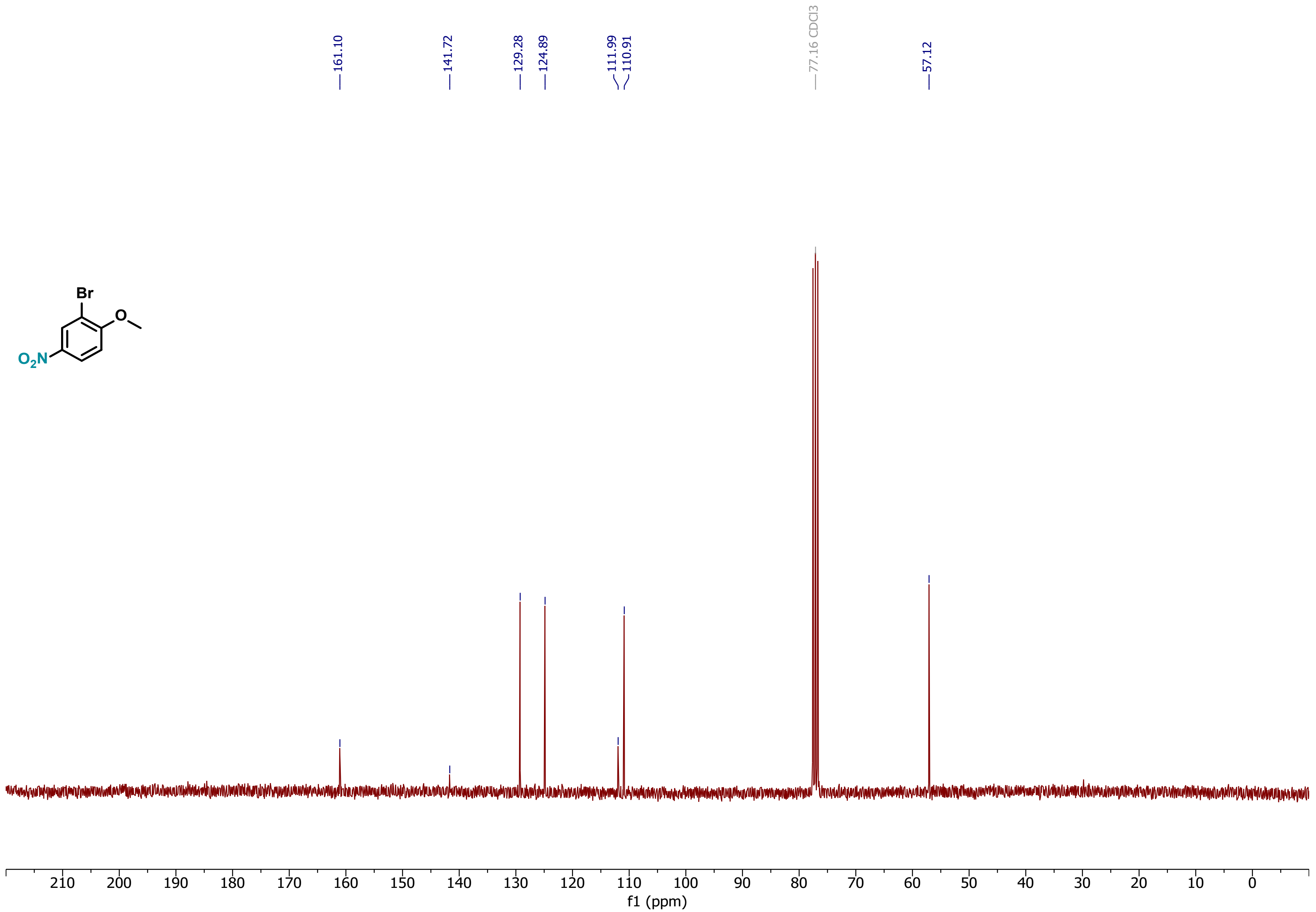
161.10
141.72
129.28
124.89
111.99
110.91
77.16 CDCl3
57.12
Br
O
O2N
210
200
190
180
170
160
150
140
130
120
110
100
90
80
70
60
50
40
30
20
10
0
f1 (ppm)

1-bromo-2-methoxy-3-nitrobenzene (35b)

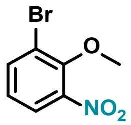

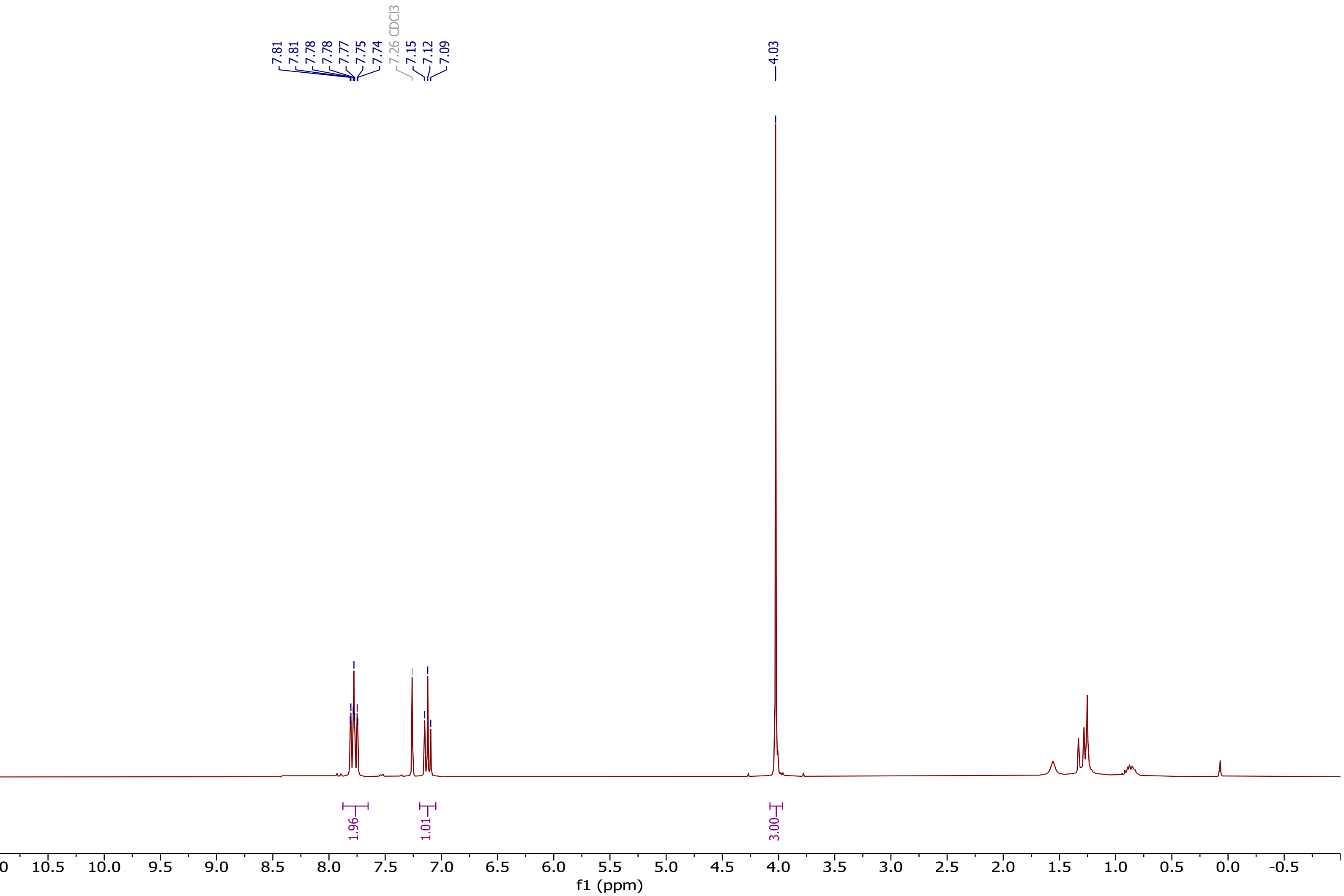

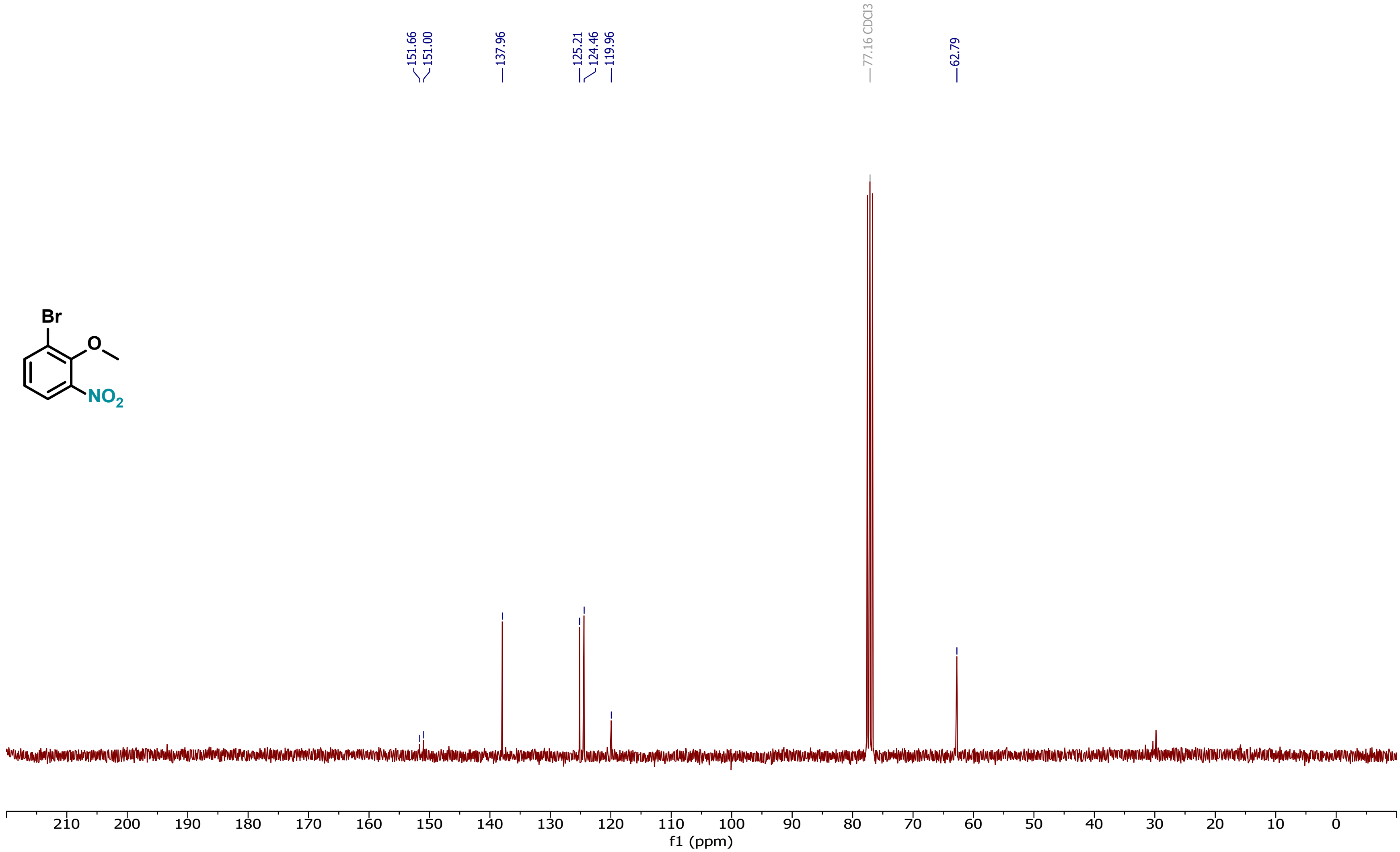
151.66
151.00
137.96
125.21
124.46
119.96
77.16 CDCl3
62.79
Br
O
NO2
210
200
190
180
170
160
150
140
130
120
110
100
90
80
70
60
50
40
30
20
10
0
f1 (ppm)

1-bromo-4-(tert-butyl)-2-nitrobenzene (36)

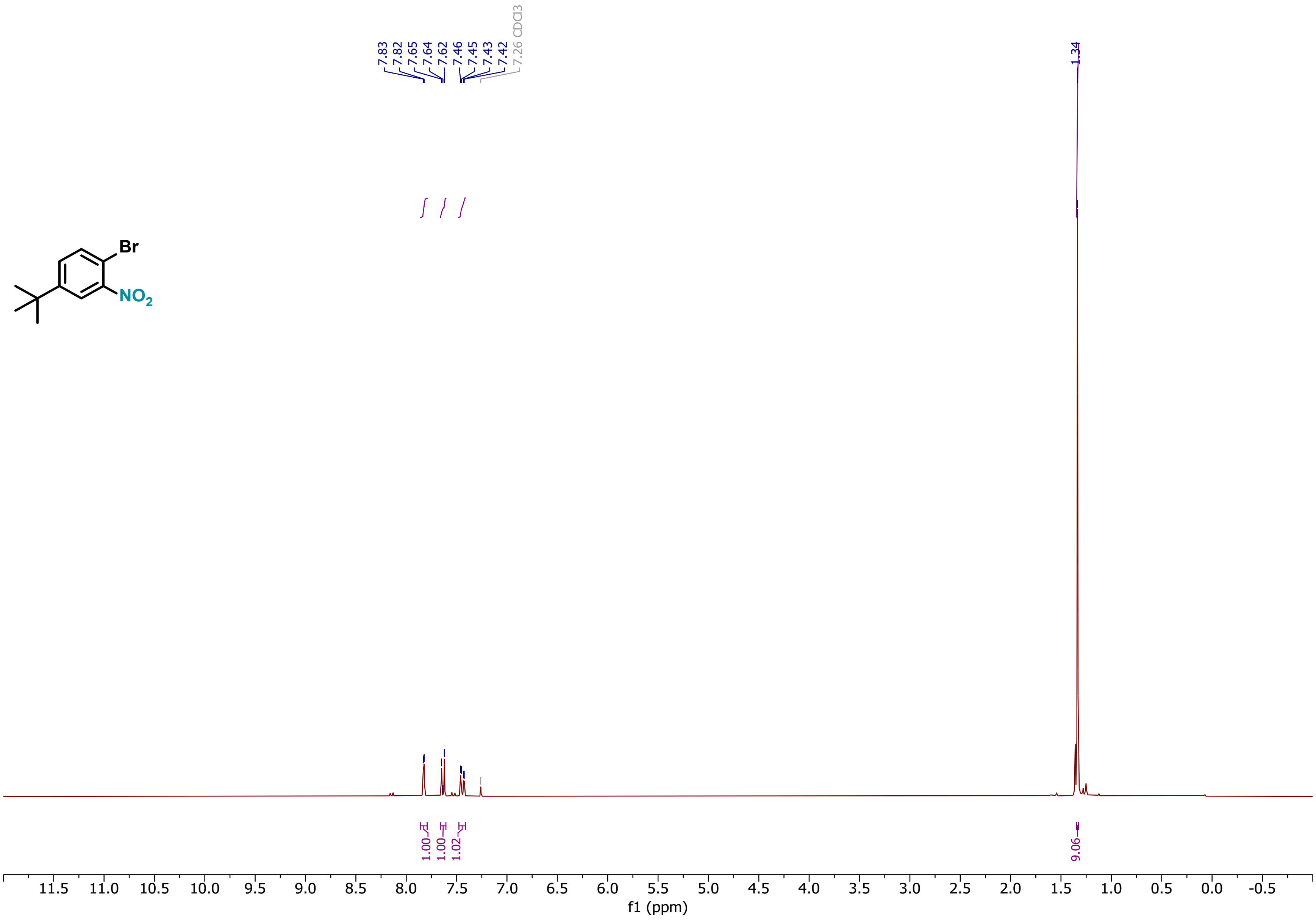

2-iodo-1,3-dimethoxy-4-nitrobenzene (37)

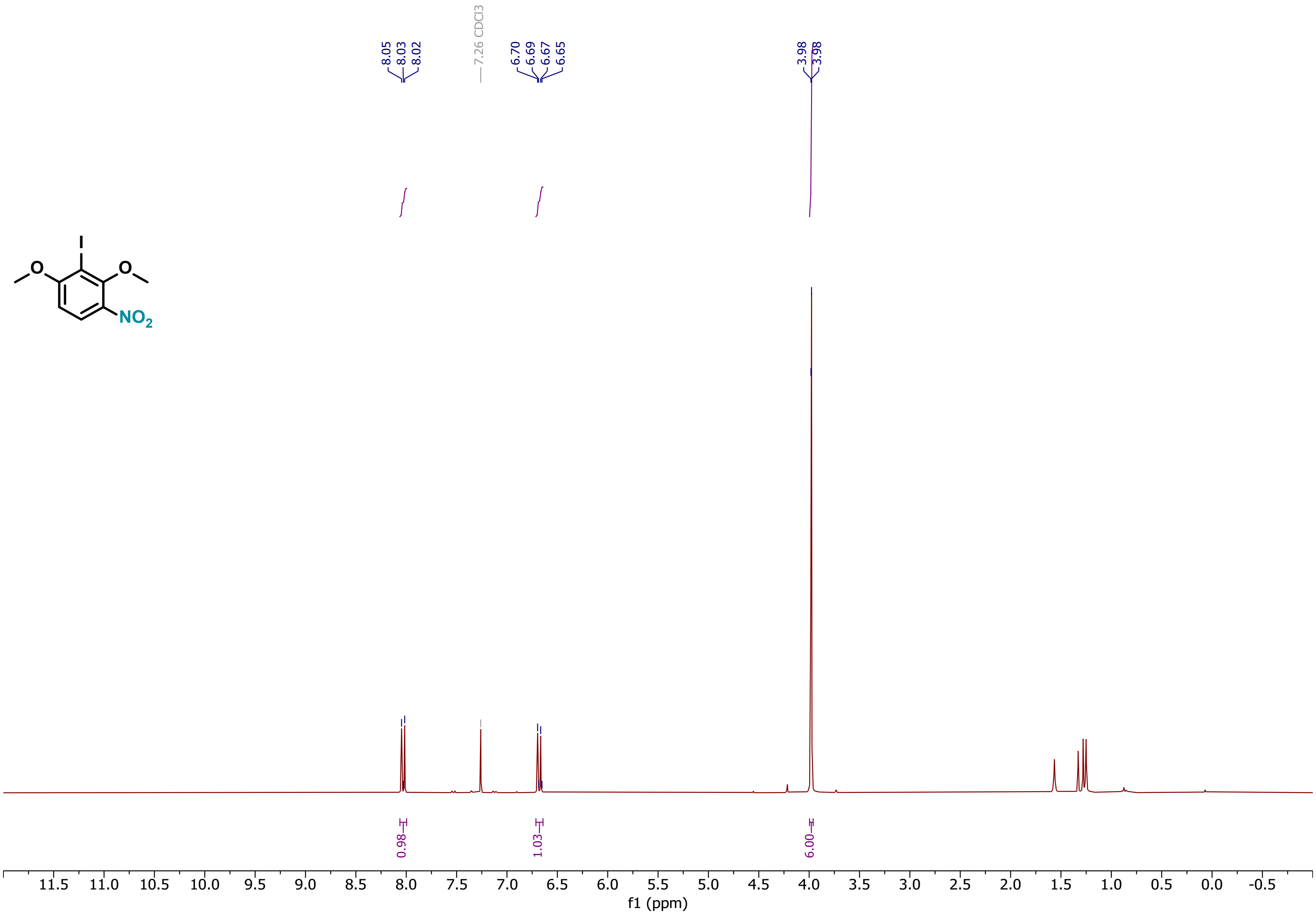

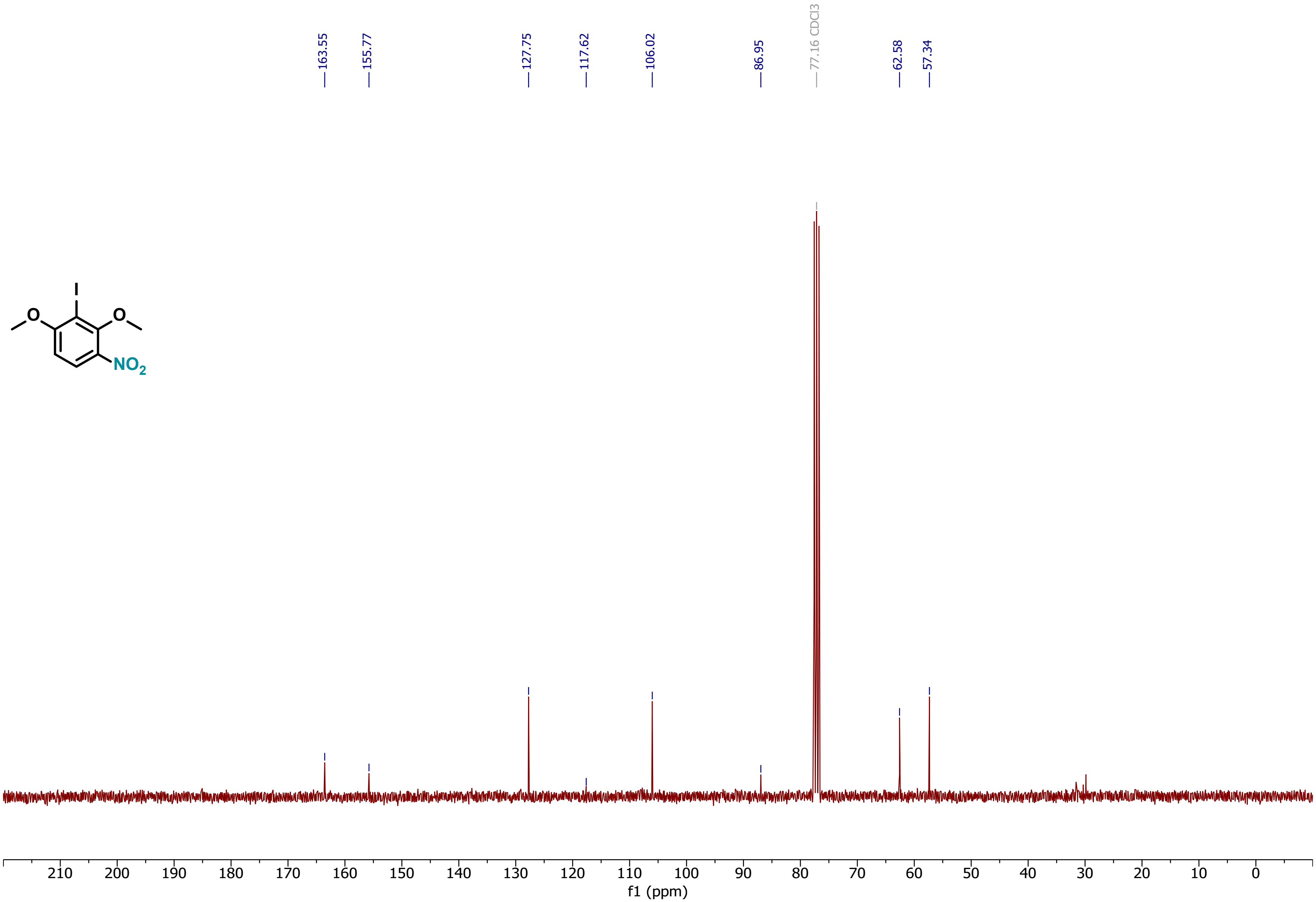
I
O
O
NO2
163.55
155.77
127.75
117.62
106.02
86.95
77.16 CDCl3
62.58
57.34
210 200 190 180 170 160 150 140 130 120 110 100 90 80 70 60 50 40 30 20 10 0
f1 (ppm)

4-methoxy-1-nitro-2-(trifluoromethyl)benzene (38a)

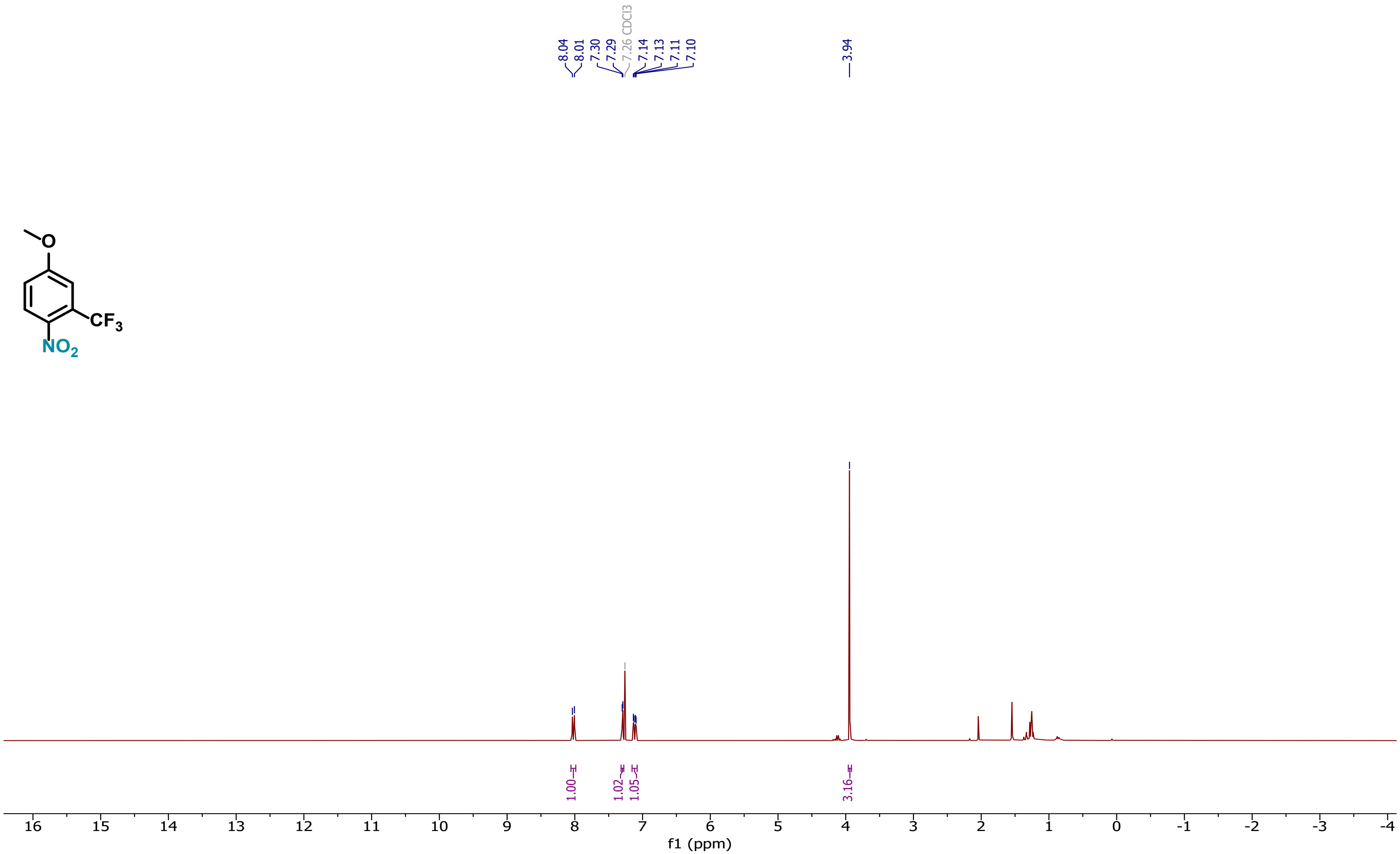

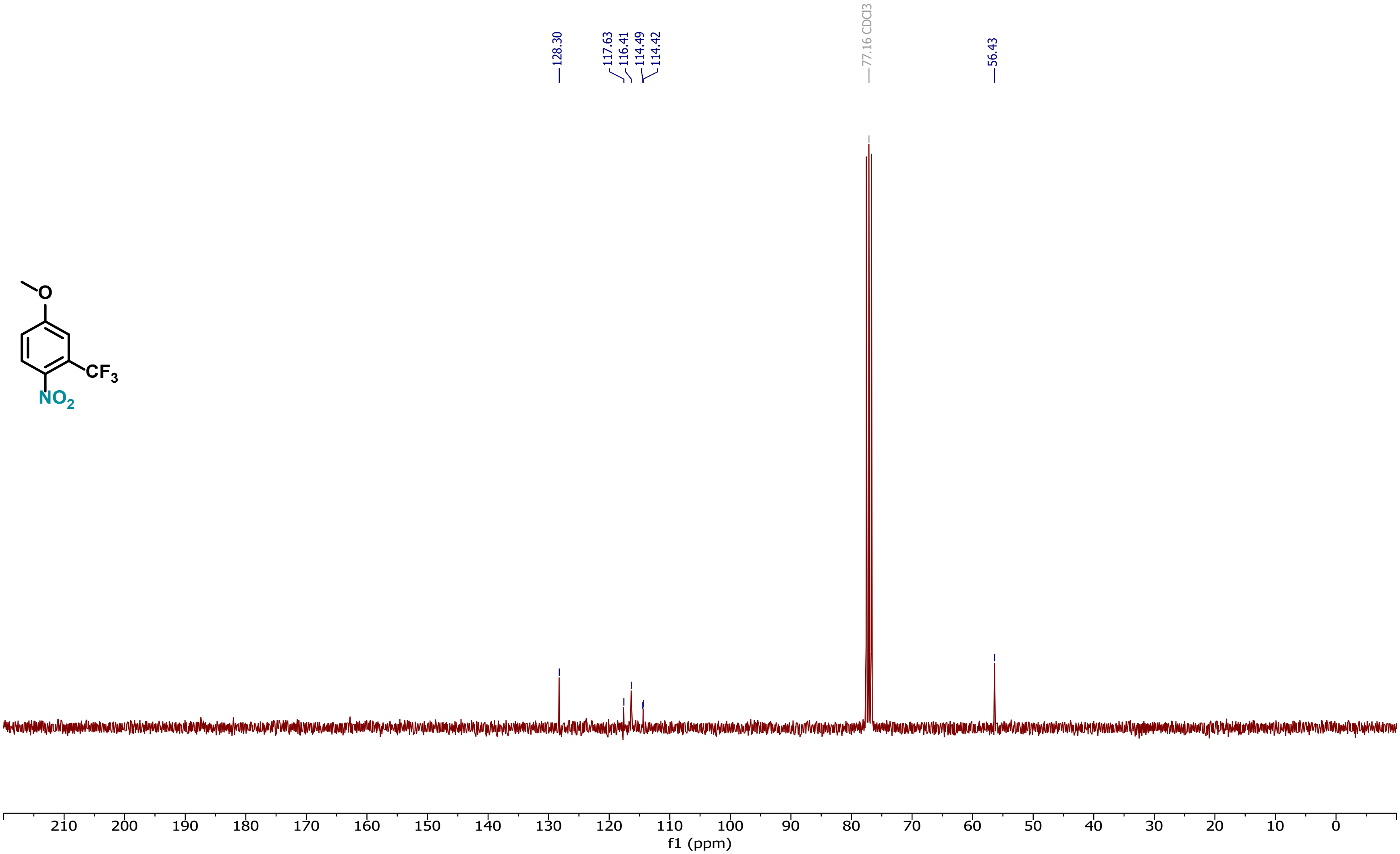

128.30
117.63
116.41
114.49
114.42
77.16 CDCl3
56.43
O
CF3
NO2
210 200 190 180 170 160 150 140 130 120 110 100 90 80 70 60 50 40 30 20 10 0
f1 (ppm)

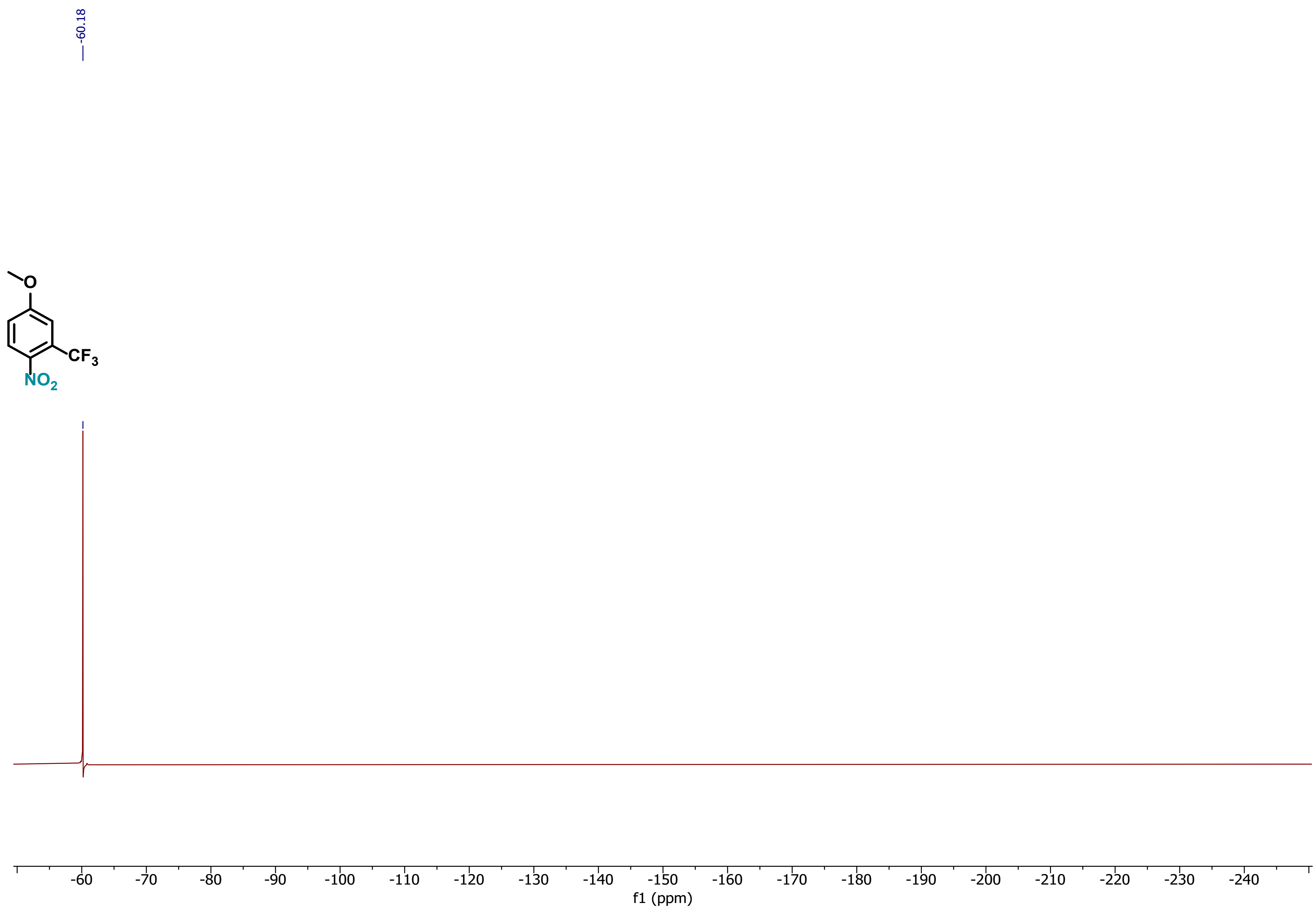
-60.18
O
CF3
NO2
-60
-70
-80
-90
-100
-110
-120
-130
-140
-150
-160
-170
-180
-190
-200
-210
-220
-230
-240
f1 (ppm)

2-methoxy-1-nitro-4-(trifluoromethyl)benzene (38b)

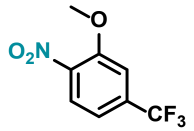

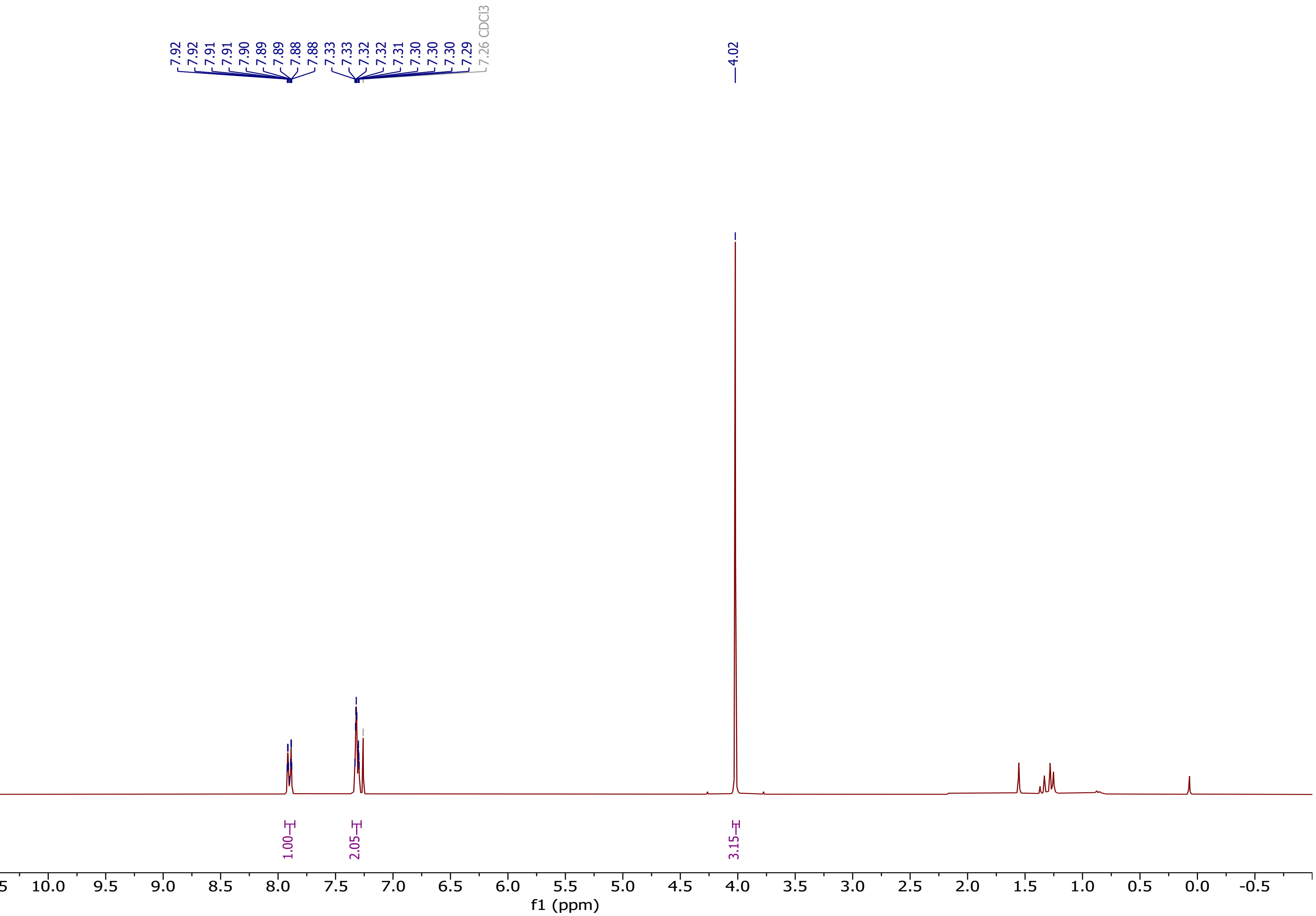

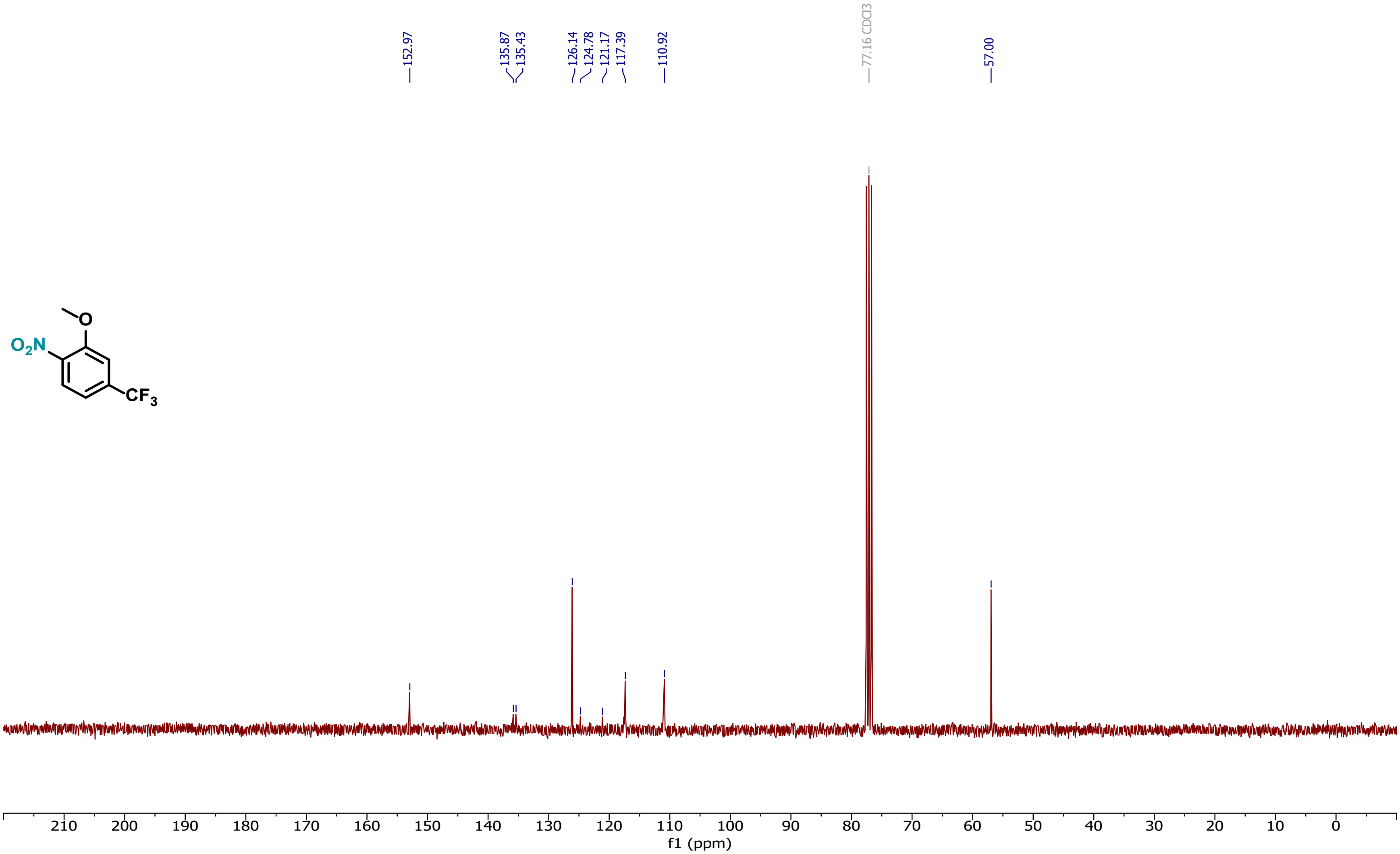
152.97
135.87
135.43
126.14
124.78
121.17
117.39
110.92
77.16 CDCl3
57.00
210
200
190
180
170
160
150
140
130
120
110
100
90
80
70
60
50
40
30
20
10
0
f1 (ppm)

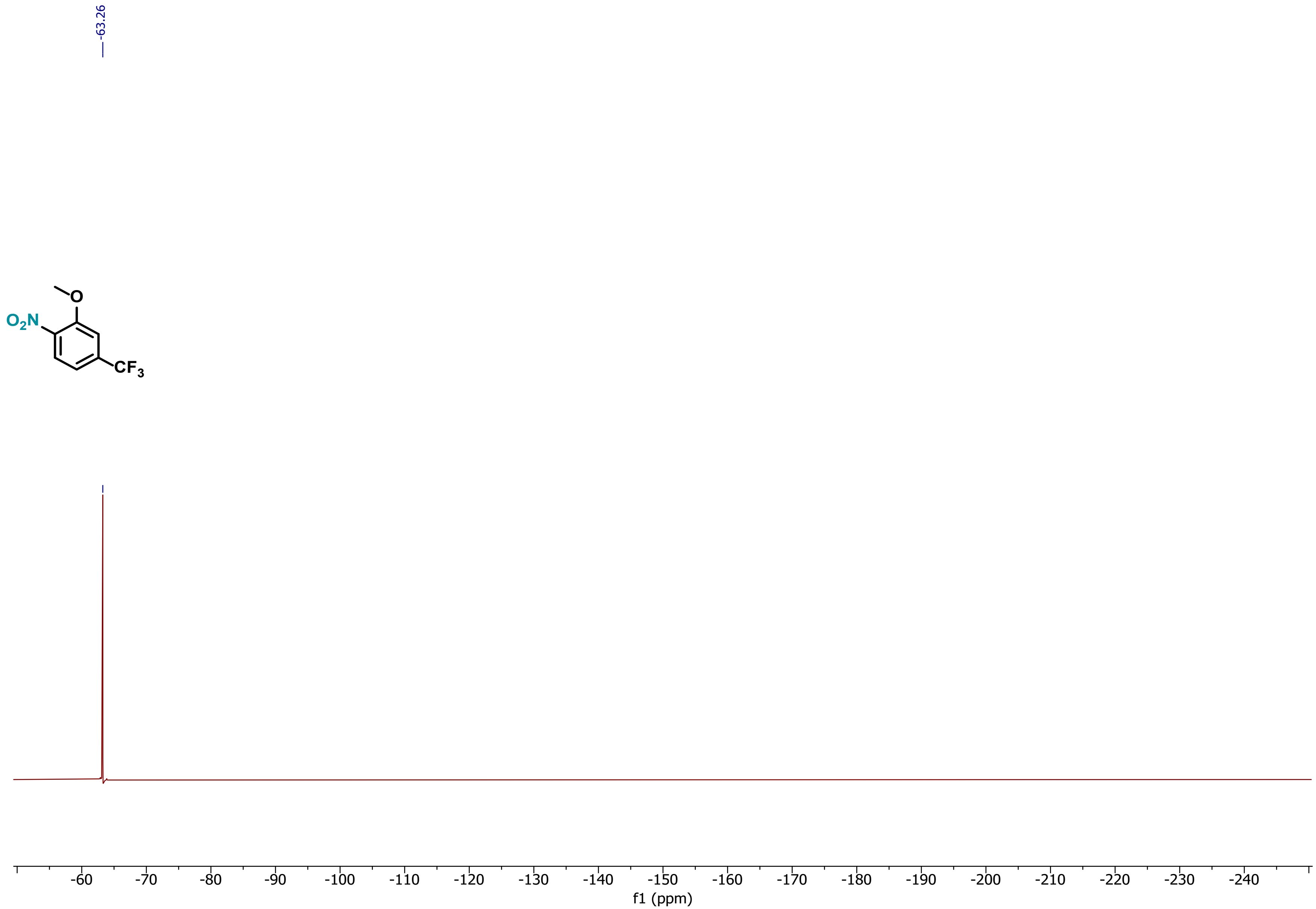

-63.26
O2N
O
CF3
-60
-70
-80
-90
-100
-110
-120
-130
-140
-150
-160
-170
-180
-190
-200
-210
-220
-230
-240
f1 (ppm)

methyl 5-chloro-2-methoxy-3-nitrobenzoate (39)

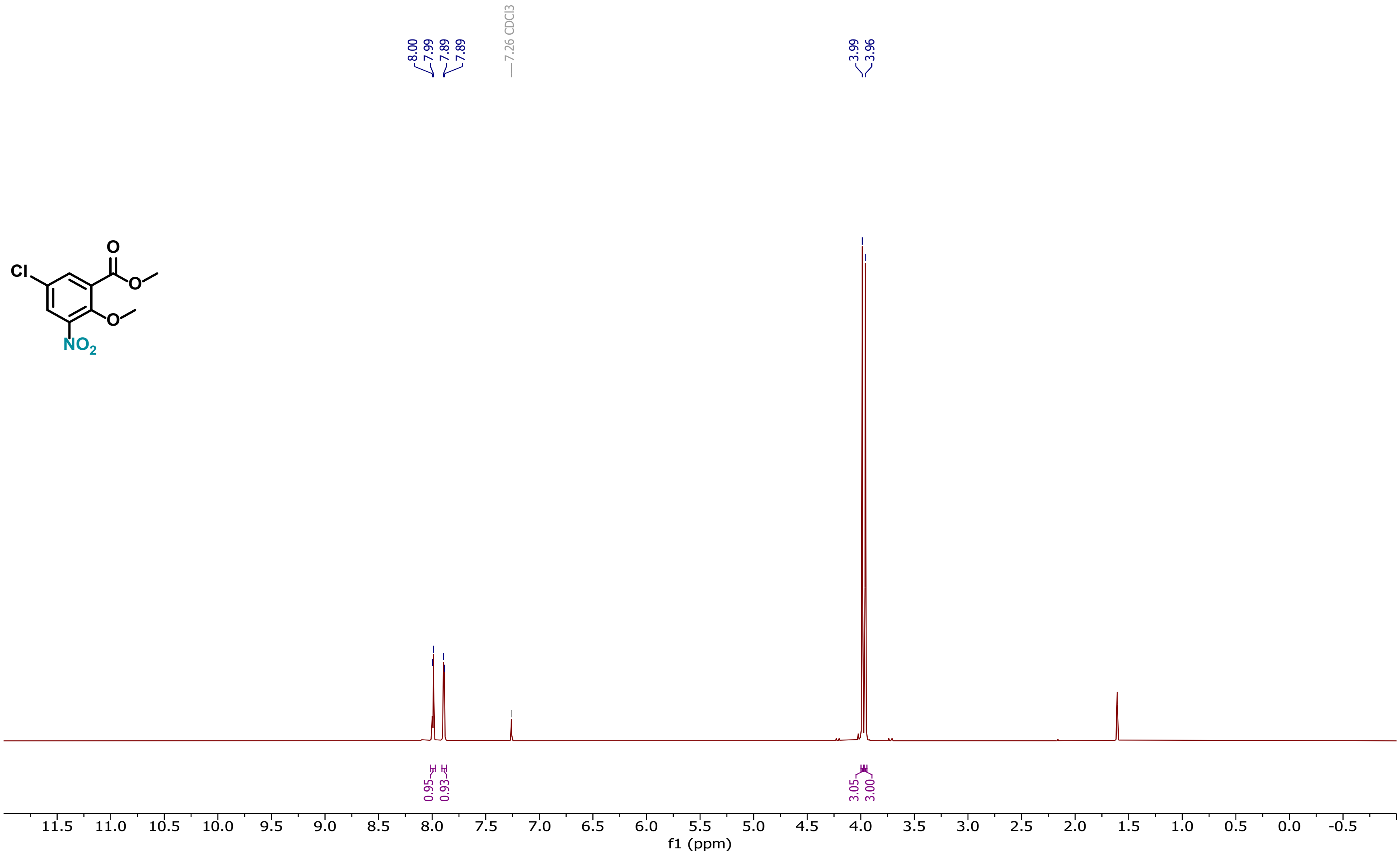

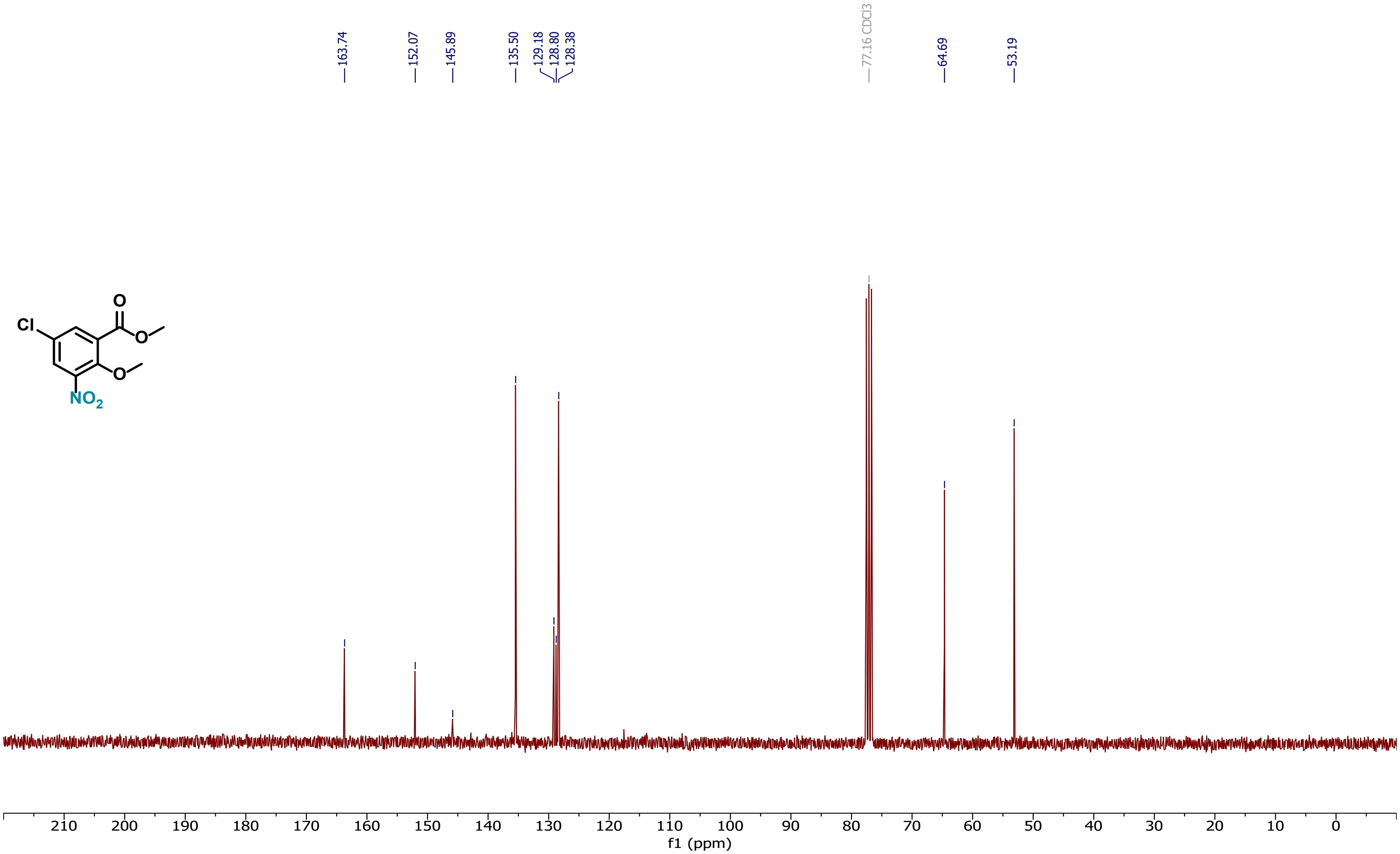
163.74
152.07
145.89
135.50
129.18
128.80
128.38
77.16 CDCl3
64.69
53.19
Cl
O
O
O
NO2
210
200
190
180
170
160
150
140
130
120
110
100
90
80
70
60
50
40
30
20
10
0
f1 (ppm)

2-methoxy-5-nitrobenzaldehyde (40)

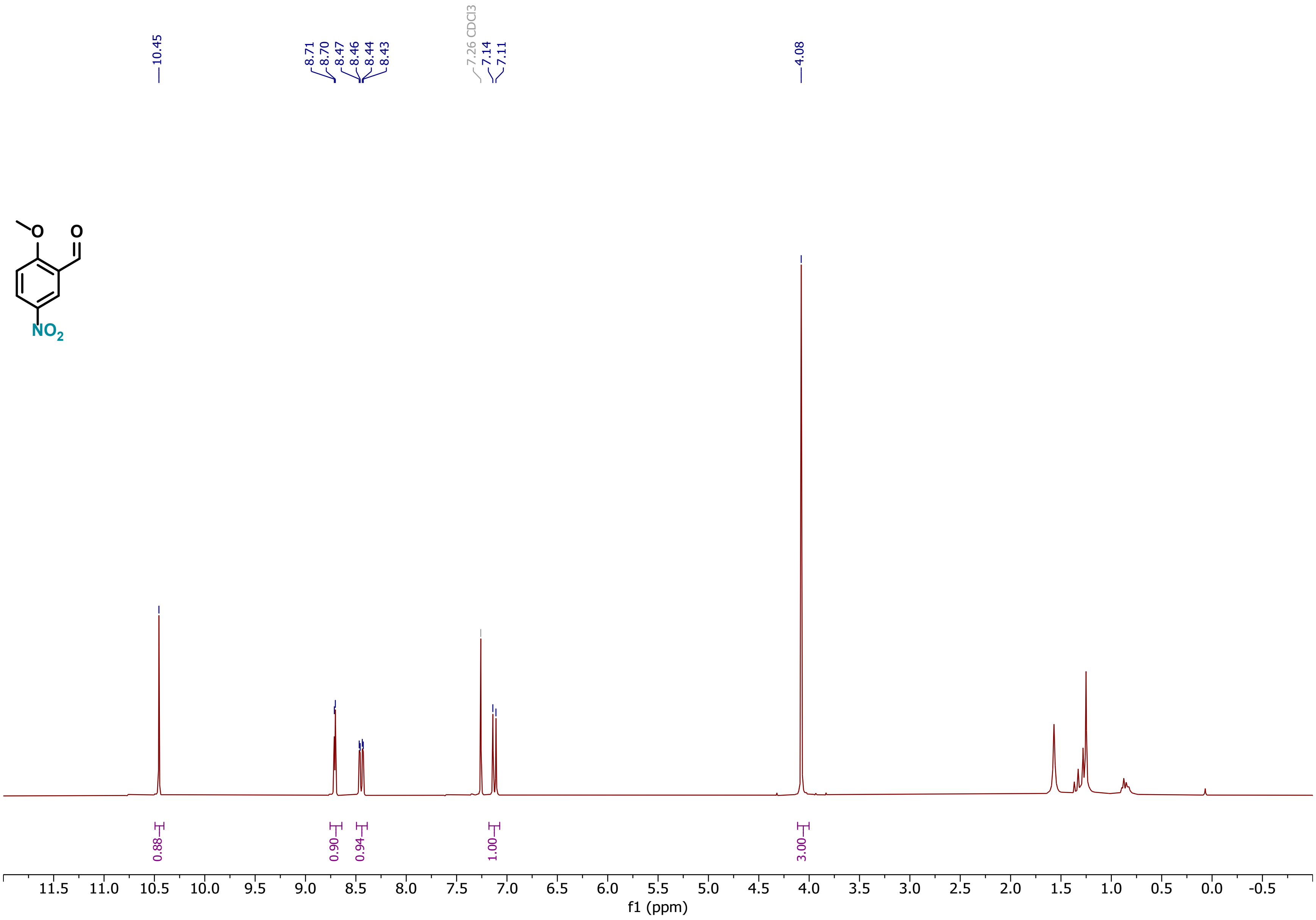

4-nitrophenyl acetate and 2-nitrophenyl acetate (41)

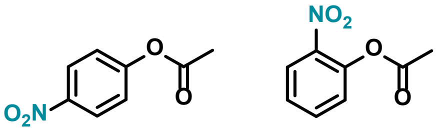

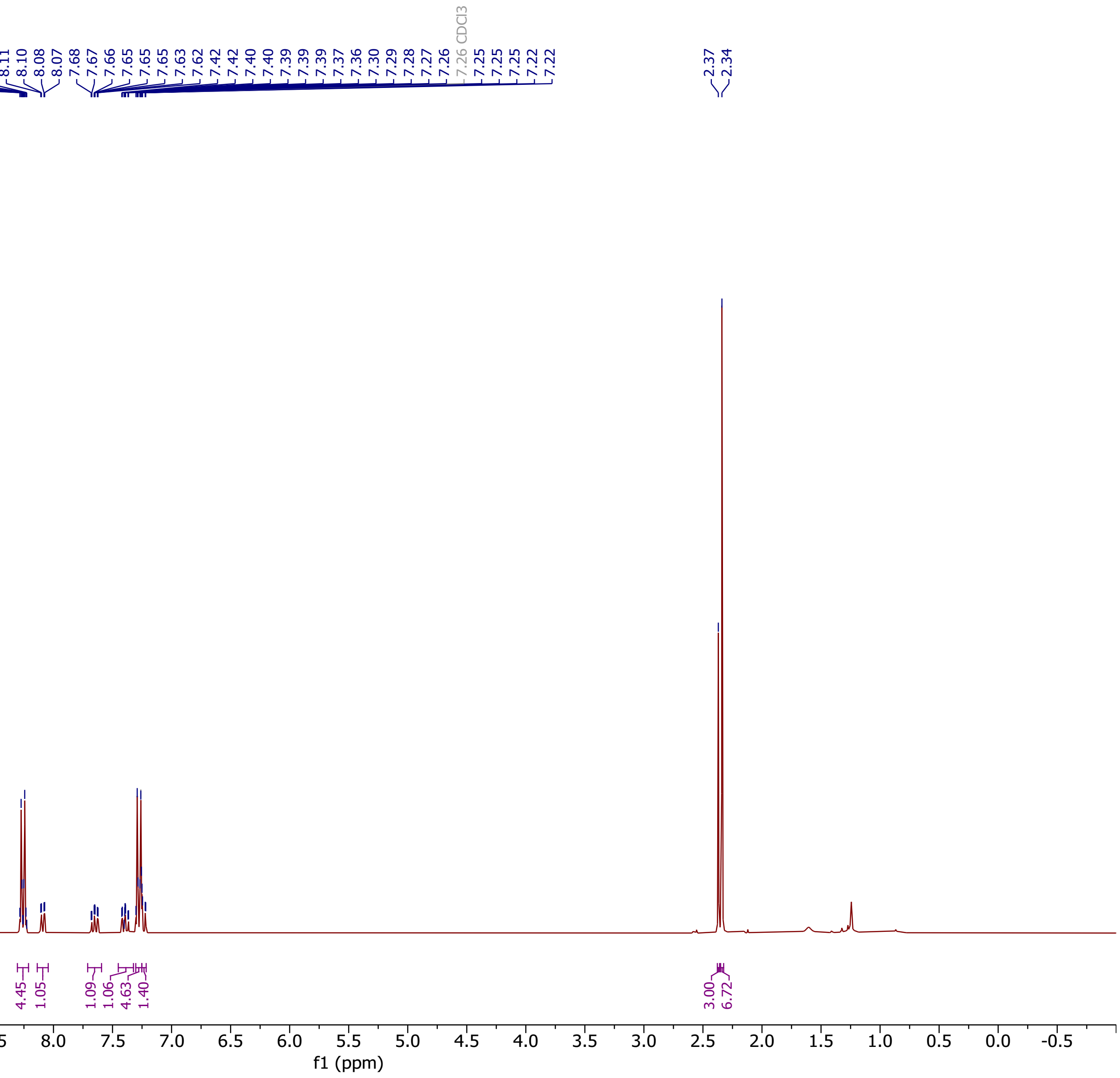

1-nitronaphthalene (42)

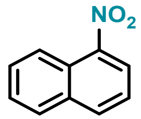

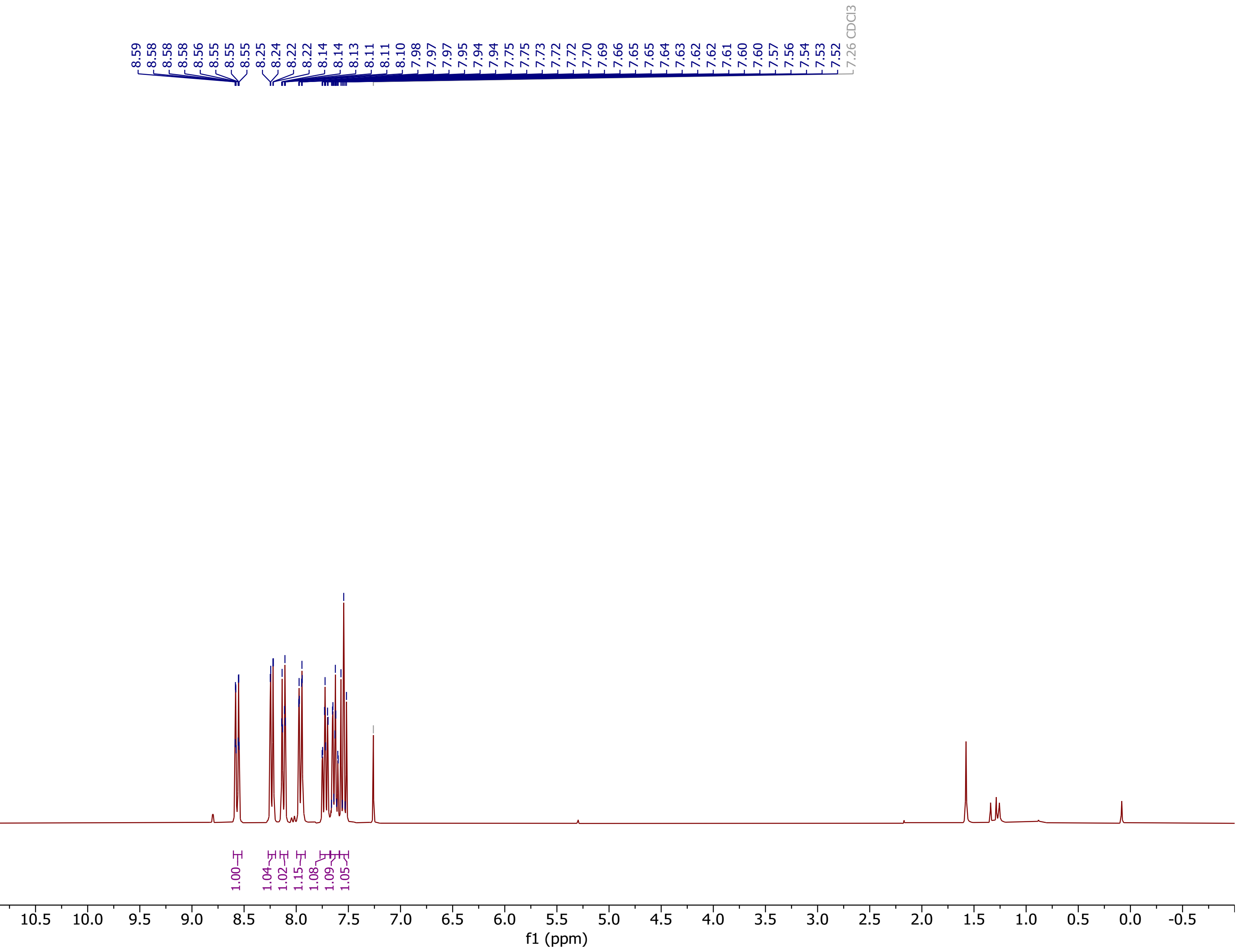

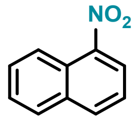
NO2

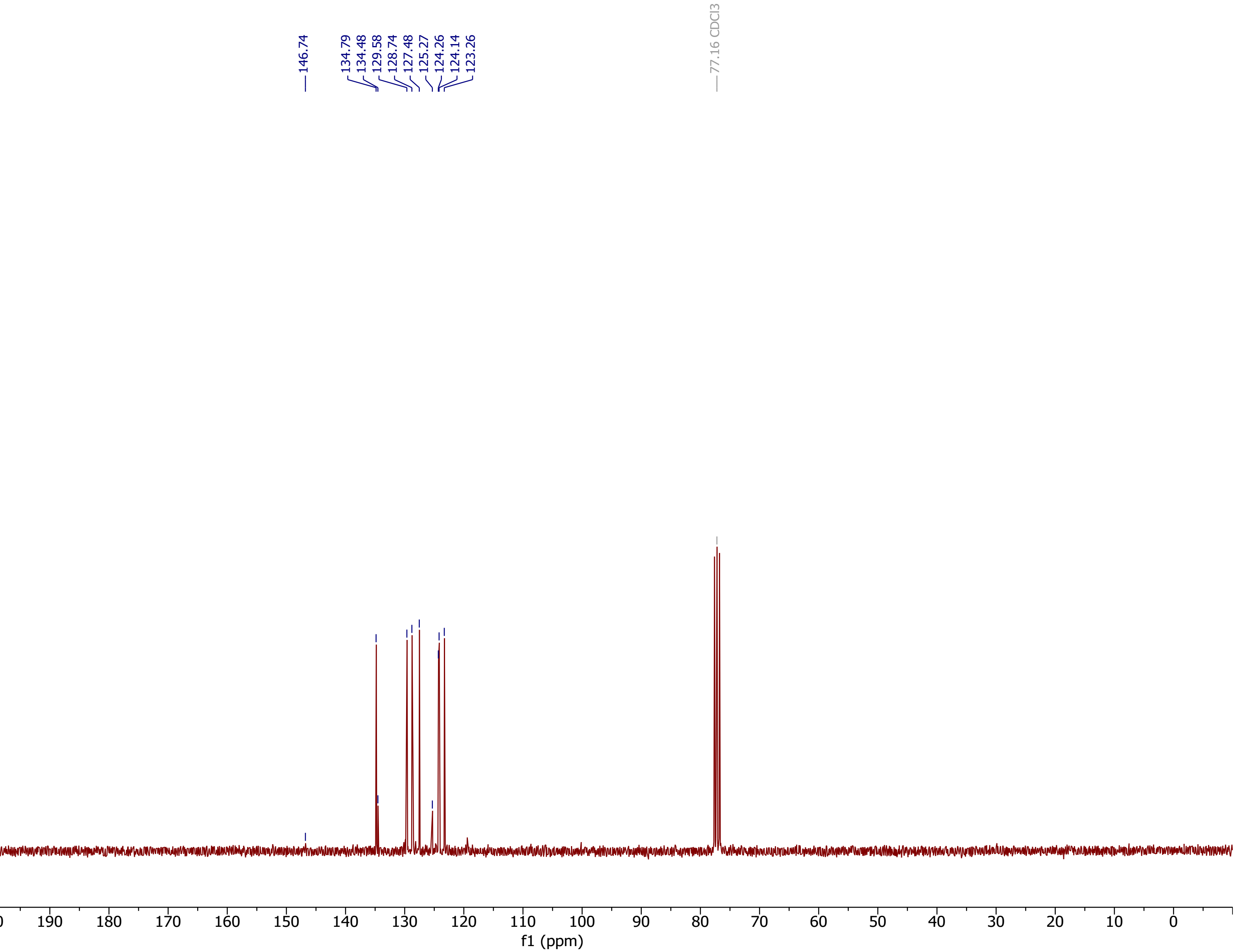
146.74
134.79
134.48
129.58
128.74
127.48
125.27
124.26
124.14
123.26
77.16 CDCl3
210
200
190
180
170
160
150
140
130
120
110
100
90
80
70
60
50
40
30
20
10
0
f1 (ppm)

2-methoxy-1-nitronaphthalene (43a)

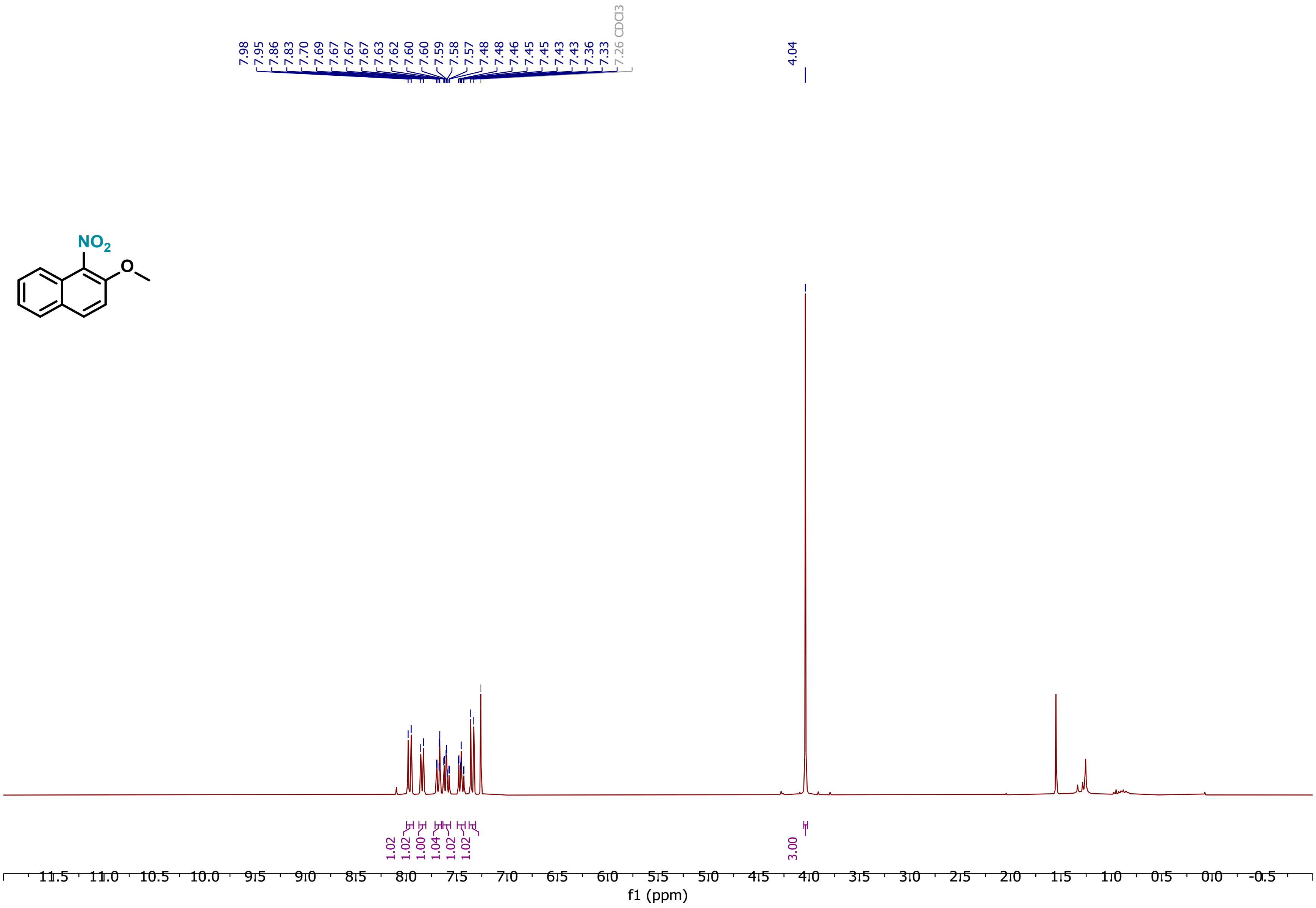

2-methoxy-6-nitronaphthalene (43b)

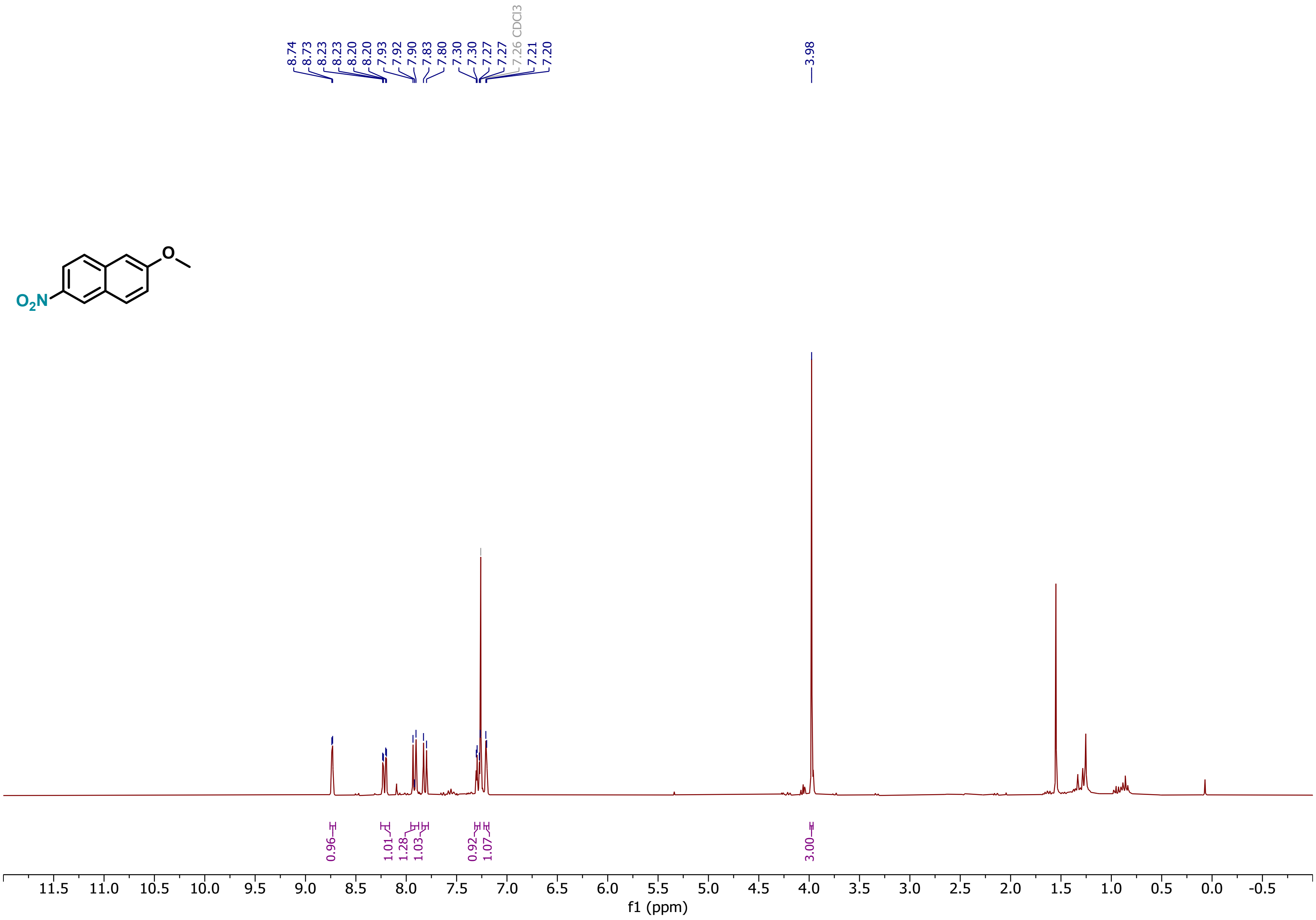

2-methoxy-8-nitronaphthalene (43c)

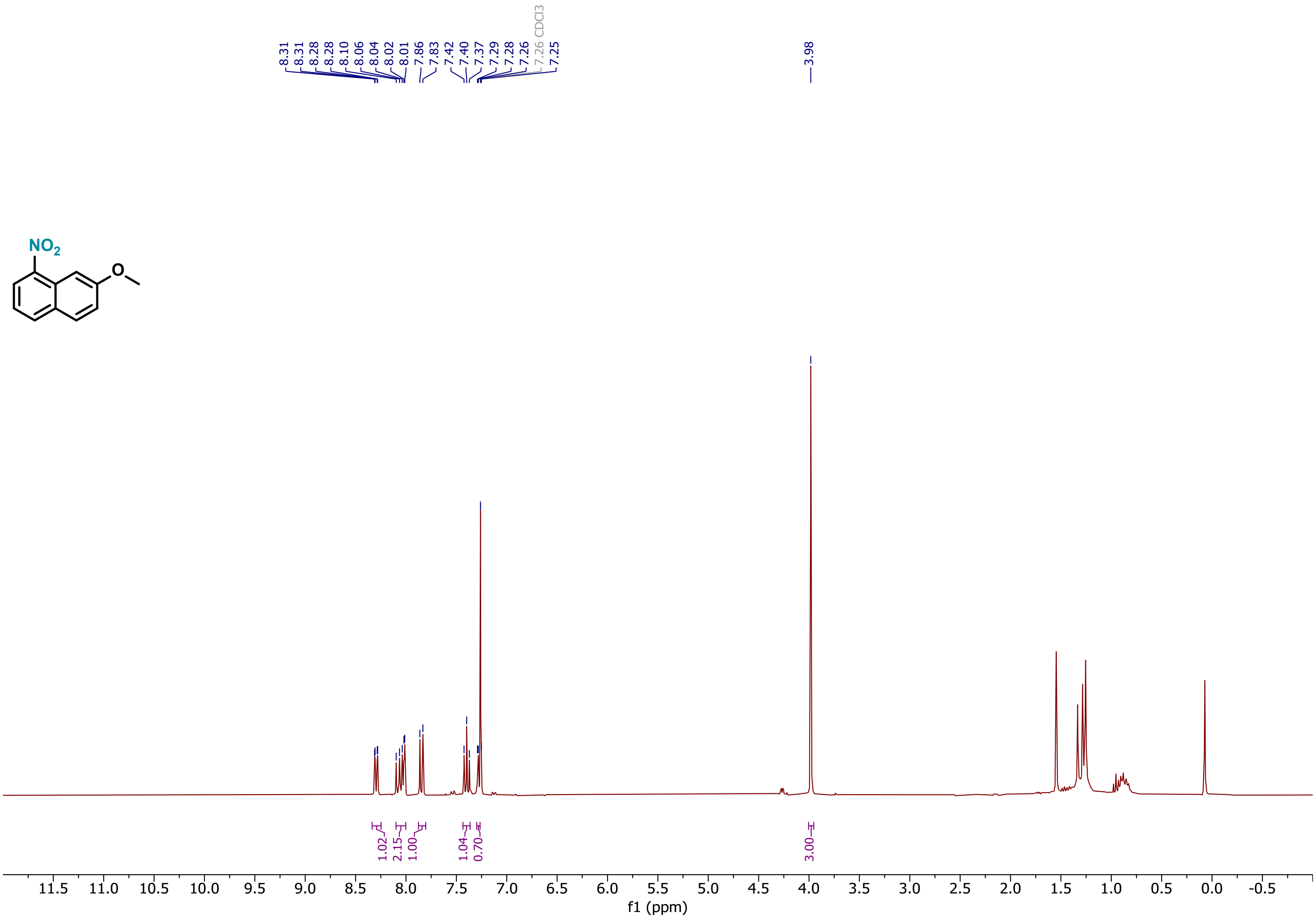

9-bromo-10-nitroanthracene (44)

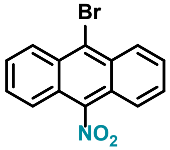

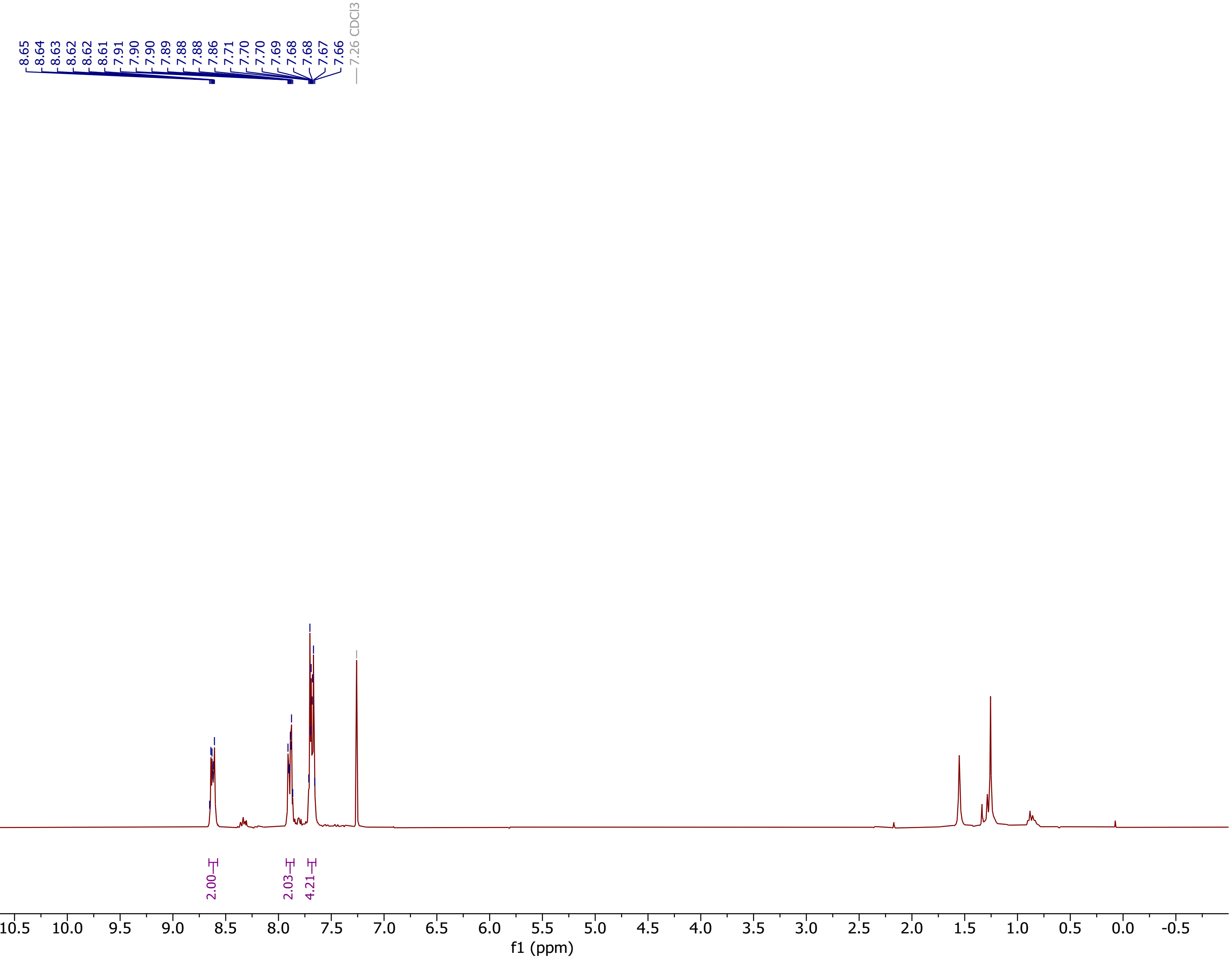

2-nitrodibenzo[b,d]thiophene (45)

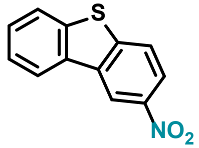

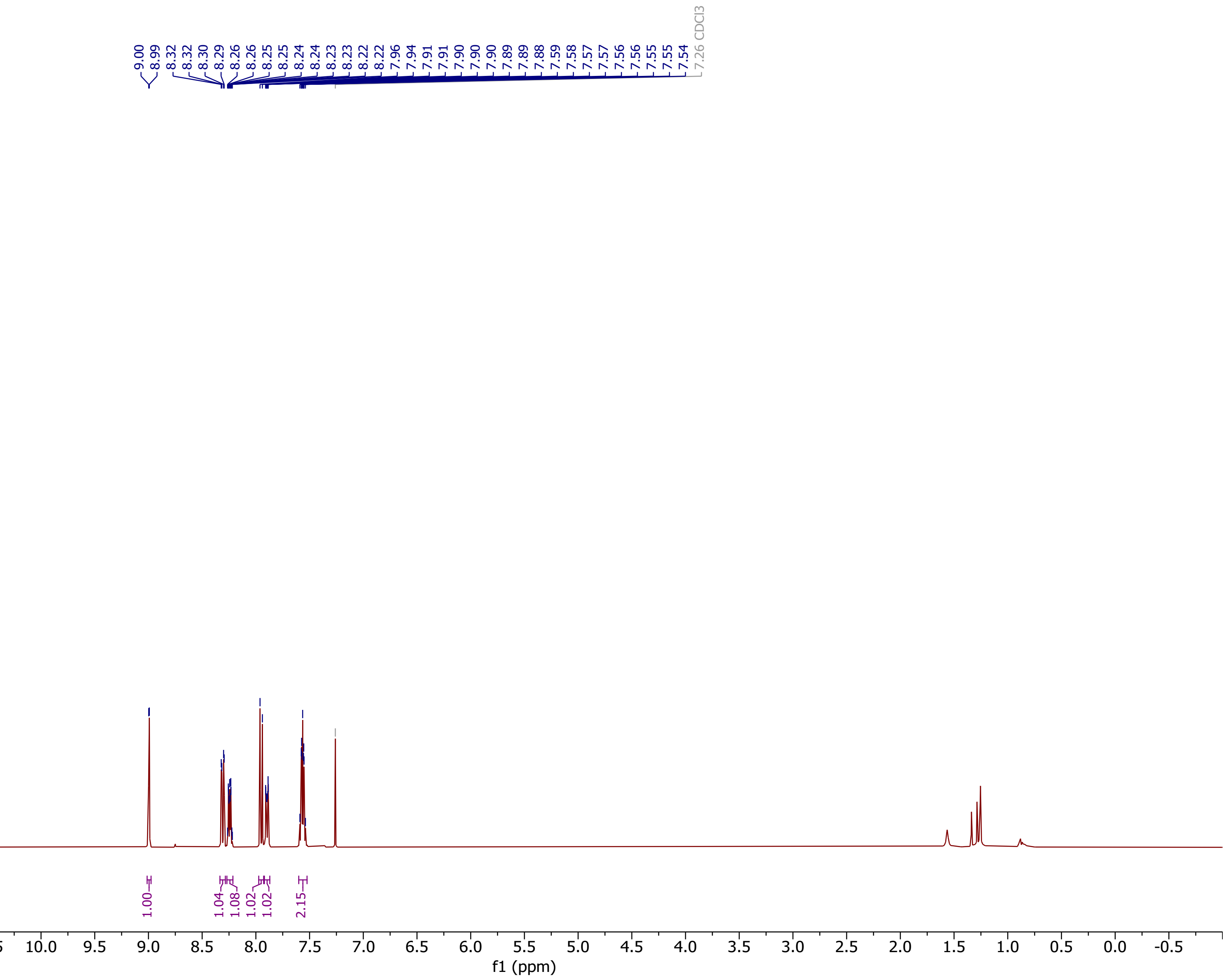

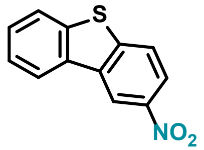
S
NO2

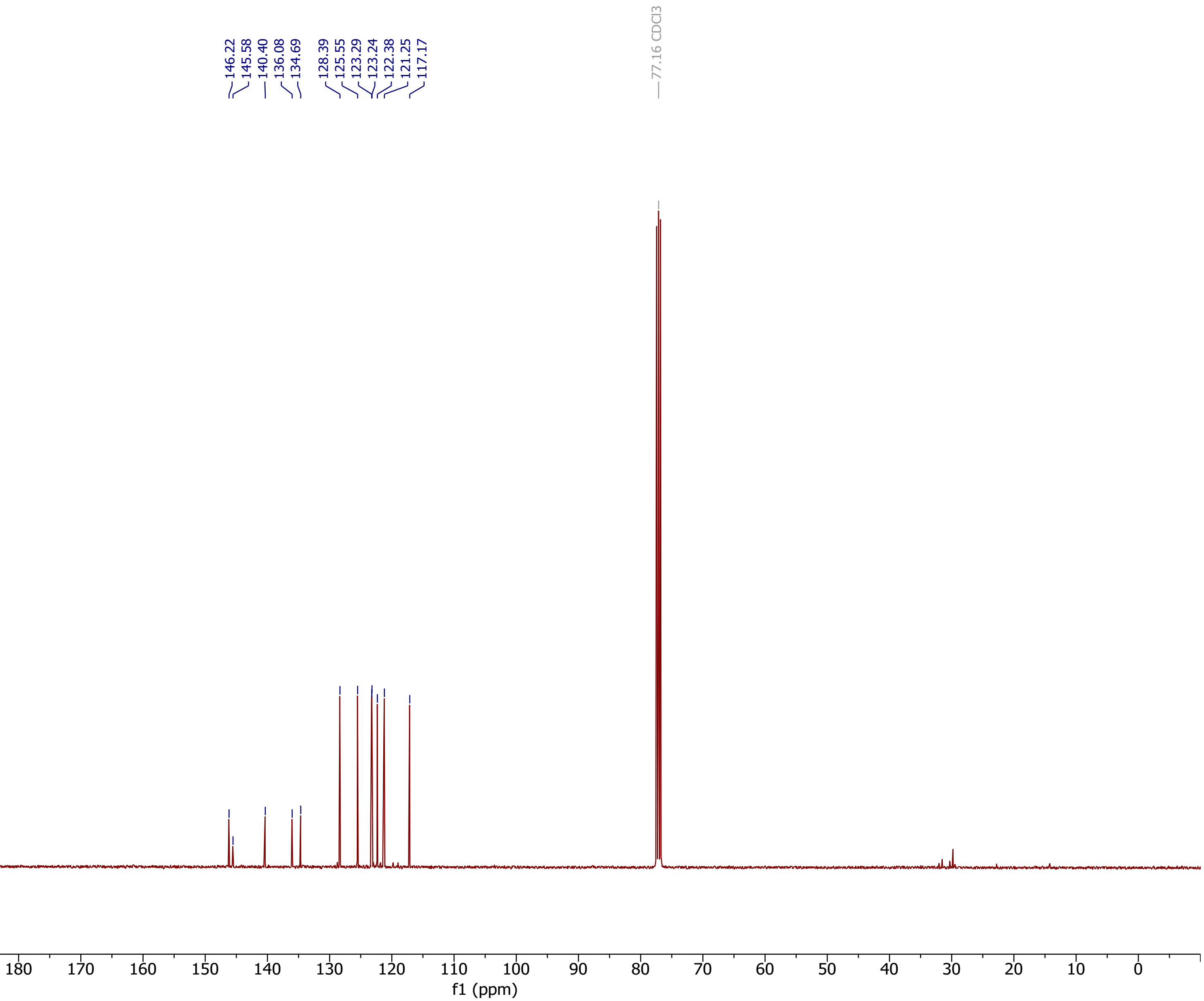
146.22
145.58
140.40
136.08
134.69
128.39
125.55
123.29
123.24
122.38
121.25
117.17
77.16 CDCl3
210
200
190
180
170
160
150
140
130
120
110
100
90
80
70
60
50
40
30
20
10
0
f1 (ppm)

1-(4-nitrophenyl)-2-phenyldiazene (46)

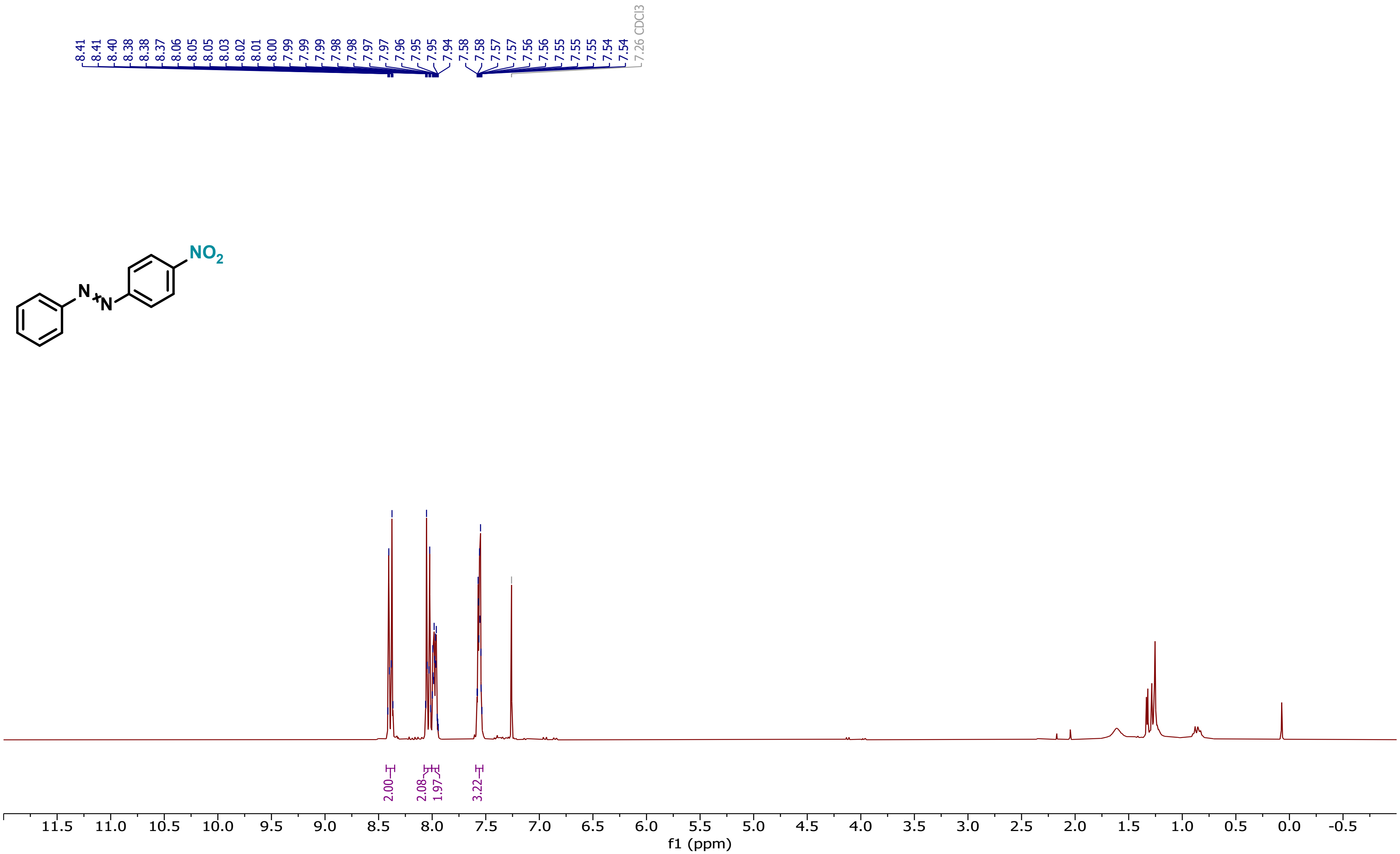

9-(4-bromophenyl)-3-nitro-9H-carbazole (47)

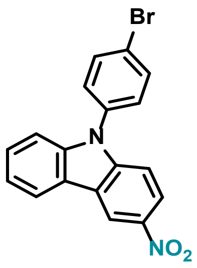

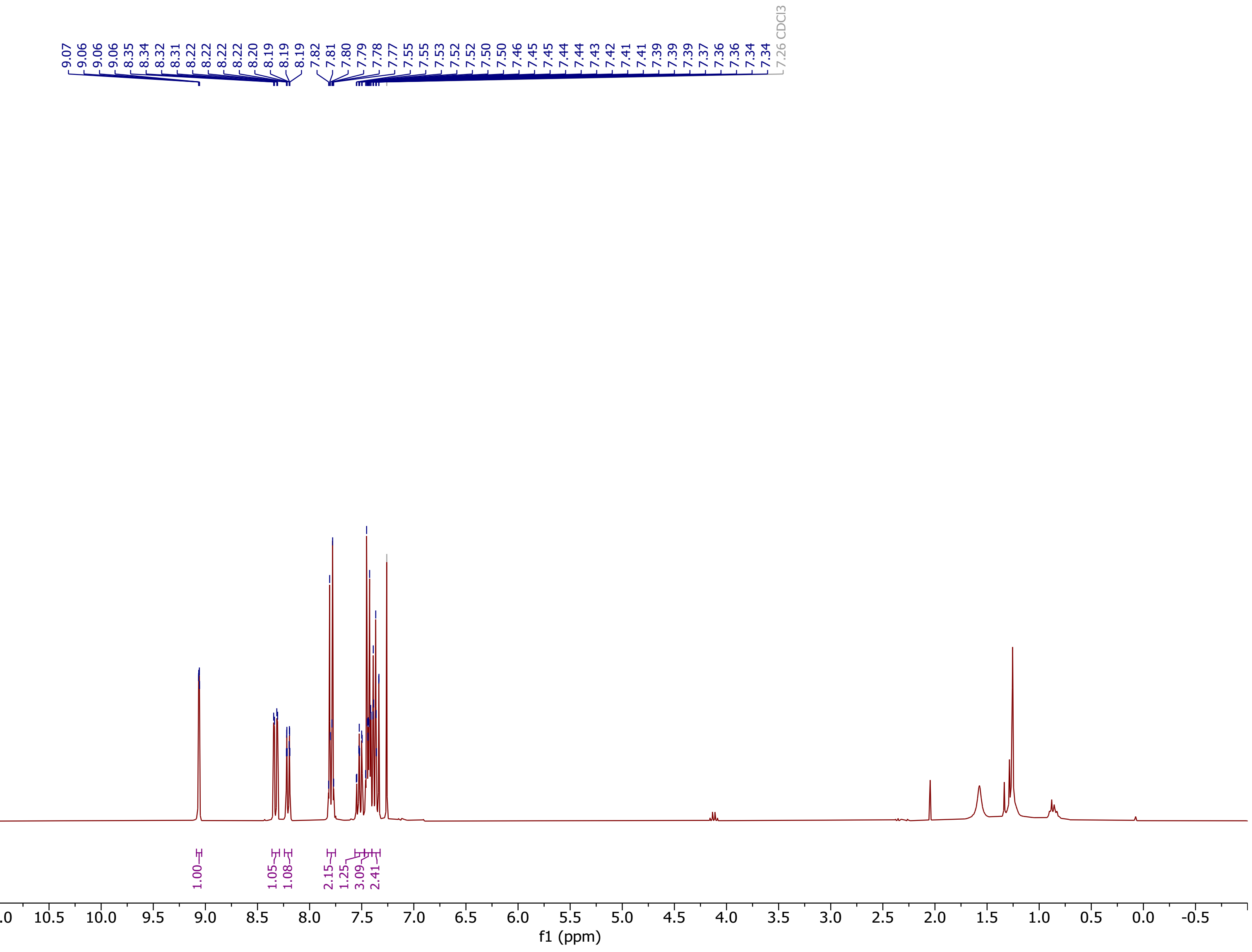

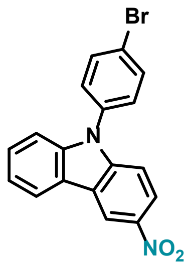
Br
N
NO2

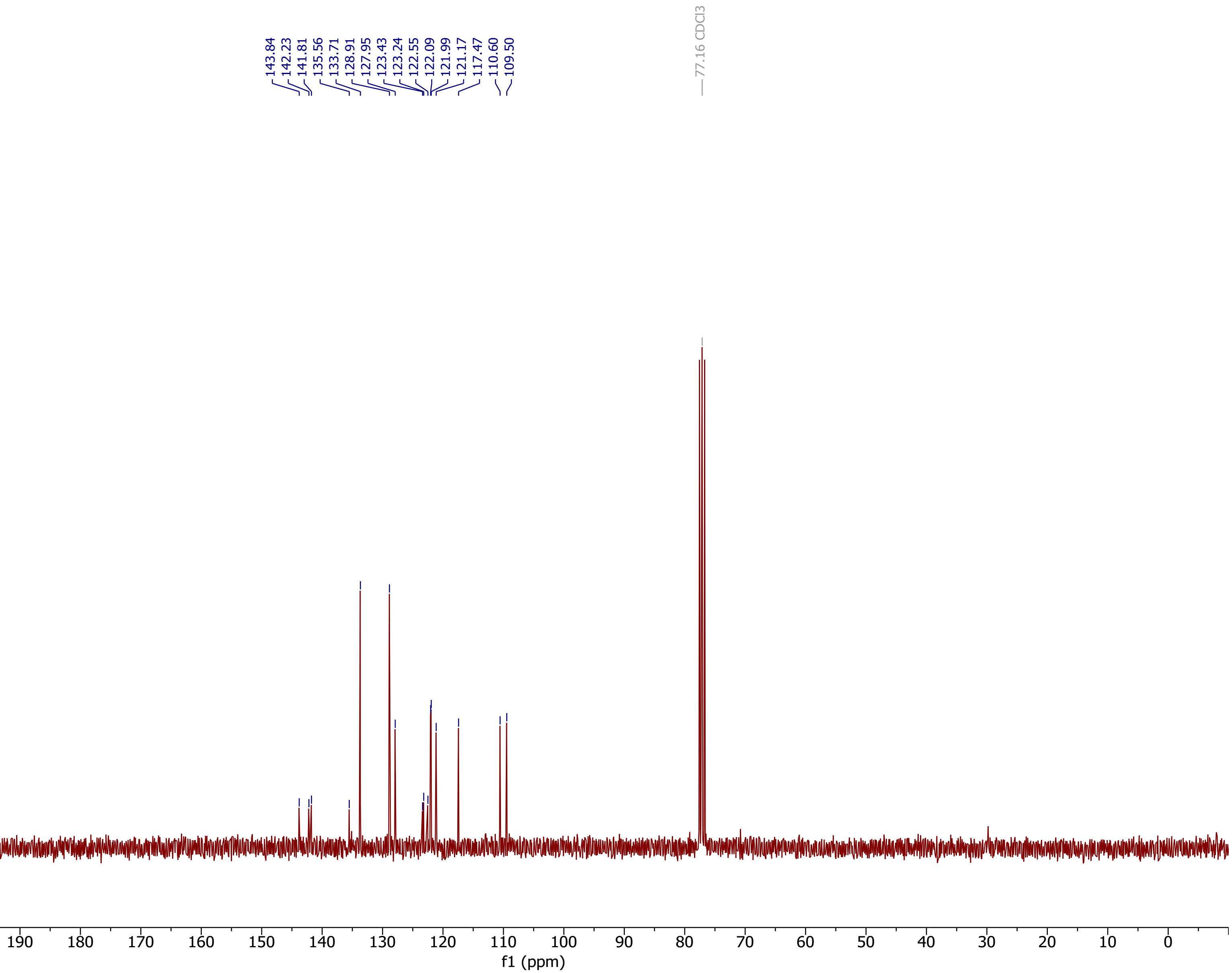
143.84
142.23
141.81
135.56
133.71
128.91
127.95
123.43
123.24
122.55
122.09
121.99
121.17
117.47
110.60
109.50
77.16 CDCl3
210
200
190
180
170
160
150
140
130
120
110
100
90
80
70
60
50
40
30
20
10
0
f1 (ppm)

(8R,9S,13S,14S)-3-methoxy-13-methyl-2-nitro-6,7,8,9,11,12,13,14,15,16-decahydro-17H-cyclopenta[a]phenanthren-17-one (48a)

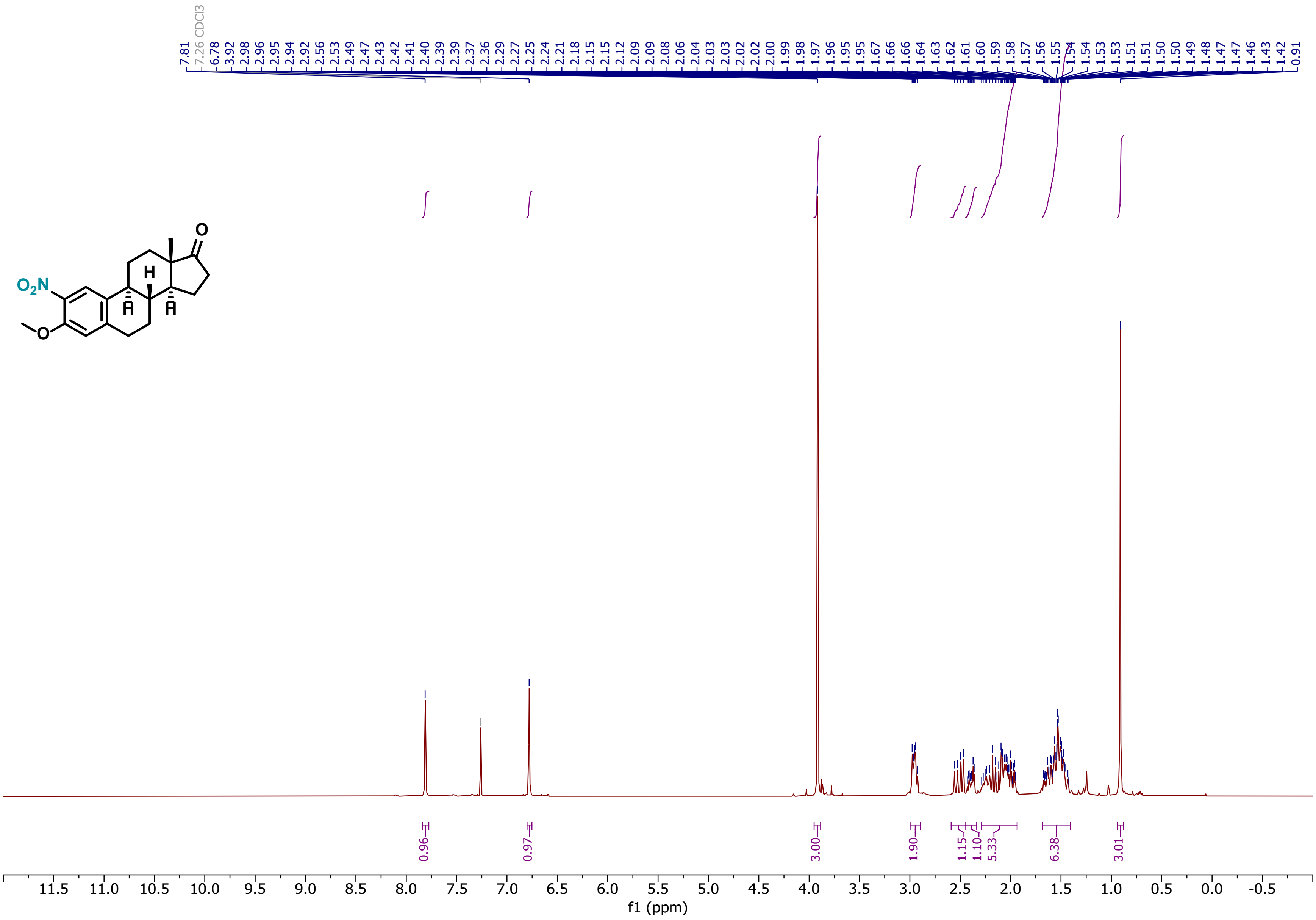

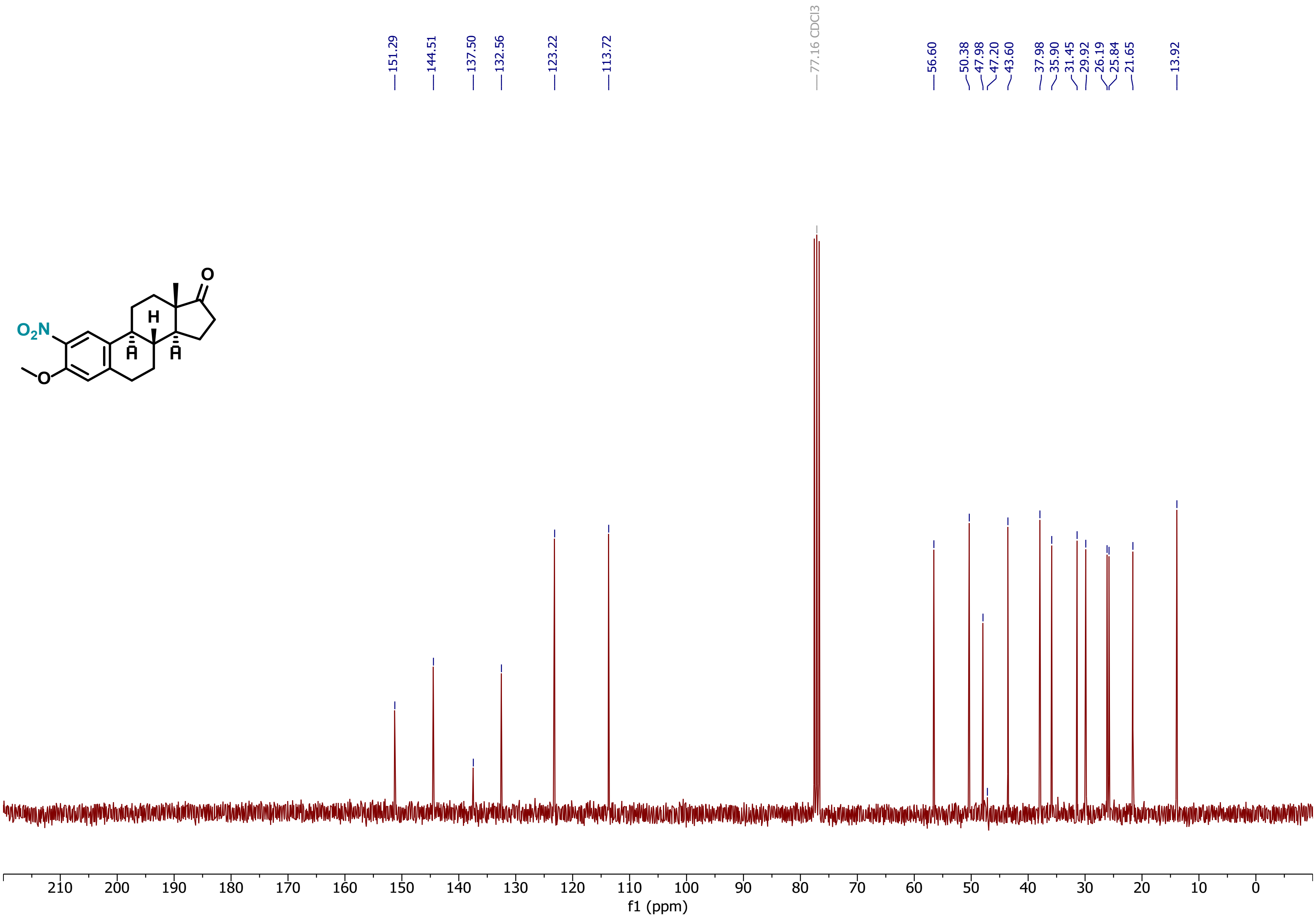

151.29
144.51
137.50
132.56
123.22
113.72
77.16 CDCl3
56.60
50.38
47.98
47.20
43.60
37.98
35.90
31.45
29.92
26.19
25.84
21.65
13.92
O2N
O
H
210 200 190 180 170 160 150 140 130 120 110 100 90 80 70 60 50 40 30 20 10 0
f1 (ppm)

(8R,9S,13S,14S)-3-methoxy-13-methyl-4-nitro-6,7,8,9,11,12,13,14,15,16-decahydro-17H-cyclopenta[a]phenanthren-17-one (48b)

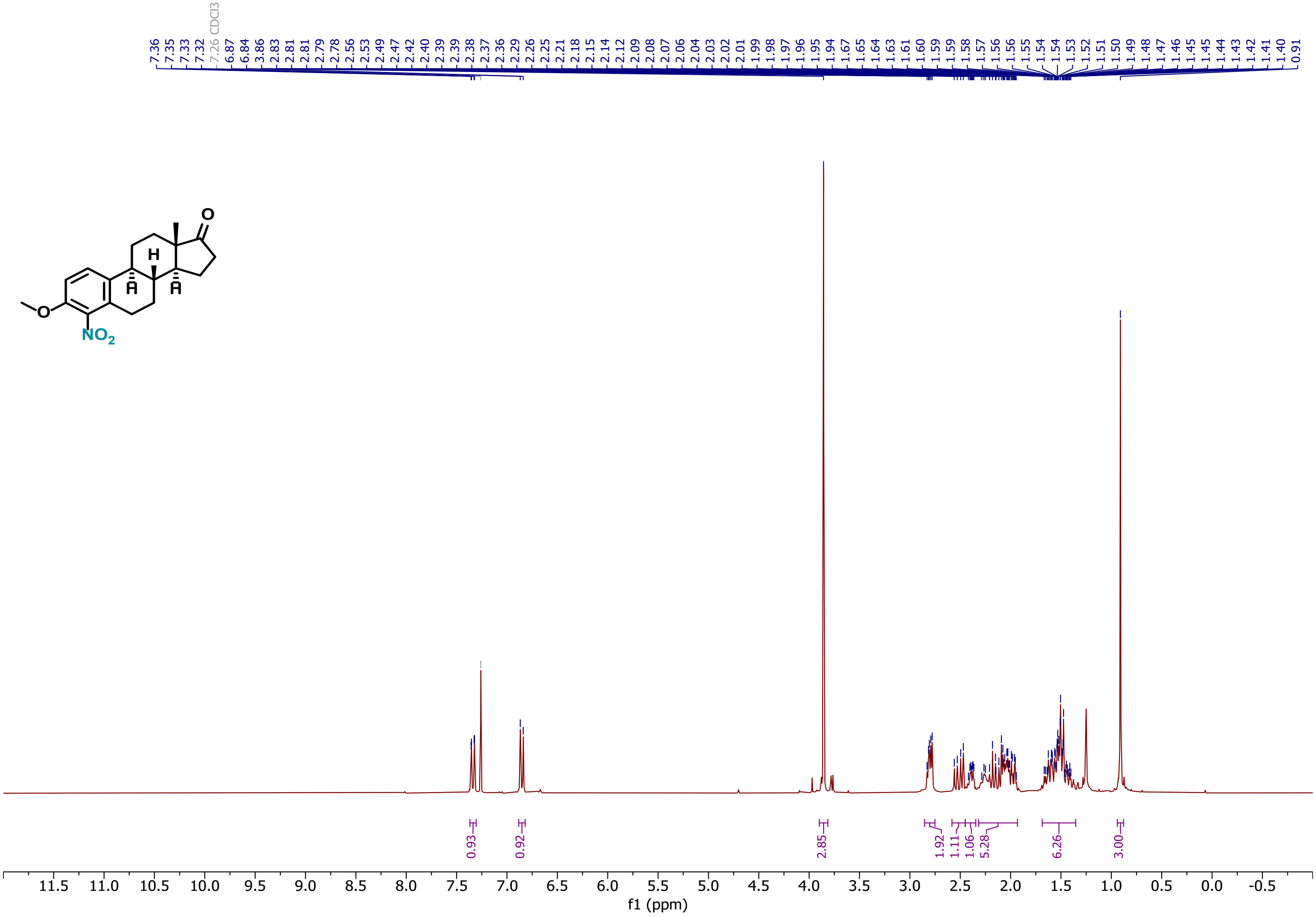

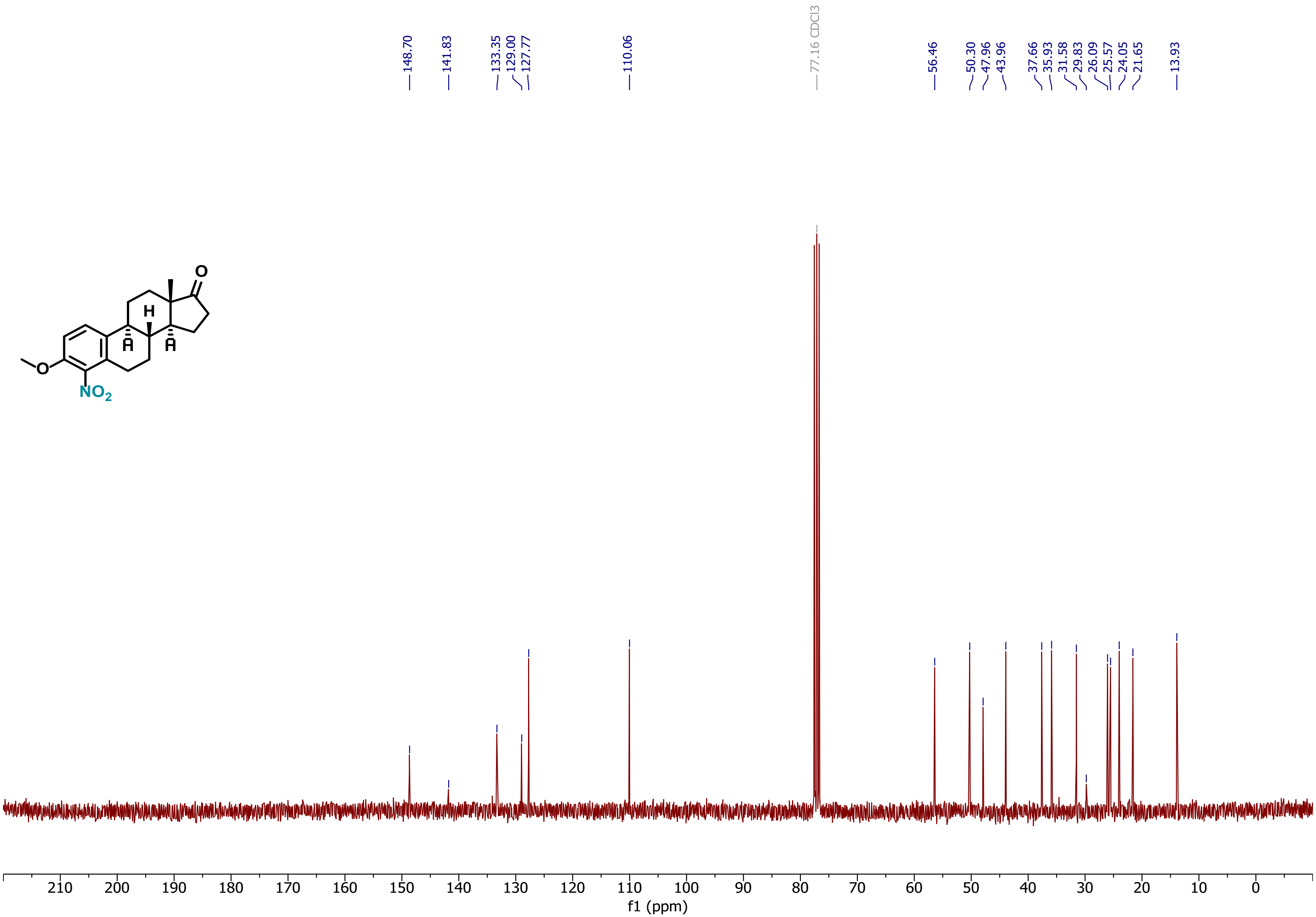

148.70
141.83
133.35
129.00
127.77
110.06
77.16 CDCl3
56.46
50.30
47.96
43.96
37.66
35.93
31.58
29.83
26.09
25.57
24.05
21.65
13.93
O
H
H
H
O
NO_2
210 200 190 180 170 160 150 140 130 120 110 100 90 80 70 60 50 40 30 20 10 0
f1 (ppm)

isopropyl 2-(4-(4-chlorobenzoyl)-2-nitrophenoxy)-2-methylpropanoate (49)

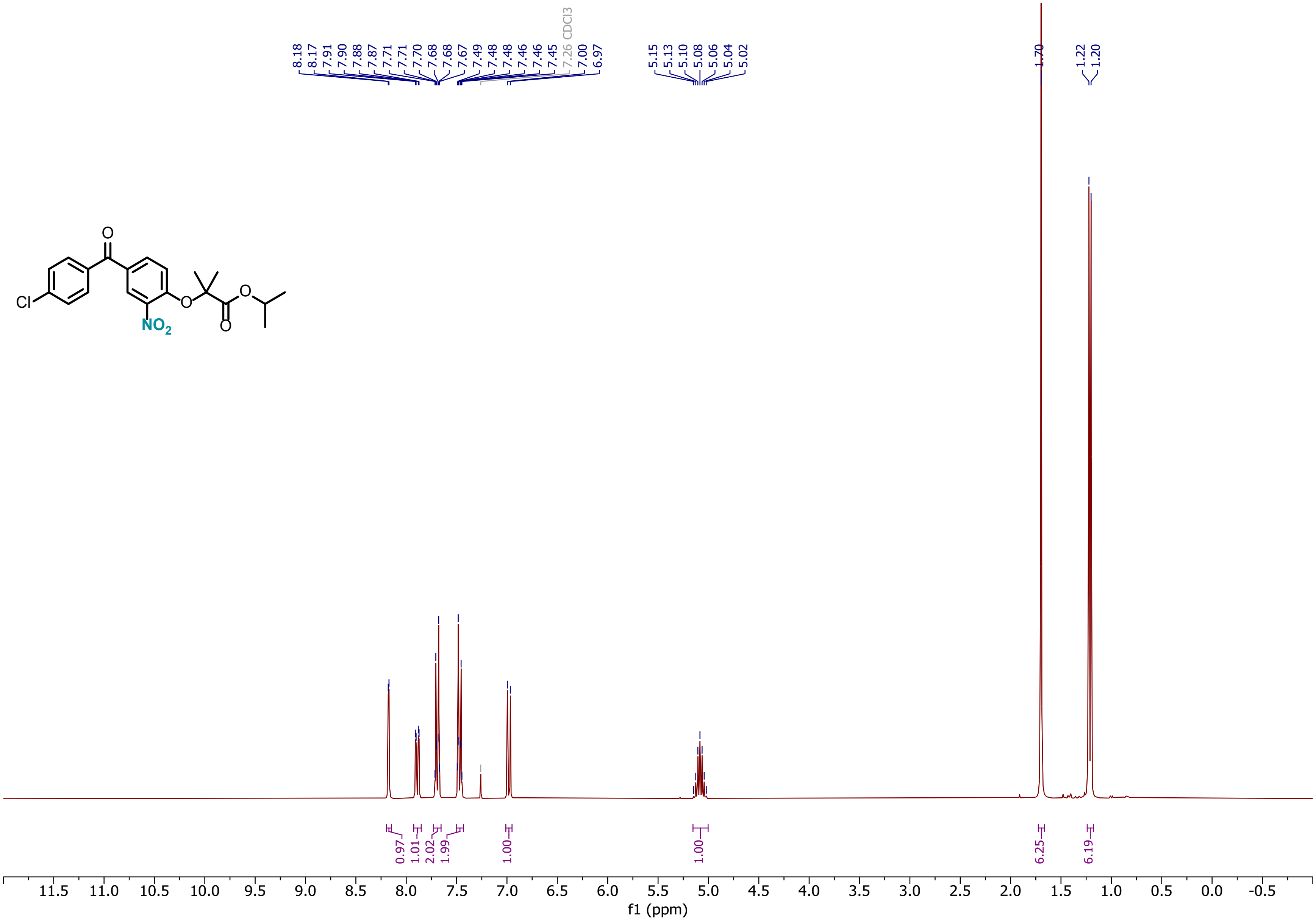

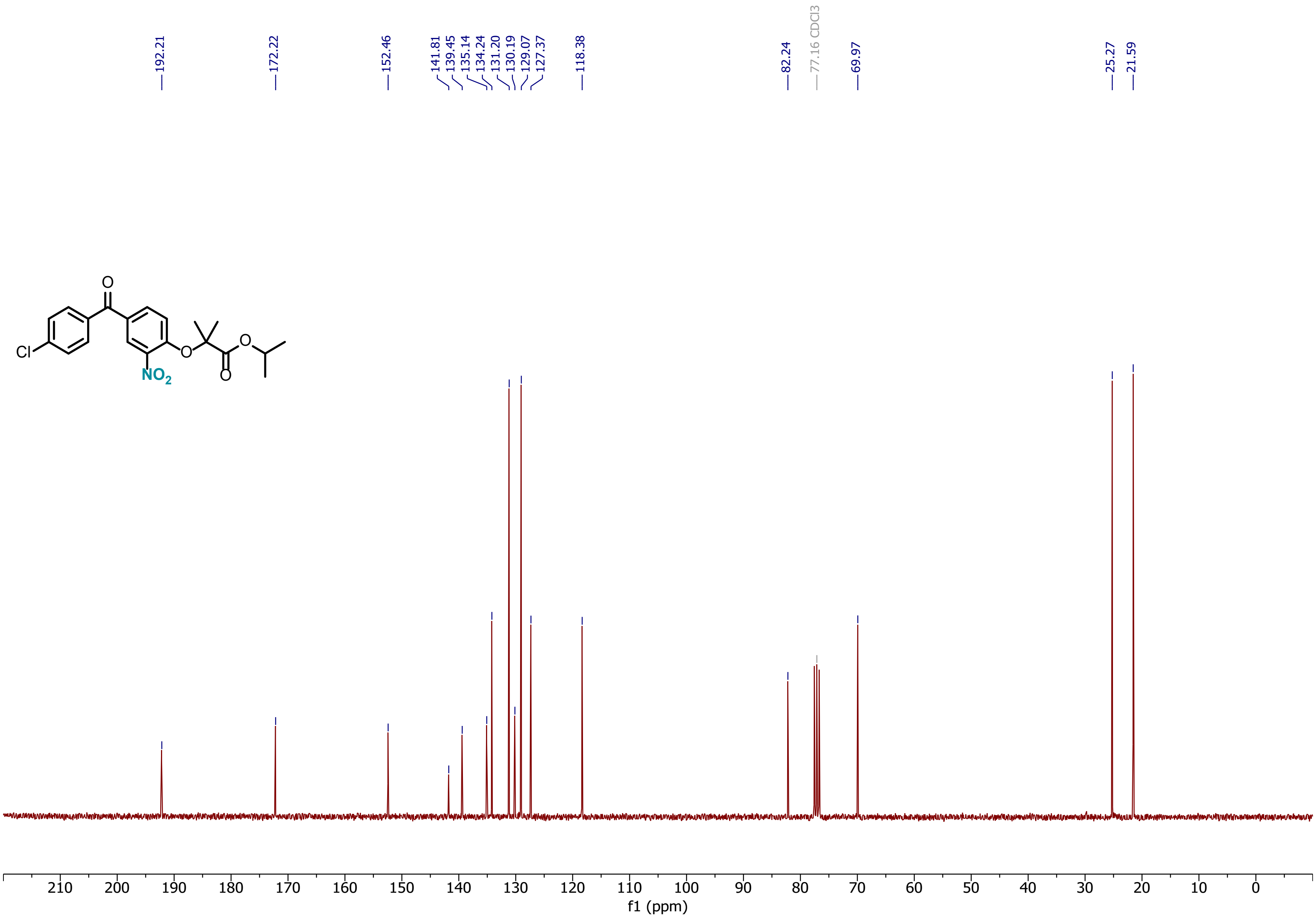
192.21
172.22
152.46
141.81
139.45
135.14
134.24
131.20
130.19
129.07
127.37
118.38
82.24
77.16 CDCl3
69.97
25.27
21.59
Cl
O
O
O
O
NO2
210 200 190 180 170 160 150 140 130 120 110 100 90 80 70 60 50 40 30 20 10 0
f1 (ppm)

1-nitro-4-(2-thiocyanatoethyl)benzene (51)

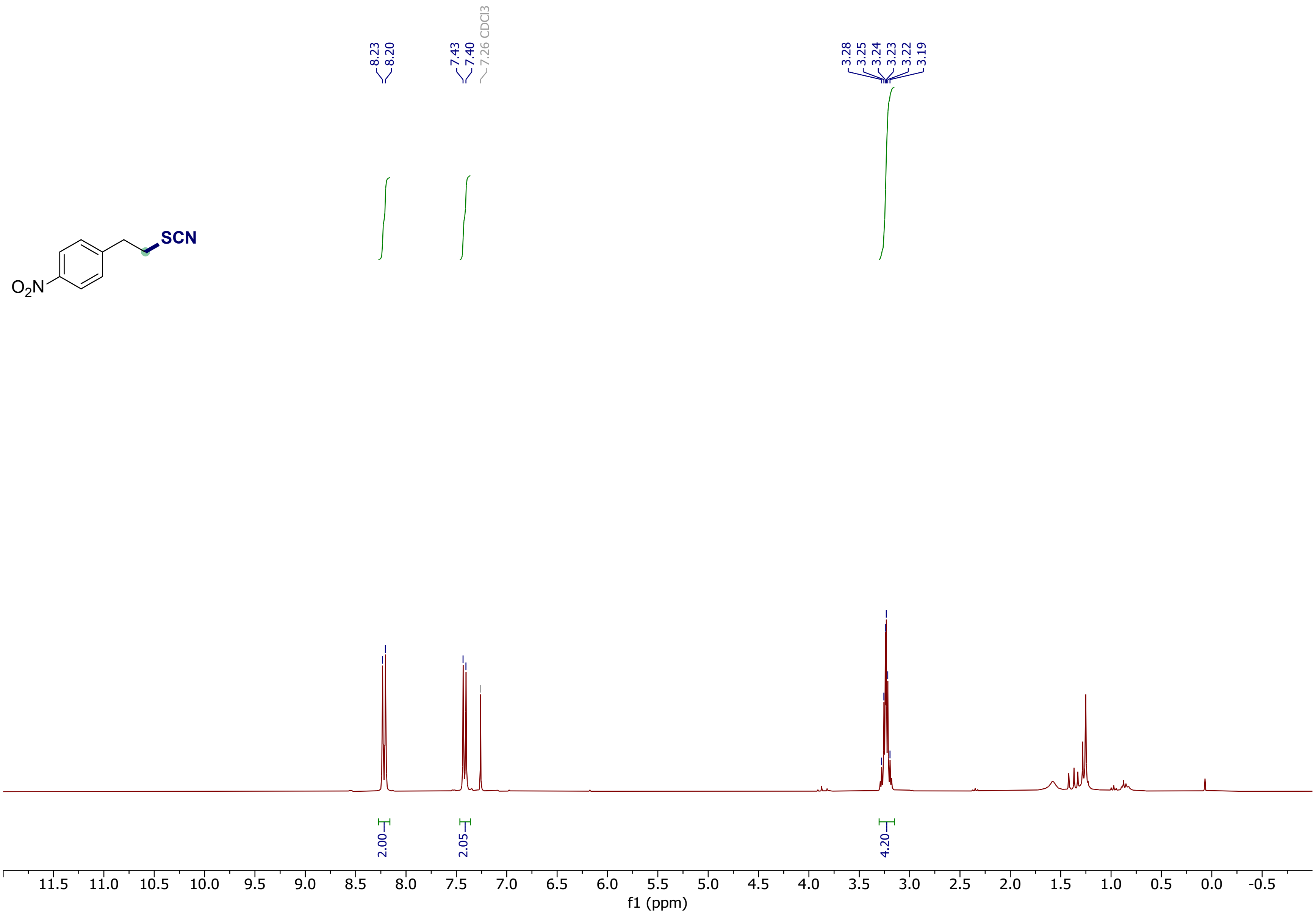

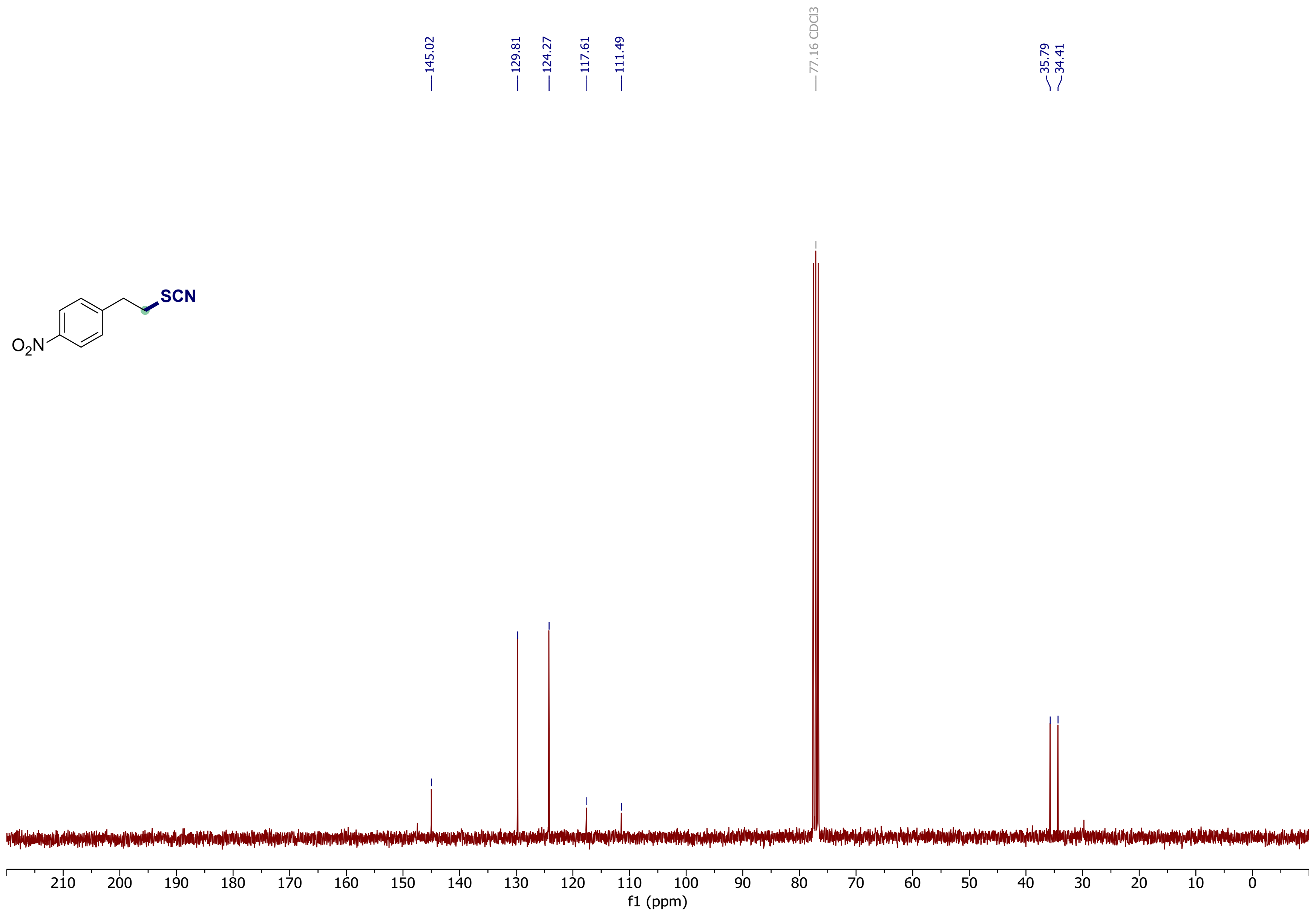

SCN
O2N
145.02
129.81
124.27
117.61
111.49
77.16 CDCl3
35.79
34.41
210
200
190
180
170
160
150
140
130
120
110
100
90
80
70
60
50
40
30
20
10
0
f1 (ppm)

2-(bromomethyl)isoindoline-1,3-dione (52)

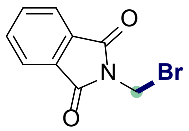

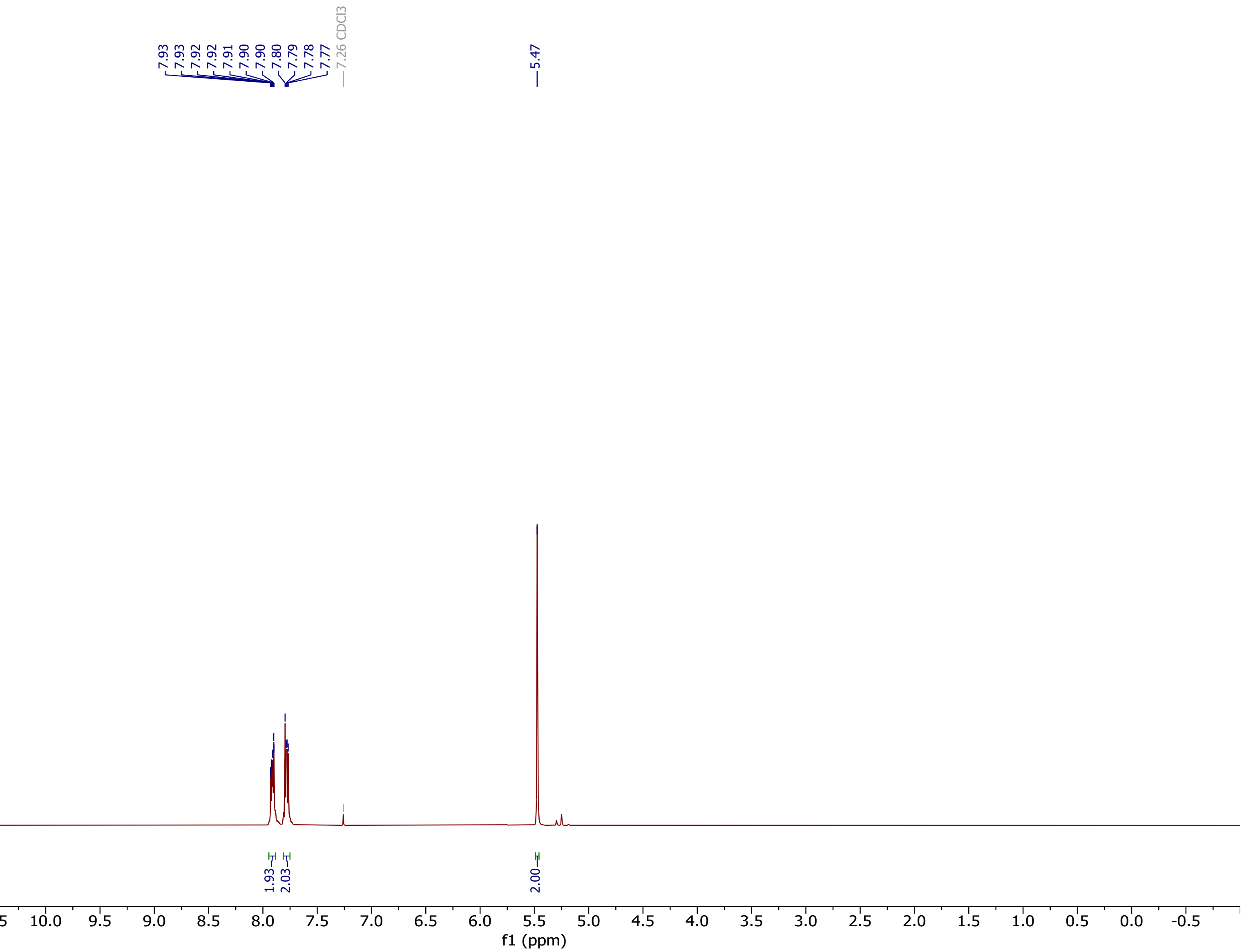

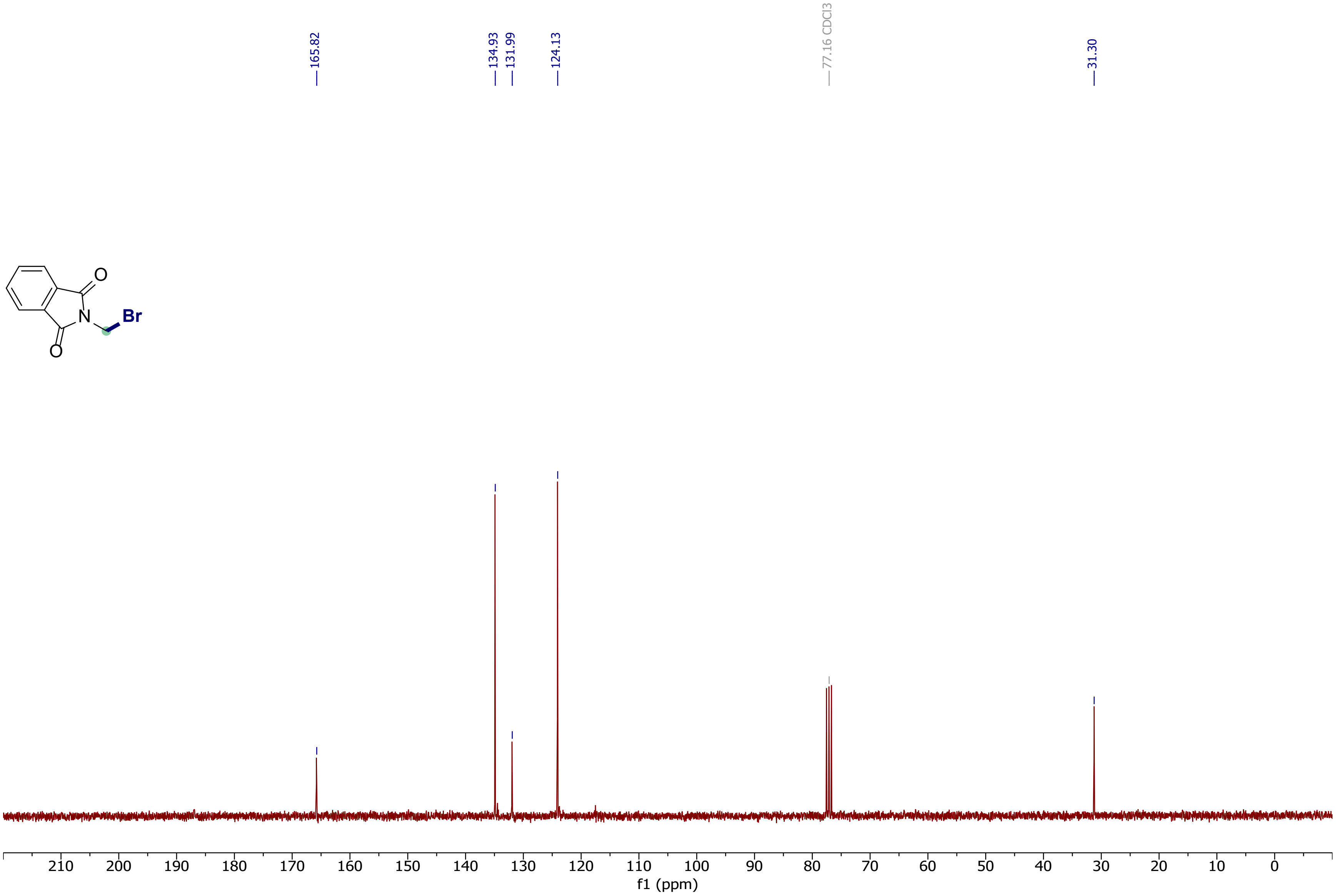
Br
165.82
134.93
131.99
124.13
77.16 CDCl3
31.30
210 200 190 180 170 160 150 140 130 120 110 100 90 80 70 60 50 40 30 20 10 0
f1 (ppm)

3-(4-nitrophenyl)propanenitrile synthesis (53)

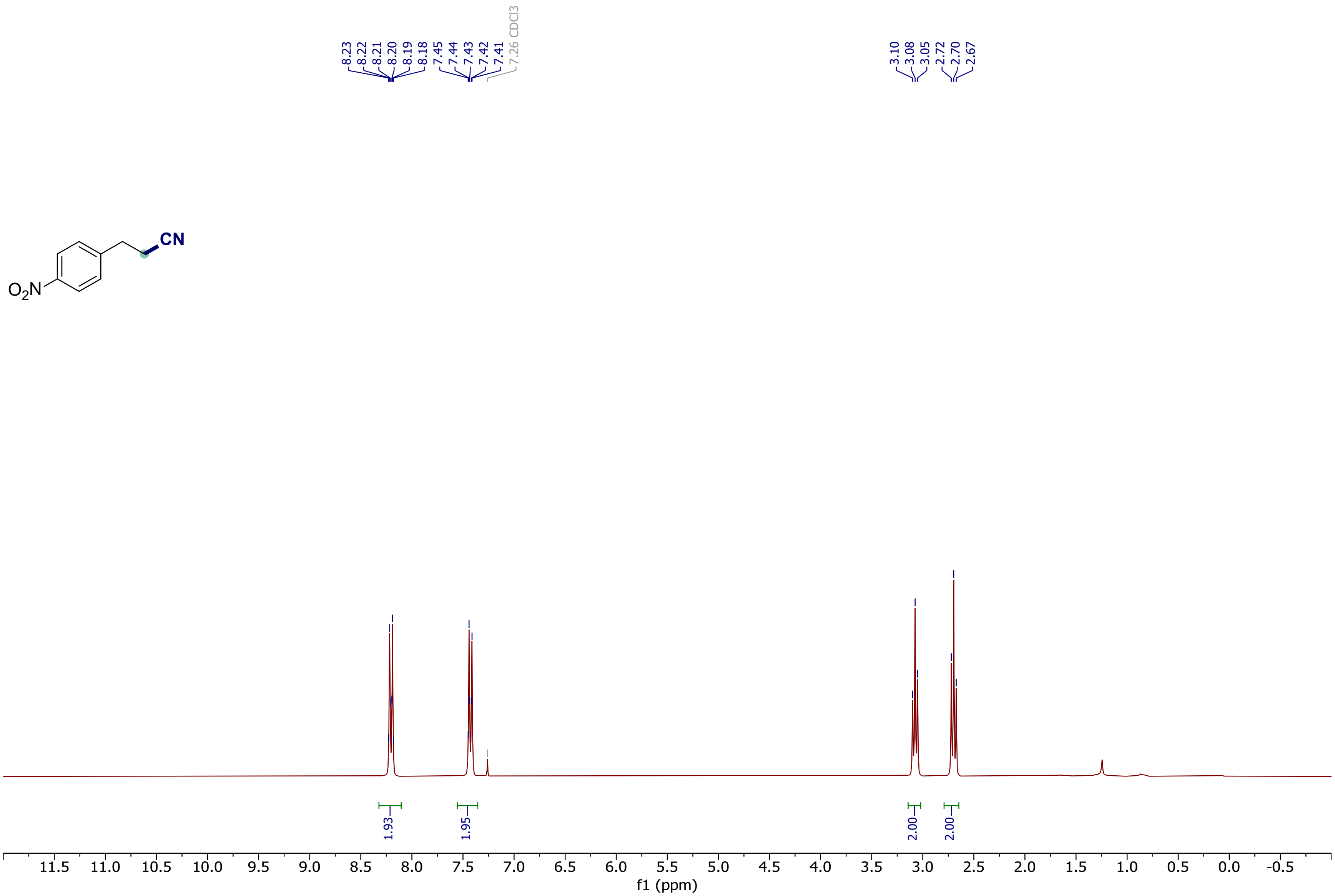

2-(azidomethyl)isoindoline-1,3-dione (54)

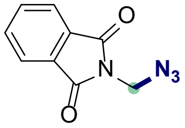

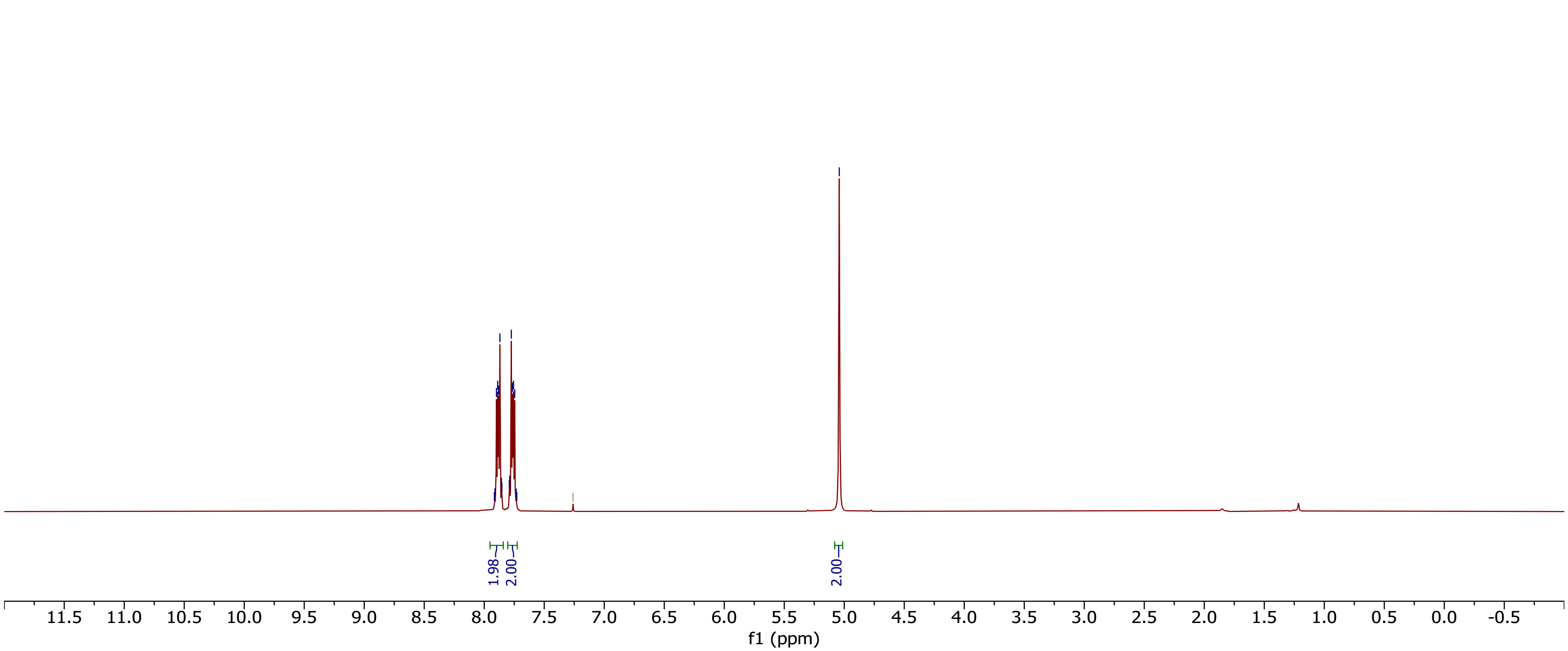

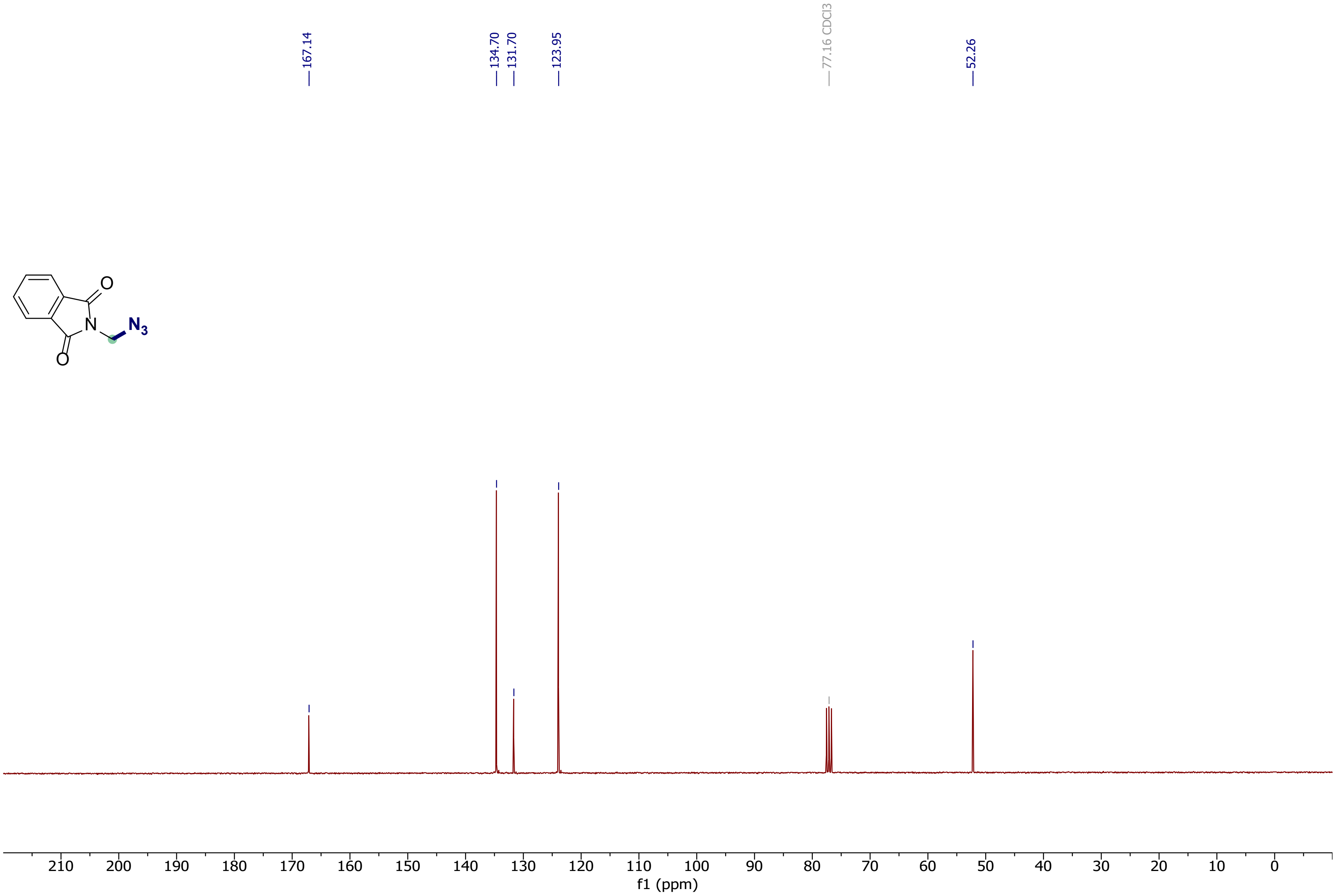
167.14
134.70
131.70
123.95
77.16 CDCl3
52.26
O
N
N3
O
210
200
190
180
170
160
150
140
130
120
110
100
90
80
70
60
50
40
30
20
10
0
f1 (ppm)

8-bromooctyl nitrate (55)

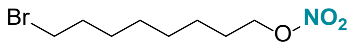

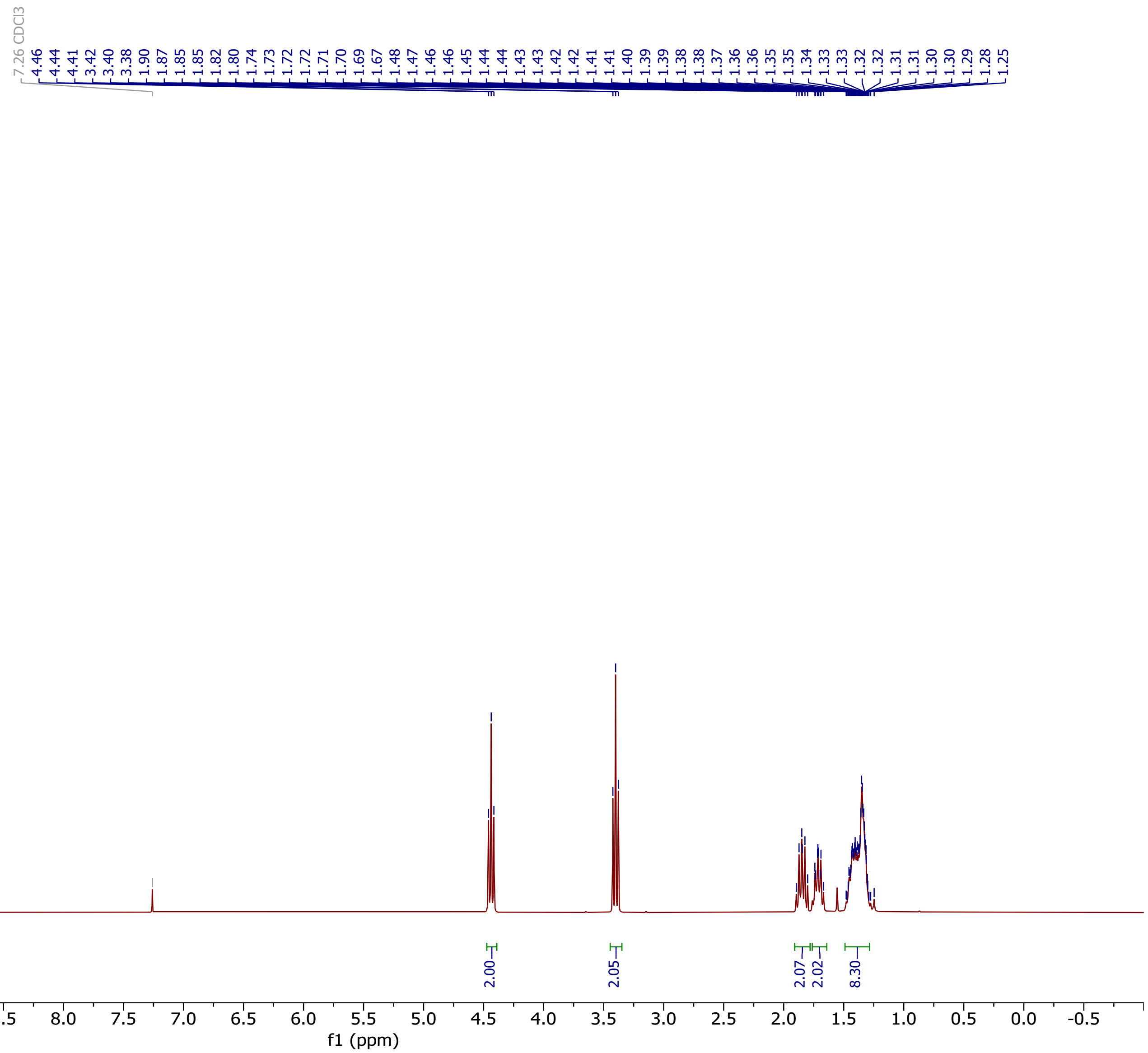

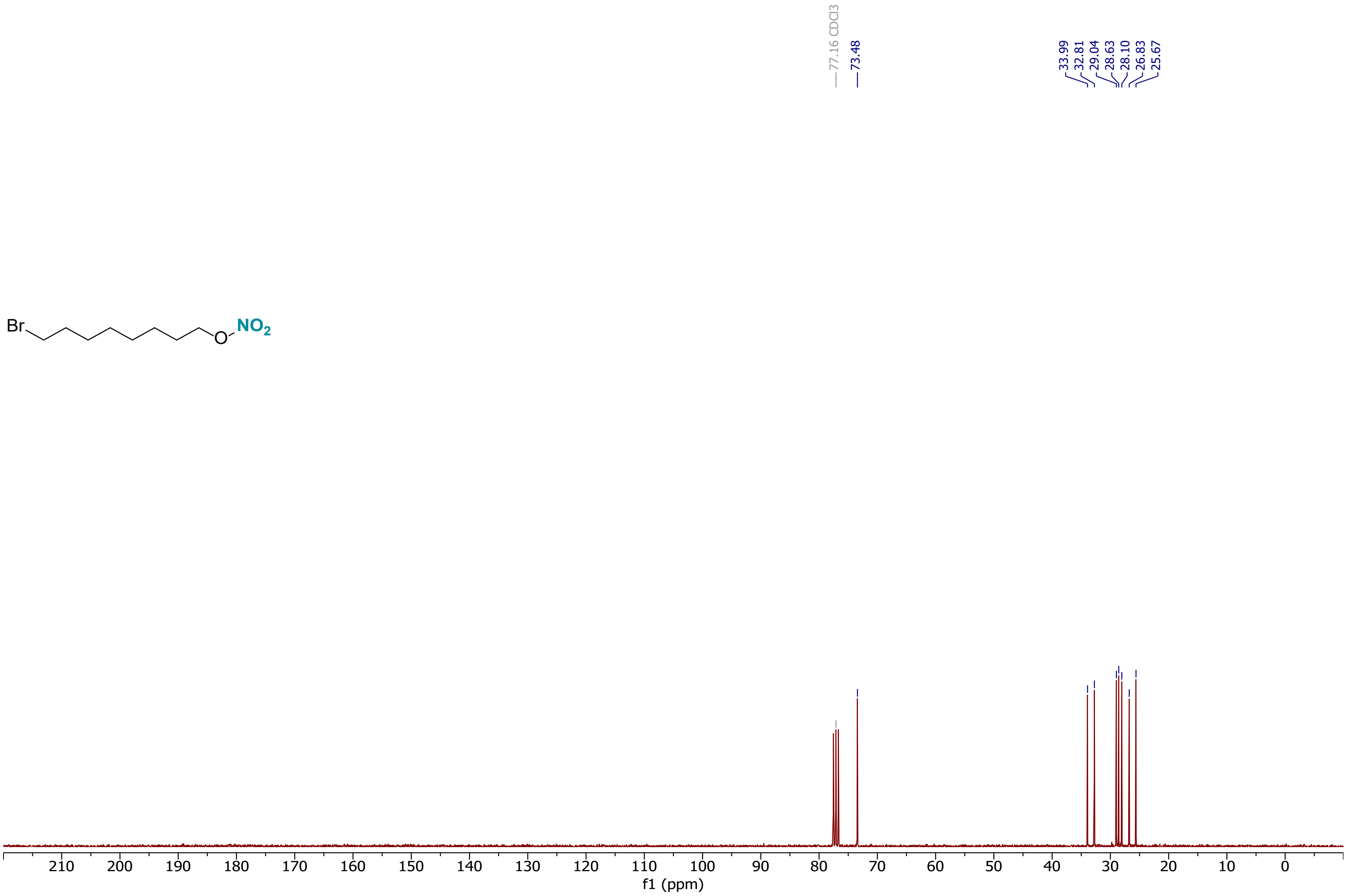

Br
O
NO2
77.16 CDCl3
73.48
33.99
32.81
29.04
28.63
28.10
26.83
25.67
210
200
190
180
170
160
150
140
130
120
110
100
90
80
70
60
50
40
30
20
10
0
f1 (ppm)

8-hydroxyoctyl nitrate (56)

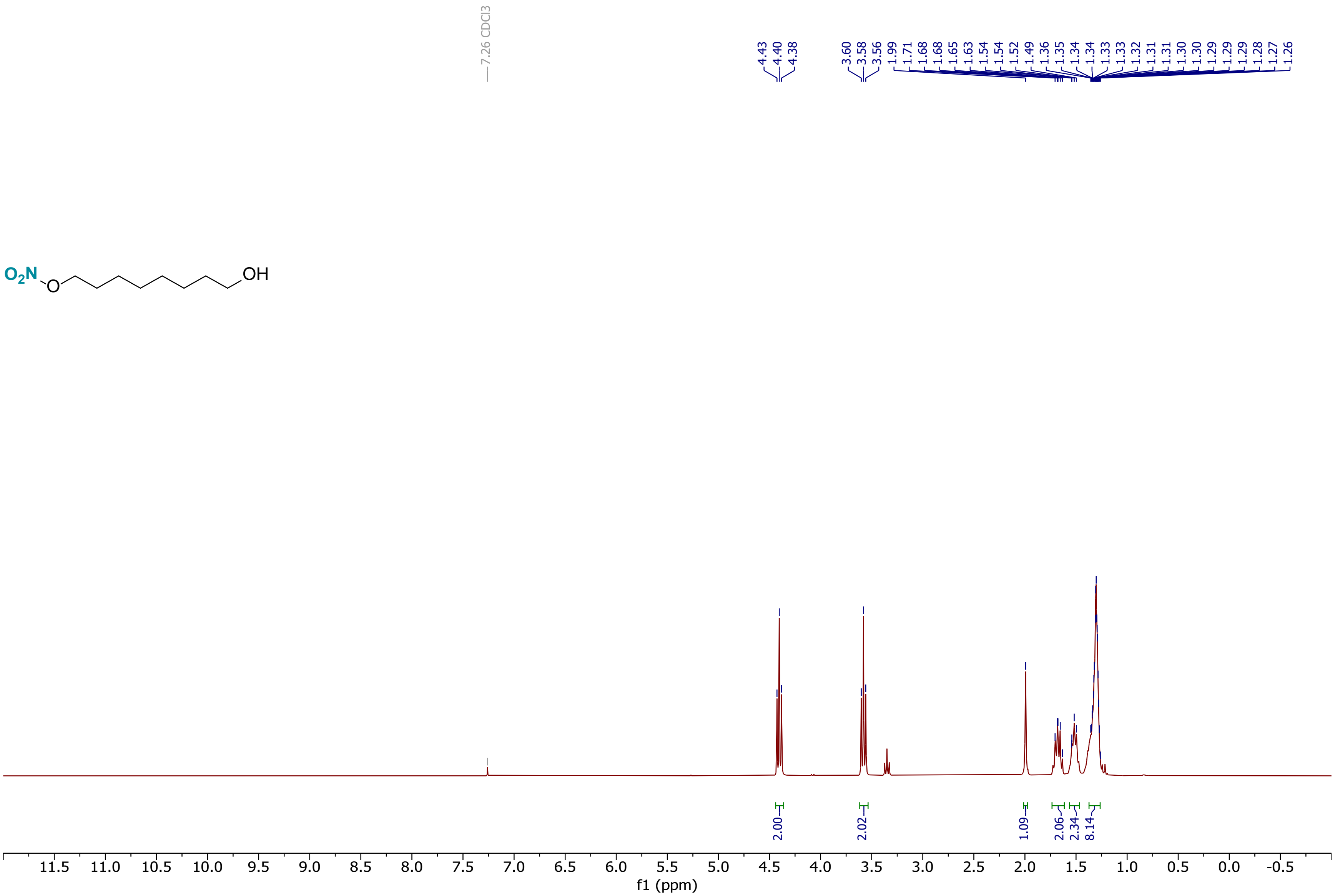

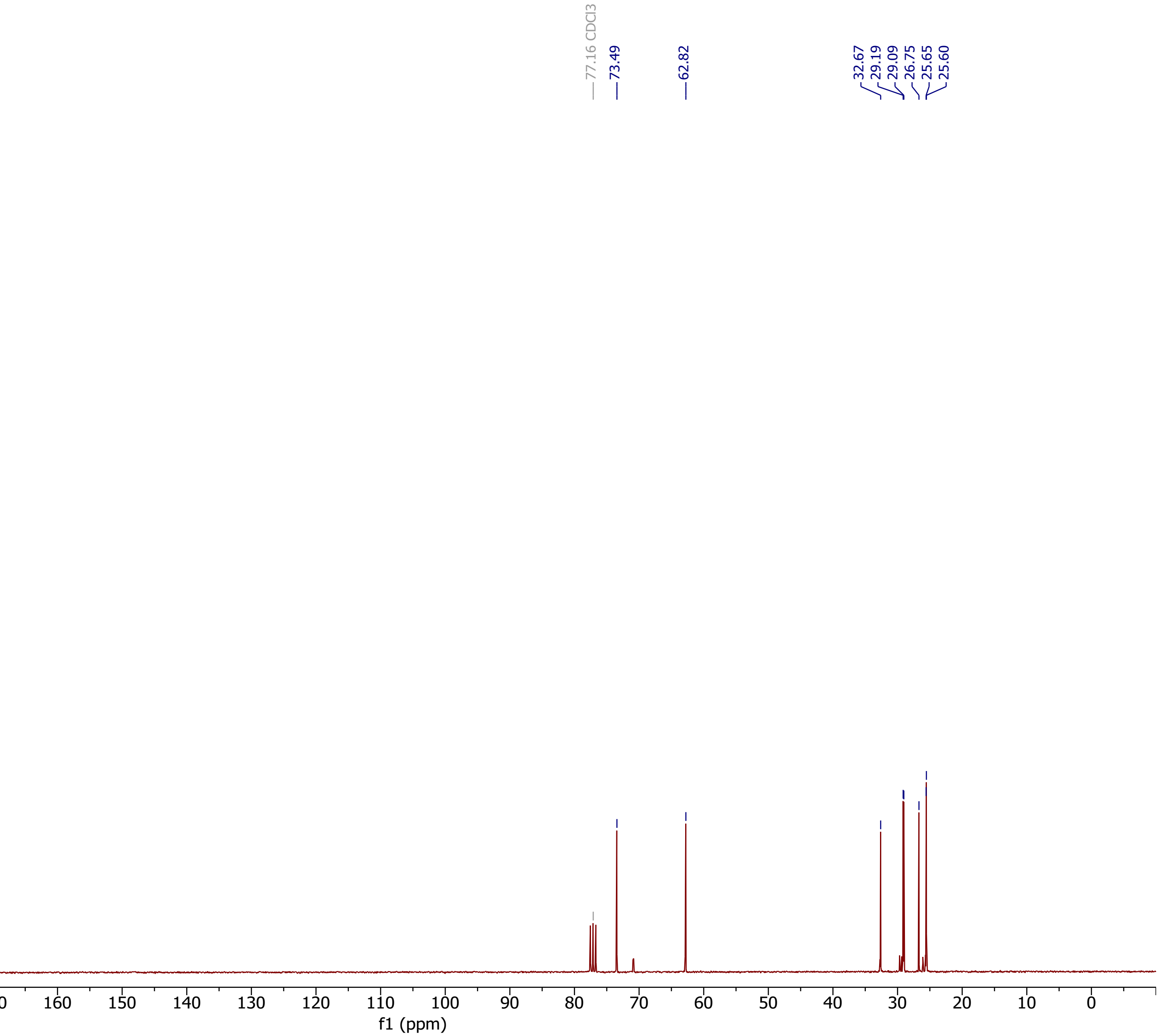

77.16 CDCl3
73.49
62.82
32.67
29.19
29.09
26.75
25.65
25.60

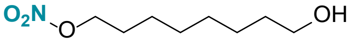

O2N
O
OH

REFERENCES

[1] D. Kong, M. M. Amer, C. Bolm, *Green Chem.* **2022**, *24*, 3125-3129.

[2] A. J. Fernandes, V. Valsamidou, D. Katayev, *Angew. Chem. Int. Ed.* **2024**, *63*, e202411073.

[3] C. J. Easton, A. J. Ivory, C. A. Smith, *J. Chem. Soc., Perkin Trans. 2* **1997**, 503-508.

[4] P. Fr⊘syen, *Phosphorus, Sulfur, Silicon Relat. Elem.* **1997**, *129*, 89-97.

[5] M. I. Kitt, E. Amir, E. R. Sloane, D. G. Fraser, J. E. Cerritelli, C. S. M. Sabanos, J. H. McNeely, J. K. Snyder, L. H. Doerrer, A. B. Beeler, *ACS Catal.* **2024**, *14*, 4799-4806.

[6] N. Iranpoor, F. Kazemi, *Synth. Commun.* **1999**, *29*, 561-566.

[7] N. Iranpoor, F. Kazemi, P. Salehi, *Synth. Commun.* **1997**, *27*, 1247-1258.

[8] Q. Cai, I. M. McWhinnie, N. W. Dow, A. Y. Chan, D. W. C. MacMillan, *J. Am. Chem. Soc.* **2024**, *146*, 12300-12309.

[9] M. Ma, J. Feng, R. Li, S.-W. Chen, H. Xu, *Bioorg. Med. Chem. Lett.* **2015**, *25*, 2773-2777.

[10] J. R. Hwu, K. A. Vyas, H. V. Patel, C.-H. Lin, J.-C. Yang, *Synthesis* **1994**, *1994*, 471-473.

[11] J. Dormer, R. B. Moodie, *J. Chem. Soc., Perkin Trans. 2* **1994**, 1195-1200.

[12] Y. Zheng, Q.-Q. Hu, Q. Huang, Y. Xie, *Org. Lett.* **2024**, *26*, 3316-3320.

[13] S. Patra, I. Mosiagin, R. Giri, T. Nauser, D. Katayev, *Angew. Chem. Int. Ed.* **2023**, *62*, e202300533.

[14] I. Mosiagin, A. J. Fernandes, A. Budinská, L. Hayriyan, K. E. O. Ylijoki, D. Katayev, *Angew. Chem. Int. Ed.* **2023**, *62*, e202310851.

[15] F. Jia, A. Li, X. Hu, *Org. Lett.* **2023**, *25*, 4605-4609.

[16] Q.-S. Gu, D. Yang, *Angew. Chem. Int. Ed.* **2017**, *56*, 5886-5889.

[17] I. H. Sánchez*, M. I. Larraza, F. Basurto, R. Yafñez, S. Avila, R. Tovar, P. Joseph-Nathan, *Tetrahedron* **1985**, *41*, 2355-2359.

[18] H. Fan, H. Sun, X. Peng, *Chem. Eur. J.* **2018**, *24*, 7671-7682.

[19] S. R. Salman, F. S. Kamounah, *Magn. Reson. Chem.* **1987**, *25*, 966-969.

[20] S. Saha, J. N. Moorthy, *J. Org. Chem.* **2011**, *76*, 396-402.

[21] O. S. Radchenko, E. N. Sigida, N. N. Balaneva, P. S. Dmitrenok, V. L. Novikov, *J. Heterocycl. Chem.* **2011**, *48*, 209-214.

[22] C. N. Kona, R. Oku, S. Nakamura, M. Miura, K. Hirano, Y. Nishii, *Chem* **2024**, *10*, 402-413.

[23] C. J. Erickson-Miller, D. F. Eppley, J. Jenkins, J. I. Luengo, N. Liu, A. T. Price, A. N. Shaw, S. Visonneau, K. Wiggall, **T***hrombopoietin mimetics*, US2004019190A1, **2004**.

[24] A.-K. Bauer, J. Conrad, U. Beifuss, *Org. Biomol. Chem.* **2023**, *21*, 8003-8019.

[25] M. Gon, K. Tanaka, Y. Chujo, *Chem. Eur. J.* **2021**, *27*, 7561-7571.

[26] V. Jamsheena, C. K. Mahesha, M. N. Joy, R. S. Lankalapalli, *Org. Lett.* **2017**, *19*, 6614-6617.

[27] a) Z. Zhang, Z. Zhao, M. Liu, H. Liu, Q. Li, J. Xiang, T. Wu, B. Han, *Green Chem.* **2022**, *24*, 9763-9771; b) W. Liao, Jr., S.-Y. Lin, Y.-S. Kuo, C.-F. Liang, *Org. Lett.* **2022**, *24*, 4207-4211.

[28] C. K. De, F. Pesciaioli, B. List, *Angew. Chem. Int. Ed.* **2013**, *52*, 9293-9295.

[29] M. Mascal, L. Yin, R. Edwards, M. Jarosh, *J. Org. Chem.* **2008**, *73*, 6148-6151.

[30] S. Chercheja, S. Daum, H.-G. Xu, F. Beierlein, A. Mokhir, *Org. Biomol. Chem.* **2019**, *17*, 9883-9891.

[31] S. Li, D. Ma, *J. Org. Chem.* **2024**, *89*, 6626-6630.

[32] L. Kaur, M. Kumar, V. Bhalla, *Green Chem.* **2023**, *25*, 5240-5246.
[33] L. Wang, E. Ji, N. Liu, B. Dai, *Synthesis* **2016**, *48*, 737-750.
[34] S. Endres, A. Hacker, E. Noack, G. Kojda, J. Lehmann, *Eur. J. Med. Chem.* **1999**, *34*, 895-901.
[35] C.-P. Yu, Y. Tang, L. Cha, S. Milikisiyants, T. I. Smirnova, A. I. Smirnov, Y. Guo, W.-c. Chang, *J. Am. Chem. Soc.* **2018**, *140*, 15190-15193.
[36] P. Strazzolini, A. G. Giumanini, A. Runcio, M. Scuccato, *J. Org. Chem.* **1998**, *63*, 952-958.
[37] Y. Zhu, X. Li, X. Wang, X. Huang, T. Shen, Y. Zhang, X. Sun, M. Zou, S. Song, N. Jiao, *Org. Lett.* **2015**, *17*, 4702-4705.
